# Dataset for multi-channel surface electromyography (sEMG) signals of hand gestures

**DOI:** 10.1016/j.dib.2022.107921

**Published:** 2022-02-04

**Authors:** Mehmet Akif Ozdemir, Deniz Hande Kisa, Onan Guren, Aydin Akan

**Affiliations:** a Izmir Katip Celebi University, Faculty of Engineering and Architecture, Department of Biomedical Engineering, Cigli, Izmir 35620, Turkey; b Izmir University of Economics, Faculty of Engineering, Department of Electrical and Electronics Engineering, Balcova, Izmir 35330, Turkey

**Keywords:** Biomedical signals, Biosignals, Classification, Data, Electromyography (EMG), Gesture, Movement, Muscle, Deep Learning, Machine Learning

## Abstract

This paper presents an electromyography (EMG) signal dataset for use in human-computer interaction studies. The dataset includes 4-channel surface EMG data from 40 participants with an equal gender distribution. The gestures in the data are rest or neutral state, extension of the wrist, flexion of the wrist, ulnar deviation of the wrist, radial deviation of the wrist, grip, abduction of all fingers, adduction of all fingers, supination, and pronation. Data were collected from 4 forearm muscles when simulating 10 unique hand gestures and recorded with the BIOPAC MP36 device using Ag/AgCl surface bipolar electrodes. Each participant's data contains five repetitive cycles of ten hand gestures. A demographic survey was applied to the participants before the signal recording process. This data can be utilized for recognition, classification, and prediction studies in order to develop EMG-based hand movement controller systems. The dataset can also be useful as a reference to create an artificial intelligence model (especially a deep learning model) to detect gesture-related EMG signals. Additionally, it is encouraged to use the proposed dataset for benchmarking current datasets in the literature or for validation of machine learning and deep learning models created with different datasets in accordance with the participant-independent validation strategy.

## Specifications Table

**Table:** 

Subject	Engineering> Biomedical Engineering
Specific subject area	Hand-Gesture Recognition, EMG signal classification, Signal Processing
Type of data	Signals Survey Form (see *Table 1*) Annotations
How the data were acquired	A demographic survey was done to choose proper individuals for data collection and collect background information about participants' data before the signal recording. Then, EMG signals were acquired from a 4-channel MP36 model BIOPAC device (BIOPAC Co., USA). MP36 Data Acquisition Unit includes 4 certified human-safe input channels and built-in amplifiers and uses BSL 4 software. In the data collection stage, SS2LB electrode lead sets with smart and simple sensors connectors, and non-invasive 3 M brand Red Dot monitoring Ag/AgCl surface electrodes (3.3 × 3.99 cm sizes) with disposable and highly adhesive were used. Before electrodes were attached to the forearm, the skin surface is cleaned by alcohol to remove dead cells and oils. The approximate locations of four forearm muscles were determined by an expert physician, and then BIOPAC system electrode gel (GEL1) was applied to the skin. The sensors were calibrated when the electrodes were placed on the participant. The procedure slide was begun with recording simultaneously. The whole data were collected in the same environment and conditions by minimizing environmental conditions to prevent noises.
Data format	Raw Filtered
Description of data collection	The sEMG data was collected from the dominant forearm of all participants. The recording process consists of the participant observing the gestures on the slide screen and acting their reactions, simultaneously. EMG data was recorded at a 2 kHz sampling frequency. It was collected in the form of both raw and filtered. For filtered signals, EMG data was online filtered with a sixth-order Butterworth bandpass filter with a frequency range of 5–500 Hz and a second-order notch filter at 50 Hz for the elimination of noises like motion artifacts, high-frequency noise, and power line interference by using BIOPAC Software BSL 4.0 filtering options. The amplitude of the EMG signals was in the range of −10 to 10 mV.
Data source location	Institution: Izmir Katip Celebi University, Department of Biomedical Engineering City/Town/Region: Izmir, Cigli, Balatcik Country: Turkey Latitude and longitude (and GPS coordinates, if possible) for collected samples/data: 38°30′38.1”N 27°02′01.8”E
Data accessibility	Repository name: Mendeley Data Data identification number: 10.17632/ckwc76xr2z.2 Direct URL to data: http://dx.doi.org/10.17632/ckwc76xr2z.2 https://data.mendeley.com/datasets/ckwc76xr2z/2

## Value of the Data

• This dataset is presented as large data which includes 4-channel time-series of sEMG signals that are recorded from 40 participants performing 10 unique hand gestures.
• The data is valuable for the Biomedical Signal Processing Society, especially for the tasks of hand-gesture detection and classification.
• This data can be useful for creating artificial intelligence models, especially in deep learning research to predict hand gestures.
• EMG signals are recorded in repetitive 5 cycles and 4-channel, so this can be useful when requires large data such as building deep learning models. It is also useful as a reference dataset for benchmarking models.
• The provided data is filtered from other noises so the researchers can focus only on the muscle-generated noiseless EMG signals. Raw data is also provided for advanced task-specific filtering processes.
• Participants were subjected to a comprehensive survey before signal recording, many negative factors that could affect the quality of the signal were minimized, and the elements that could threaten the participant's health were prevented.

## 1 Data Description

Hand gesture classification is a critical research topic for hand gesture-based systems with great benefits in human-computer interaction (HCI) [1]. Especially when these systems using HCI are combined with a data source such as electromyography (EMG) that provides very clear and precise information about movement, successful results are obtained with the help of artificial intelligence (AI) [2]. Hand gestures can be detected by algorithms developed based on machine learning (ML), deep learning (DL), and fuzzy logic (FL) in the field of AI approaches [3], [4], [5]. The methods mentioned may require different numbers of data due to their structure. For example, while better results can be obtained for ML without a large amount of data, huge datasets are needed to build a DL model. Since the hand is an inherently complex and high degree-of-freedom limb, there are many hand movements that can be further diversified [6]. Another point is the number of participants in the dataset. It is known that the dataset can be handled and used in the various models when it has larger and more diverse [7]. Along with these, the choice of used channels when collecting EMG data is also a critical point. Because, while more channels provide more information and a more precise judgment about hand movement, at the same time, more channel information can sometimes cause unnecessary computational load and reduce the training speed [8].

Various EMG-based datasets have been published to perform human-computer or human-robot interaction [9,13]. These data sets are used as input data in AI algorithms. Despite the diversity of data in the literature, there is an irregularity in the parameters that affect the amount of data. To obtain a successful human-computer interaction, the AI model should be trained with EMG-based data including the optimal number of sensors, subjects, and gestures [12]. Classification performance may not be sufficient when the number of gestures increases, the number of participants is insufficient, or the unsuitable electrodes (i.e., few or insufficient numbers, and incorrect placement). Also, while many important datasets are rich in terms of the number of participants, they ignored the gender distribution [9,^11^,12]. The data of male participants were mostly used. This imbalance may cause some of the data to be unused and bias the trained model. The proposed dataset and survey may be more useful for researchers who want to investigate gender-related parameters. Another parameter is how long the movements are performed. In our study, 6 s was set as the time to perform a movement because of anticipating the delays that may occur while performing the movement. It also may be useful for the examining transition between the neutral stage and gesture moment. It has been observed that this period was set as a short period of time, on average 3 s, in [9] and [12] which are among the popular datasets. Another important point is the number of repetitions. Moreover, while the repetitive signal of the same participant can contribute significantly to the creation of an AI model, the EMG signals may deviate from the representation of the movement with increasing repetition numbers as the excessive repetition time will tire the muscles. This may not be a problem when the measurements are spread over days or long rest periods, but it is important that the recording process is of optimum difficulty and time for the participant. Also, the shortness of the measurement period and standardization of the environmental conditions are important for the uniformity of the measurements. Finally, it is important to keep the number of channels at the optimum. This is important for balancing the computational load and obtaining correct information from the muscles responsible for the related movements. Also, some of the available datasets offer a non-adjustable electrode placement for the participants [13]. Even so, the muscle lengths and locations of the participants may be partially different. For this reason, the muscles must be determined specifically for each participant by an expert physician. Additionally, it is important that a machine learning or deep learning model trained with current datasets should be validated with different datasets in accordance with the participant-independent validation strategy. Thus, the success of the trained model in testing the data of new participants can be compared as a measure of feasibility. For these reasons, there is still a need for new EMG-based datasets for hand gestures that contain accurate information and are well prepared.

**Fig. 1:**
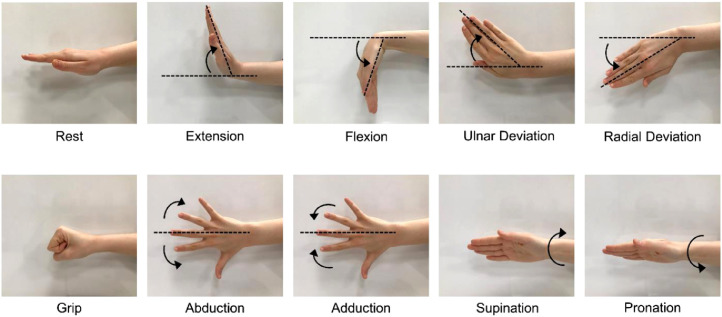
The performed ten hand gestures.

**Fig. 2:**
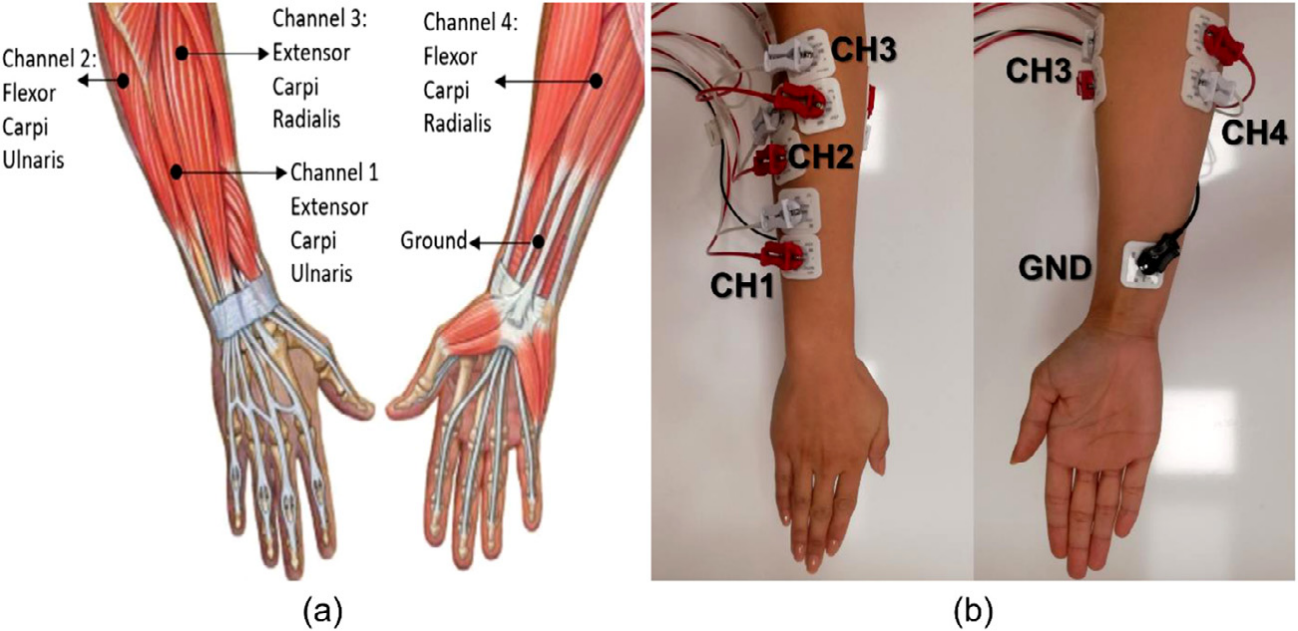
Location of channels and ground; (a) posterior and anterior views of the right upper limb, and (b) 4-channel electrode placement with a ground electrode.

**Fig. 3:**
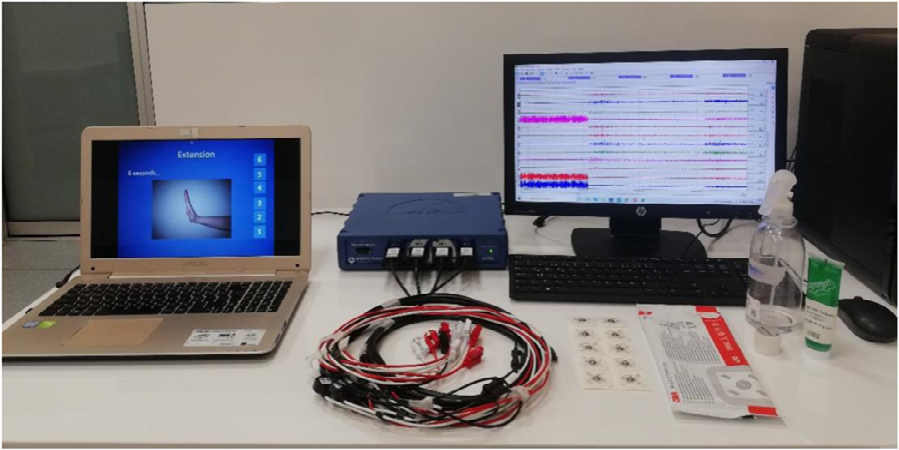
Measurement system: 4-channel MP36 BIOPAC device and the computer systems.

In this study, a novel surface EMG dataset was created using appropriate numbers of channels, participants, and gestures. This dataset consists of the most used basic hand gestures and wrist movements in daily life. The ten utilized hand gestures are rest or neutral state, extension of the wrist, flexion of the wrist, ulnar deviation of the wrist, radial deviation of the wrist, grip, abduction of all fingers, adduction of all fingers, supination, and pronation as shown in Fig. 1. Although there are some datasets structured on various channels like single, 8, or 24, a 4-channel data system has been proposed to obtain optimum information with a minimum number of electrodes as shown in Fig. 2. While a movement can be controlled by more than one muscle, more channels increase the computational complexity. Further, the anatomical locations of our suggested 4-channel system have been approved by the expert physician. The approximate location of electrodes is shown in (a) part of Fig. 2, its placement on a right-handed participant's limb is shown in Fig. 2(b), and the experimental setup is shown in Fig. 3. Moreover, in this dataset more participants have been reached compared to the recent datasets. Also, the presented dataset was recorded at a high quality of 2 kHz sampling rate. Additionally, EMG signals were collected from each participant within 5 repetitions. Thus, the presented dataset can be used in applications that are open to validation and require a lot of data to build an AI model [14]. The presented dataset may be a reference for validation methods to be developed in accordance with the patient-independent validation strategy.

The proposed data repository on Mendeley Data contains 6 different file types:

**(i)** The *“survey_responses.xlsx”* file is the list of answers given by the anonymous participants to the demographic survey questions and the participant information except for personal information. Survey questions and possible answers are listed in Table 1. It can be used for various analyzes and to examine the effect of answers on the signals in classification problems.

**Table 1:** Questions of the survey.

Question No.	Questions	Possible Answers
1	What is your name?	String value
2	What is your gender?	• Female • Male
3	What is your age?	Numeric value
4	What is your weight?	Numeric value in kilograms
5	What is your height?	Numeric value in meters
6	Do you have any muscle disease?	• Yes • No
7	Do you use any muscle related medication?	• Yes • No
8	Have you taken any medication in the last 24 h?	• Yes • No
9	Have you used any stimulus in the last 24 h? (Alcohol etc.)	• Yes • No
10	What is your dominant hand?	• Right • Left • Both
11	Do you have a pacemaker or brain pacemaker?	• Yes • No

**(ii)** The *“4CH_RAW+4CH_FILTERED_EMG.gtl”* file is the BSL software setting file that provides for collecting 4-channel EMG signals in the shape of both raw and online filtering. The filtering parameters are set as the cutoff frequencies of 5–500 Hz sixth-order Butterworth bandpass filter, and a second-order 50 Hz notch filter by using the BIOPAC BSL 4.0 software.

**(iii)** The *“procedure.pptx”* file is the time-sensitive signal collection procedure file that has been performed by participants.

**(iv-v)** In the *“sEMG-dataset”* folder, there are files containing the sEMG data of the participants and this file consists of two subfolders as *“raw”* and *“filtered”.* The *“raw”* folder consists of raw sEMG signals of 40 participants. The *“filtered”* folder consists of filtered sEMG signals of 40 participants. Both folders consist of two subfolders as *“csv”* and *“mat”*. While the *“csv”* folders consist of only four-channel sEMG *a“data”* variable of 40 participants as *“.csv”* file format, *“mat”* folders consist of 40 participants' data and each participants' file includes;

- ***“data”*** variable which consists of 4-channel sEMG signals, and it has 4 × 1,280,000 dimension (4 channel x 1,280,000 signal length = 640 s data collection length x 2000 sampling frequency),
- ***“fs”*** variable which is sampling rate as a “2000” hertz,
- ***“iD”*** variable which indicates the participant iD as “1 to 40”,
- ***“isi”*** variable which is sampling interval as “0.5”,
- ***“isi_units”*** variable is the sampling interval unit as “ms",
- ***“labels”*** variable indicates the related channels' labels,
- ***“start_sample”*** variable which is the starting time of the signal recording as “0”, and
- ***“units”*** variable which is the sEMG data units as “mV”.

These files are ordered with *“participant ID”*. While *“.csv”* files are provided for easy importation in Python *“.mat”* files are stored in MATLAB® in a way that can be easily imported.

**Fig. 4:**
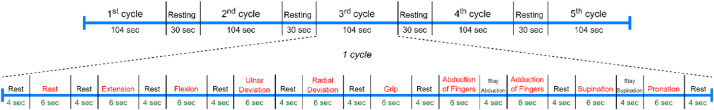
The timeline of the recording.

**(iv)** The *“EMG_gesture_segmentation.m”* file is the main code structure that provides automated segmentation of gesture moments from each participant's four-channel sEMG data. It is written in MATLAB® and each step of the code is explained in detail in itself and is suitable for the EMG signal analysis process. It provides loading the sEMG data from the disk and segmentation of gesture moments. Also, it creates a suitable framework for future analysis. It is basically segments both multi-channel and channel-based sEMG data of each gesture moment based on the sampling frequency and the desired segment length for each cycle. Segmentation operations are executed according to the timeline given in Fig. 4. An additional **Algorithm 1.** is presented in the Supplementary Material for better understanding. The data recording procedure is explained in detail in the following section.

## 2 Experimental Design, Materials, and Methods

The whole data collection process was practiced according to the Helsinki Declaration. Firstly, before the EMG recording, the details of the procedure to be performed were explained to all participants. A survey including 11 questions in Table 1 such as whether the participants are healthy, whether they have muscle or heart disease, whether they have a heart or brain pacemaker, and the status of drug or alcohol usage was applied to the participants in order to prevent the recording process from incorrect measurement and health conditions. Although there is no age limit in the dataset, age and gender distribution were kept equal at a certain standard deviation level to ensure consistency within the dataset. 40 healthy participants have between the ages of 18–29 and are equal in gender distribution. The average age for all participants was 22.63±2.1. The average weight of females and males were 58.7 ± 9.54 kg and 76.3 ± 13.8 kg, and their average heights were 166.4 ± 5.74 cm and 178.4 ± 4.44 cm, respectively. EMG data were collected from each participant's dominant hand for HCI or biorobotics applications as mentioned in recent datasets [10,12]. Of the 40 participants, 3 participants are left-handed (2 females, one male), one participant (male) uses both hands (ambidextrous), and the remaining are right-handed. The participant using both hands was asked which hand one used the most, and data were obtained from one's right arm in accordance with one's answer.

The amplitude of EMG signals is usually a low level. Therefore, it was aimed to provide data from the muscles closer to the skin surface to obtain noiseless data. For this reason, four distinct surface muscles close to the skin surface were specified by the expert so that the best quality signals can be received during the determined hand gestures. These muscles are *extensor carpi radialis, flexor carpi radialis, extensor carpi ulnaris*, and *flexor carpi ulnaris*. The 4-channel electrode system was utilized to collect data from these muscles. The area with the least amount of electrical activity near the wrist and tendon was chosen for the ground electrode as shown in Fig. 2(a,b).

BIOPAC device (BIOPAC Co., USA) is an appropriate system for the collection, analysis, and preparation of biopotential and physiological signals of participants on the computer. This system possesses a multichannel recording structure that can collect data simultaneously. In this data collection process, completely harmless and non-invasive Ag/AgCl surface electrodes (3.3 × 3.99 cm sizes 3 M brand Red Dot monitoring electrodes with disposable and highly adhesive) were placed on the approximate location of four specified muscles utilizing a 4-channel MP36 model BIOPAC EMG system shown in Fig. 3. Before the electrodes were placed, in order to reduced the skin impedance, the surface of the arm was wiped with alcohol for lifting dead cells and oil. Then the electrode gel was used to further reduce the skin impedance discrepancy.

The experimental recording timeline is demonstrated in Fig. 4. The entire process of collecting an EMG record from a participant took 640 s which has included repetitive 5 cycles. All cycles contain ten hand gestures and resting time except the fifth cycle which has only ten gestures. One cycle was 104 s and between two cycles, there were 30 s long rest periods owing to prevent fatigue. The recording process consists of the participants observing the gestures on the slide and acting their reactions simultaneously. Before the recording process started, a short training period was conducted to perform the gestures correctly, and all the gestures were shown to the participants and asked to perform them. Subjects sit upright and comfortably throughout the procedure. They extend their arms parallel to the ground and hold them in the air throughout the cycle. The procedure begins with the rest position. There are 4 s rests before and after each gesture to highlight the transitions between gestures. In the rest position, the hand is straight, parallel to the ground, and the fingers are neither too close nor too far apart. After a 4 s rest, another 6 s rest movement is performed to be used as the neutral state. Then comes a rest of 4 s. Except for the 2 passes at the end of the loop, this 4 s rest takes place after each 6 s gesture. After the neutral rest movement, extension, flexion, ulnar deviation, and radial deviation are performed respectively. During these gestures, the hand remains open, straight, and the fingers are close to each other and as parallel as possible. Participants are asked to perform these gestures in the best way possible anatomically and in accordance with the visuals. For these four gestures, they are asked to keep the angle between the forearm and the hand as small as possible. Then the grip movement is performed. Subjects are asked to grip their hands normally, neither too tight nor too loose. After the grip, it is returned to rest, and then abduction of all fingers is performed. In this abduction, the fingers are kept as far apart as possible, and the fingers remain tense. Then, unlike other gestures, when 6 s is over, there is no rest for the transition and they are asked to stay in the abduction position for 4 more seconds. After 4 s, they are asked to switch from abduction position to adduction of all fingers position. Fingers stay as close together as possible for 6 s. When this adduction is finished, it is returned to rest again. Then, keeping the fingers as close together as possible and the hand straight, supination is performed with the palm facing upwards. Supination is done and current position is retained, and the 6 s period is expected to expire. Then, as in the previous stage, is not returned to the rest, and the supination movement remains for 4 more seconds. When the 4 s is over, the 6 s gesture period begins and the supination moves to the pronation movement. When the movement time is over, returns to rest and stays in this position for 4 s. Then the slide shows the completion of the cycle and the long rest period of 30 s begins. Subjects are asked to leave their hands free and put them down and rest. After 30 s, the next cycle starts. In this way, the same cycle is repeated 5 times.

**Fig. 5:**
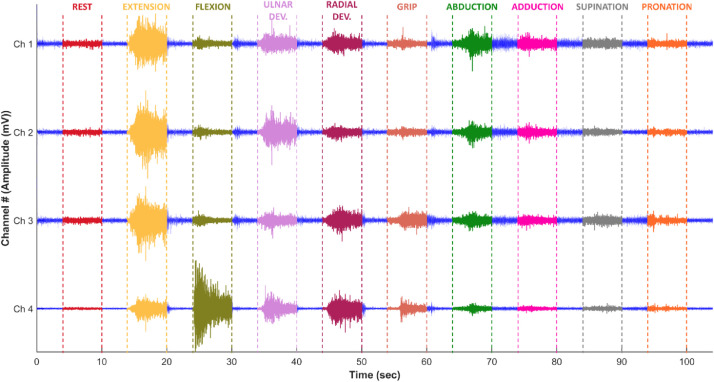
An example plotting of four-channel sEMG signal of 10 hand gestures.

640 s‑long EMG data were recorded at a 2 kHz sampling frequency as both raw and filtered formats by using the procedure described in detail above. The amplitude of the recorded EMG signals was in the range of −10 to 10 mV, and they were filtered with a sixth-order Butterworth bandpass filter with a frequency range of 5–500 Hz and a second-order Notch filter with a bandstop frequency of 50 Hz to the elimination of noises like e.g. motion artifacts, high-frequency noise, and power line interference [15,16]. The filtering process was carried out online using the BIOPAC BSL 4.0 software. An example plotting of the obtained sEMG signals is shown in Fig. 5.

**Fig. 6:**
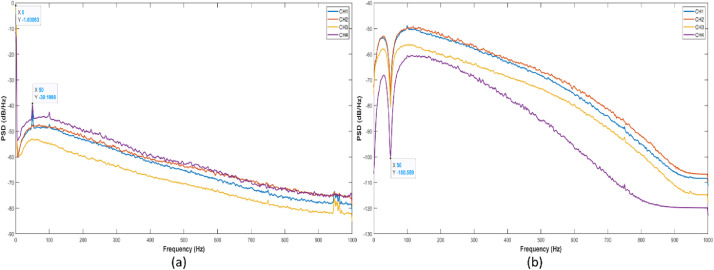
Power spectral density estimate plottings of sEMG signals based on Welch's Method: (a) raw sEMG data and (b) filtered sEMG data.

Measurements were taken in a quiet laboratory environment to prevent noise that may occur on the signal. Also, in order to prevent distortion and noise that may occur in the measured signal, it was requested that take off the metal items such as belts, earrings, buckles, mobile phones, etc. The recorded data of each participant has *1,280,000* samples (*640* *s long x 2* *kHz sampling rate*). Finally, the power spectral density plottings obtained by Welch's Method for both raw and filtered data to observe the frequency/power distributions in the signals are given in Fig. 6. Thanks to the filtering procedure, the DC component and the harmonics originating from the main source at 50 Hz, which are clearly visible in Fig. 6(a), are significantly eliminated in Fig. 6(b). We kindly encourage researchers to perform new and advanced task-specific filtering processing over raw files and to analyze filtered signals.

## Ethics Statements

The authors declare that the work described has been carried out in accordance with the Declaration of Helsinki of the World Medical Association revised in 2013 for experiments involving humans. The ethical committee approval was obtained from **Izmir Katip Celebi University Committee of Scientific Research and Publication Ethics in Science and Engineering** with the approval code of **2020-BA2FEN-0001** and dated **19/10/2020**. In the ethical committee approval form, there is an approval text for sharing the collected EMG data and the responses to the survey with the scientific community in a way that keeps the participant's personal information confidential. Since the data involved human subjects, volunteer participants were informed about why and how to conduct the research. Informed consent has been obtained from each subject participating in the study. Before the experiment started, the purpose of the data collection and how the recording procedure will be carried out are explained step-by-step with visual materials. Then, a voluntary participant survey and consent form was applied to the participants. An information form was given stating that their participation in this data collection process is completely voluntary and that they have the right not to participate during the process, or to leave anywhere or instantly after participating.

## CRediT Author Statement

**Mehmet Akif Ozdemir:** Project administration, Conceptualization, Methodology, Data curation, Software, Validation, Visualization*,* Writing - review & editing, Funding acquisition; **Deniz Hande Kisa:** Data curation, Methodology, Investigation, Visualization, Writing - original draft; **Onan Guren:** Funding acquisition, Supervision; **Aydin Akan:** Funding acquisition, Supervision.

## Declaration of Competing Interest

The authors declare that they have no known competing financial interests or personal relationships that could have appeared to influence the work reported in this paper.

## Appendix Supplementary materials

Image, application 1

Image, application 2

Image, application 3

Image, application 4
