# Supplementary material for: Dataset for multi-channel surface electromyography (sEMG) signals of hand gestures
Source: Data Brief. 2022 Feb 4;41:107921. doi: 10.1016/j.dib.2022.107921 (PMC8844426; doi:10.1016/j.dib.2022.107921)
Supplement: Supplementary file 1 [file mmc1.pdf]

T.C. İZMİR KÂTİP ÇELEBİ ÜNİVERSİTESİ
FEN VE MÜHENDİSLİK BİLİMLERİ
BİLİMSEL ARAŞTIRMA ve YAYIN ETİK KURULU

TARİH : **19/10 /2020**

KATILIMCILAR : **Etik Kurul Başkanı Prof. Dr. Adnan KAYA**
Etik Kurul Üyesi Prof. Dr. Şerafettin DEMİÇ
Etik Kurul Üyesi Prof. Dr. Mehmet ÇEVİK
Etik Kurul Üyesi Prof. Dr. Semih ENGİN
Etik Kurul Üyesi Prof. Dr. Nilgül ÇETİN
Etik Kurul Üyesi Prof. Dr. Buket OKUTAN BABA
Etik Kurul Üyesi Prof. Dr. Ramazan SEREZLİ

**FEN VE MÜHENDİSLİK BİLİMLERİ<br>BİLİMSEL ARAŞTIRMA VE YAYIN ETİĞİ KURULU<br>ARAŞTIRMA ONAY FORMU**	
**Araştırmanın Adı**	EMG Sinyalleri ve Yapay Zeka Yöntemleri Kullanılarak El Hareketi Tahmini
**Araştırmanın Niteliği**	Özgün Araştırma
**Araştırmanın Araştırmacıları**	Yürütücü: Mehmet Akif ÖZDEMİR, Araştırmacı: Deniz Hande KISA
**Araştırma Yürütücüsünün İletişim Bilgileri**	**Adresi:** İzmir Katip Çelebi Üniversitesi- Mühendislik ve Mimarlık Fakültesi- Biyomedikal Mühendisliği, Balatçık/Çiğli/İZMİR<br>**e-posta adresi:** makif.ozdemir@ikcu.edu.tr<br>**Telefonu:** 0551 425 92 99
**Araştırmanın Amacı**	EMG tabanlı kontrol sistemlerinde kullanılması amacıyla el hareketi tahminini yapay zeka yöntemleriyle gerçeklemek, yapay zeka modeli oluşturmak.
**Araştırmanın Gerekçesi**	EMG sinyali verilerine dayanarak kontrol sistemlerinde hızlı ve etkili bir biçimde motor aktivitesini sağlama amacıyla yeni bir derin öğrenme yaklaşımı sunulması ve otomatik el hareketi tahminini gerçekleştirerek literatüre katkıda bulunulması.
**Araştırmanın Yöntemi**	1-EMG sinyallerinin temini<br>2-Ön sinyal işleme ve bölütleme<br>3-Sinyallerin zaman-frekans analizi<br>4-Yapay zeka olarak derin öğrenme modeli kullanılması<br>5-Elde edilen veriler ile yöntemin test edilmesi ve raporlanması
**Kullanılacak biyolojik, psikolojik, teknik vb. tüm yöntemleri açıklayan etik ile ilgili özet**	EMG sinyallerinin temini için Biyomedikal Mühendisliği laboratuvarlarında bulunan ve eğitim amaçlı kullanılan sinyal toplama ve analiz cihazı BIOPAC MP36 ile EMG verilerinin gönüllü katılımcılardan toplanması amaçlanmaktadır. Bu sinyal alma işlemi tamamen zararsız olup yüzey EMG elektrolarının kol deri yüzeyine yapıştırılması ile gerçekleştirilecektir. Veri ölçümü esnasında gönüllülere el hareketlerini içeren bir slayt gösterilip yönlendirilecek ve bu sayede katılımcı ile kayıt sisteminin senkronizasyonu sağlanacaktır. Sinyal toplama işlemi, gönüllü üzerinde herhangi bir analiz işlemi gerektirmeyen insan vücudunda var olan verilerin ölçülmesi prensibine dayanmaktadır. Herhangi bir doktor gözetimine ihtiyaç olmayan risksiz bir yöntemdir. Toplanan verilerin

T.C. İZMİR KÂTİP ÇELEBİ ÜNİVERSİTESİ
FEN VE MÜHENDİSLİK BİLİMLERİ
BİLİMSEL ARAŞTIRMA ve YAYIN ETİK KURULU

	istatiksel analiz yöntemi ile işlenip yapay zekayı eğitmek için kullanması planlanmaktadır. Ayrıca ölçüm sonrasında sonra gönüllü katılımcıya 8 soruluk bir anket formu uygulanacaktır. Daha sonra anket formundan ve EMG sinyal toplama işleminden elde edilen bilgiler katılımcıların kişisel verileri gizli tutulacak şekilde bilim dünyasına katkıda bulunmak amacıyla paylaşılacaktır. Bu çalışmada toplanan EMG verilerinin çalışmaya benzer nitelikte yapılabilecek potansiyel çalışmalara kaynak olması bakımından veri seti olarak paylaşılması planlanmaktadır. Bu çalışmada katılımcılardan EMG ölçümlerinin alınması, katılımcılara kısa anket uygulaması yapılması, toplanan anket ve EMG verileri üzerinde sinyal işleme ve istatiksel analiz yöntemlerinin uygulanması, elde edilen sonuçların bildiri ve makale şeklinde raporlanması ve oluşturulacak el hareketlerinden elde edilen EMG sinyalleri veri setinin bilim dünyası ile katılımcı bilgileri gizli kalacak şekilde paylaşılması için etik kurul iznine ihtiyaç duyulmaktadır.

İzmir Katip Çelebi Üniversitesi Mühendislik ve Mimarlık Fakültesi Biyomedikal Mühendisliği Klinik Mühendisliği Anabilim Dalı öğretim elemanlarından Mehmet Akif Özdemir tarafından yürütülmesi planlanan "EMG Sinyalleri ve Yapay Zeka Yöntemleri Kullanılarak El Hareketi Tahmini" adlı çalışma değerlendirilmiştir.

Tarafımıza sunulan çalışma önerisi kurulumuz üyeleri tarafından incelenmiş ve yapılan değerlendirmeler sonucunda aşağıdaki karar verilmiştir.

Araştırma etik açısından uygun bulunmuştur.	x
Araştırma etik açısından geliştirilmesi gerekmektedir.	☐
Araştırma etik açısından uygun bulunmamıştır.	☐

İmzalar:

Prof. Dr. Adnan KAYA
Başkan

Prof. Dr. Şerafettin DEMİÇ
Üye

Prof. Dr. Mehmet ÇEVİK
Üye

Prof. Dr. Semih ENGİN
Üye

Prof. Dr. Nilgül ÇETİN
Üye

Prof. Dr. Buket OKUTAN BABA
Üye

Prof. Dr. Ramazan SEREZLİ
Üye
