# Supplementary material for: Dataset for multi-channel surface electromyography (sEMG) signals of hand gestures
Source: Data Brief. 2022 Feb 4;41:107921. doi: 10.1016/j.dib.2022.107921 (PMC8844426; doi:10.1016/j.dib.2022.107921)
Supplement: Supplementary file 2 [file mmc2.pptx]

## Slide 1
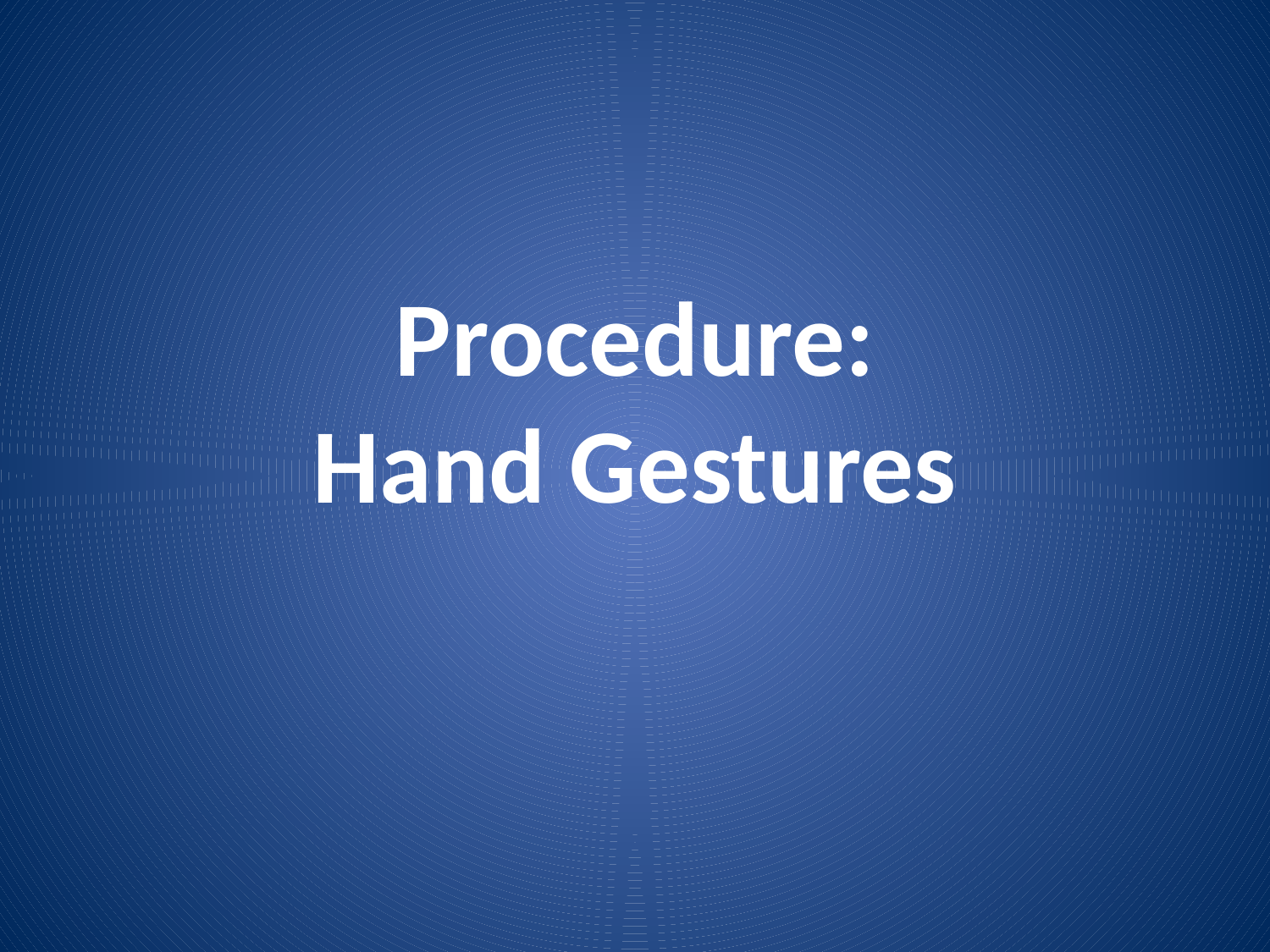

# Procedure: Hand Gestures

## Slide 2
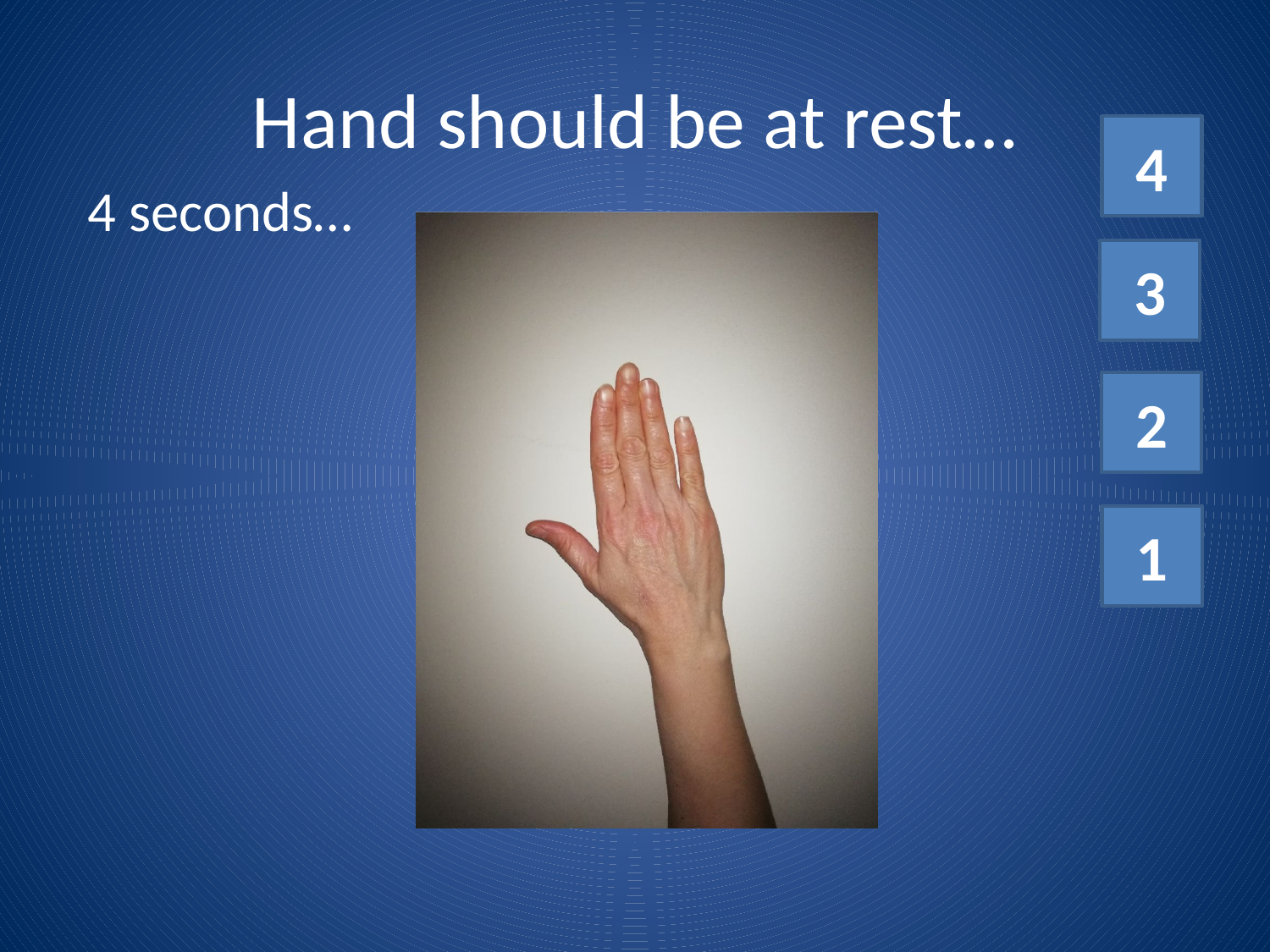

# Hand should be at rest…
4
4 seconds…
3
2
1

## Slide 3
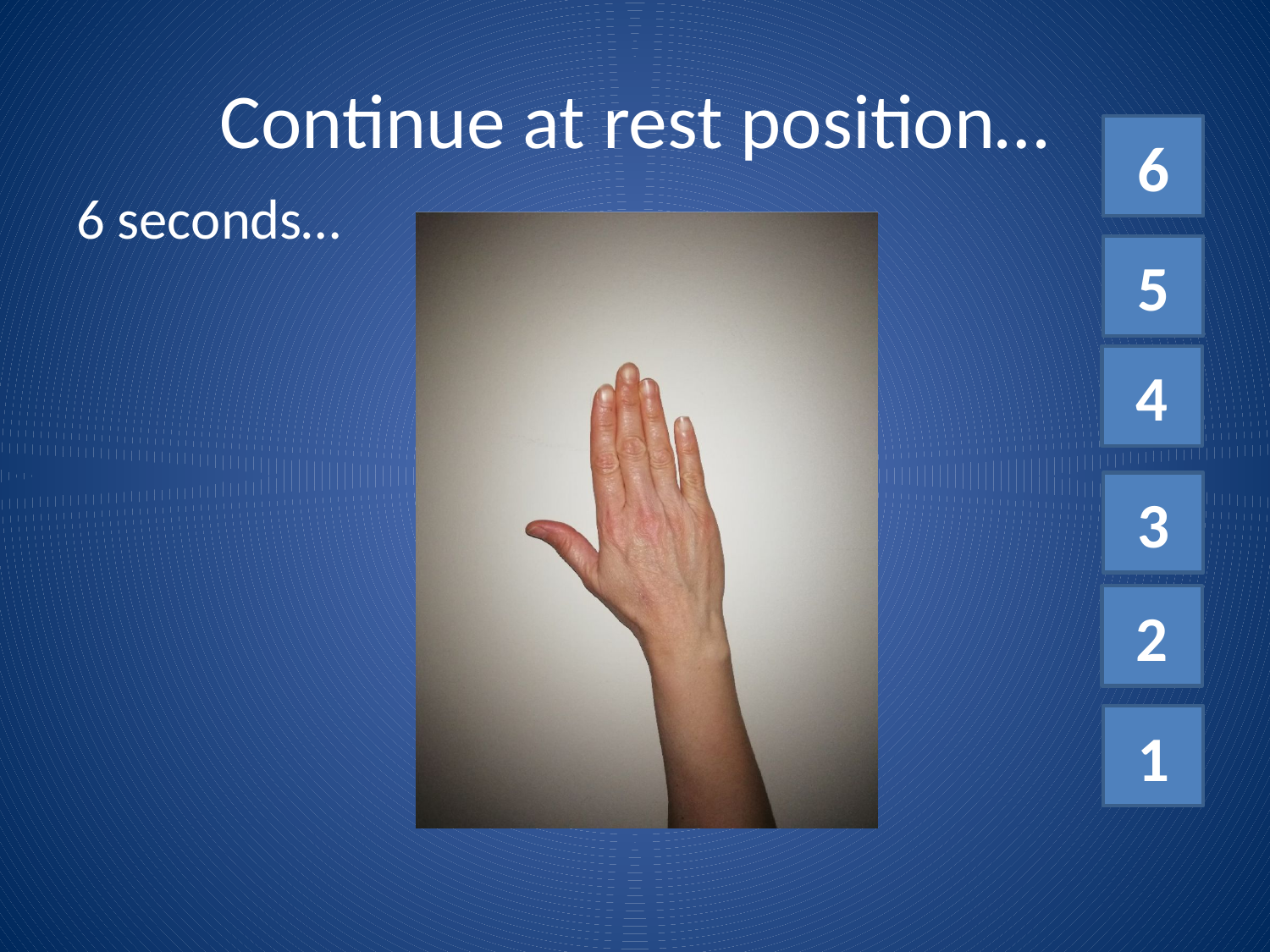

# Continue at rest position…
6
6 seconds…
5
4
3
2
1

## Slide 4
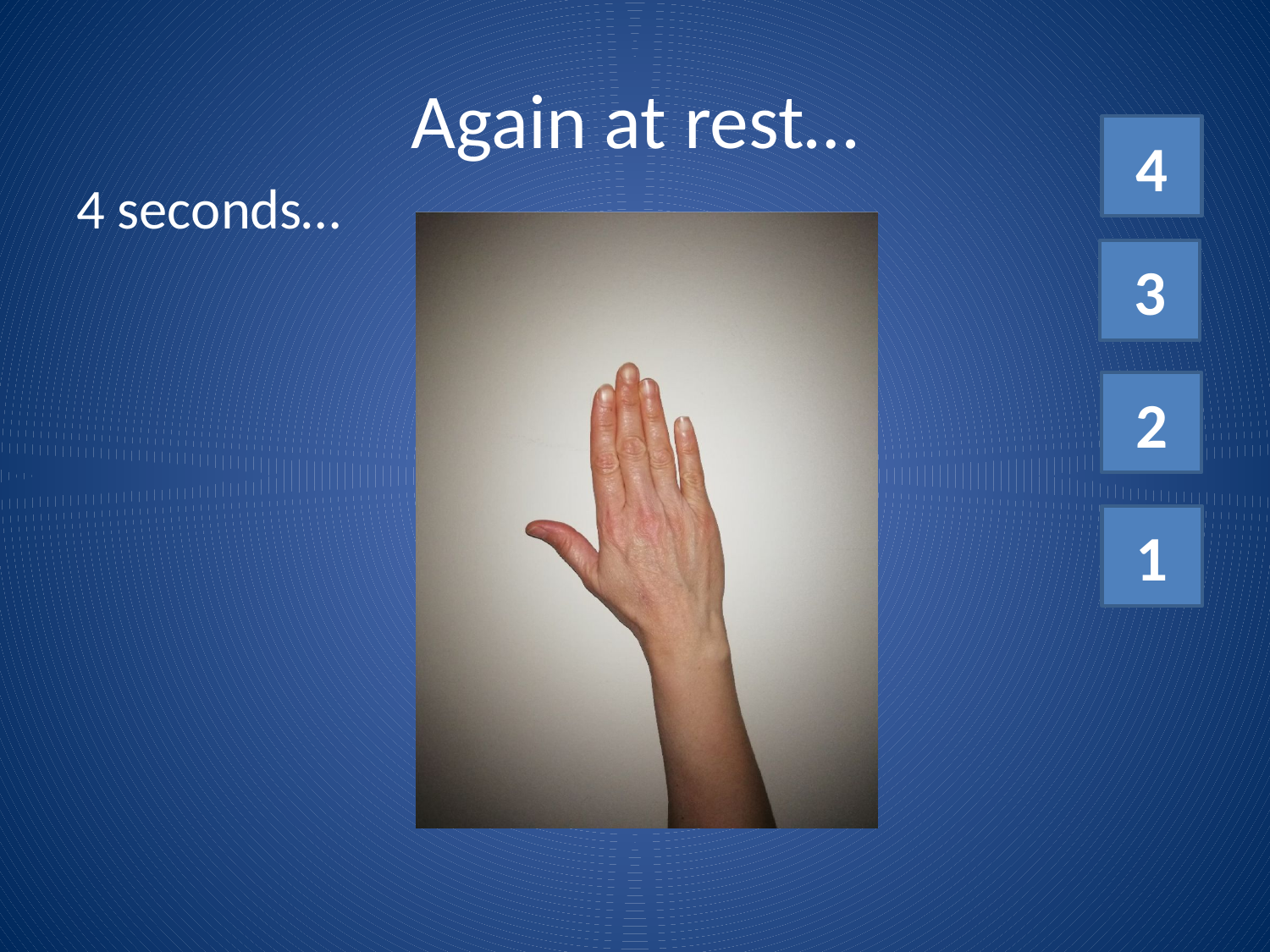

# Again at rest…
4
4 seconds…
3
2
1

## Slide 5
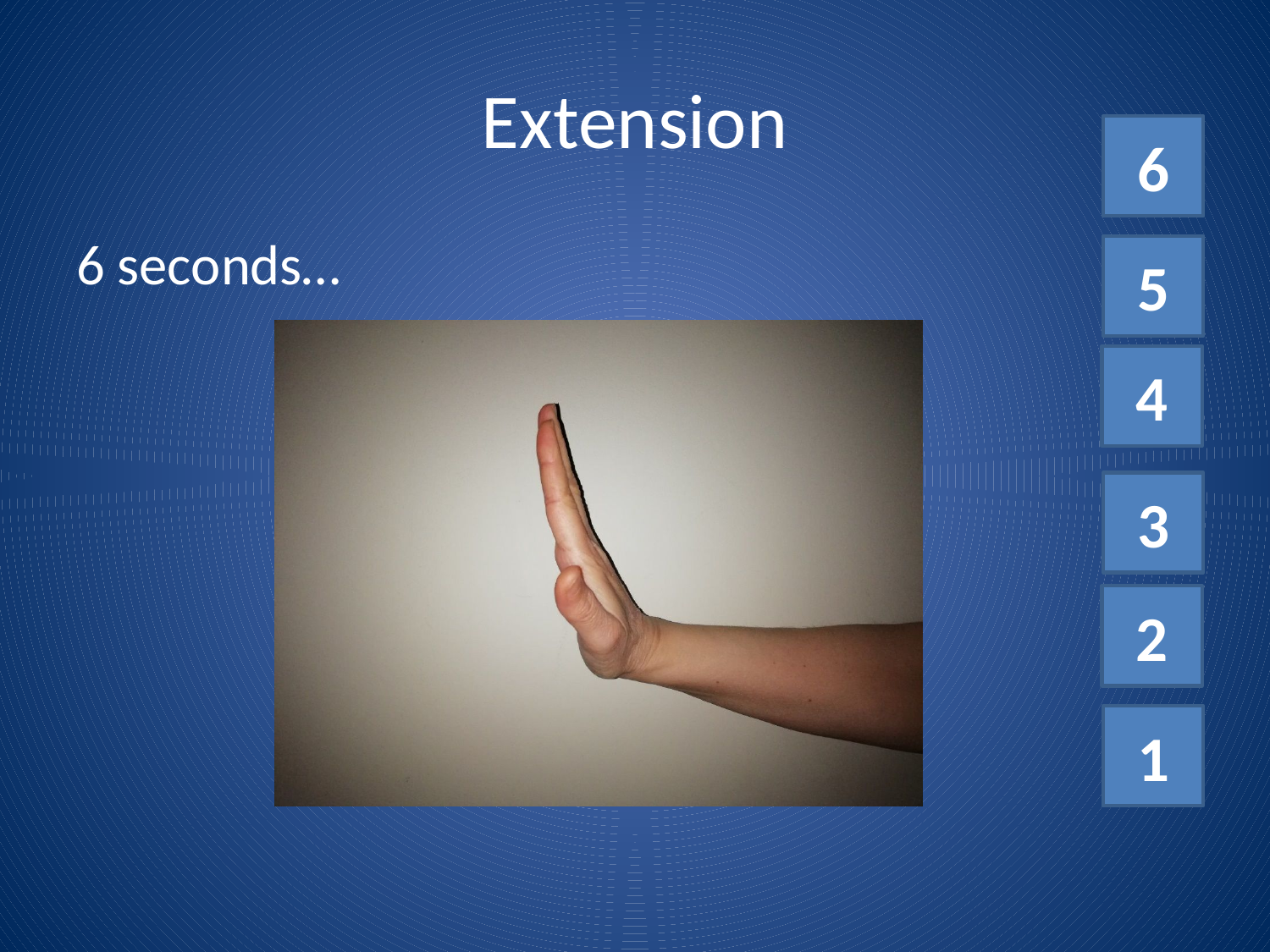

# Extension
6
6 seconds…
5
4
3
2
1

## Slide 6
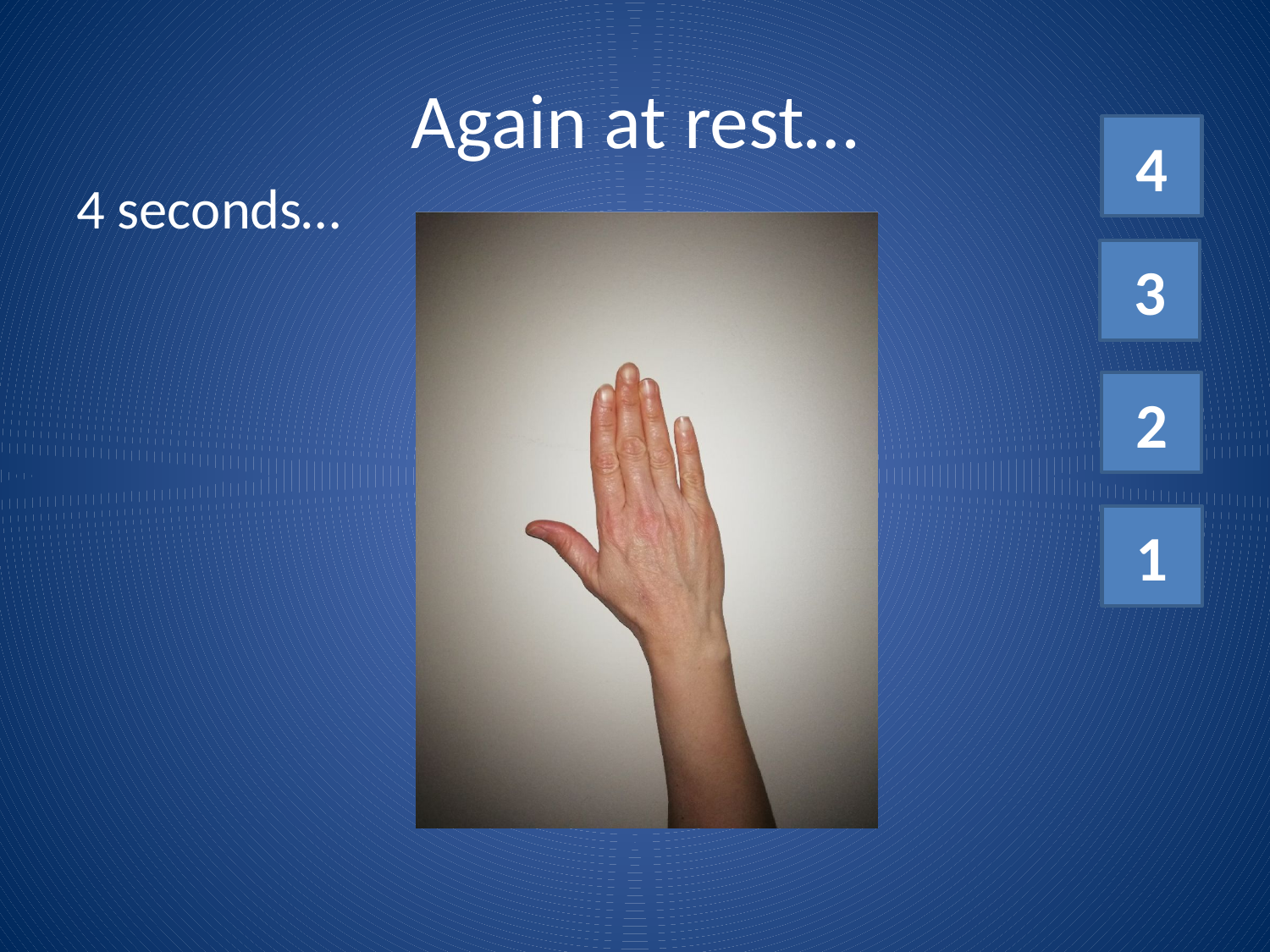

# Again at rest…
4
4 seconds…
3
2
1

## Slide 7
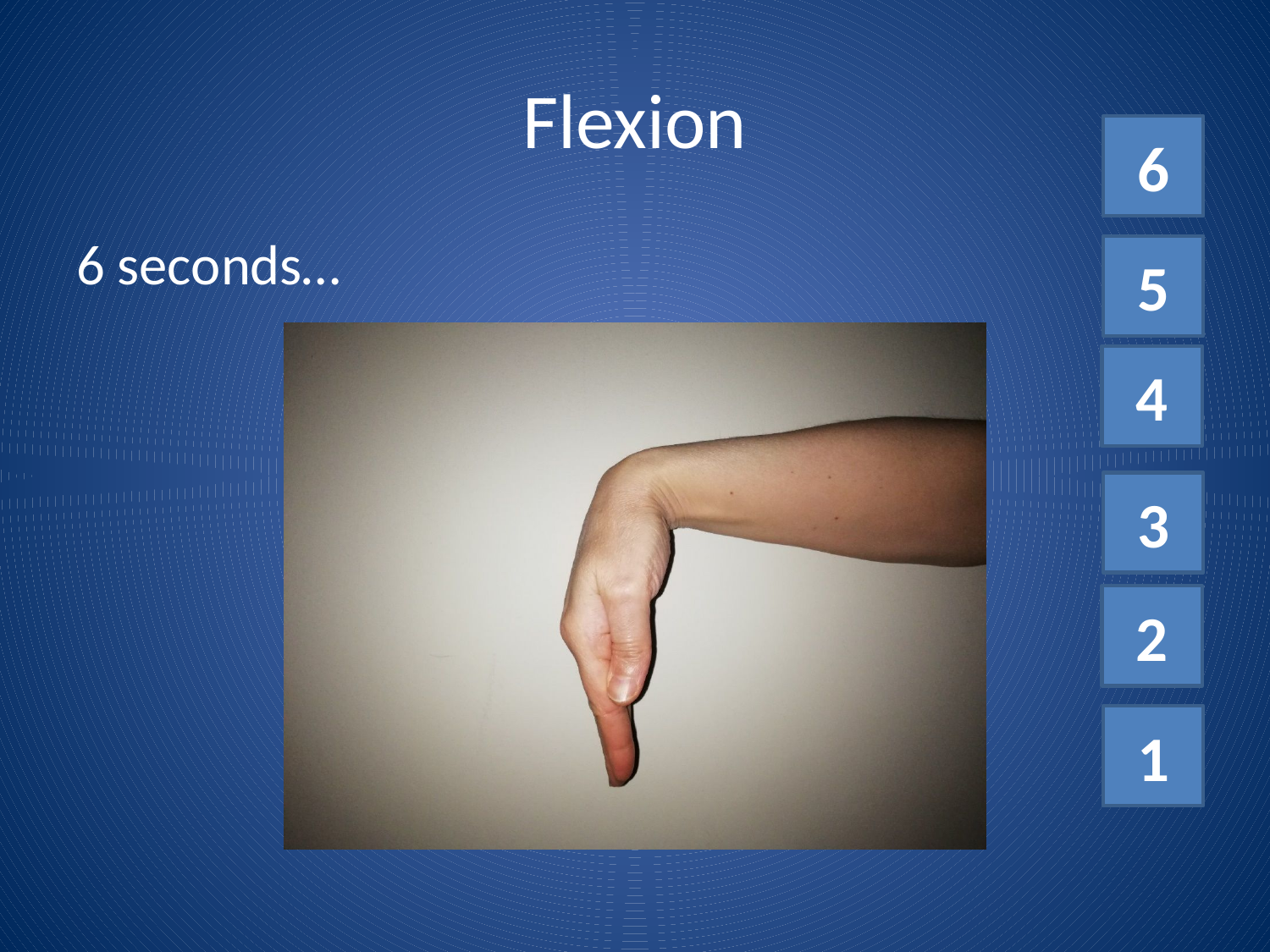

# Flexion
6
6 seconds…
5
4
3
2
1

## Slide 8
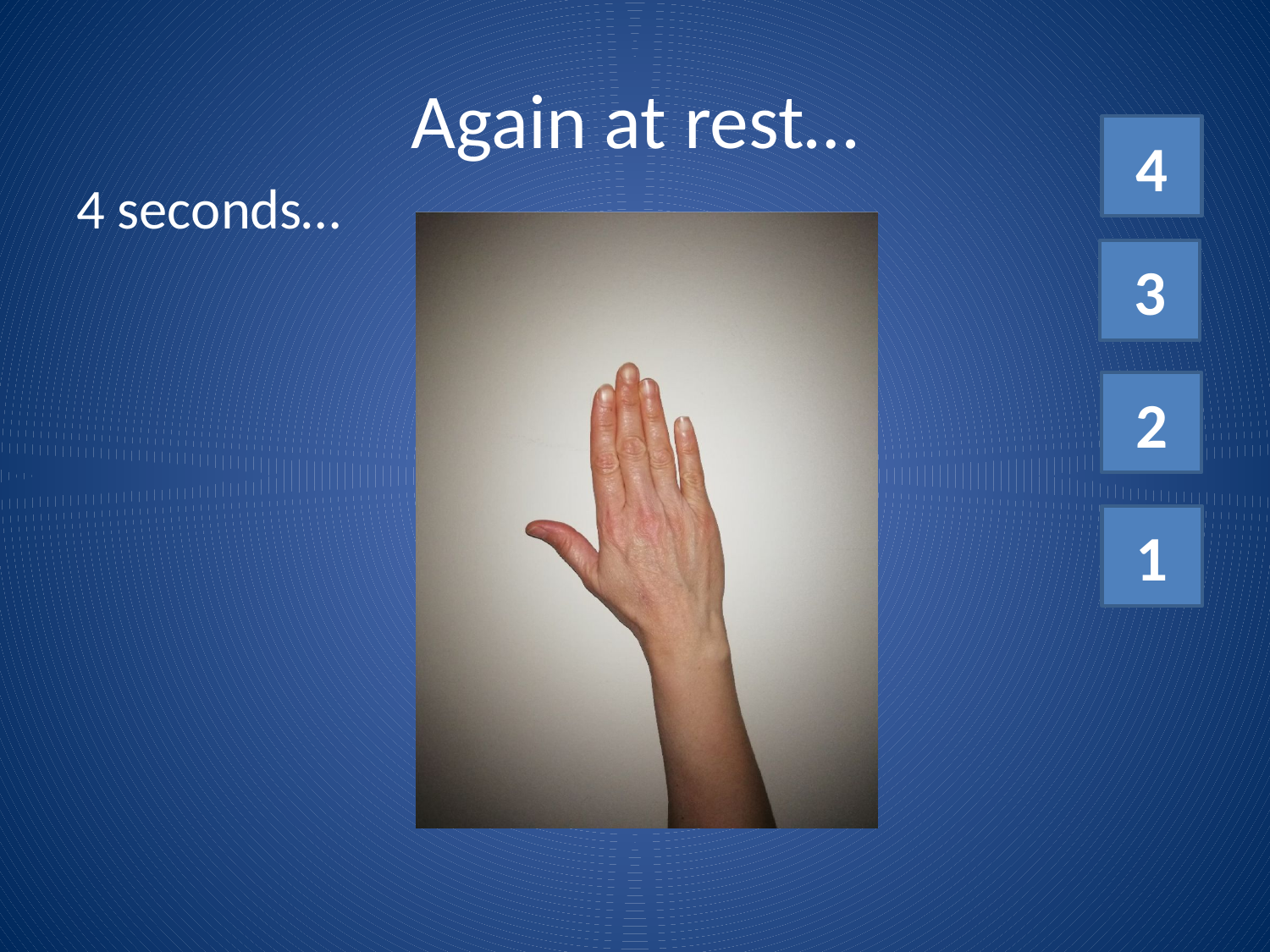

# Again at rest…
4
4 seconds…
3
2
1

## Slide 9
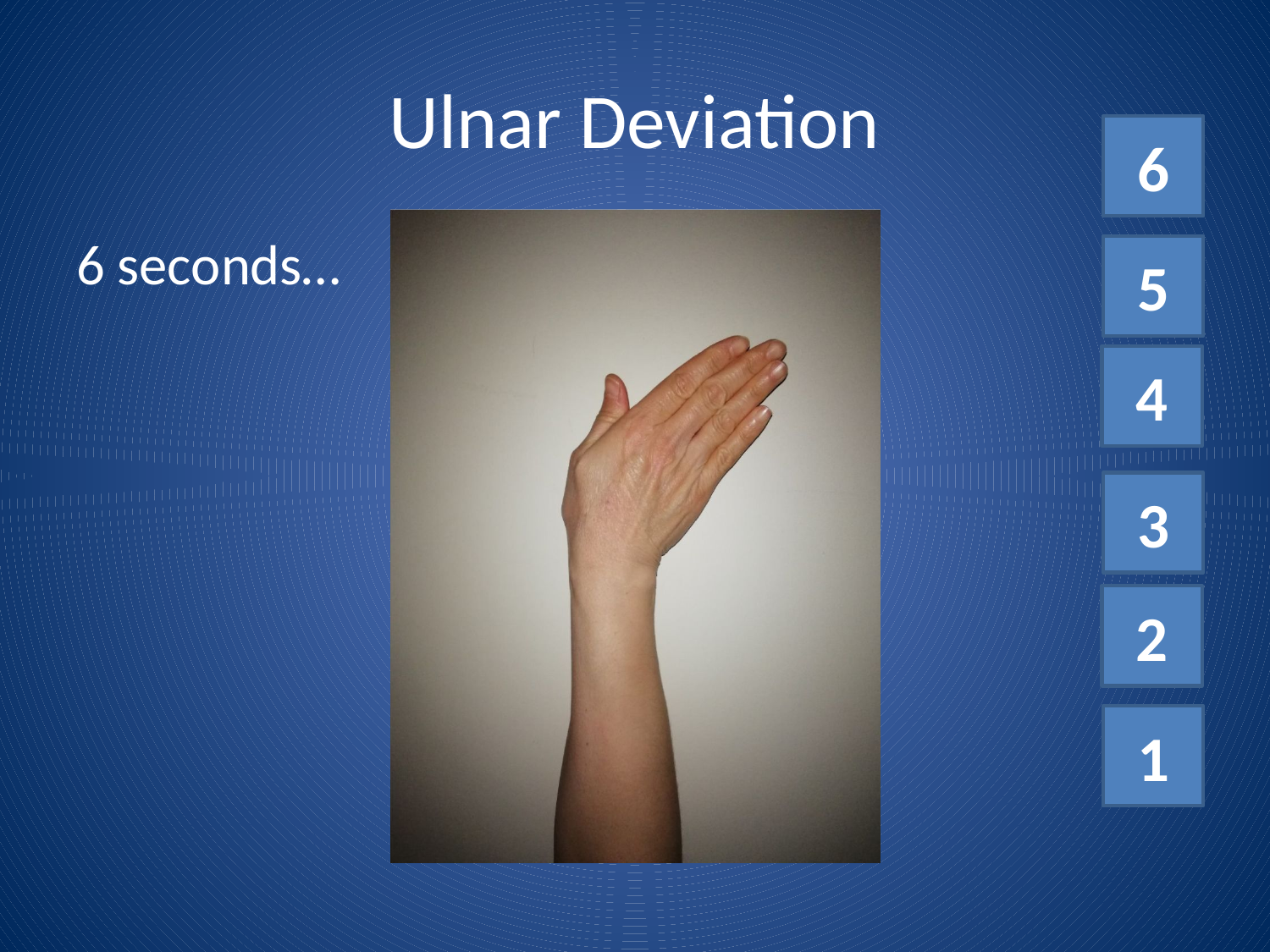

# Ulnar Deviation
6
6 seconds…
5
4
3
2
1

## Slide 10
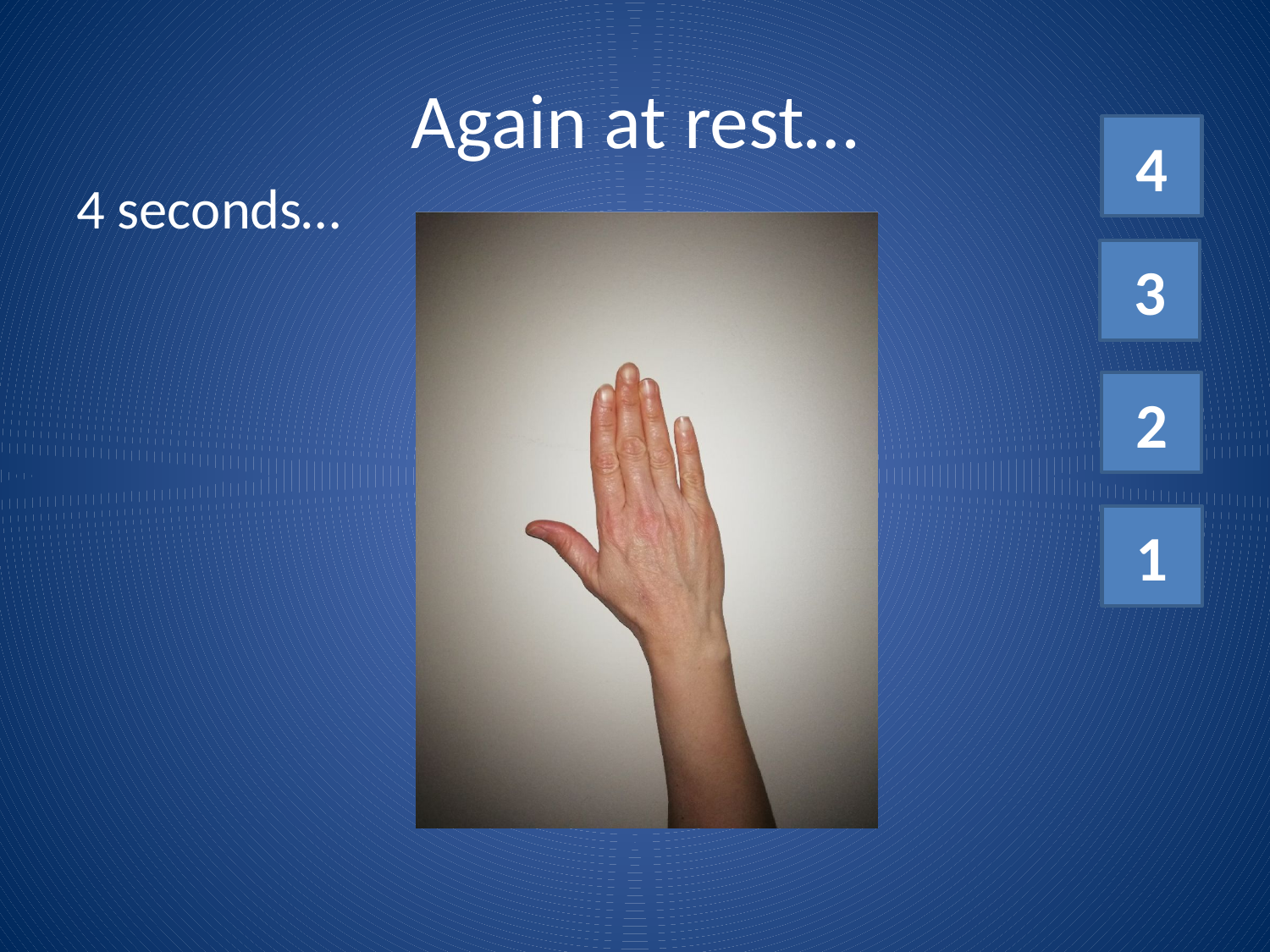

# Again at rest…
4
4 seconds…
3
2
1

## Slide 11
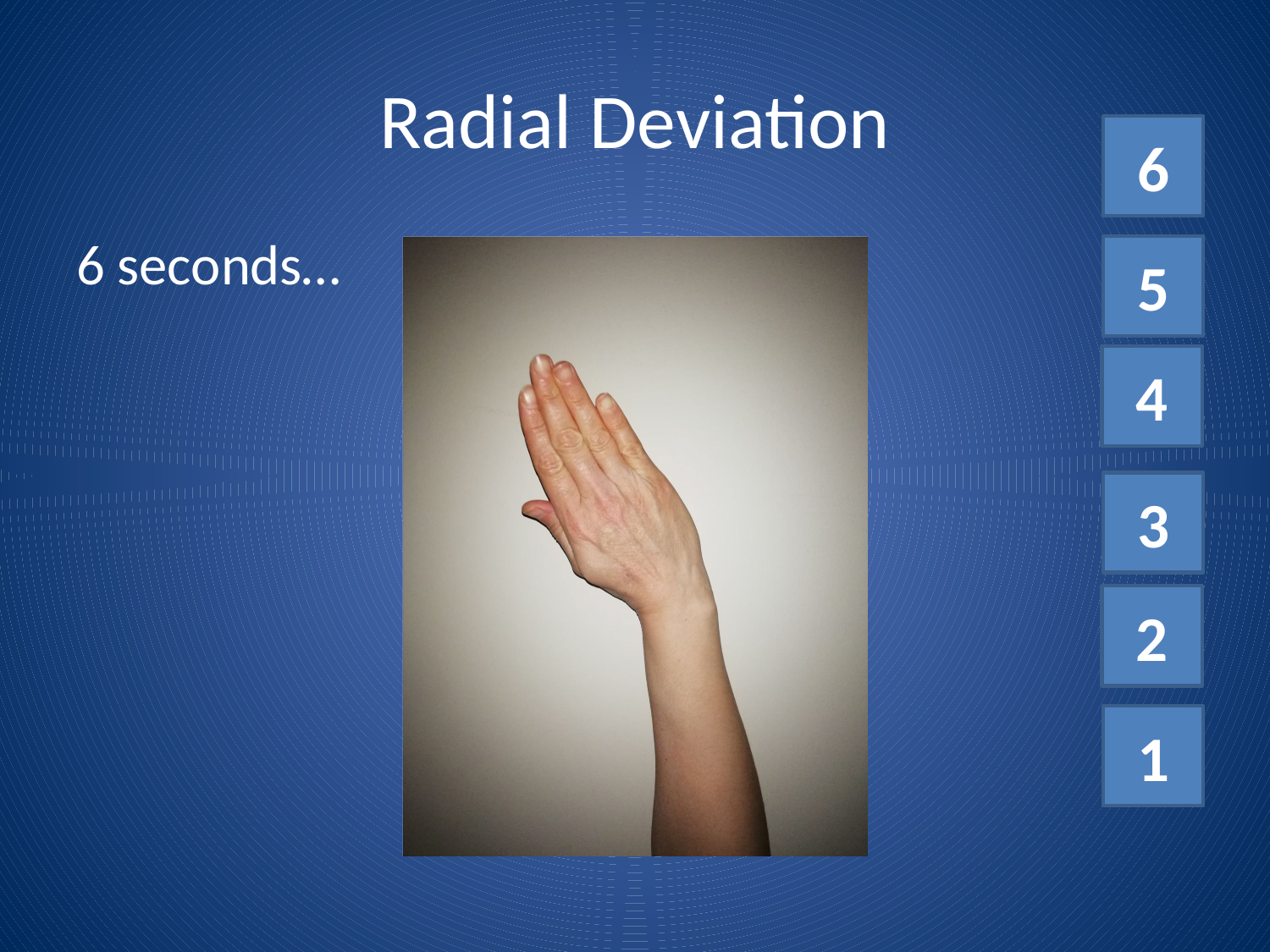

# Radial Deviation
6
6 seconds…
5
4
3
2
1

## Slide 12
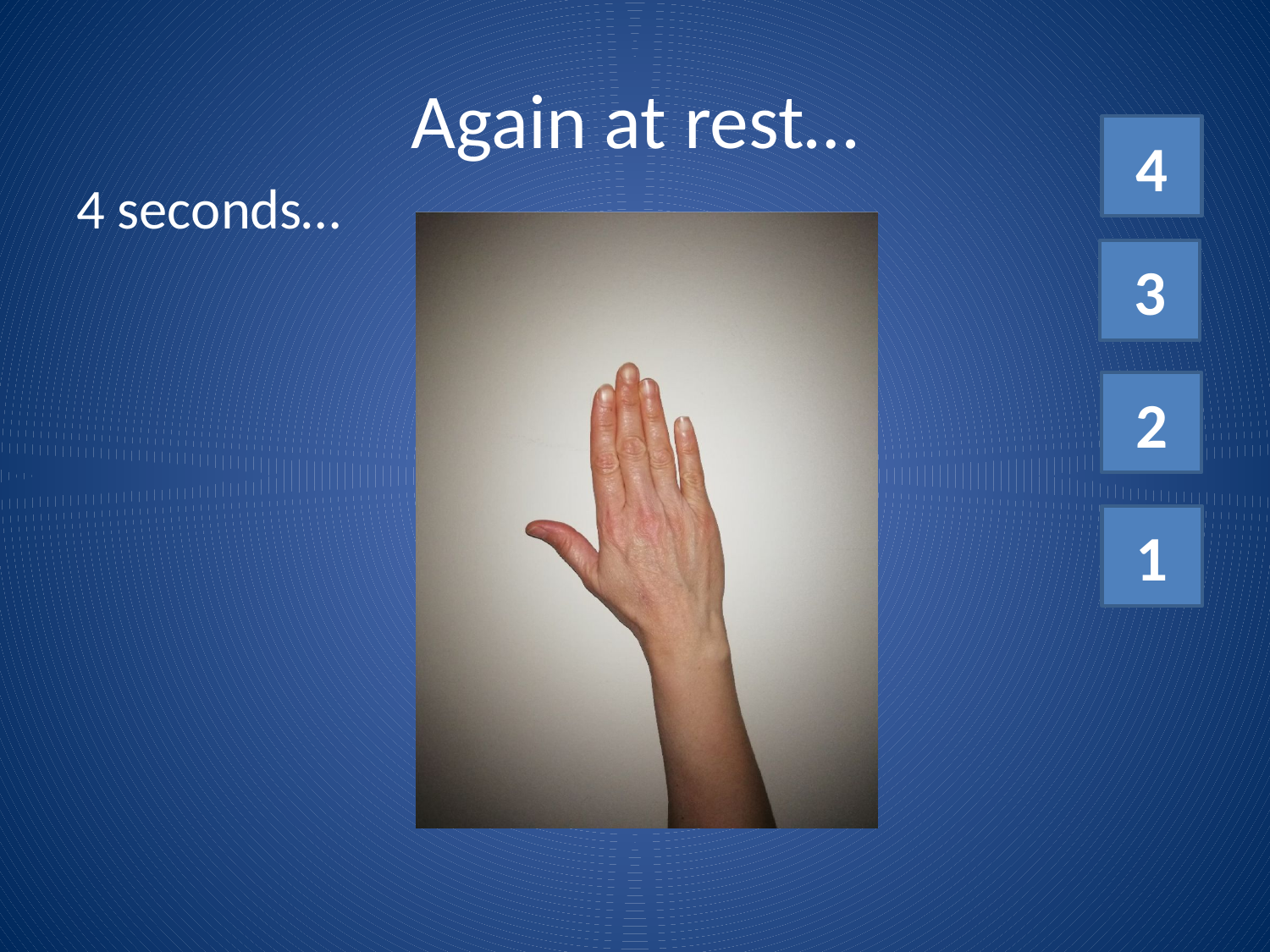

# Again at rest…
4
4 seconds…
3
2
1

## Slide 13
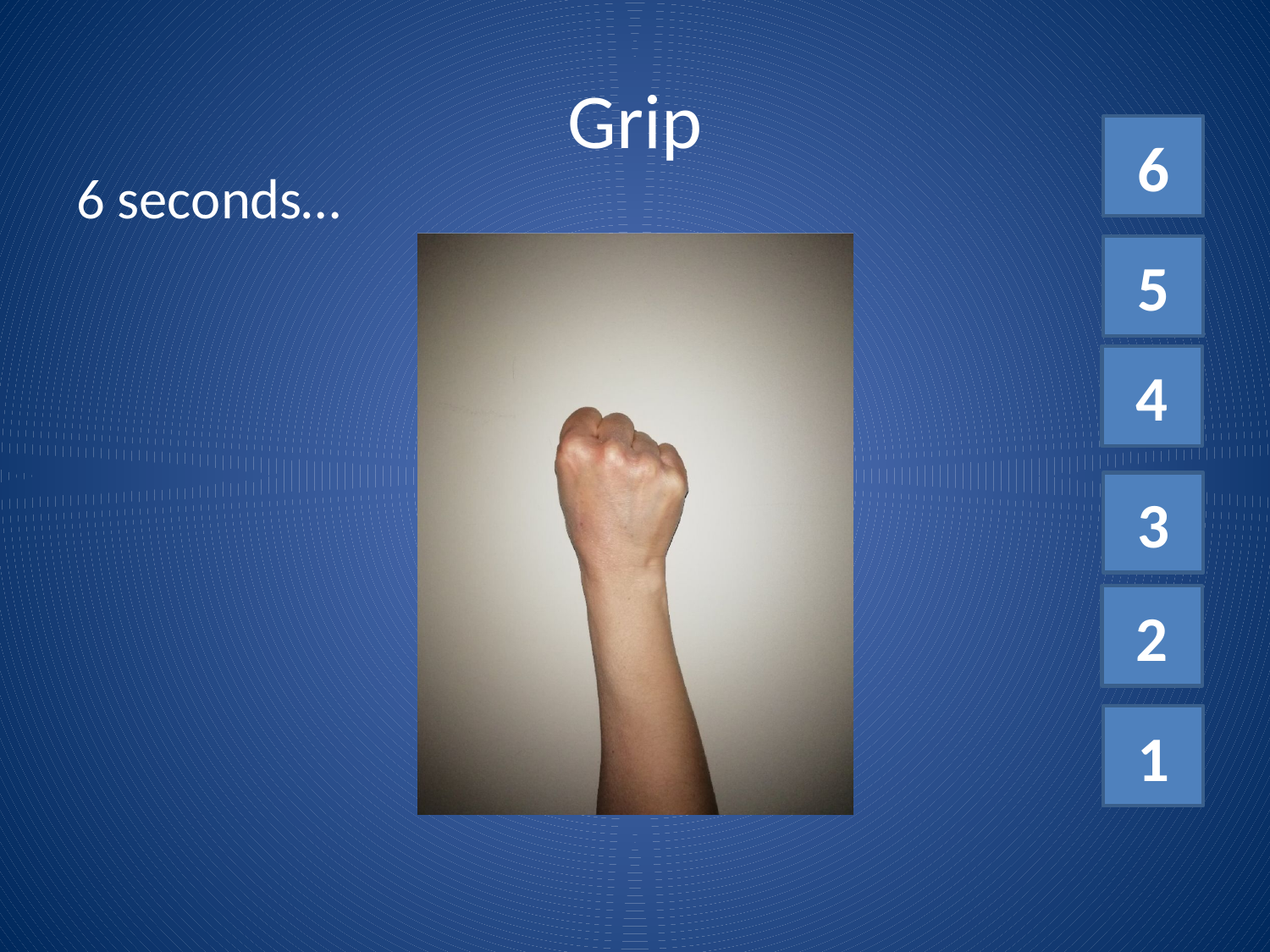

# Grip
6
6 seconds…
5
4
3
2
1

## Slide 14
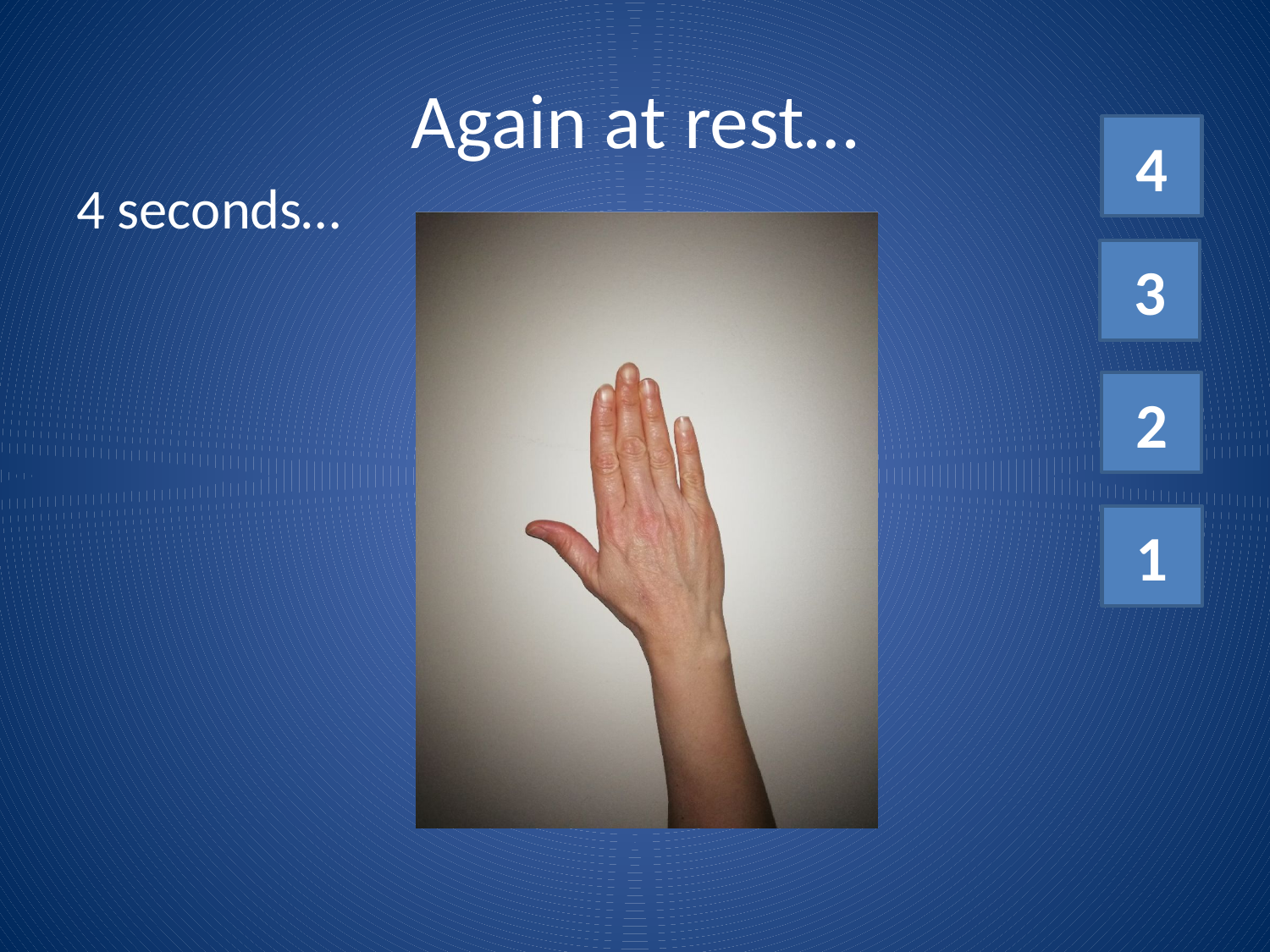

# Again at rest…
4
4 seconds…
3
2
1

## Slide 15
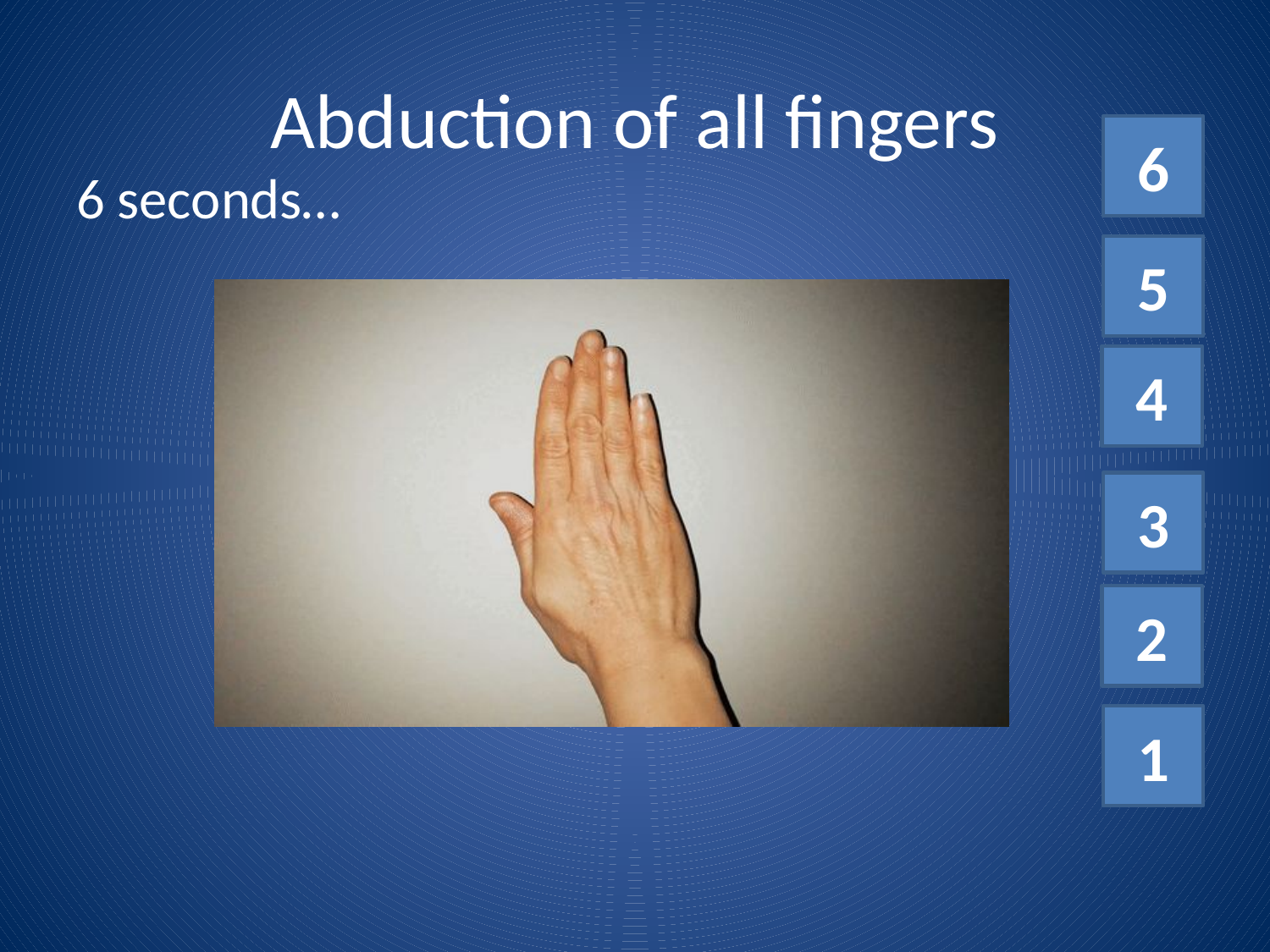

# Abduction of all fingers
6
6 seconds…
5
4
3
2
1

## Slide 16
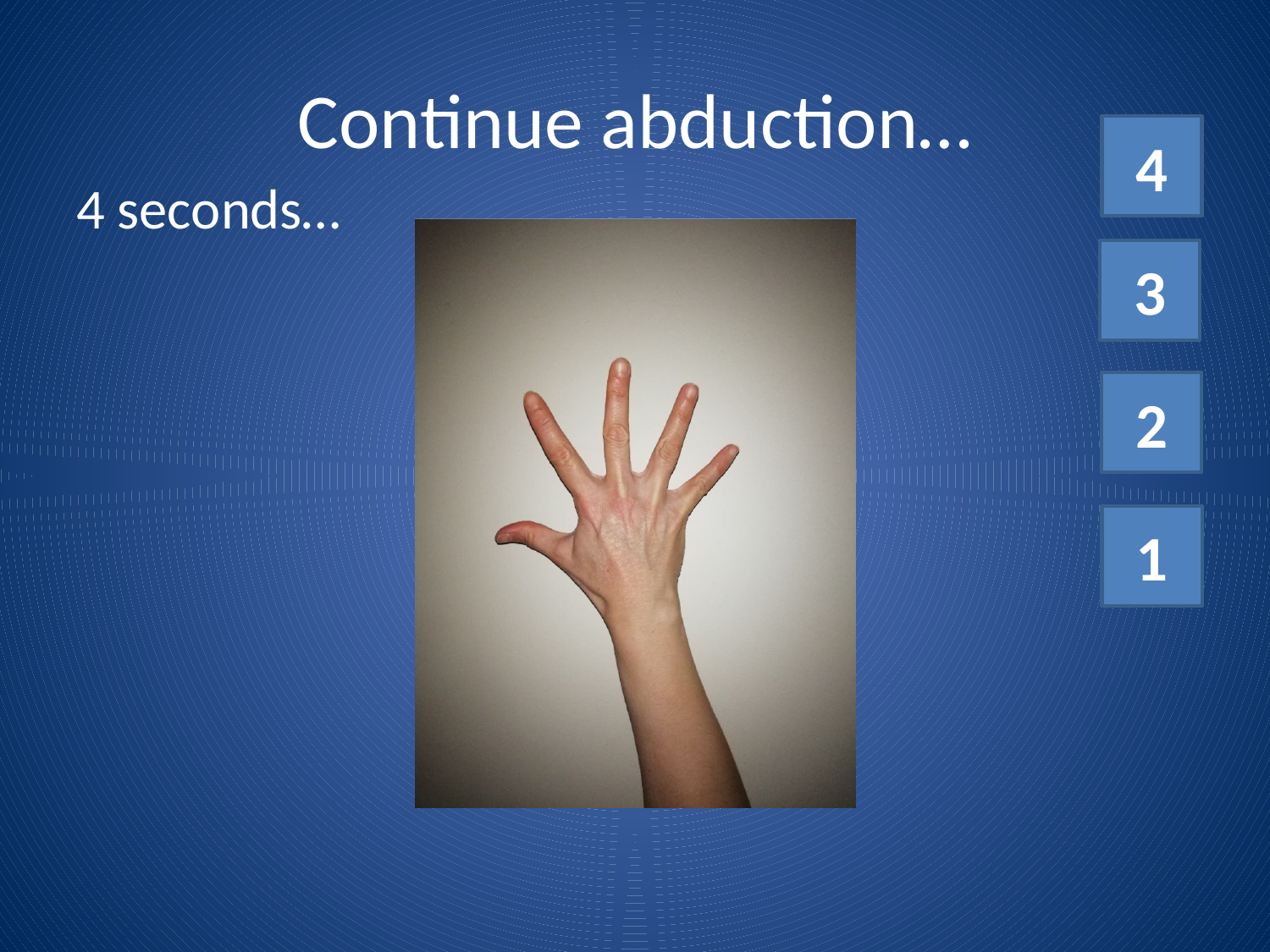

# Continue abduction…
4
4 seconds…
3
2
1

## Slide 17
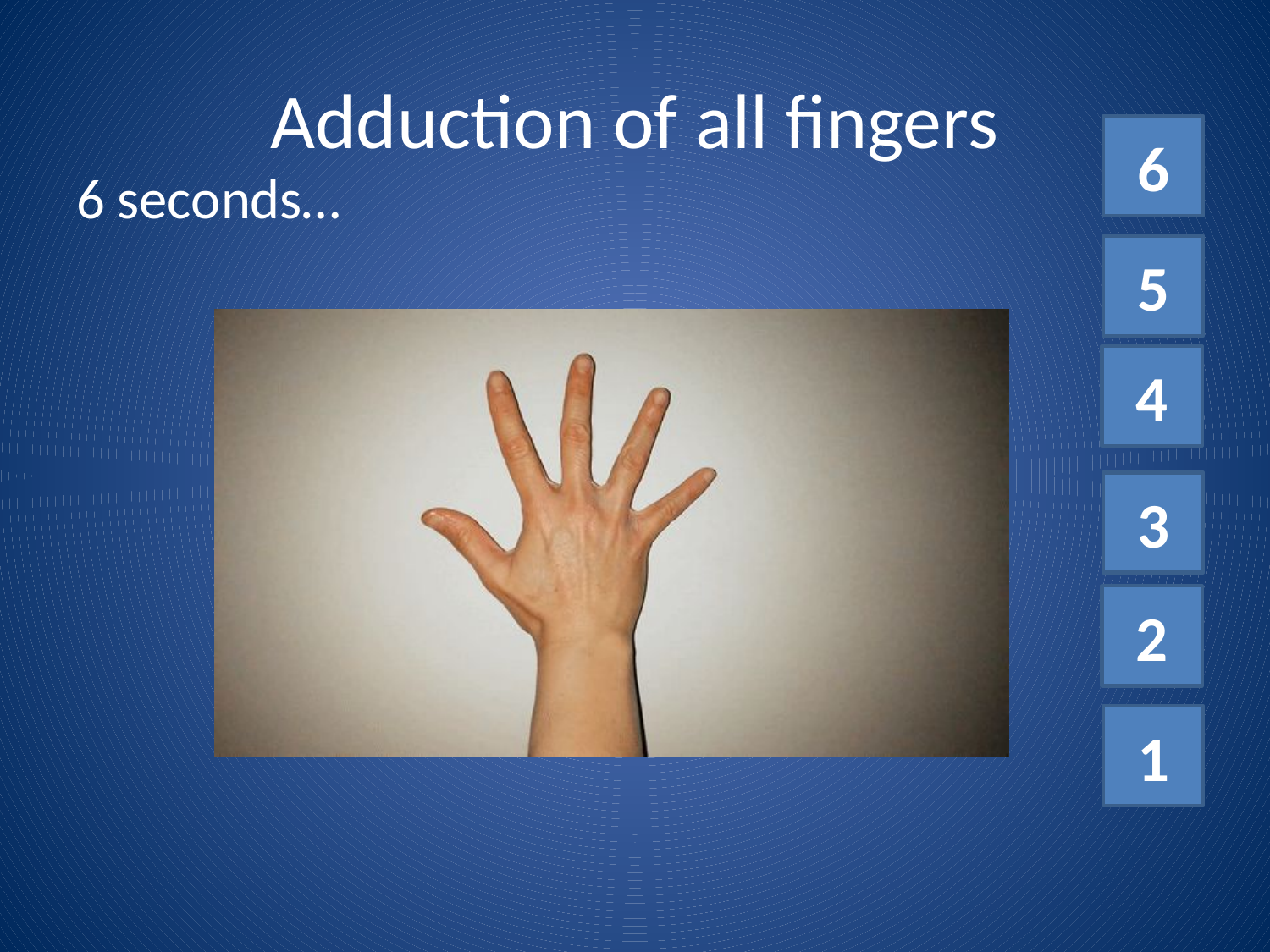

# Adduction of all fingers
6
6 seconds…
5
4
3
2
1

## Slide 18
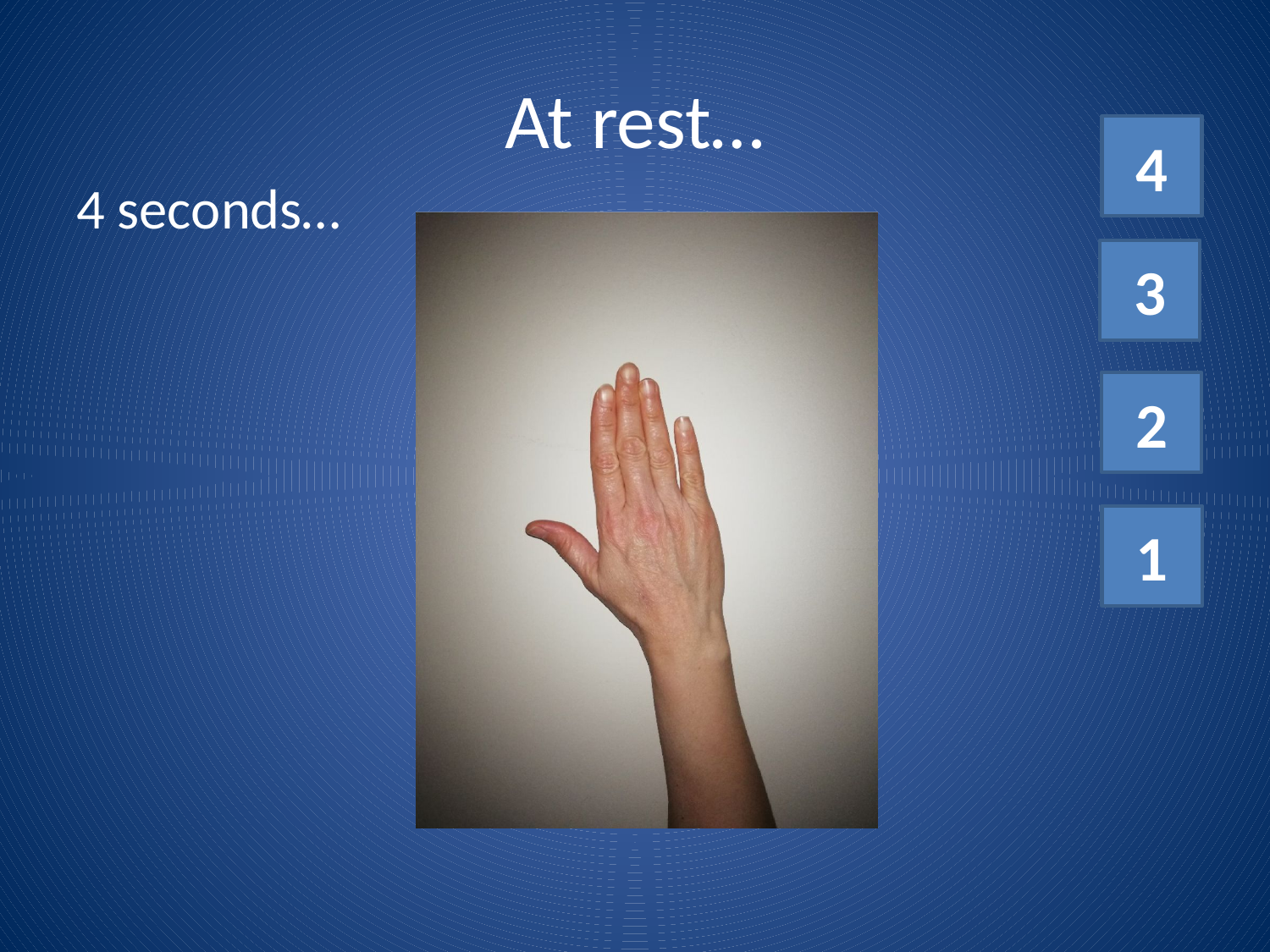

# At rest…
4
4 seconds…
3
2
1

## Slide 19
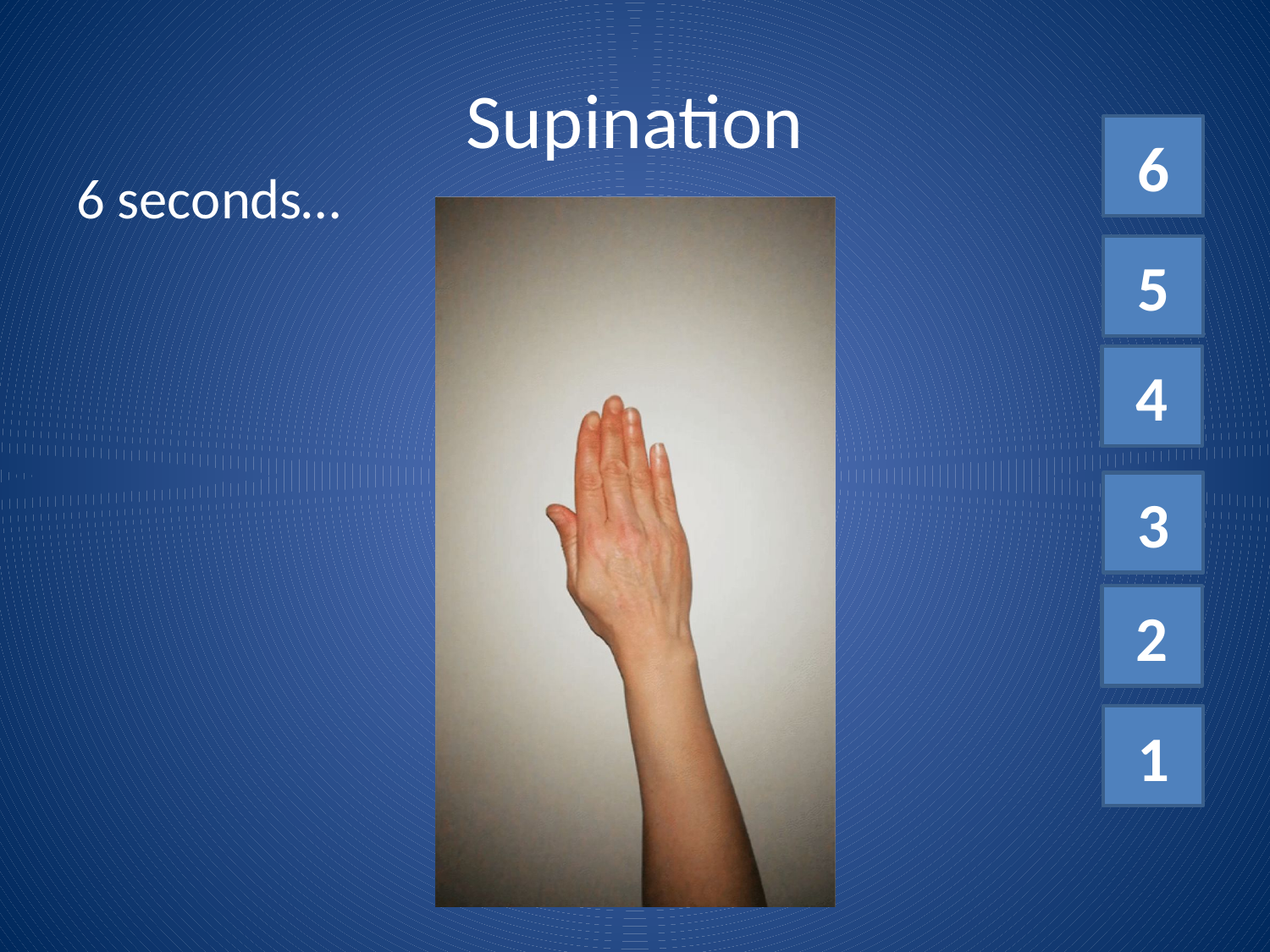

# Supination
6
6 seconds…
5
4
3
2
1

## Slide 20
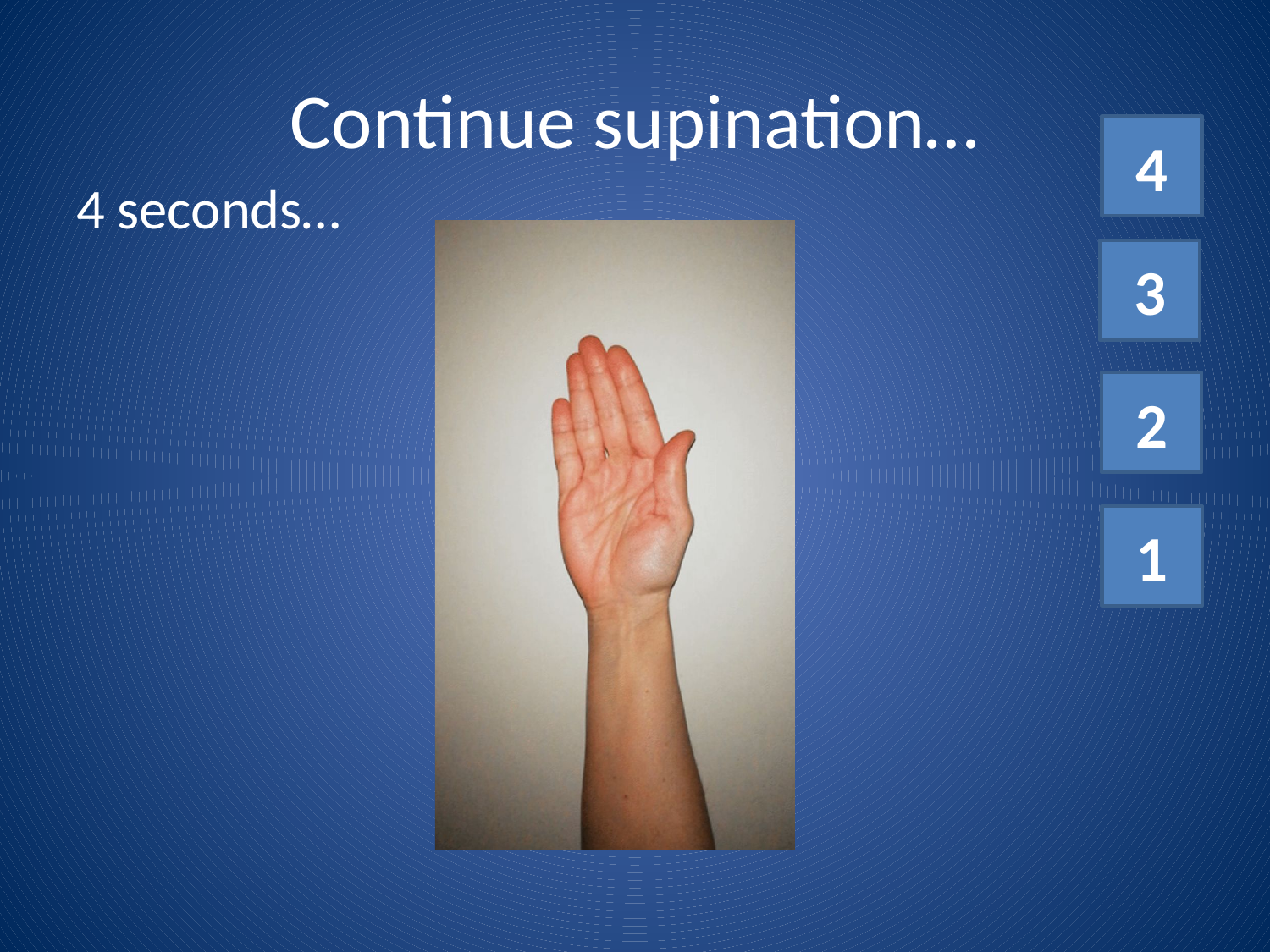

# Continue supination…
4
4 seconds…
3
2
1

## Slide 21
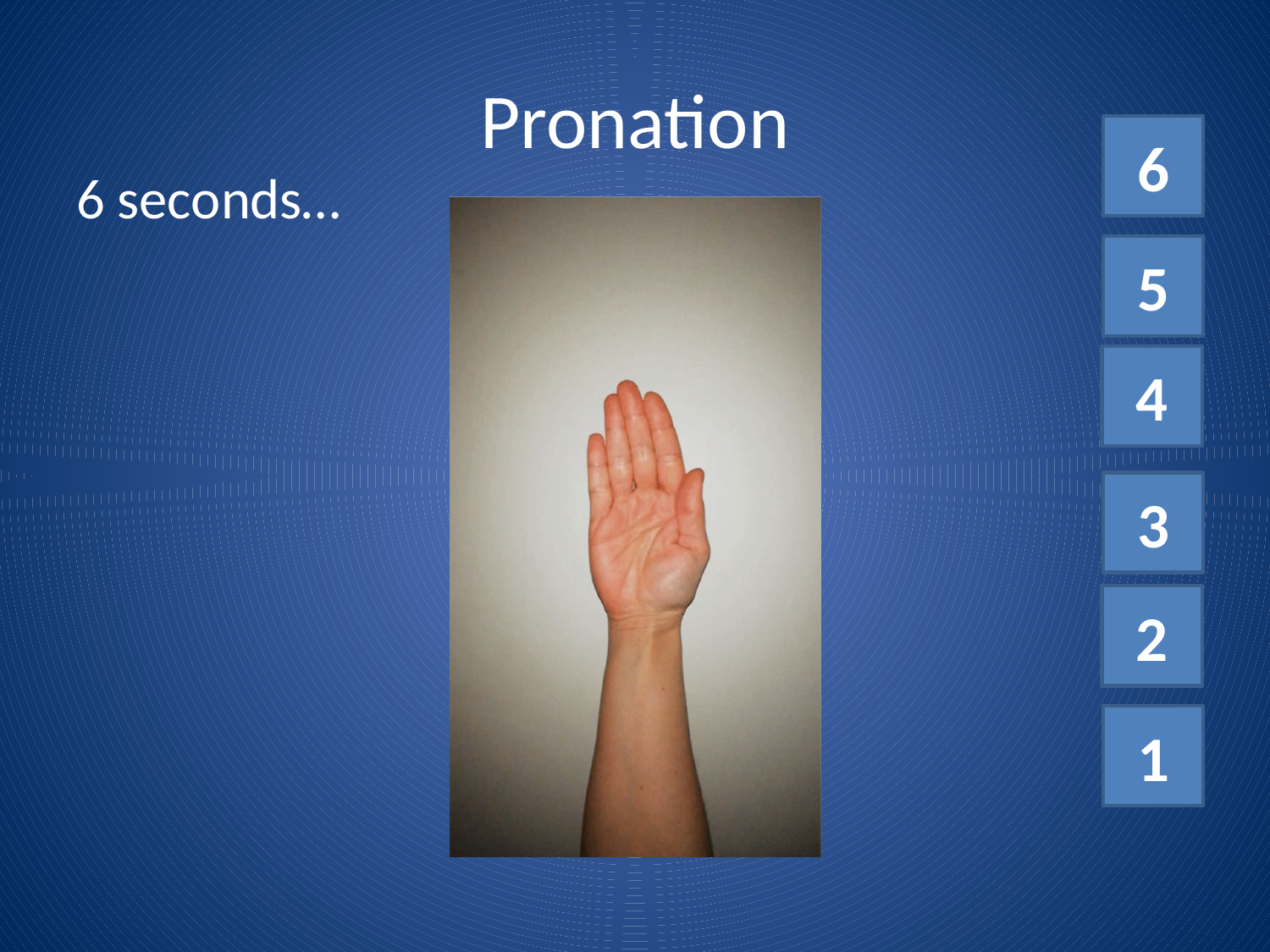

# Pronation
6
6 seconds…
5
4
3
2
1

## Slide 22
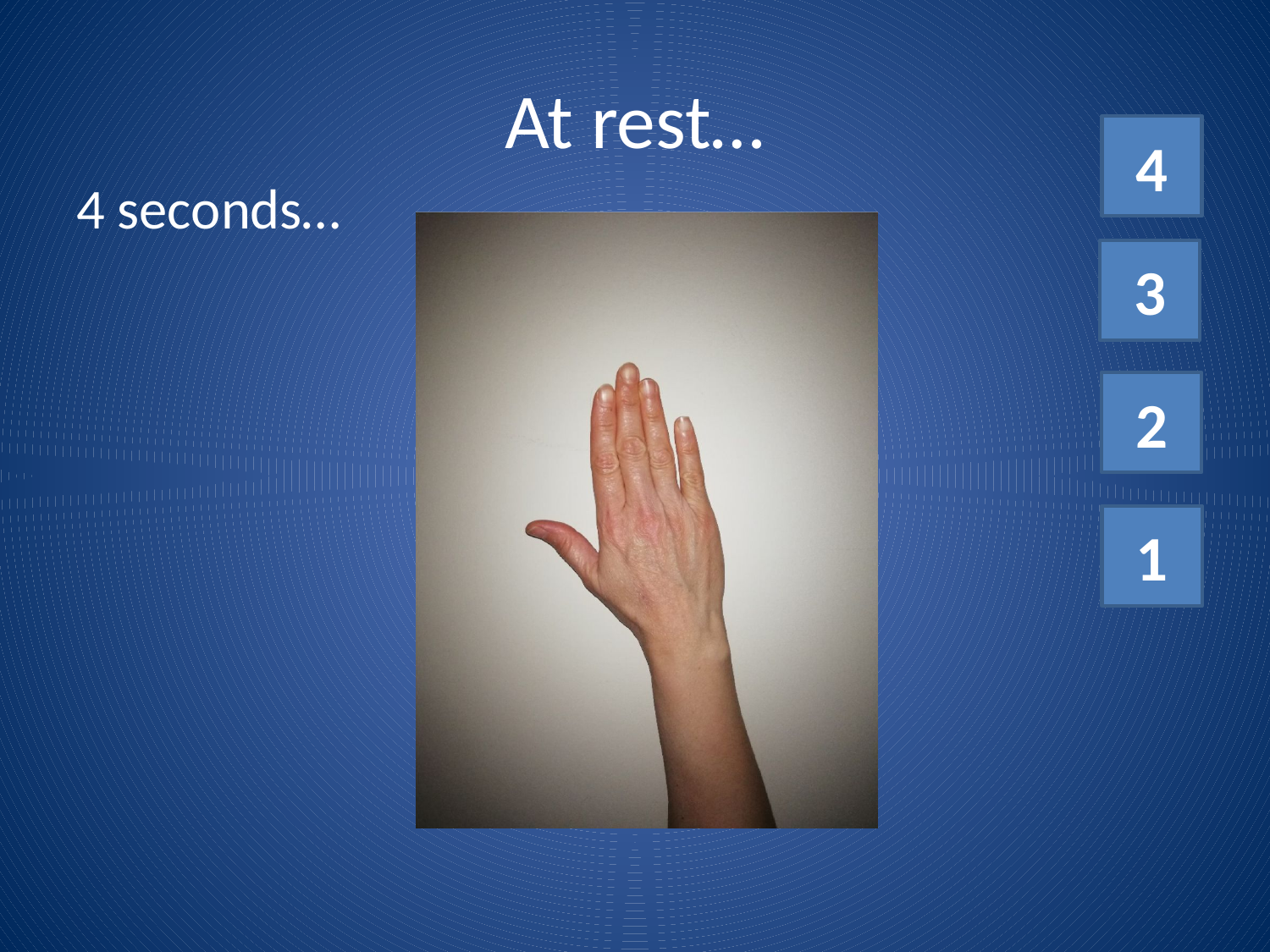

# At rest…
4
4 seconds…
3
2
1

## Slide 23
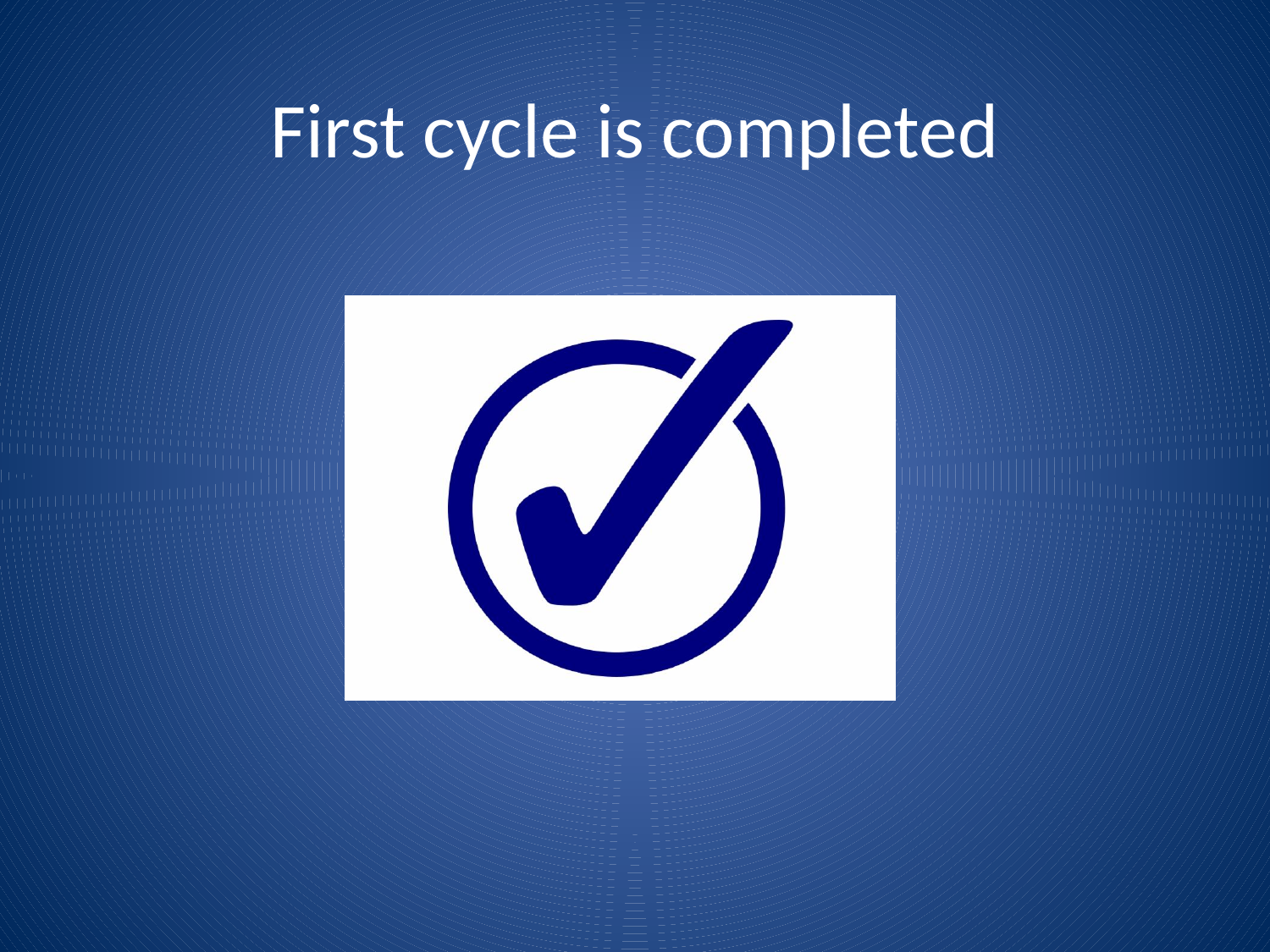

# First cycle is completed

## Slide 24
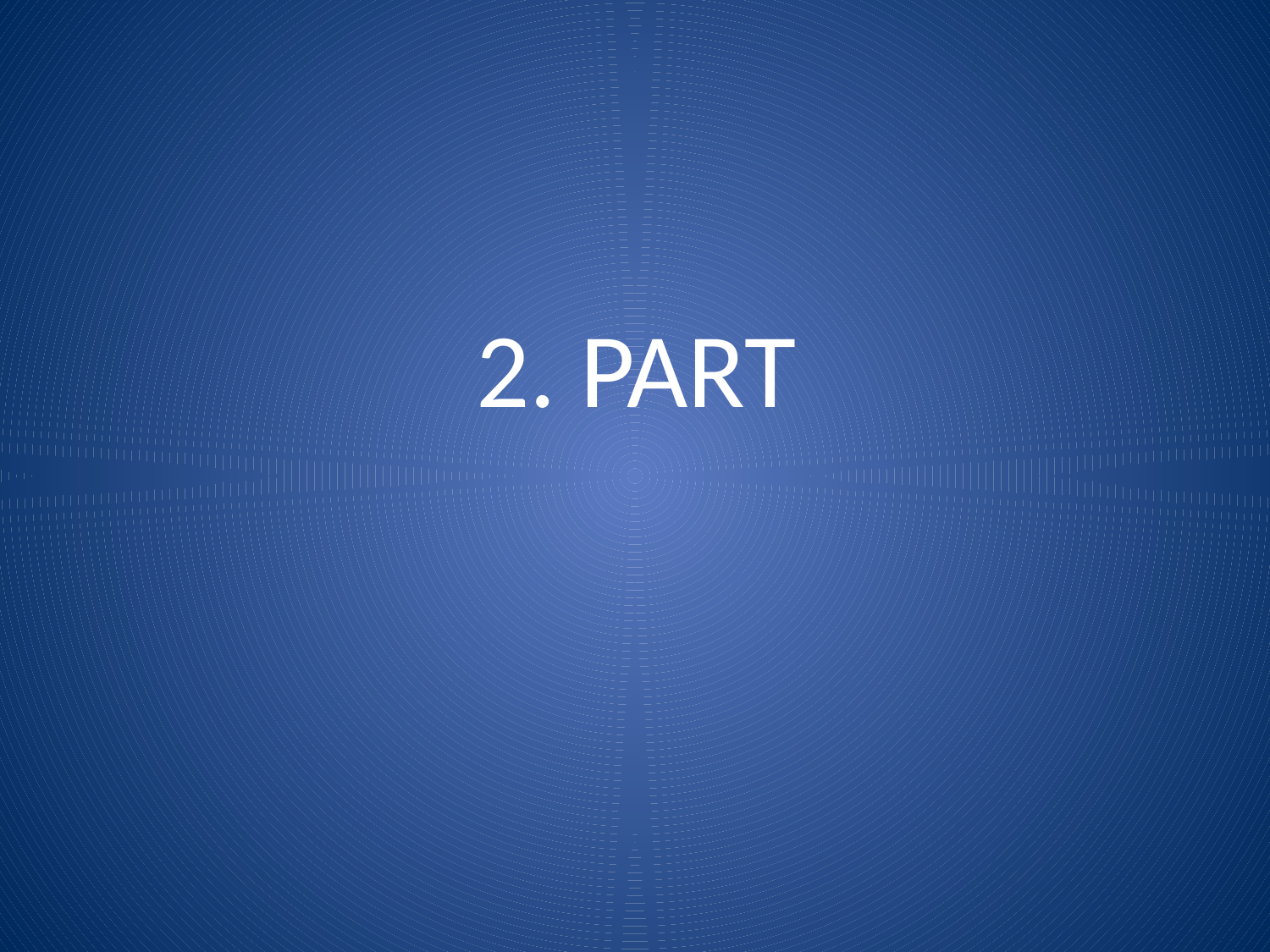

# 2. PART

## Slide 25
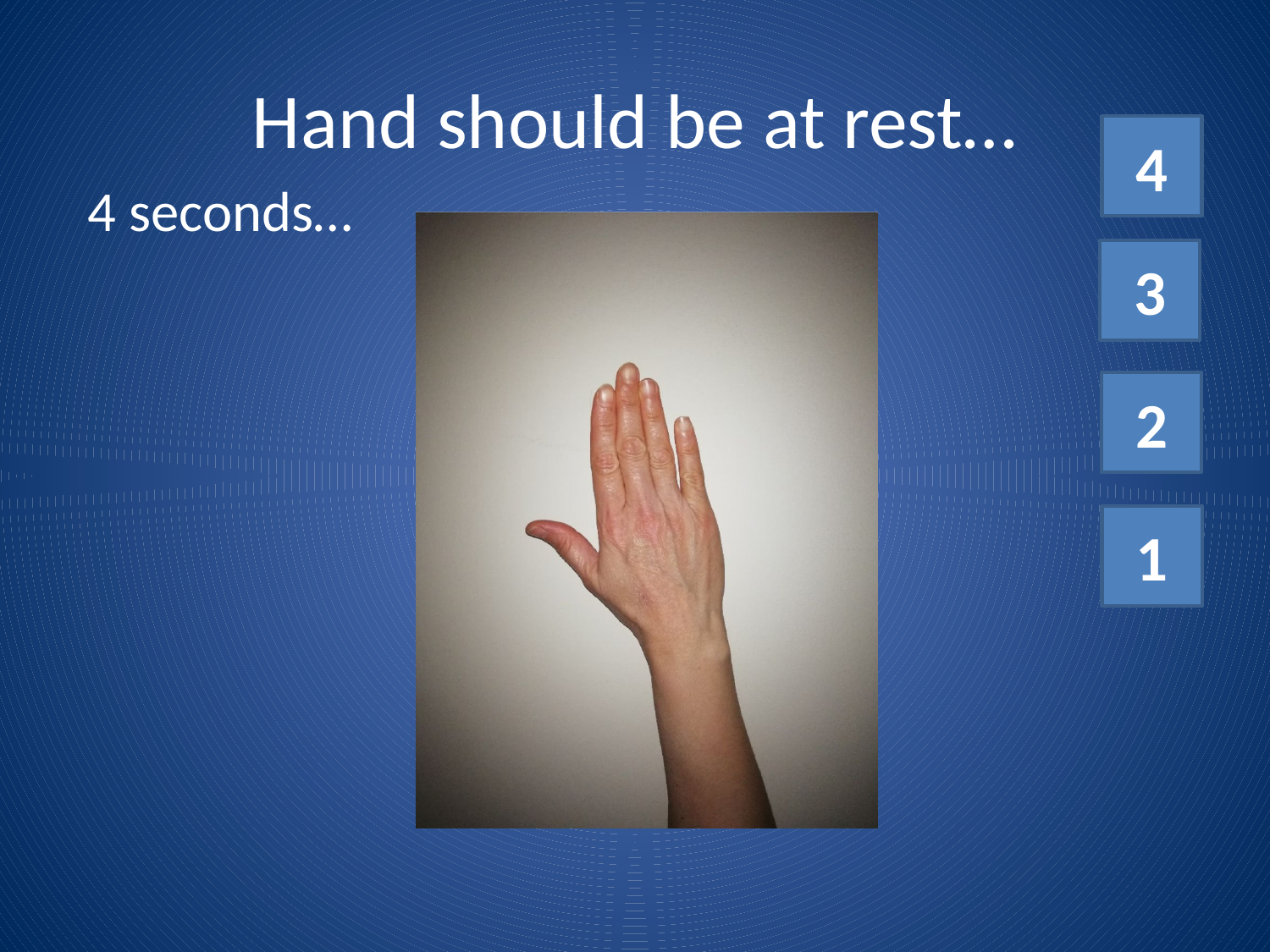

# Hand should be at rest…
4
4 seconds…
3
2
1

## Slide 26
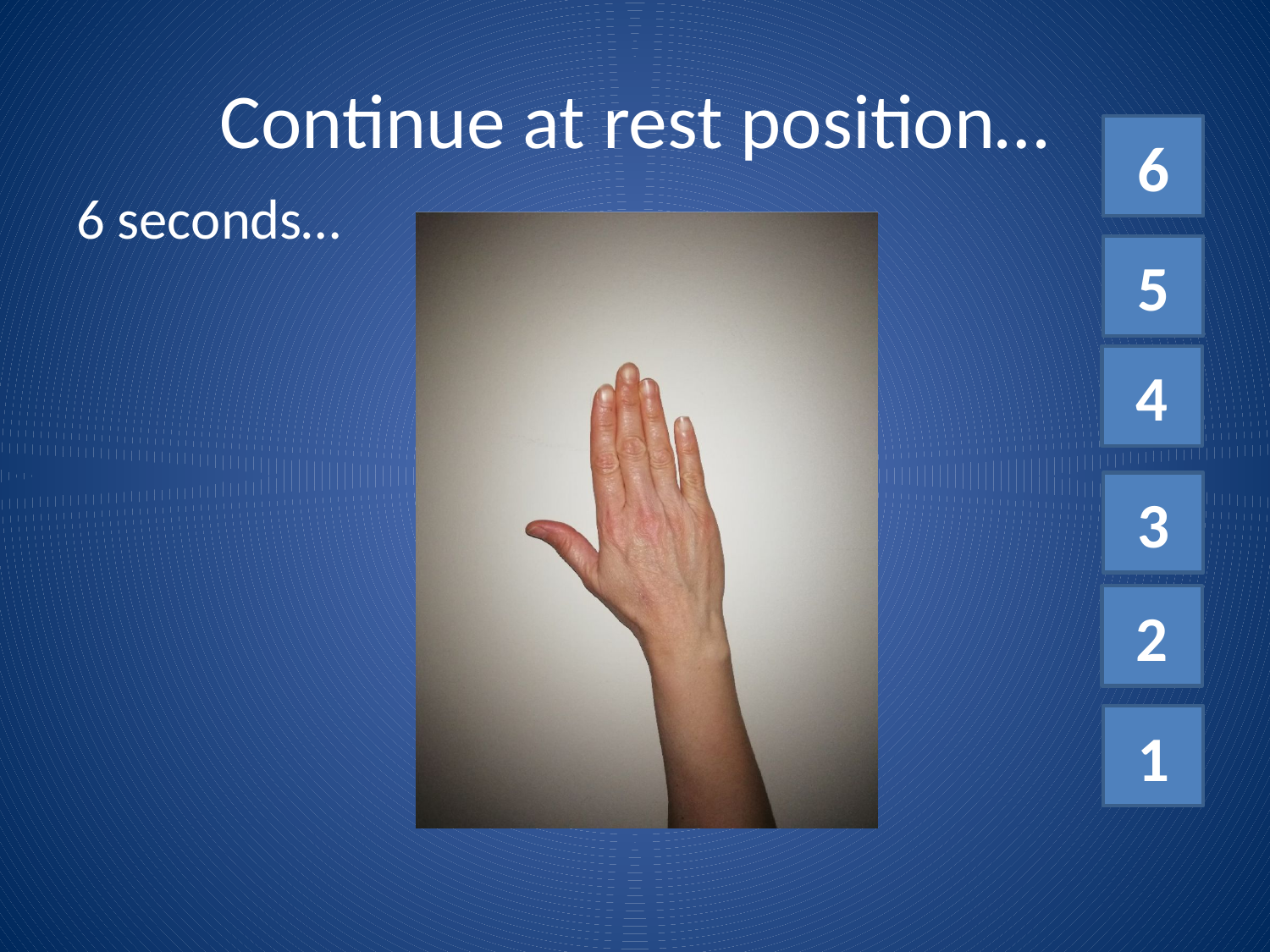

# Continue at rest position…
6
6 seconds…
5
4
3
2
1

## Slide 27
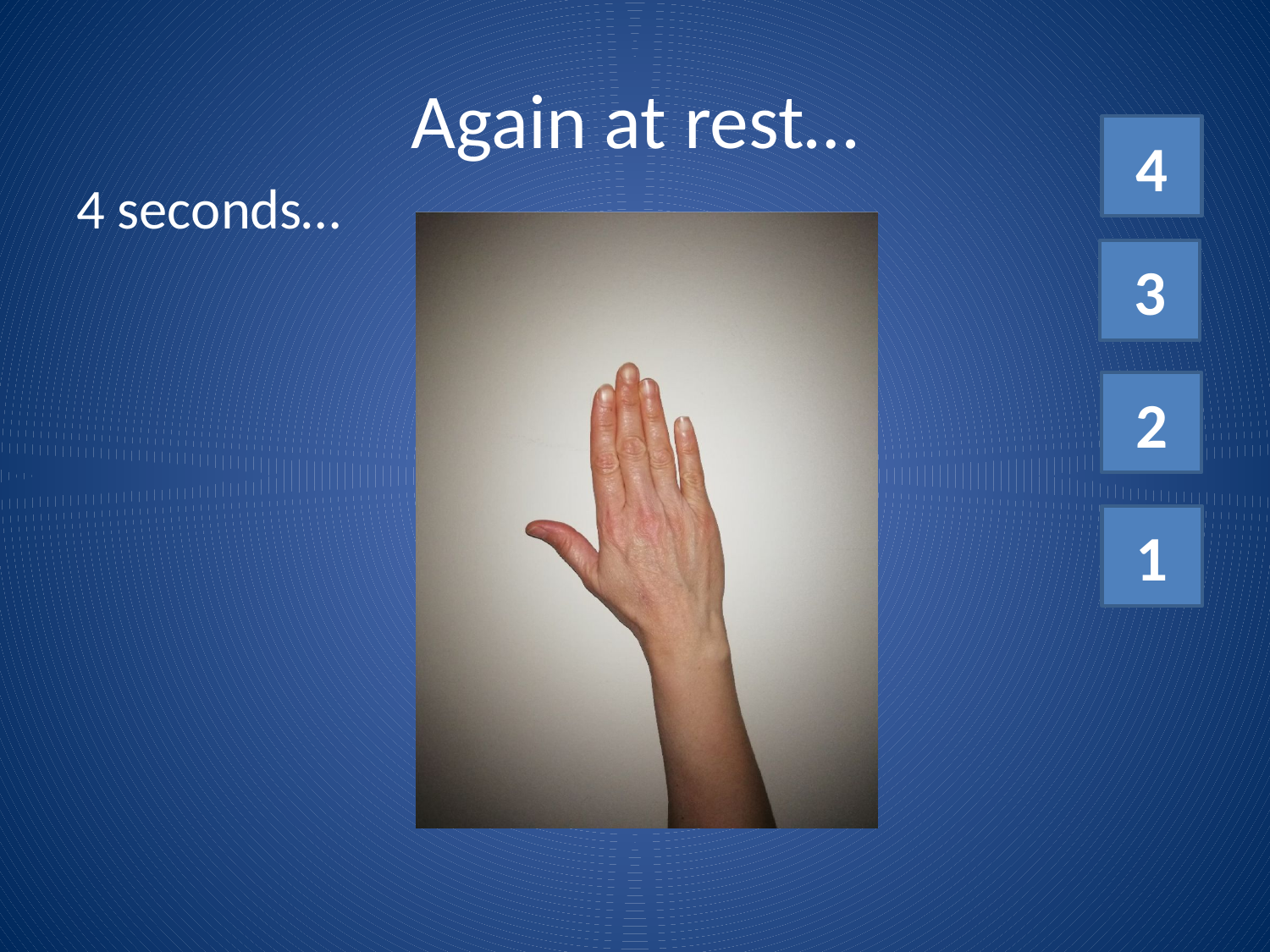

# Again at rest…
4
4 seconds…
3
2
1

## Slide 28
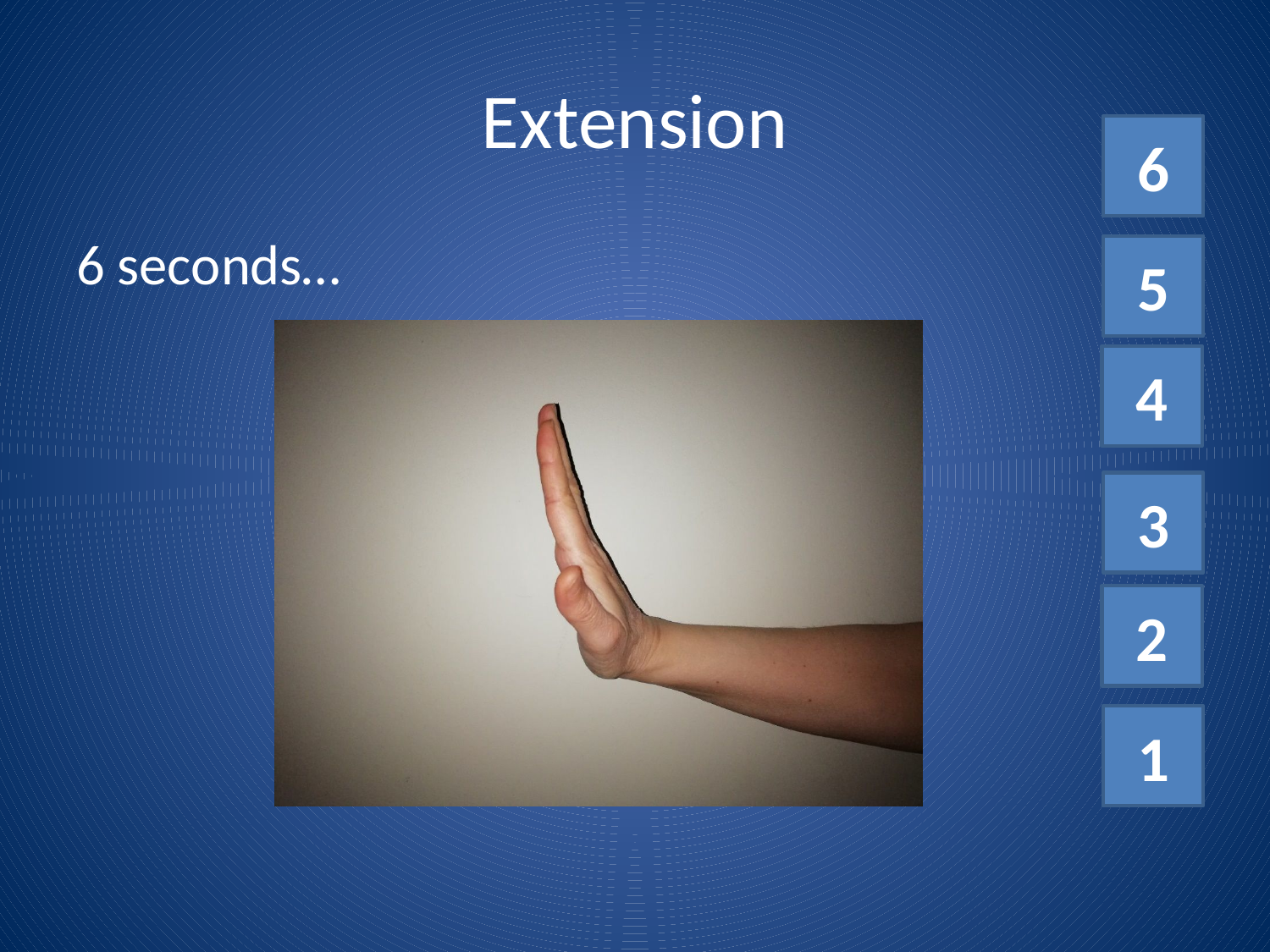

# Extension
6
6 seconds…
5
4
3
2
1

## Slide 29
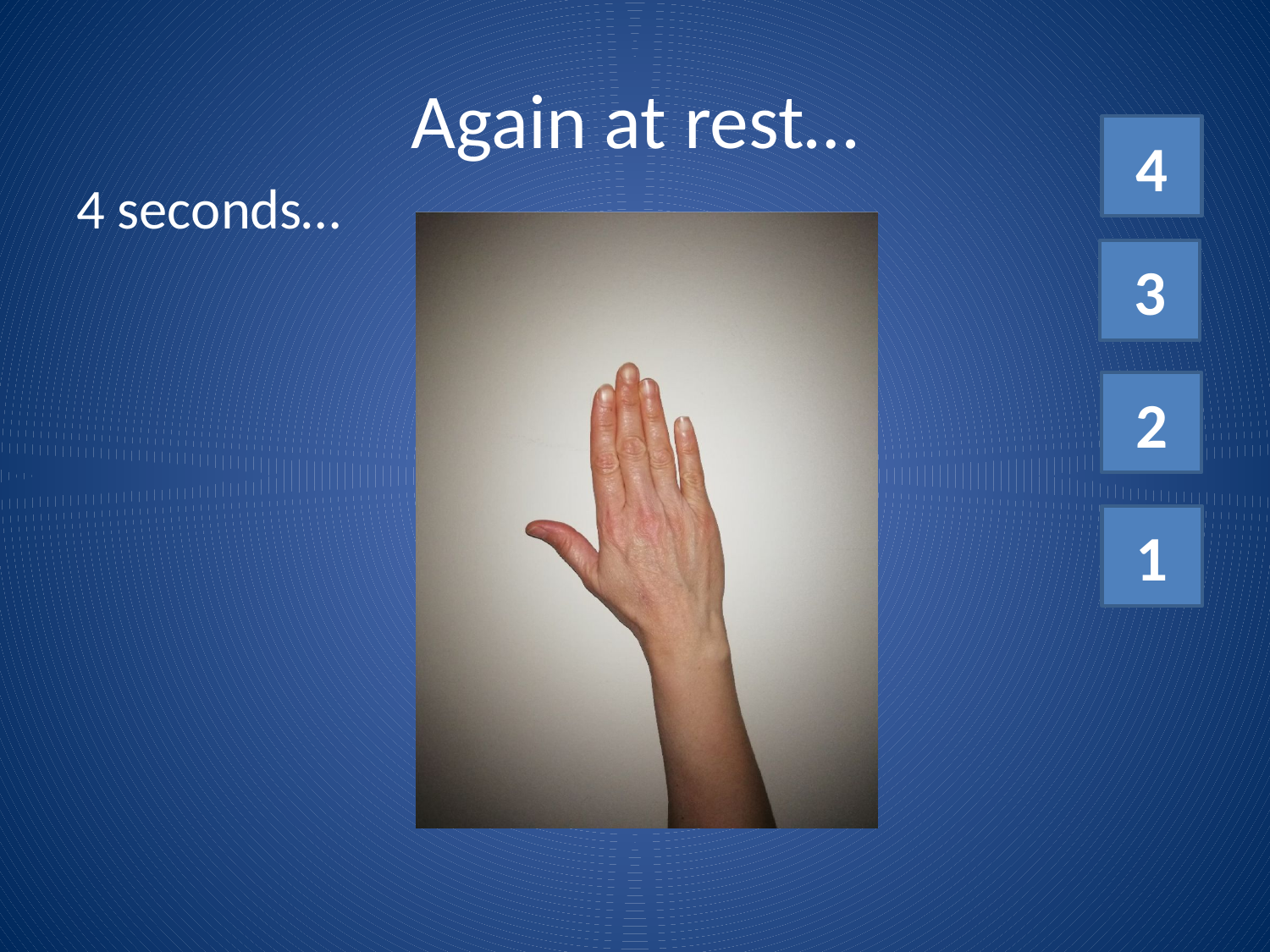

# Again at rest…
4
4 seconds…
3
2
1

## Slide 30
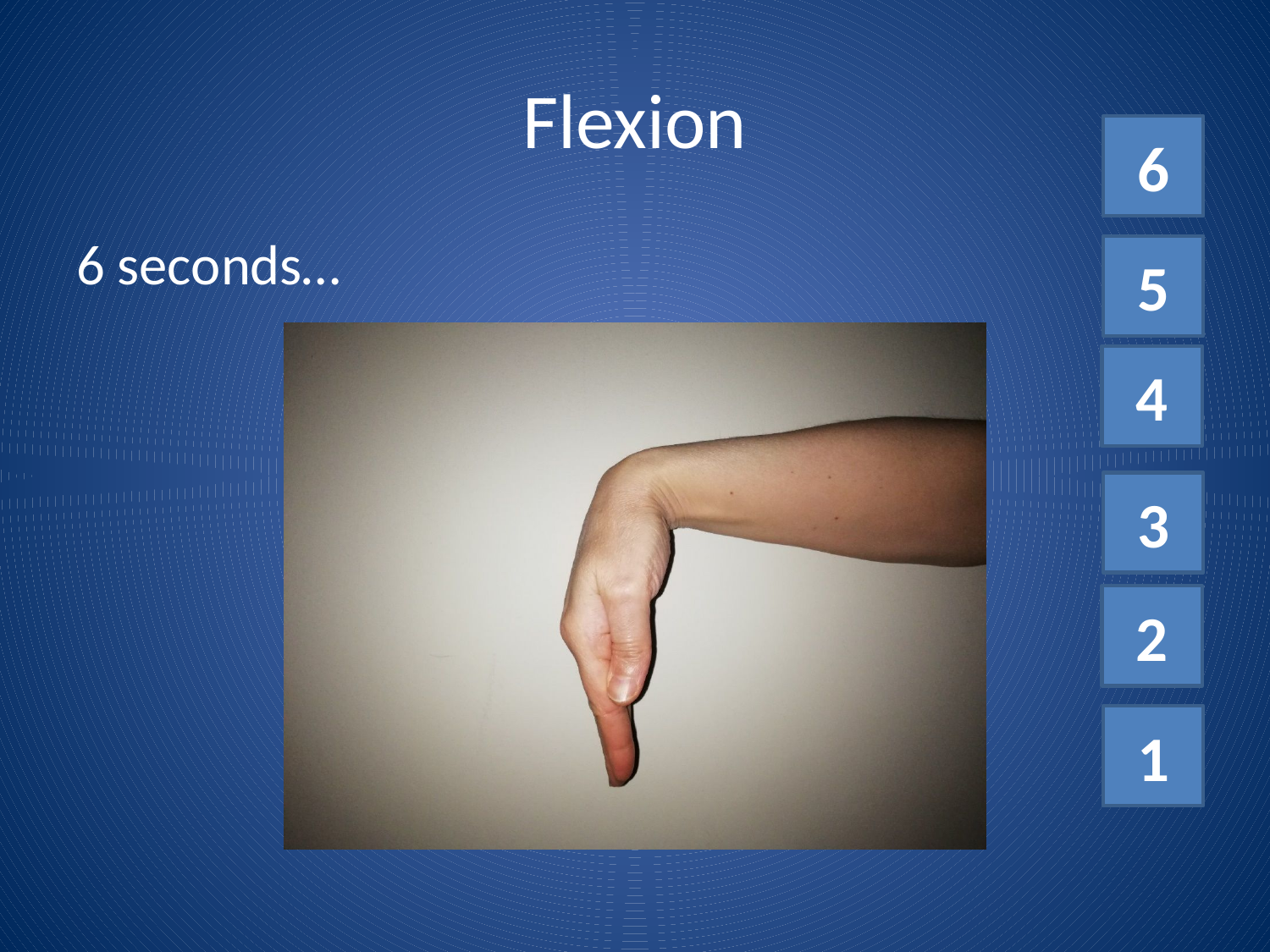

# Flexion
6
6 seconds…
5
4
3
2
1

## Slide 31
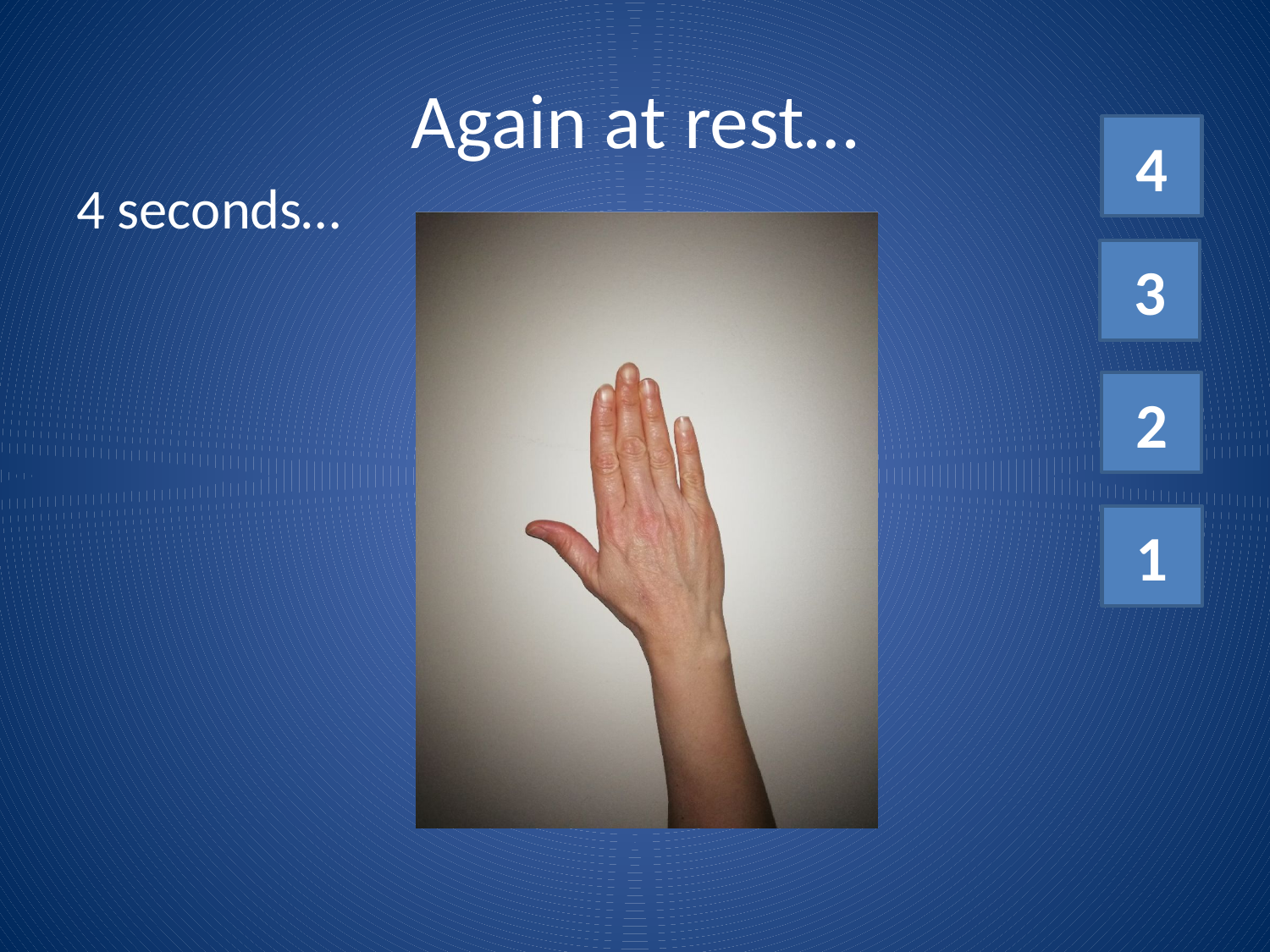

# Again at rest…
4
4 seconds…
3
2
1

## Slide 32
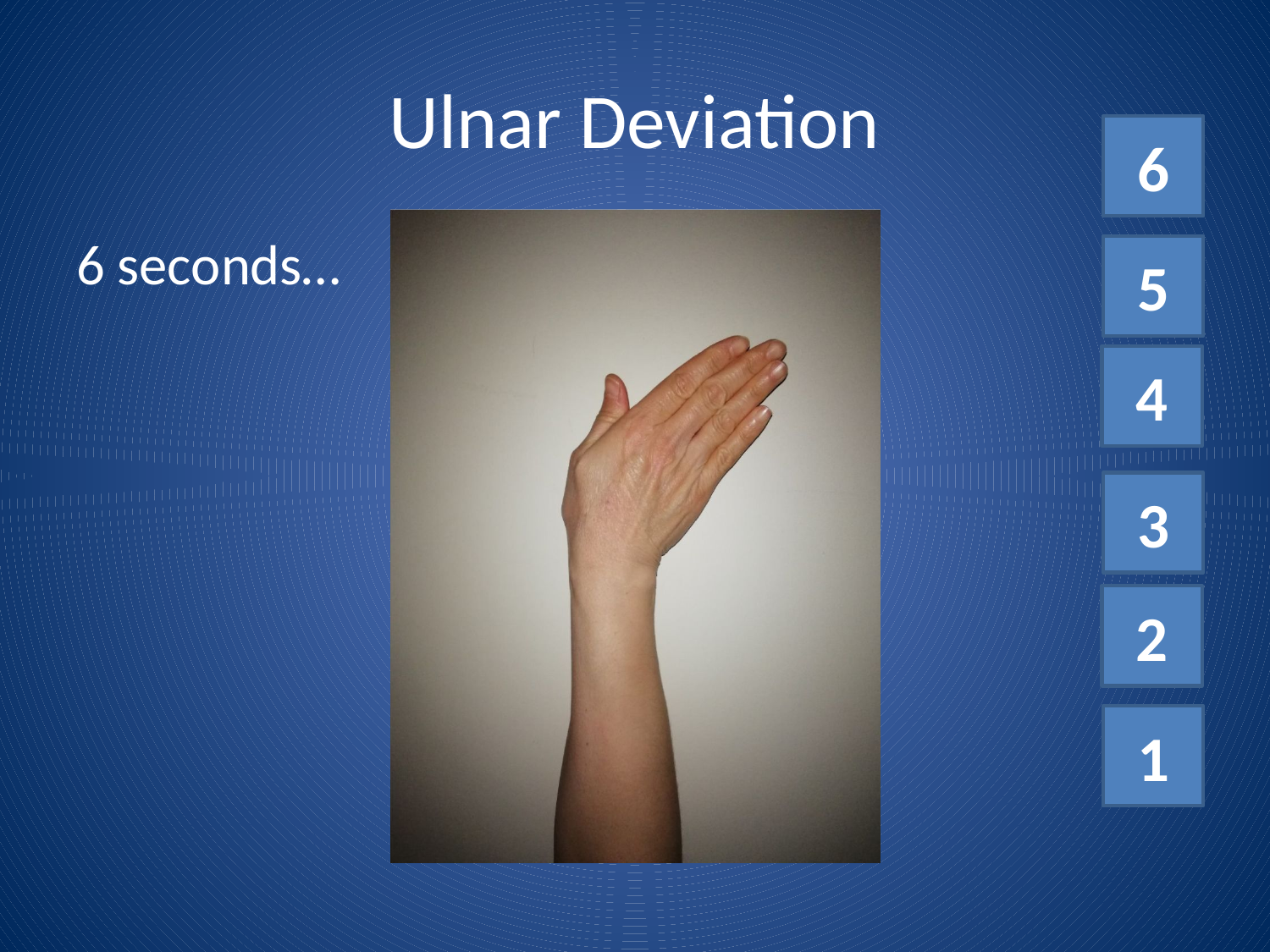

# Ulnar Deviation
6
6 seconds…
5
4
3
2
1

## Slide 33
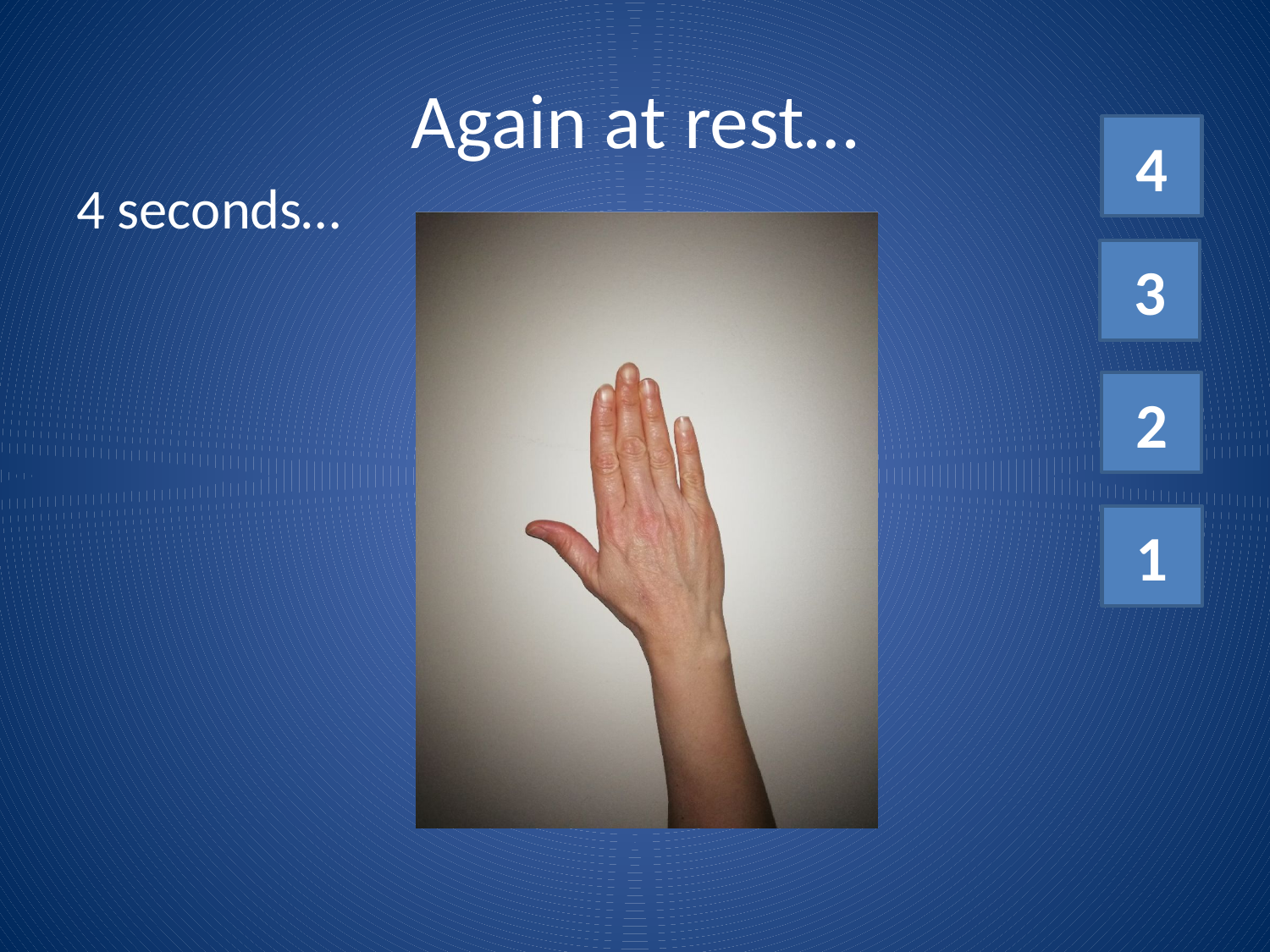

# Again at rest…
4
4 seconds…
3
2
1

## Slide 34
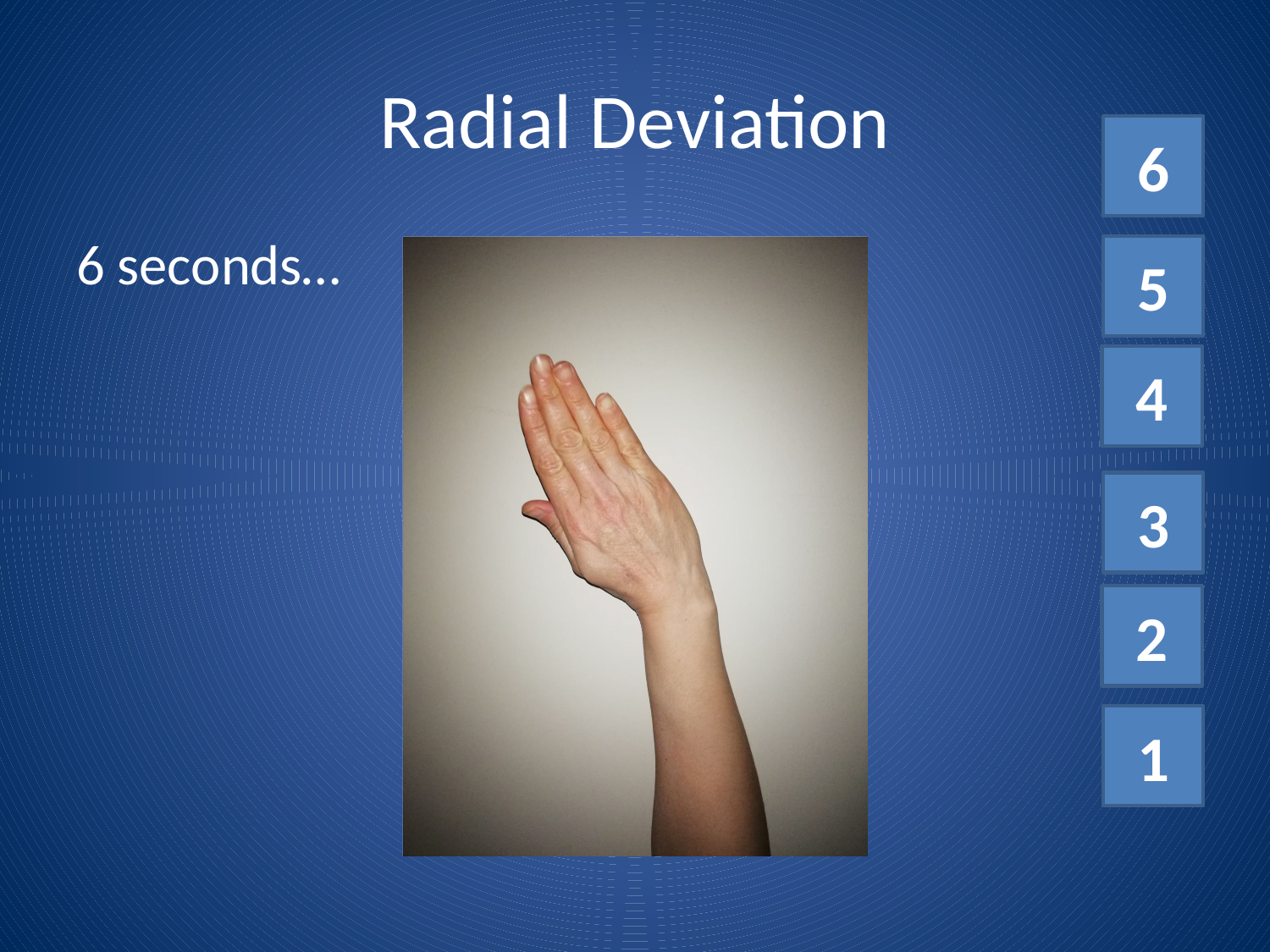

# Radial Deviation
6
6 seconds…
5
4
3
2
1

## Slide 35
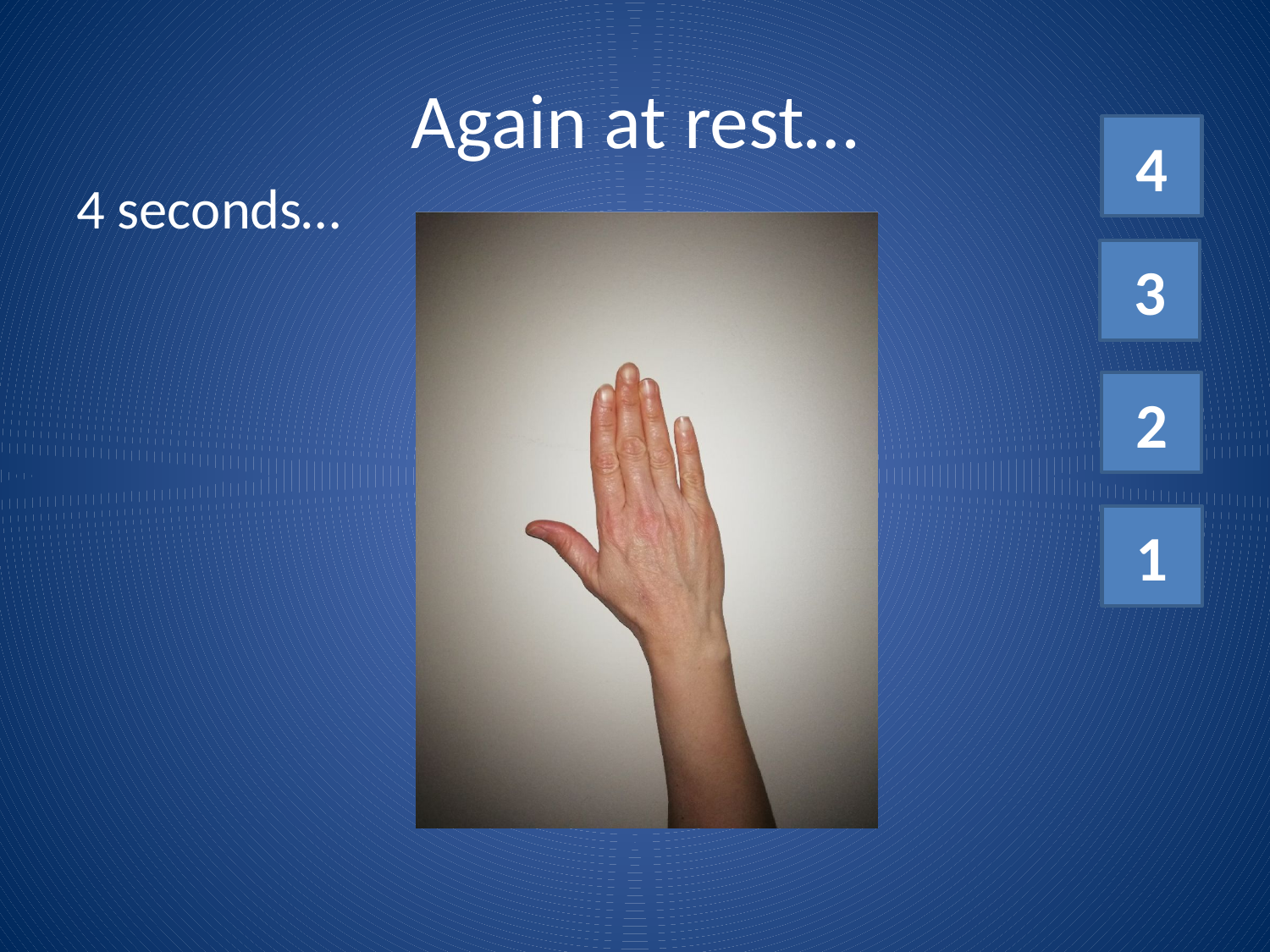

# Again at rest…
4
4 seconds…
3
2
1

## Slide 36
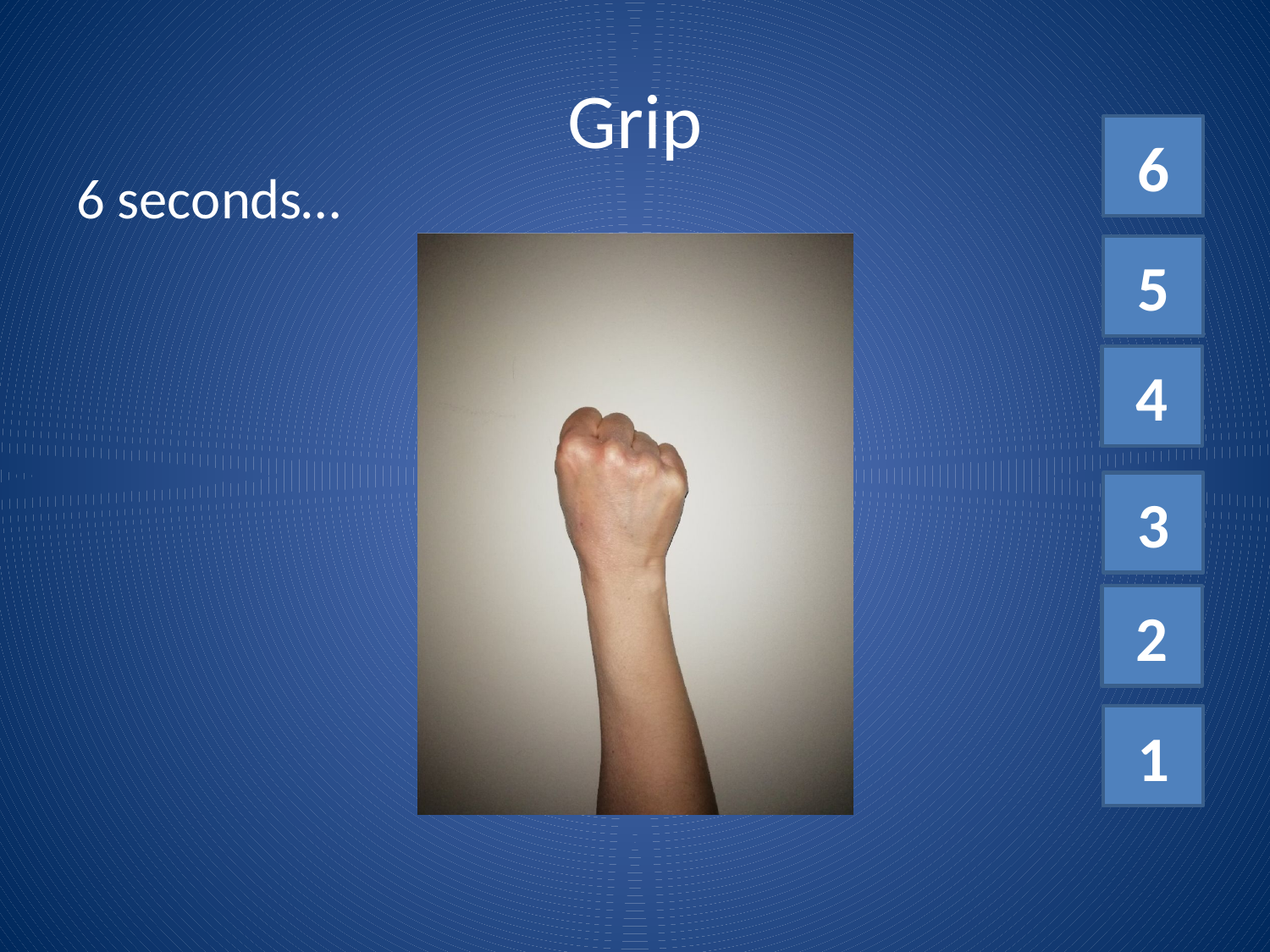

# Grip
6
6 seconds…
5
4
3
2
1

## Slide 37
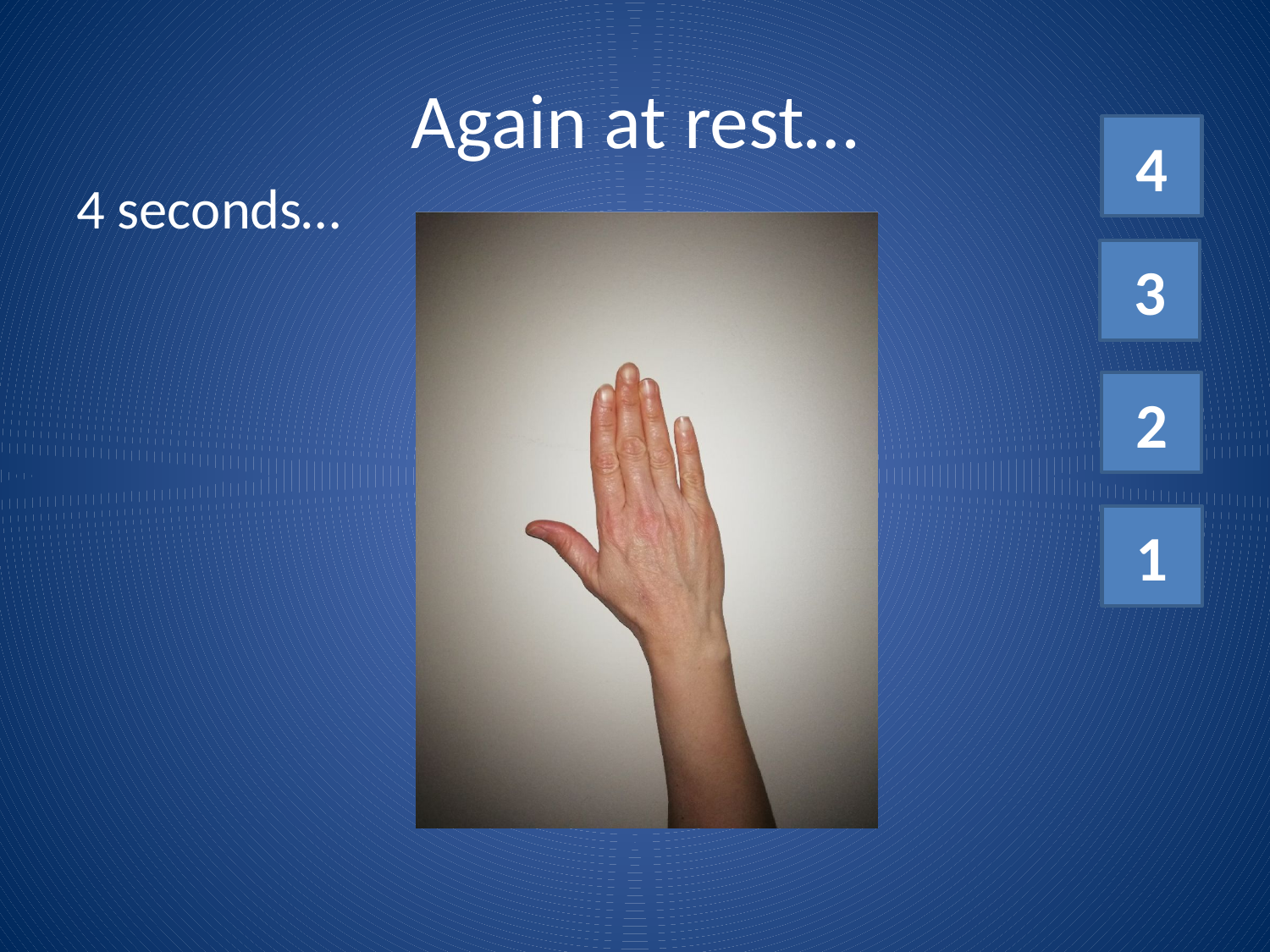

# Again at rest…
4
4 seconds…
3
2
1

## Slide 38
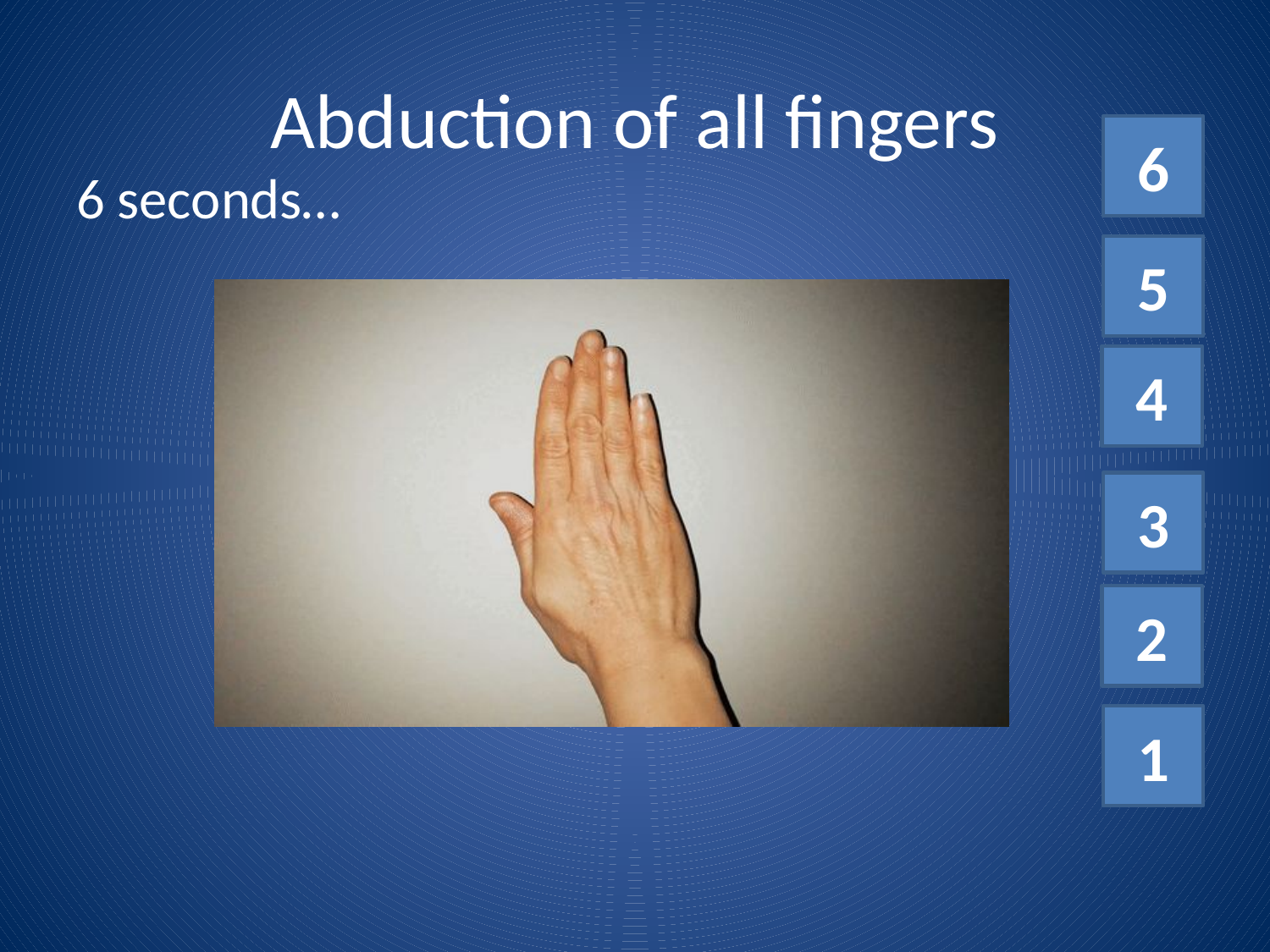

# Abduction of all fingers
6
6 seconds…
5
4
3
2
1

## Slide 39
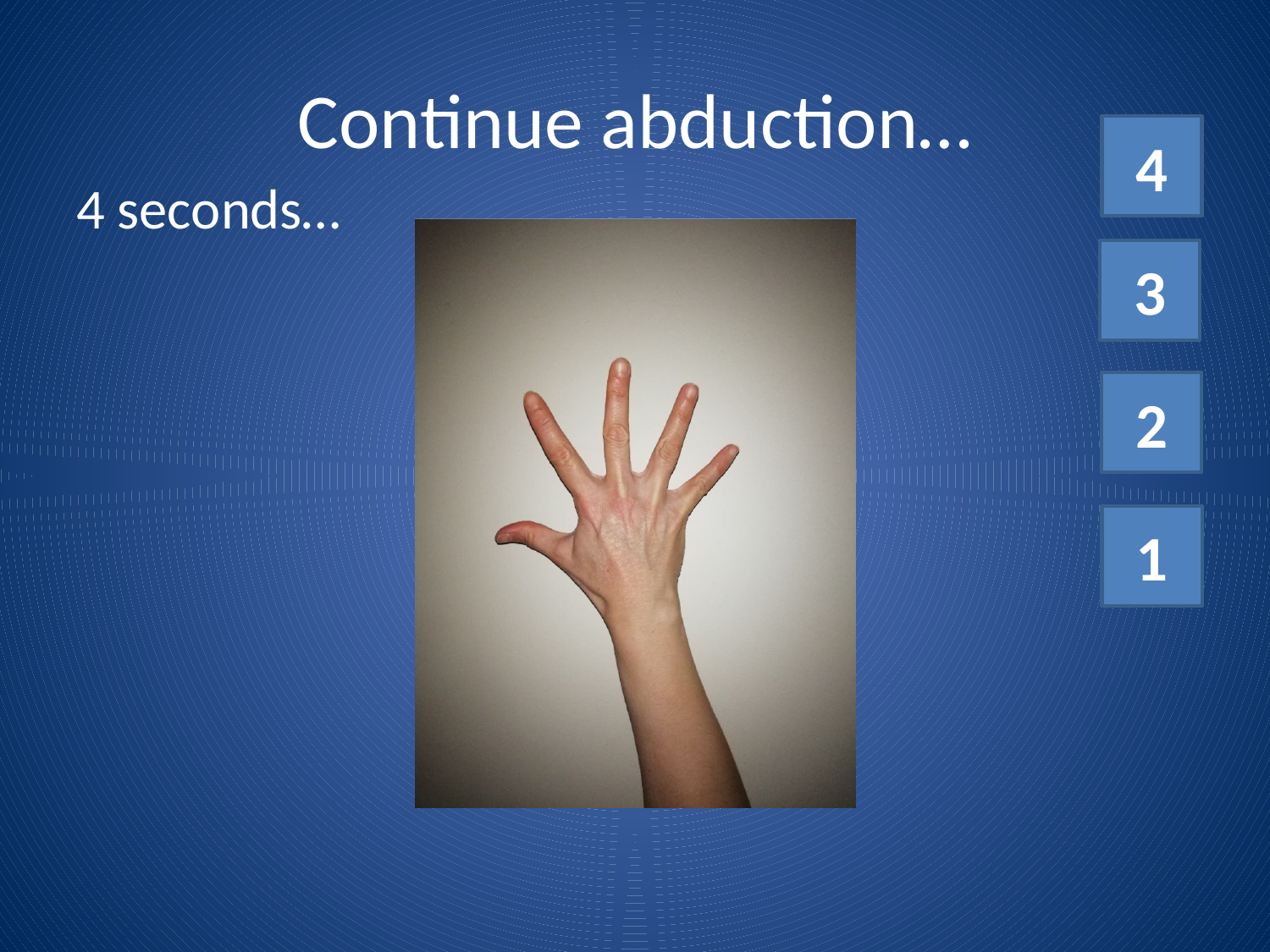

# Continue abduction…
4
4 seconds…
3
2
1

## Slide 40
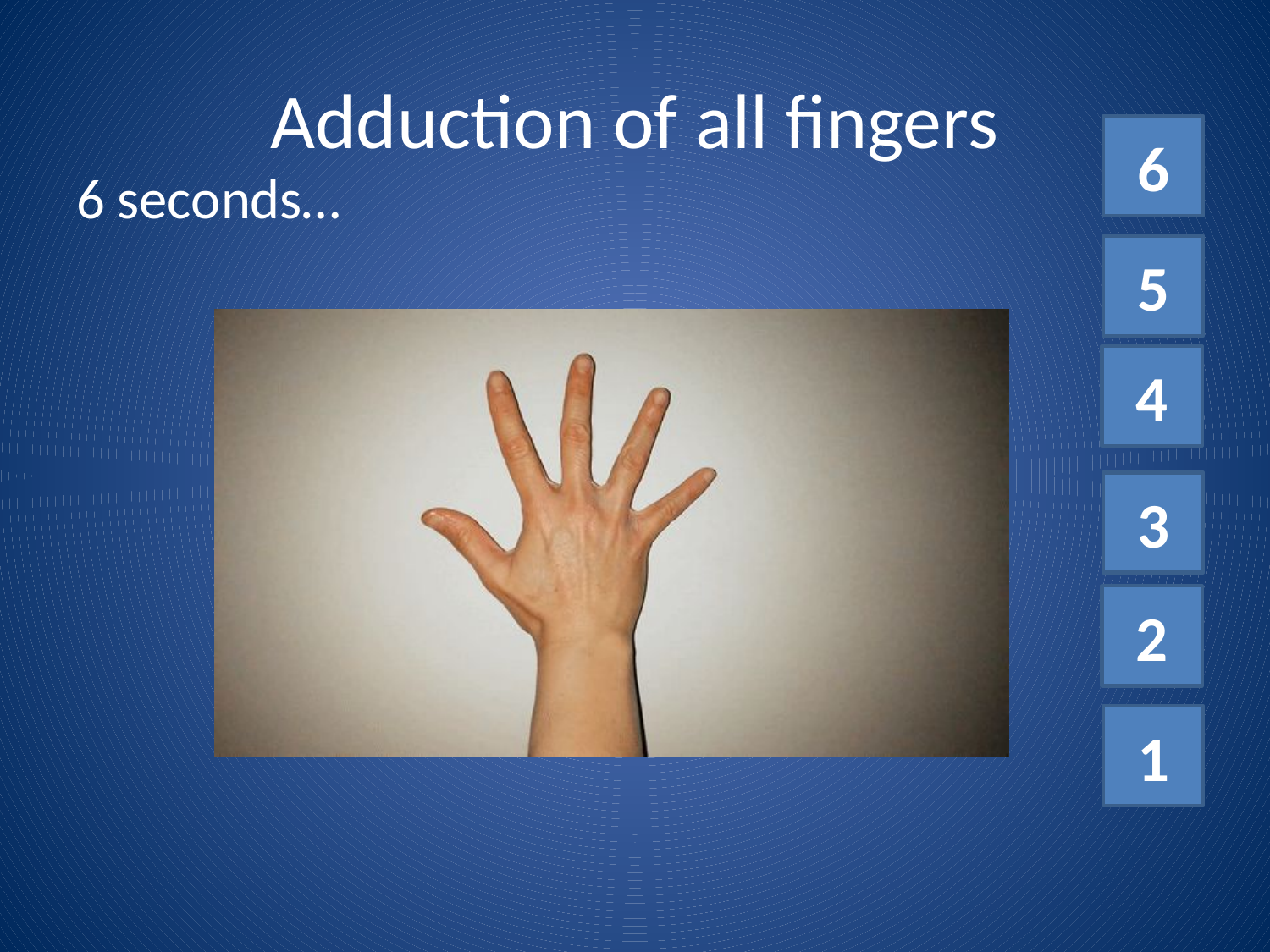

# Adduction of all fingers
6
6 seconds…
5
4
3
2
1

## Slide 41
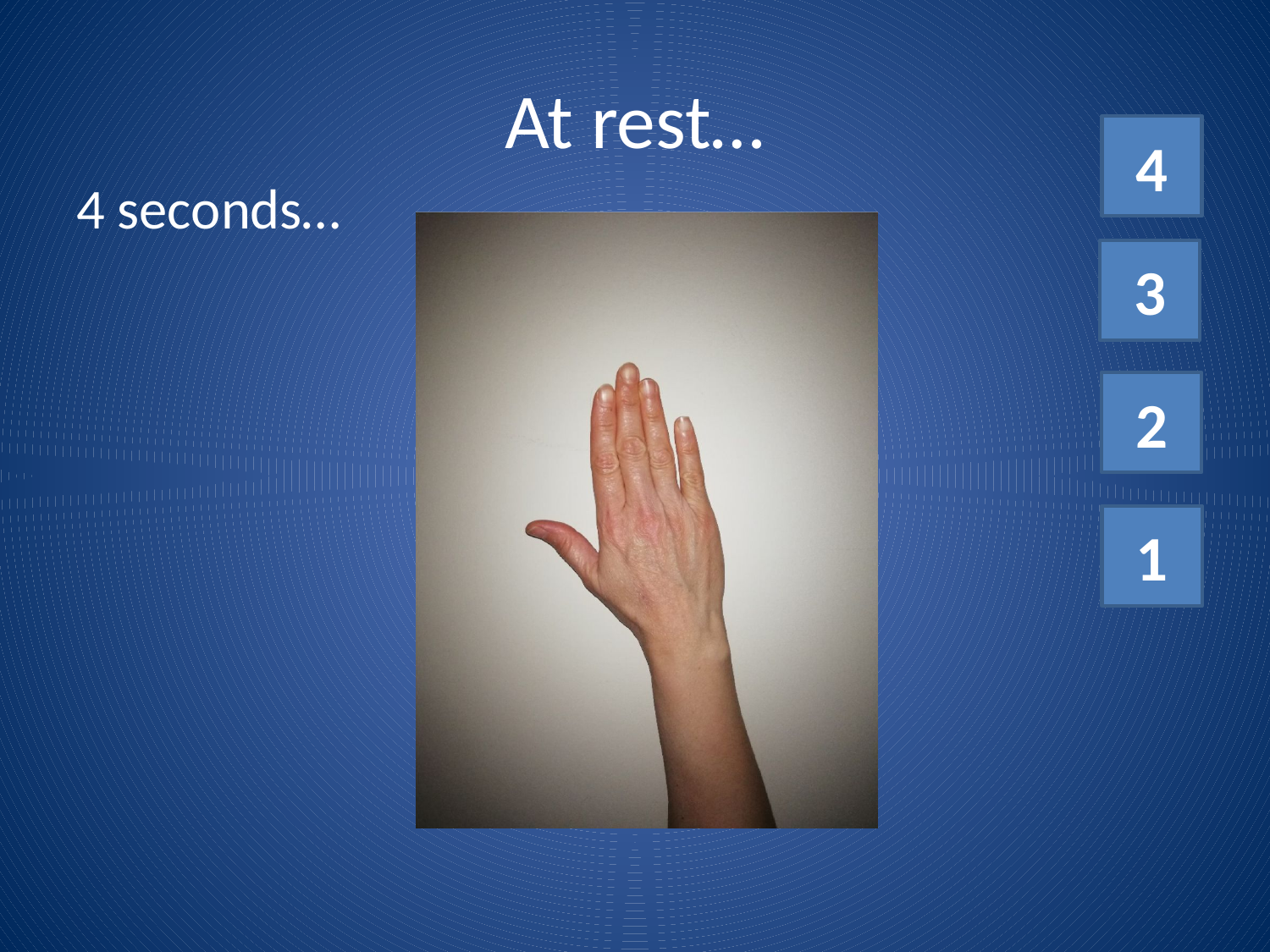

# At rest…
4
4 seconds…
3
2
1

## Slide 42
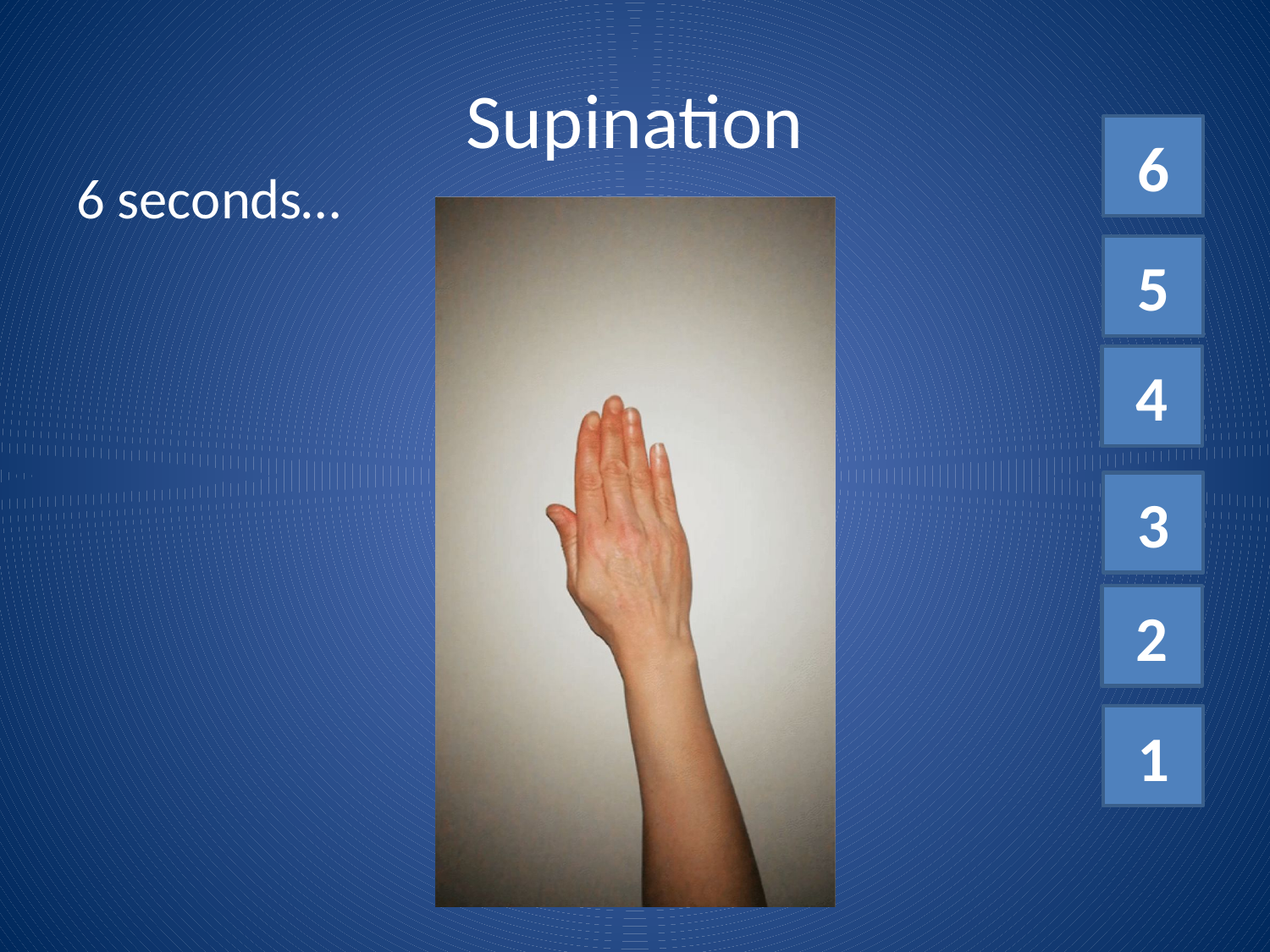

# Supination
6
6 seconds…
5
4
3
2
1

## Slide 43
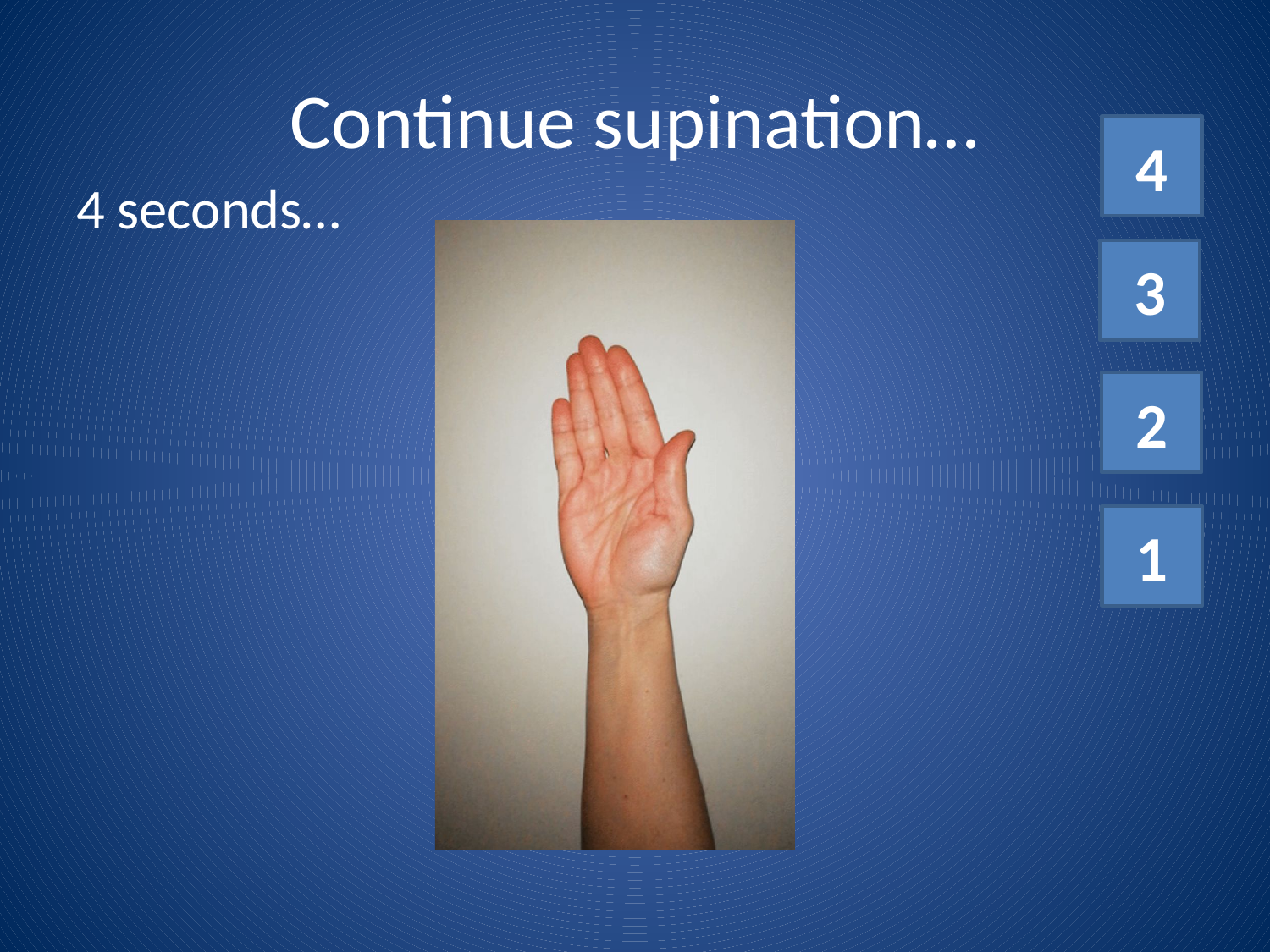

# Continue supination…
4
4 seconds…
3
2
1

## Slide 44
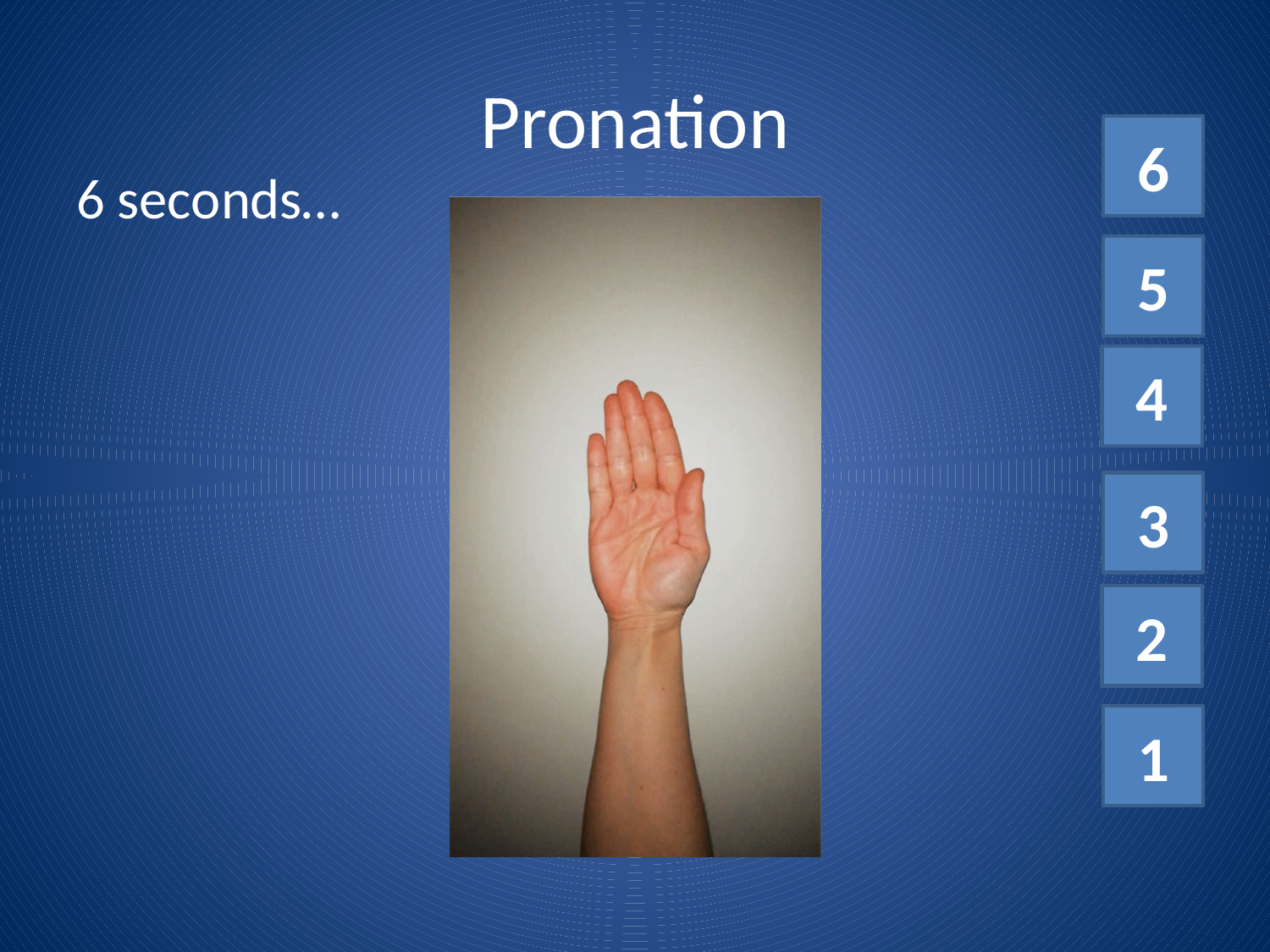

# Pronation
6
6 seconds…
5
4
3
2
1

## Slide 45
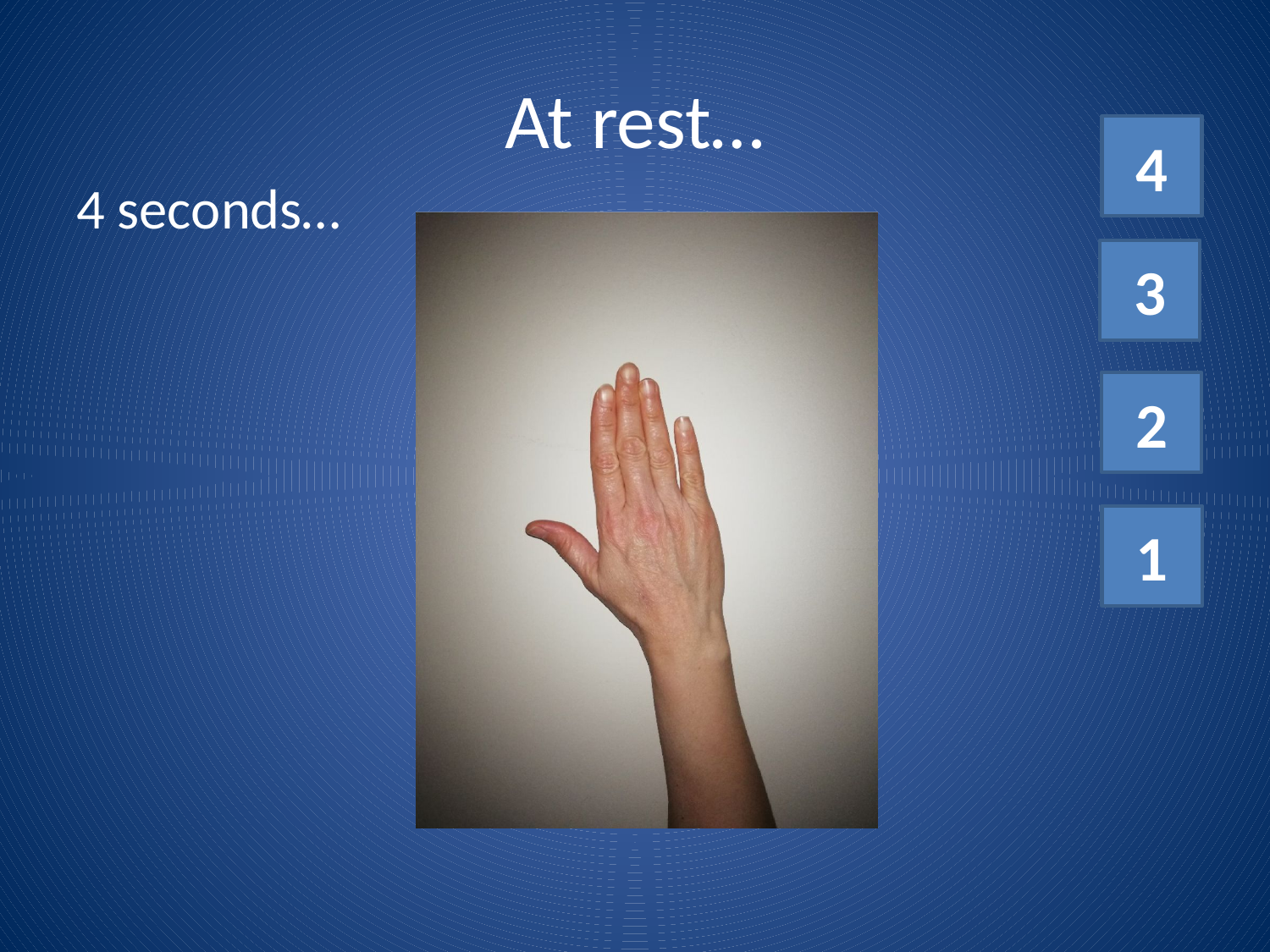

# At rest…
4
4 seconds…
3
2
1

## Slide 46
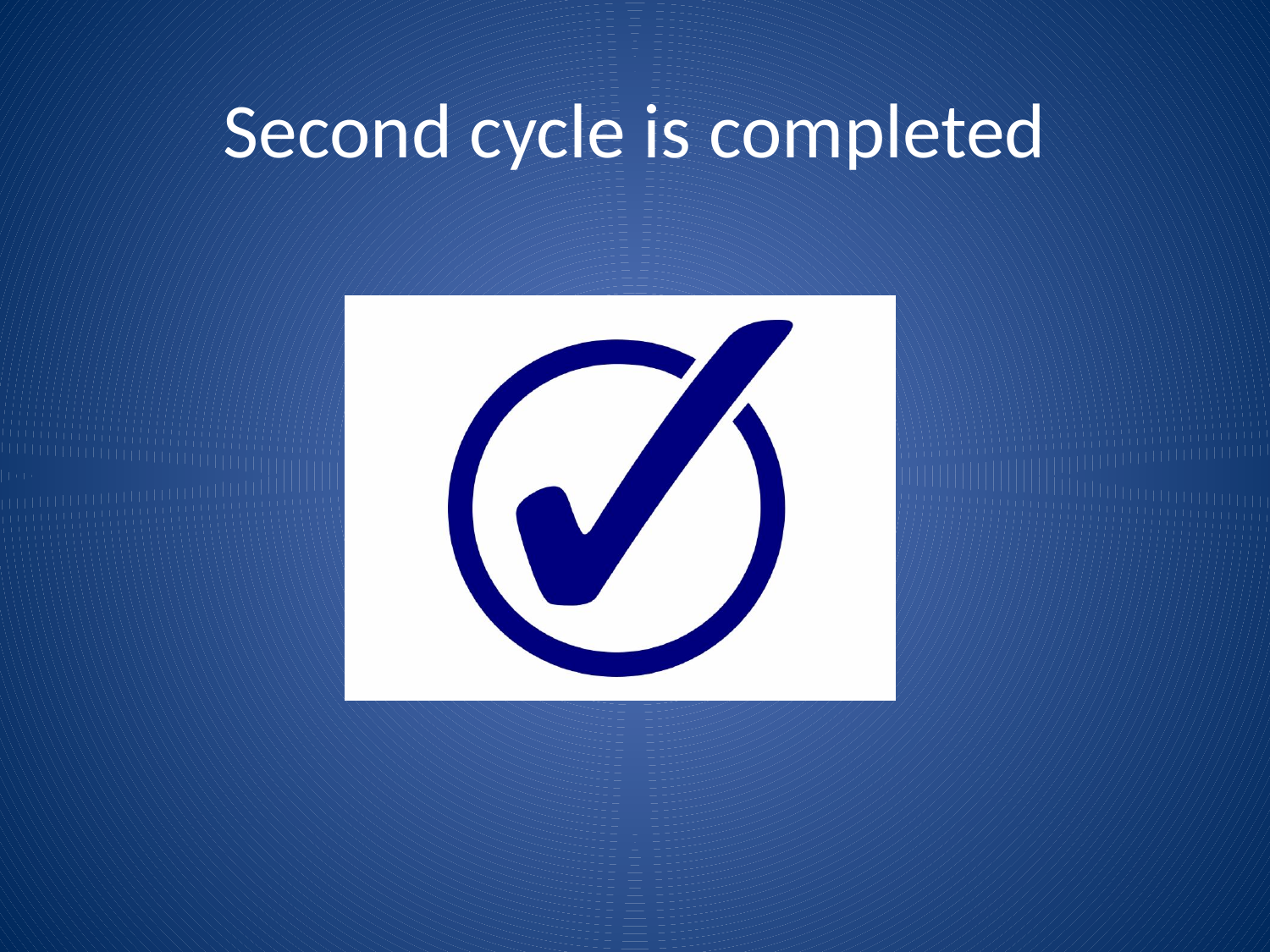

# Second cycle is completed

## Slide 47
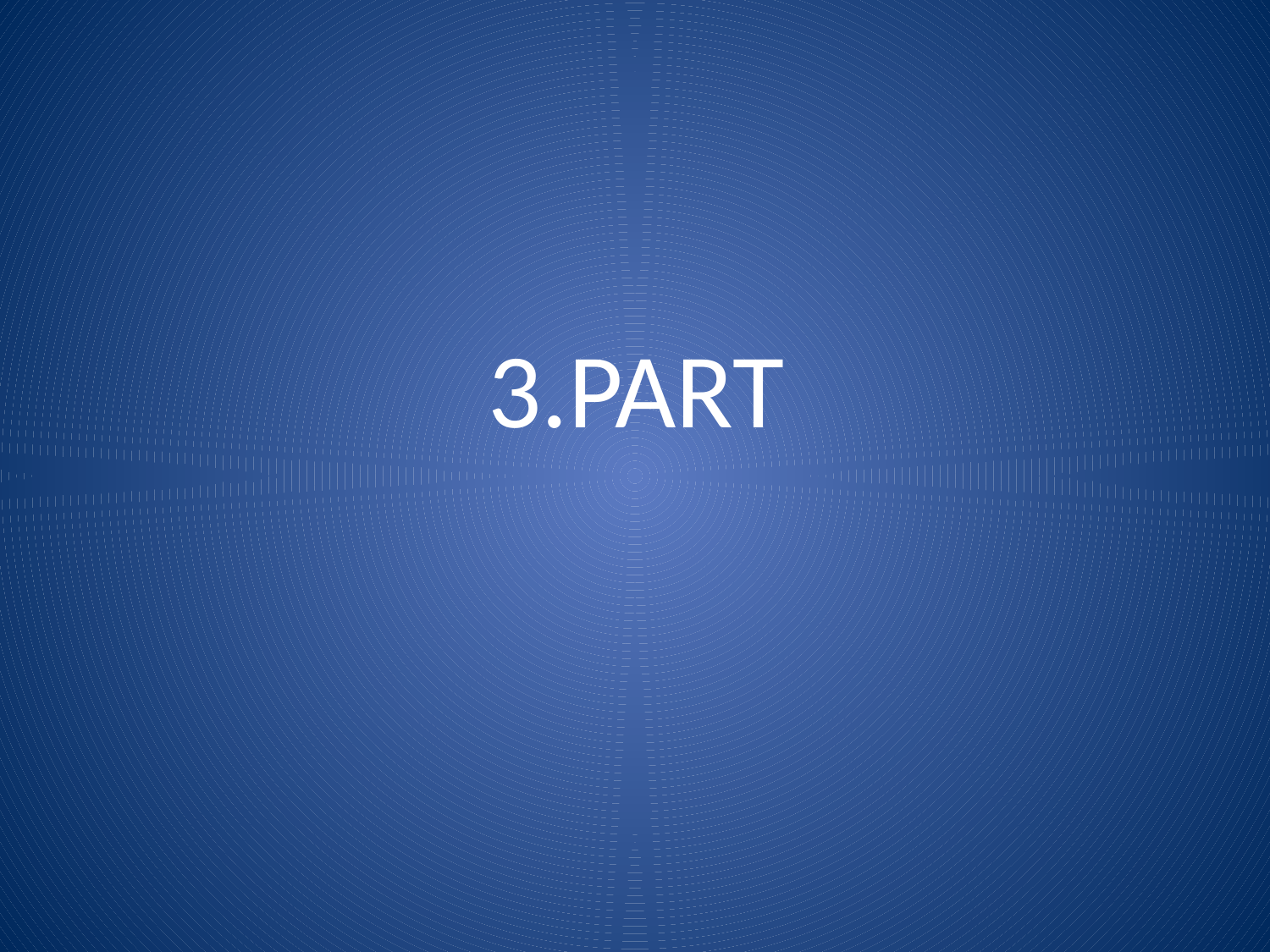

# 3.PART

## Slide 48
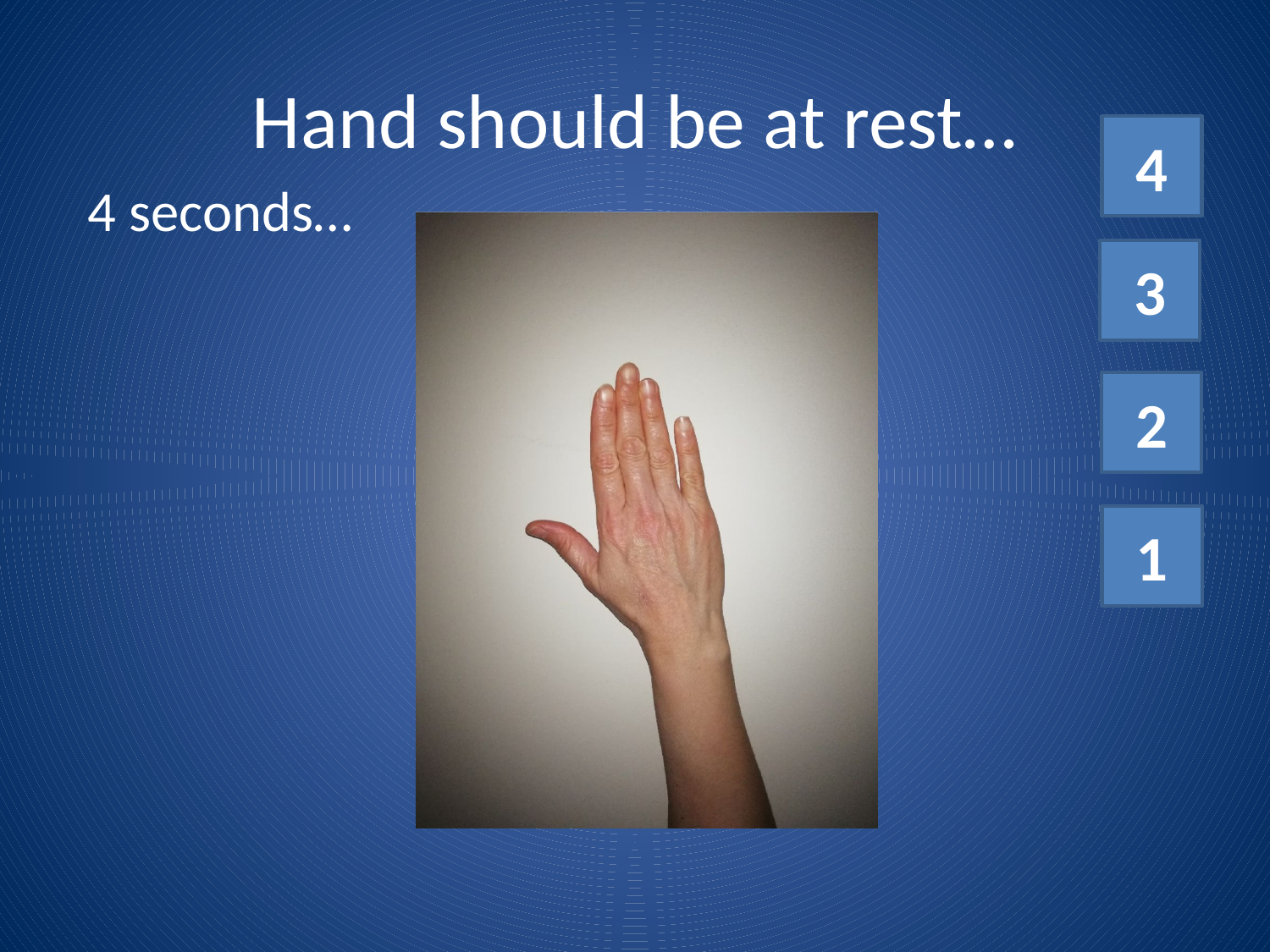

# Hand should be at rest…
4
4 seconds…
3
2
1

## Slide 49
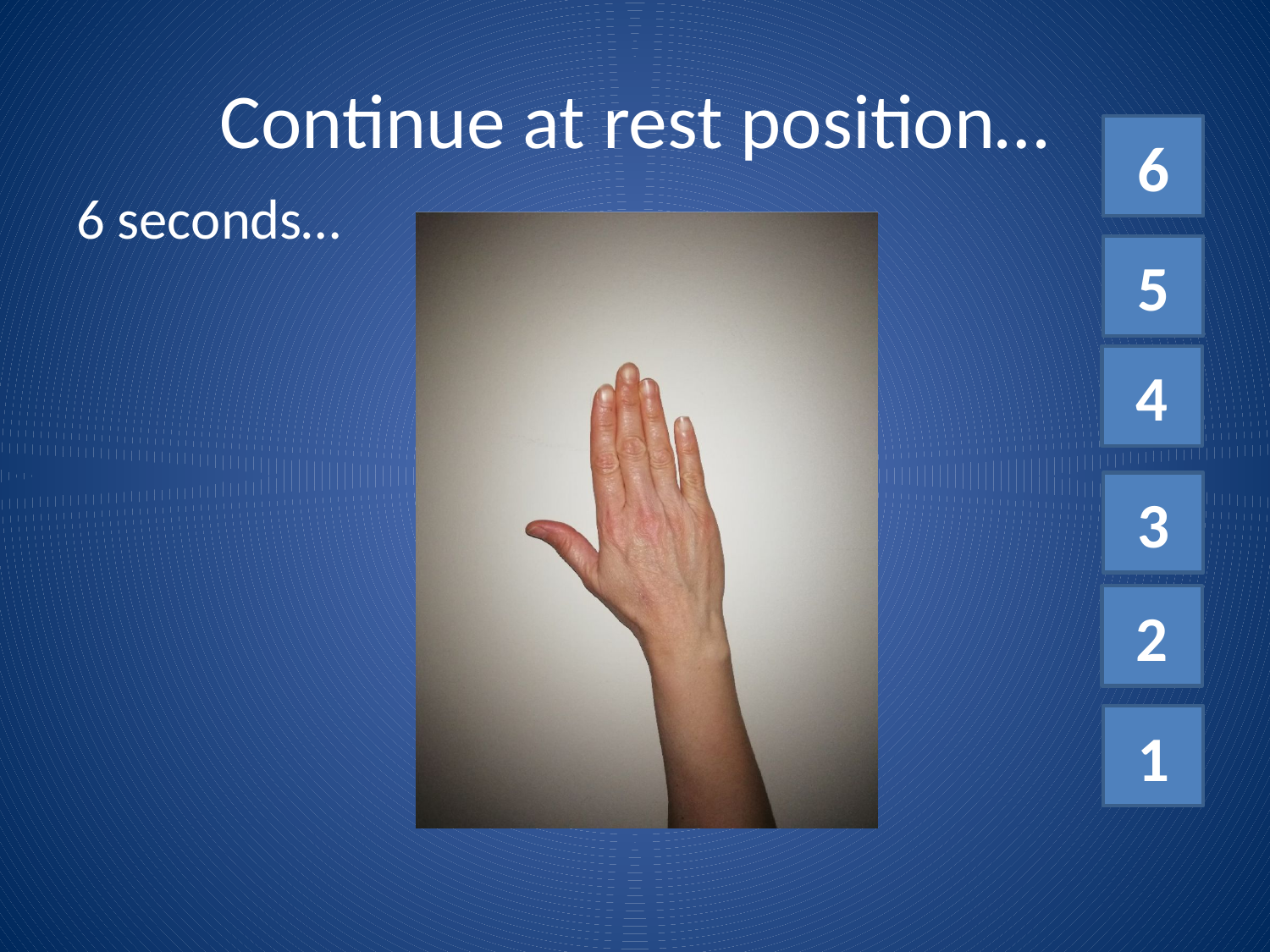

# Continue at rest position…
6
6 seconds…
5
4
3
2
1

## Slide 50
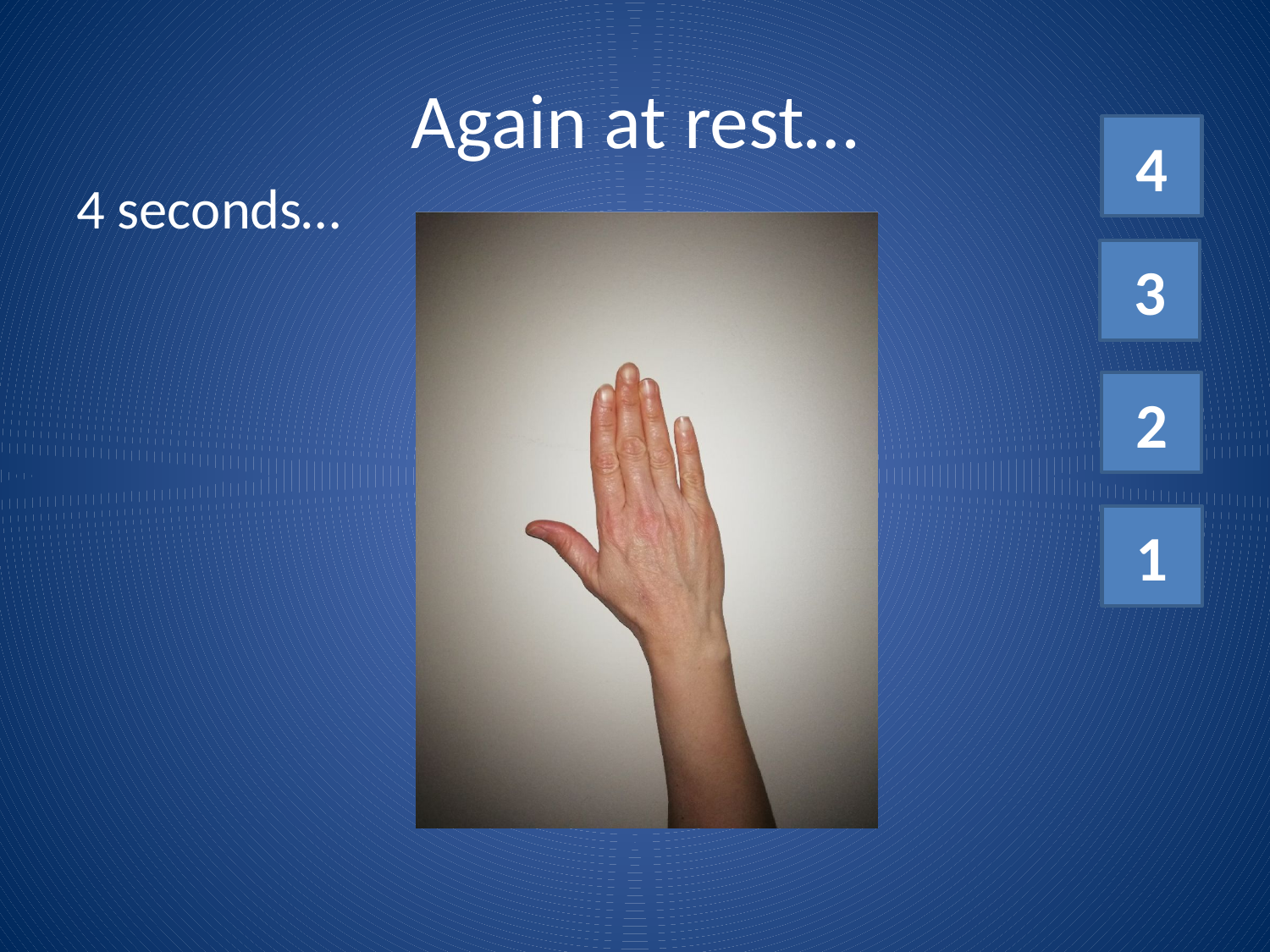

# Again at rest…
4
4 seconds…
3
2
1

## Slide 51
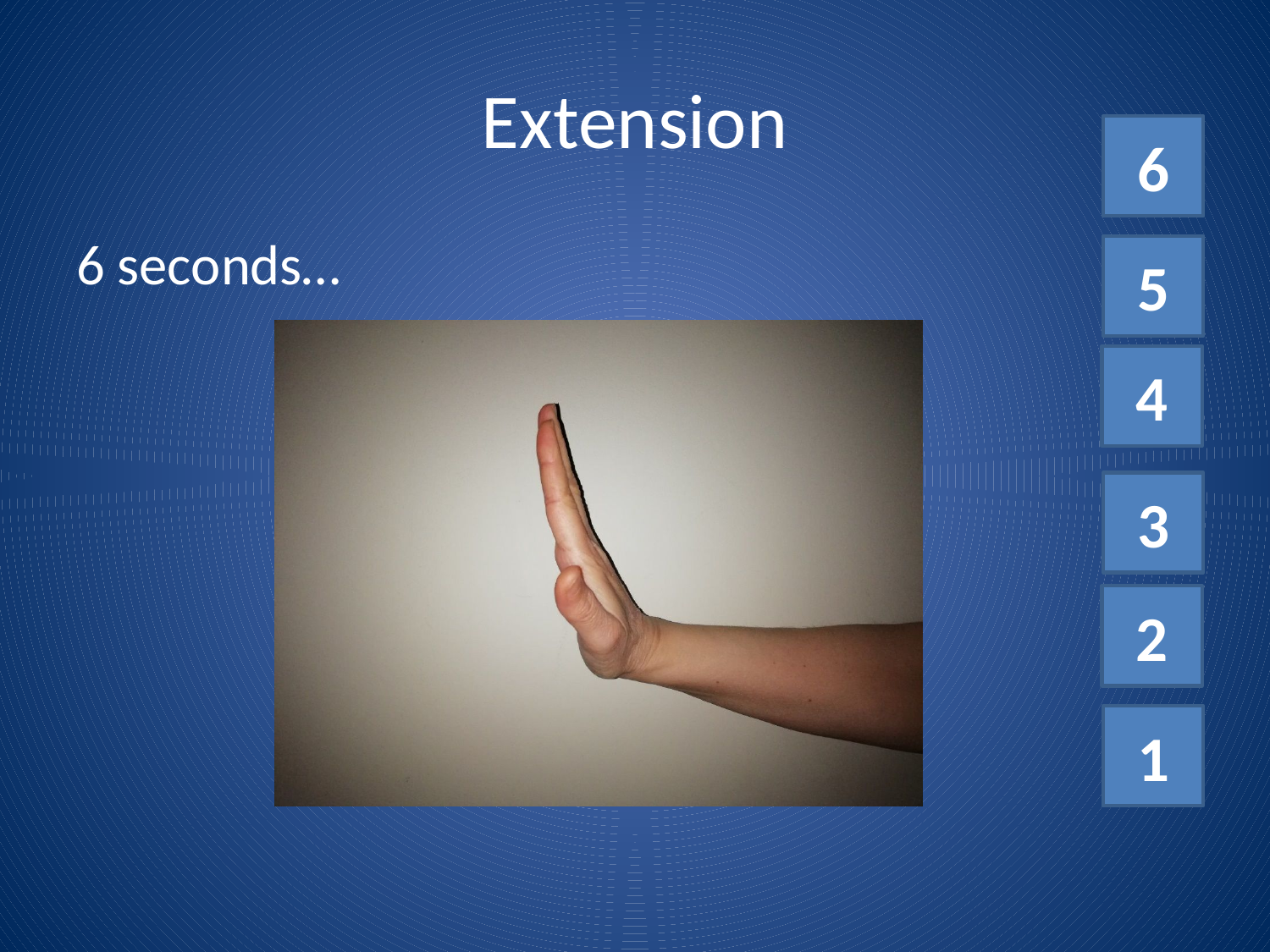

# Extension
6
6 seconds…
5
4
3
2
1

## Slide 52
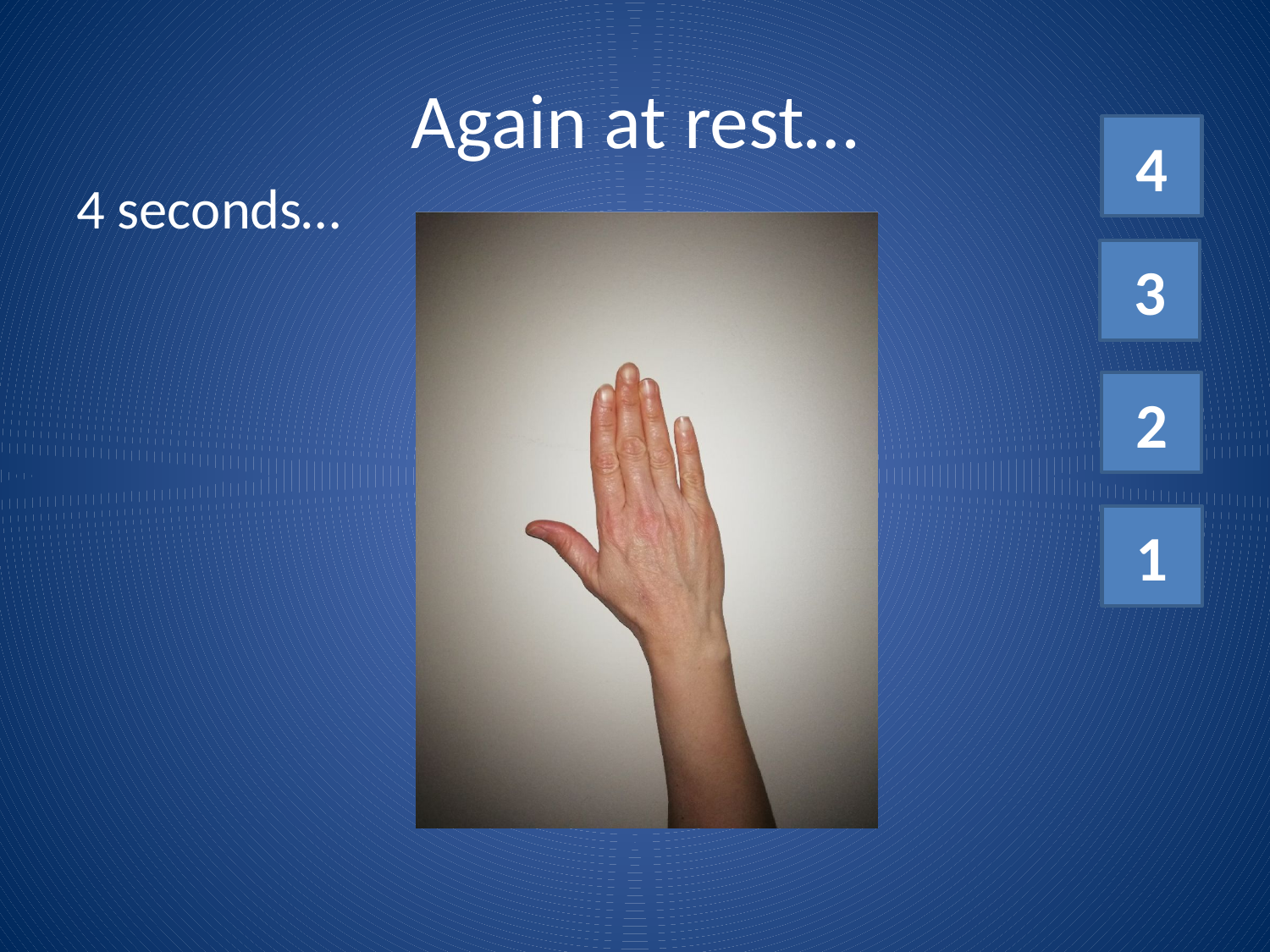

# Again at rest…
4
4 seconds…
3
2
1

## Slide 53
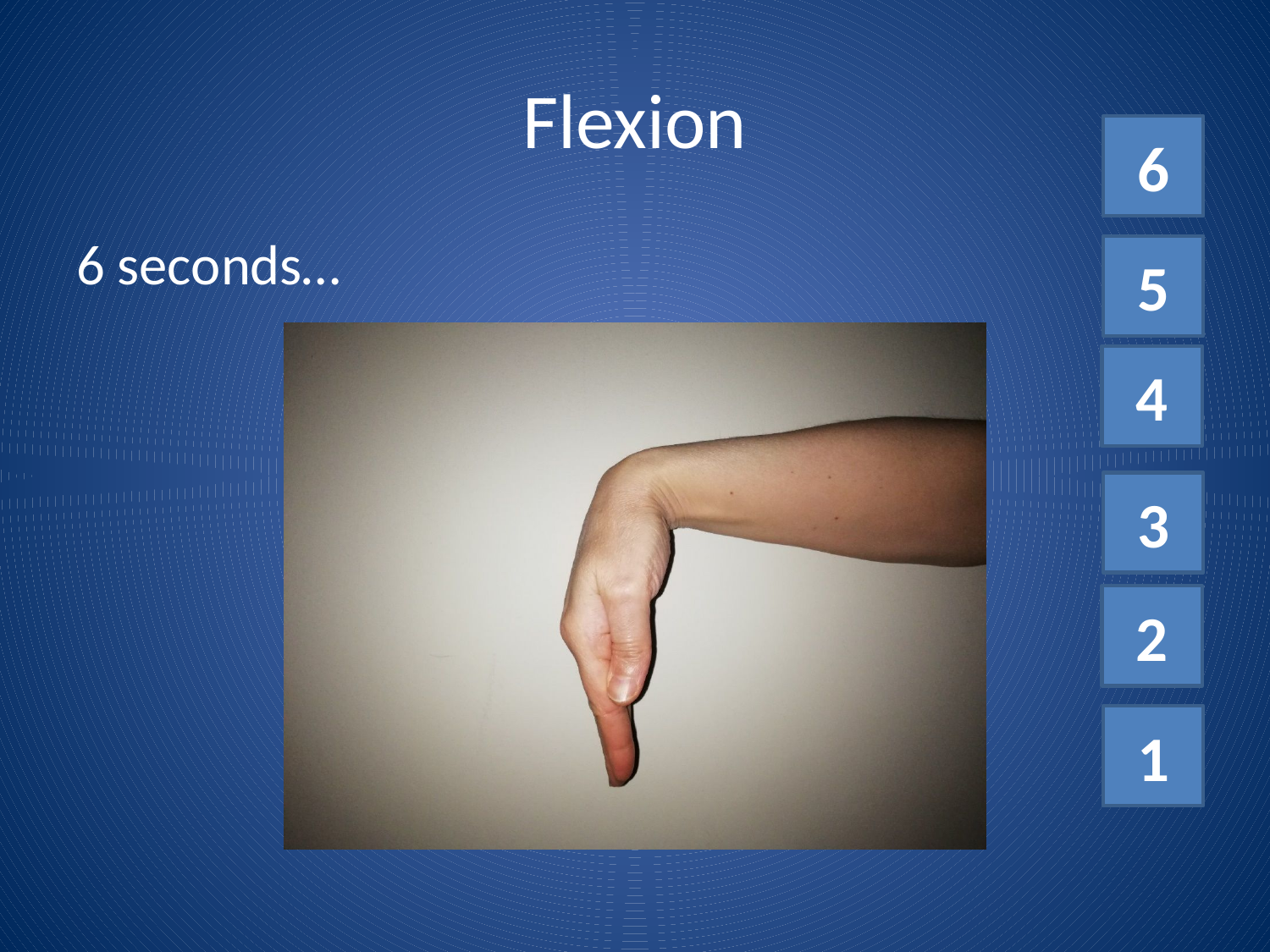

# Flexion
6
6 seconds…
5
4
3
2
1

## Slide 54
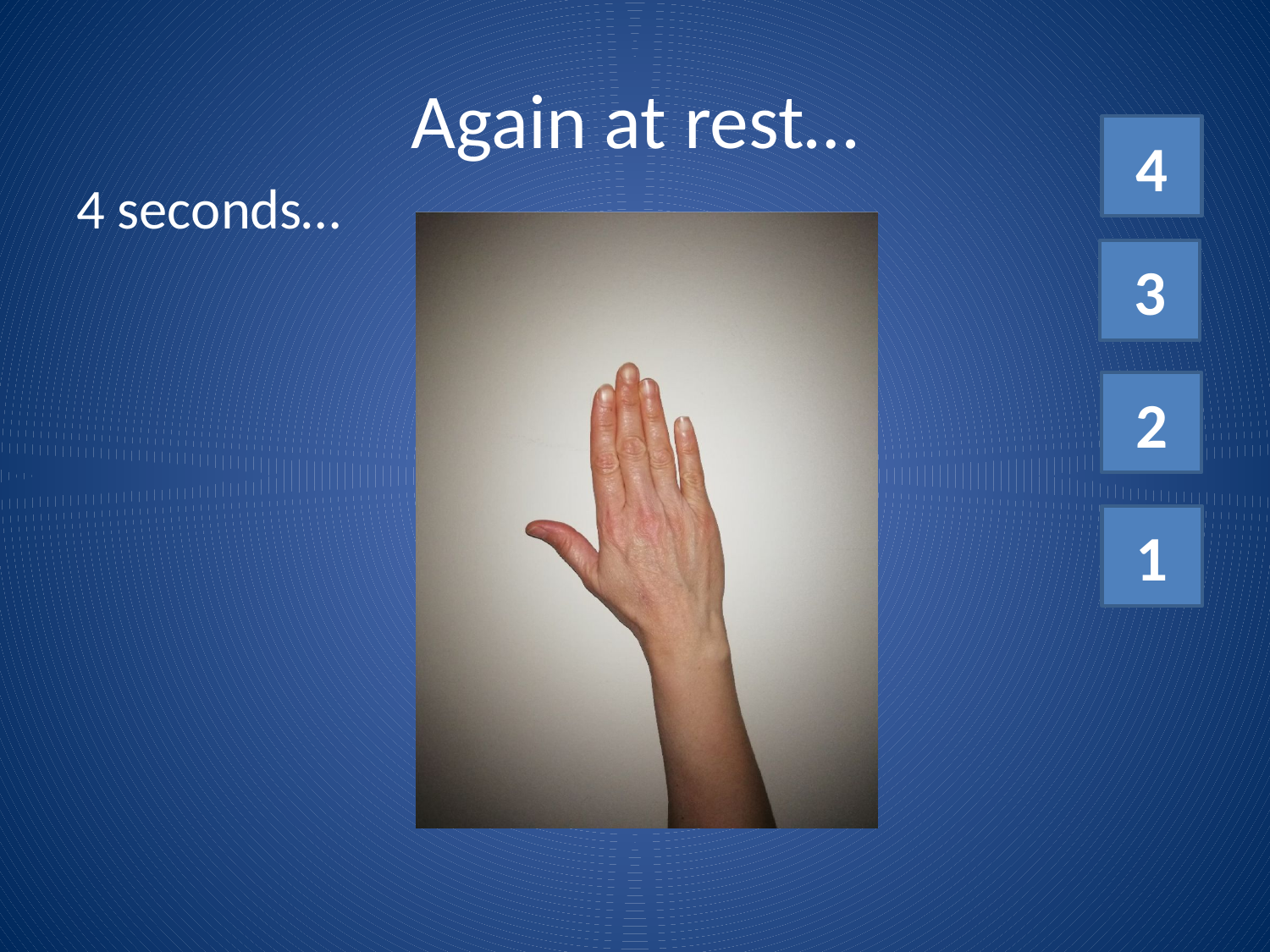

# Again at rest…
4
4 seconds…
3
2
1

## Slide 55
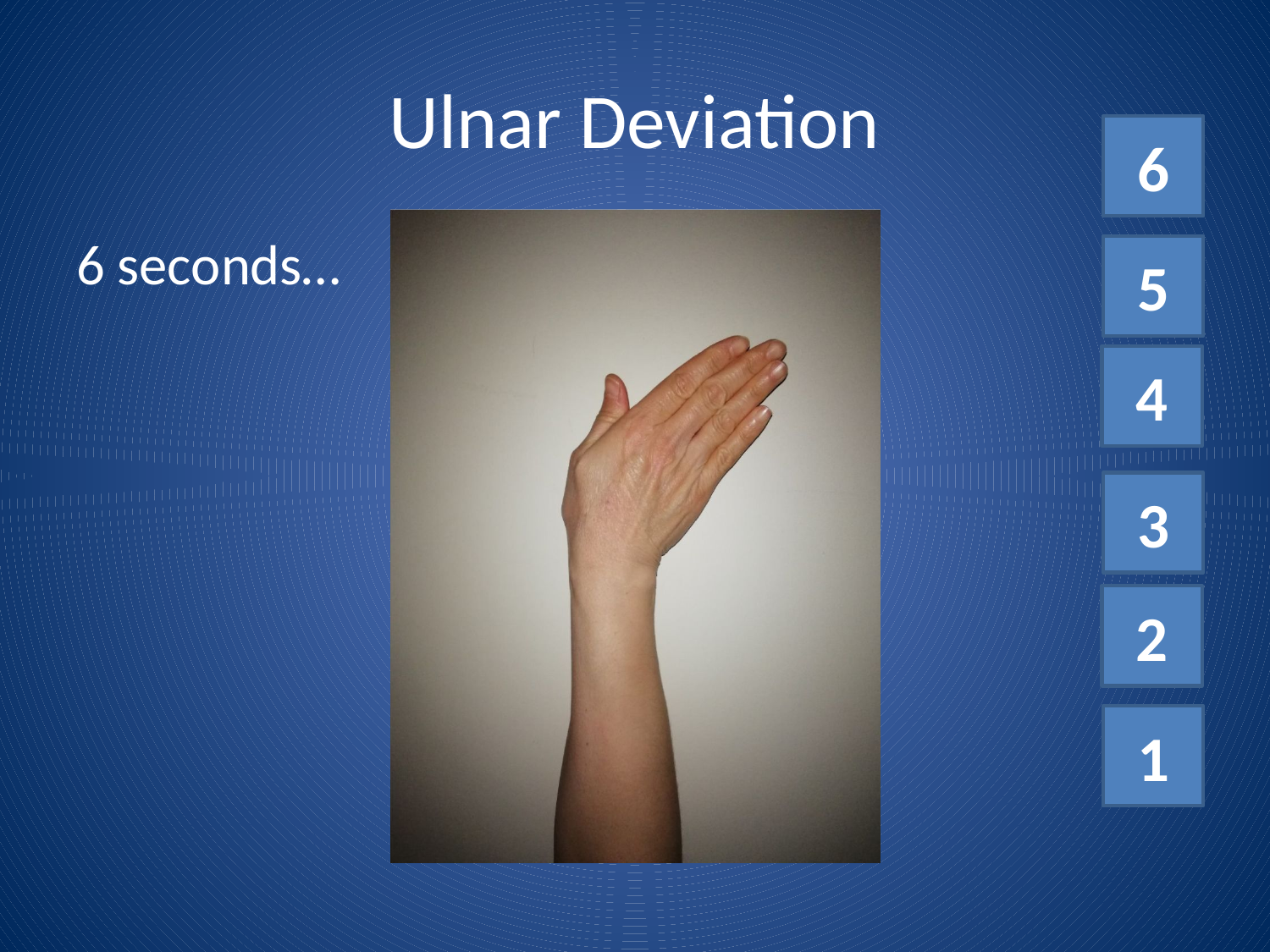

# Ulnar Deviation
6
6 seconds…
5
4
3
2
1

## Slide 56
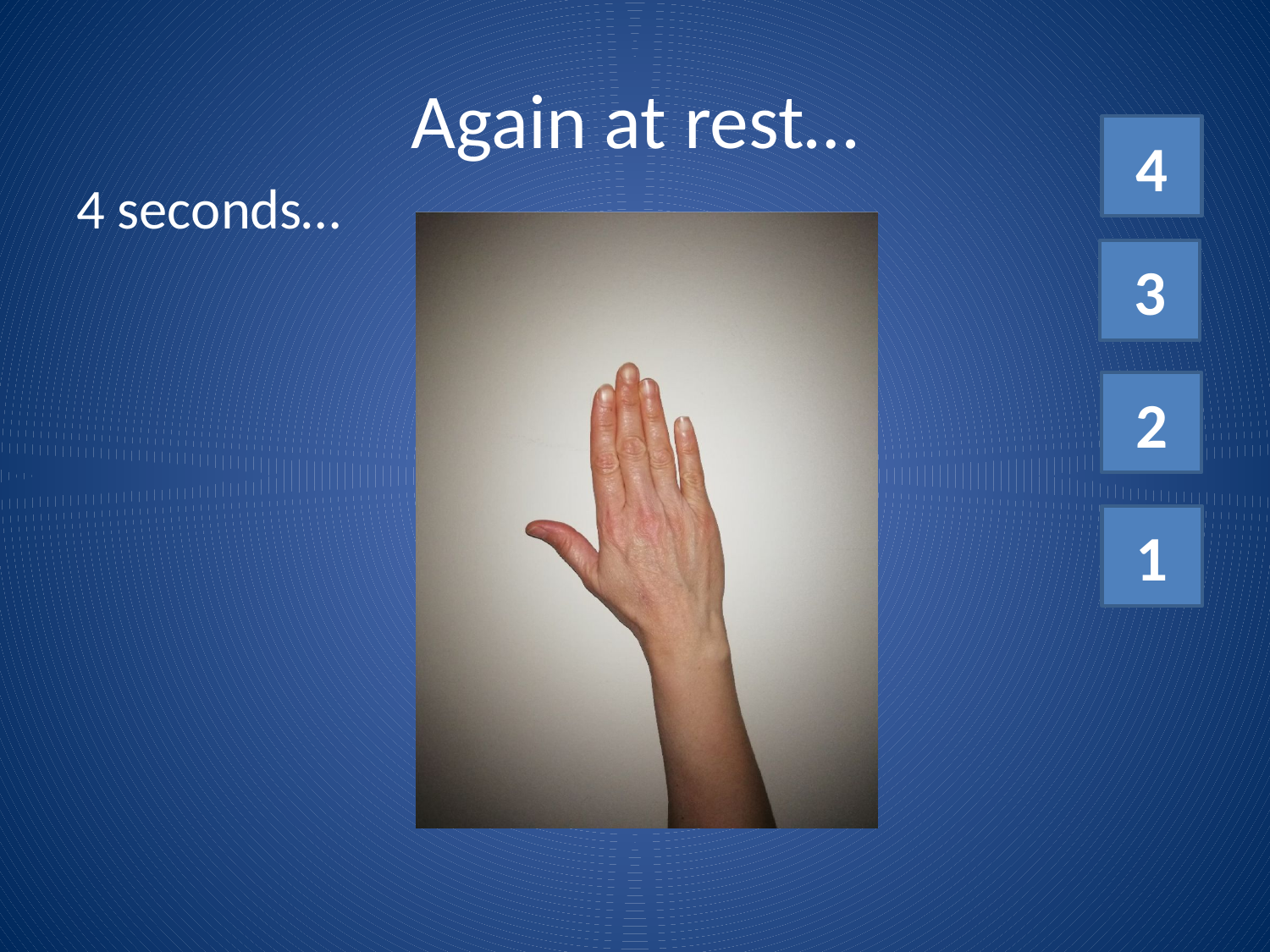

# Again at rest…
4
4 seconds…
3
2
1

## Slide 57
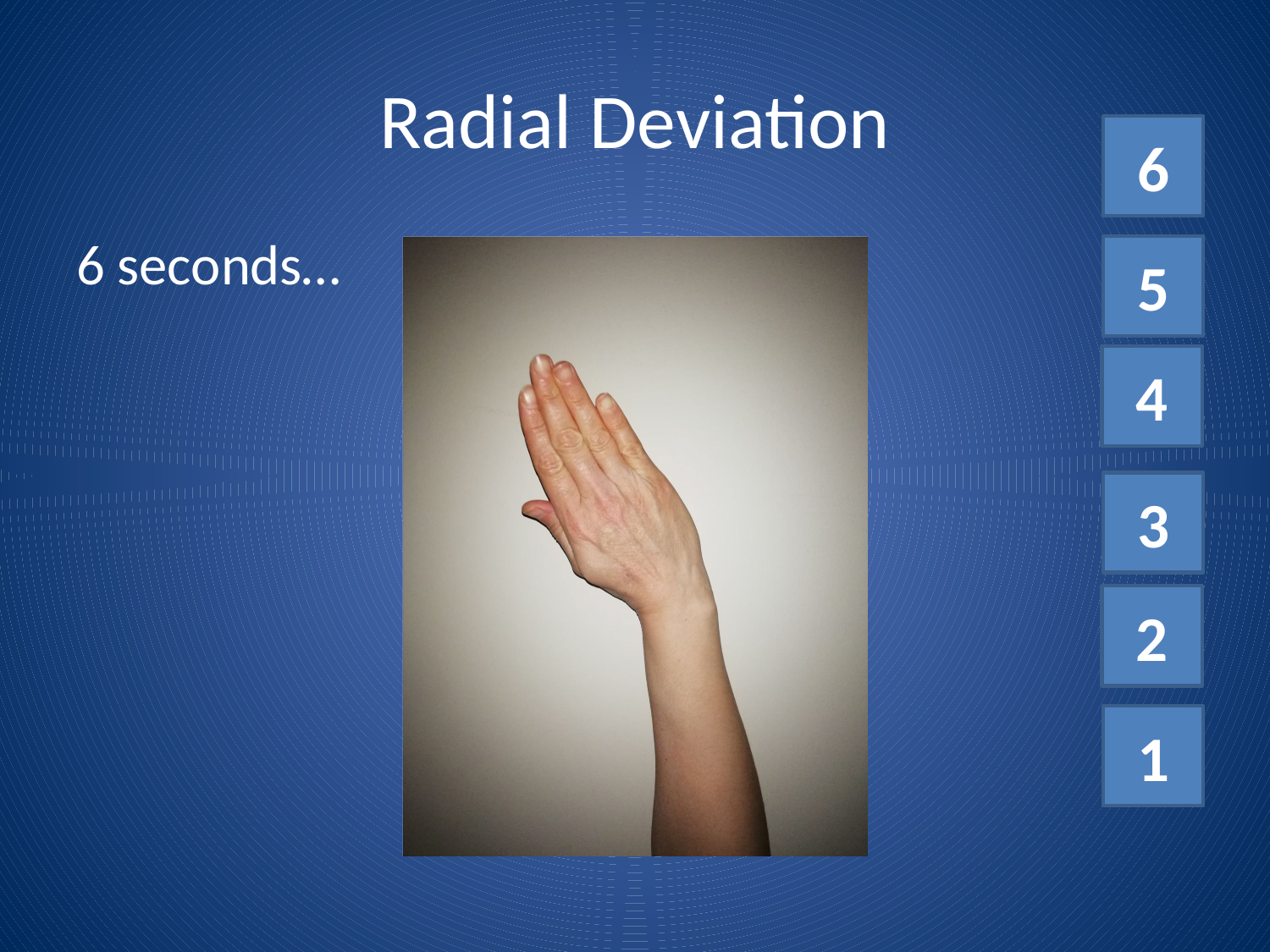

# Radial Deviation
6
6 seconds…
5
4
3
2
1

## Slide 58
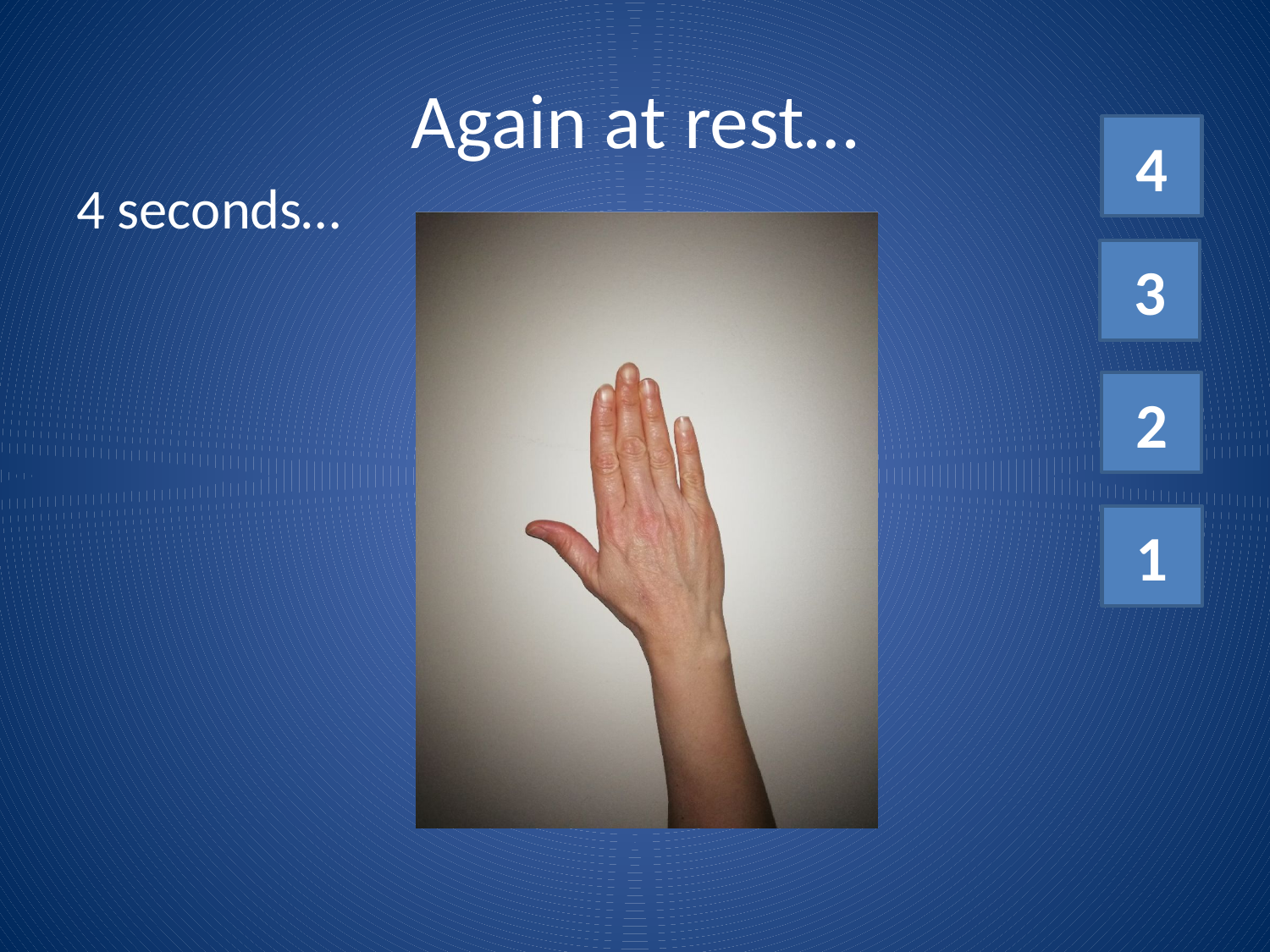

# Again at rest…
4
4 seconds…
3
2
1

## Slide 59
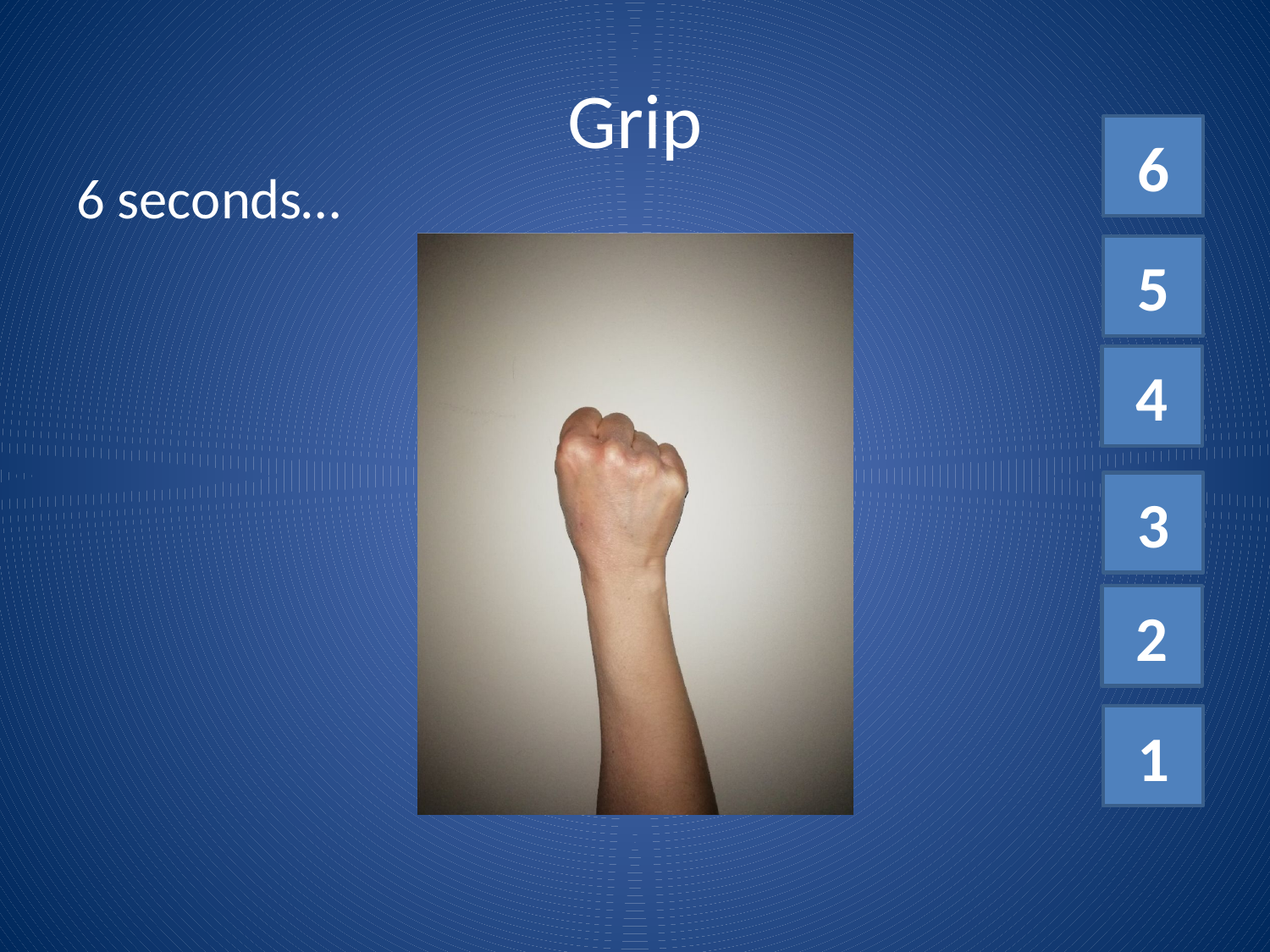

# Grip
6
6 seconds…
5
4
3
2
1

## Slide 60
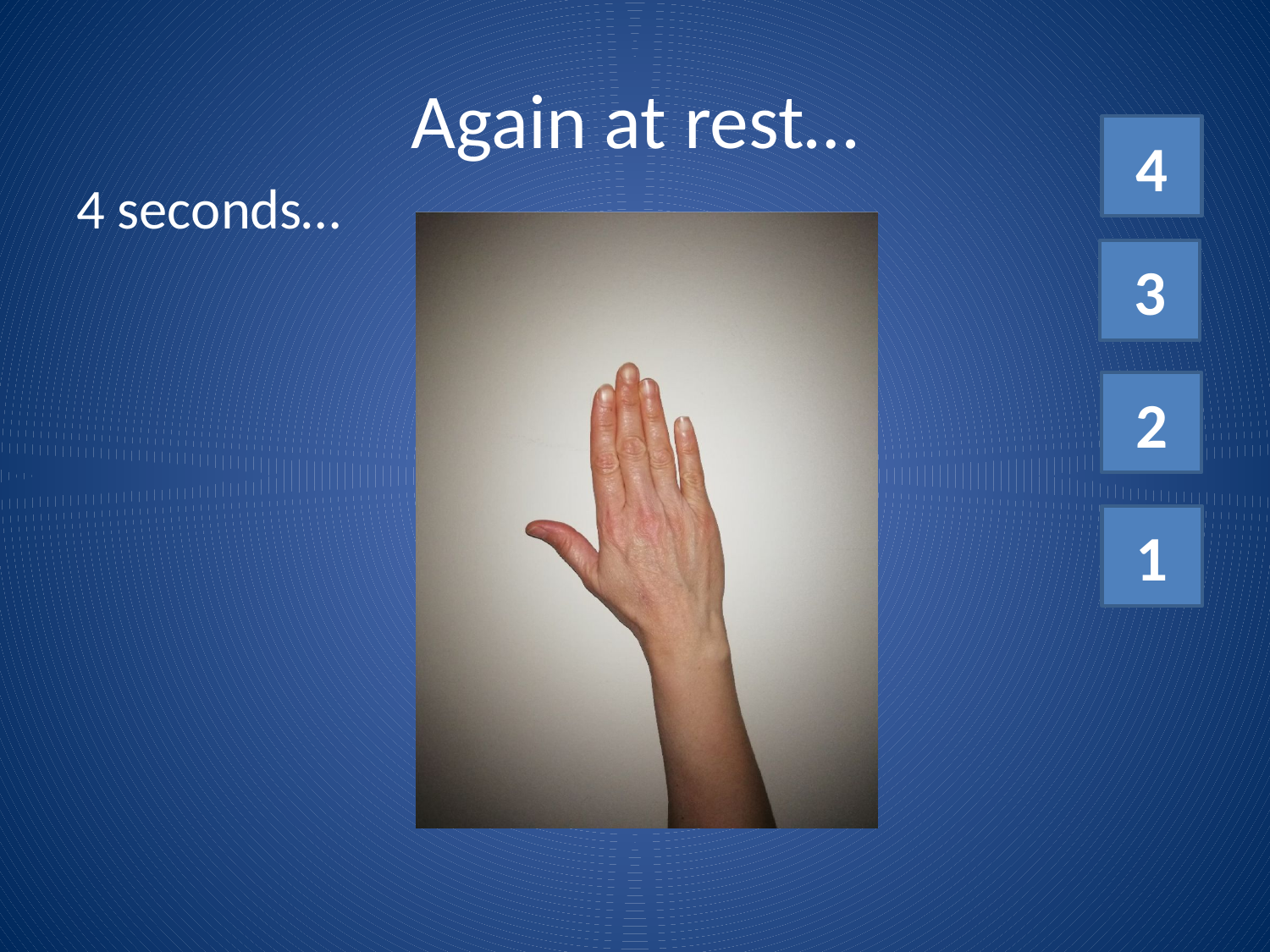

# Again at rest…
4
4 seconds…
3
2
1

## Slide 61
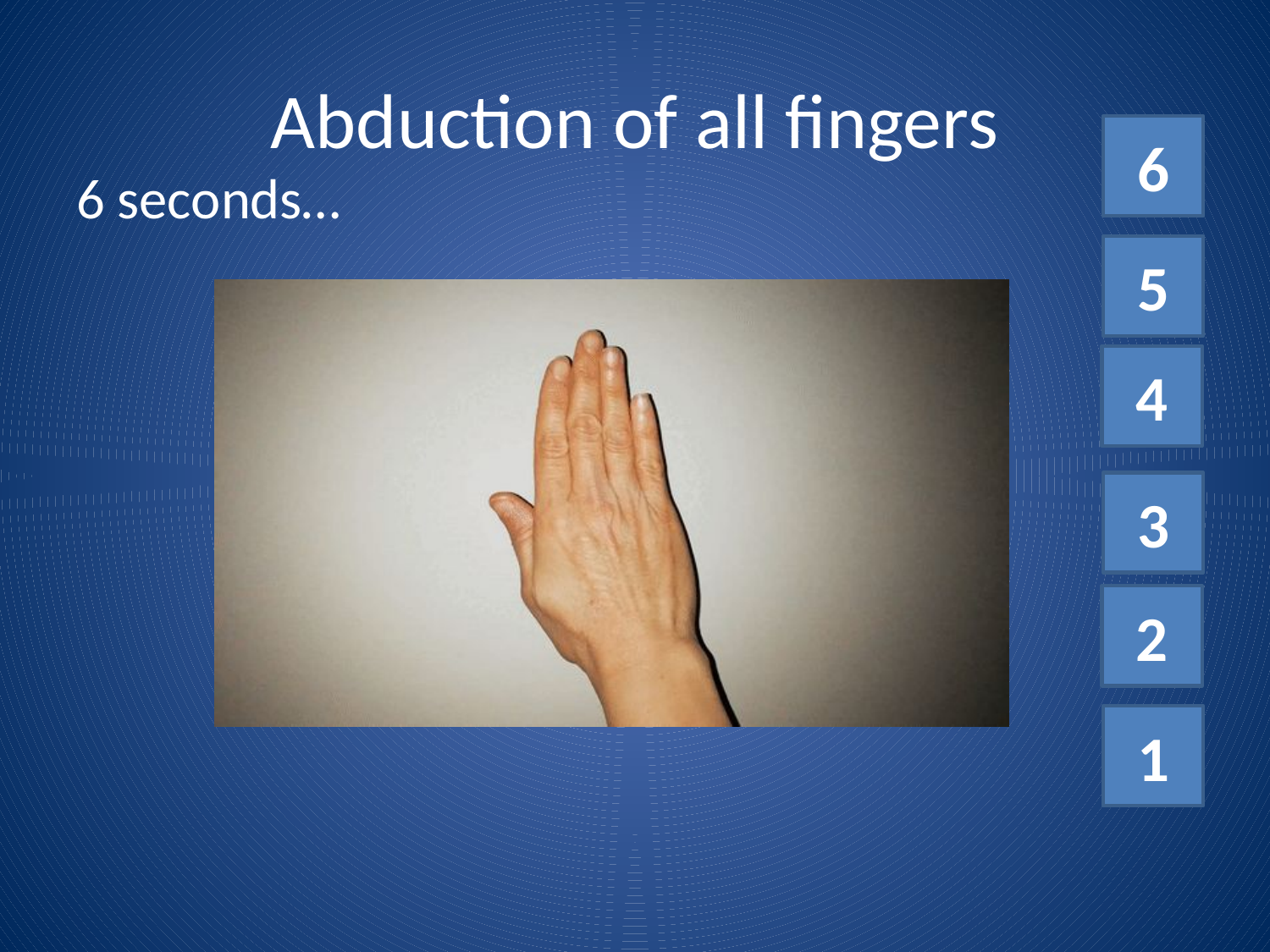

# Abduction of all fingers
6
6 seconds…
5
4
3
2
1

## Slide 62
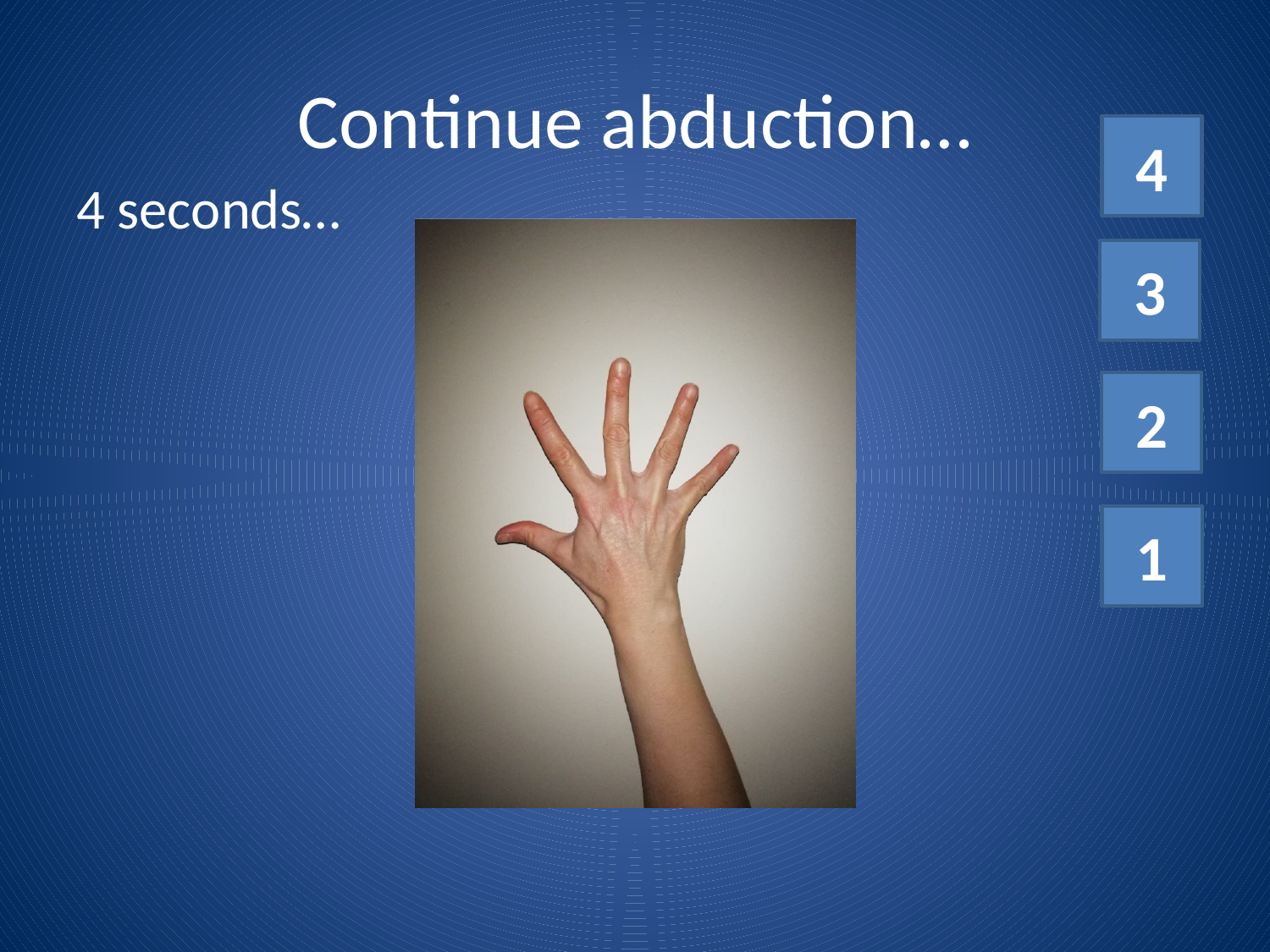

# Continue abduction…
4
4 seconds…
3
2
1

## Slide 63
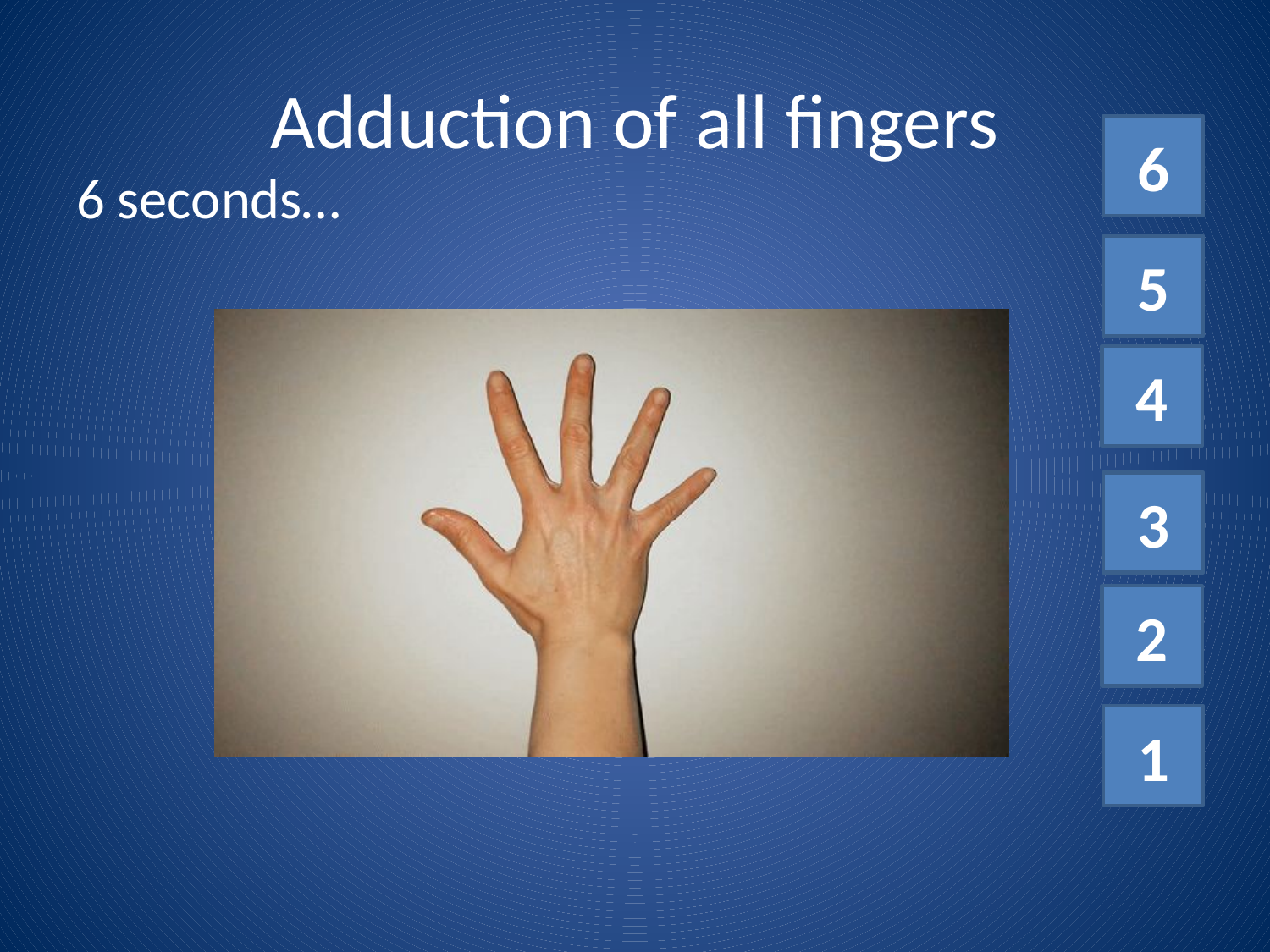

# Adduction of all fingers
6
6 seconds…
5
4
3
2
1

## Slide 64
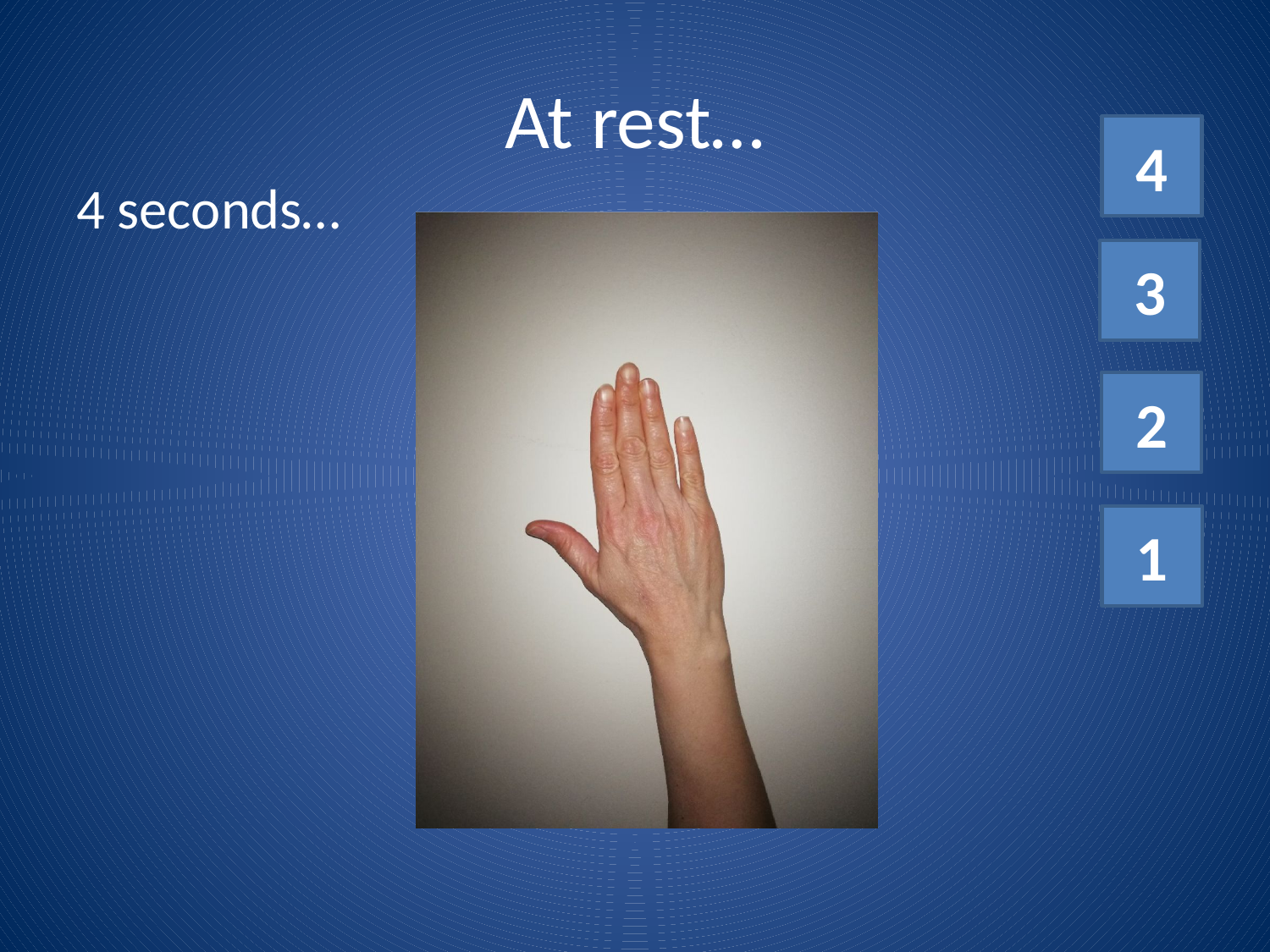

# At rest…
4
4 seconds…
3
2
1

## Slide 65
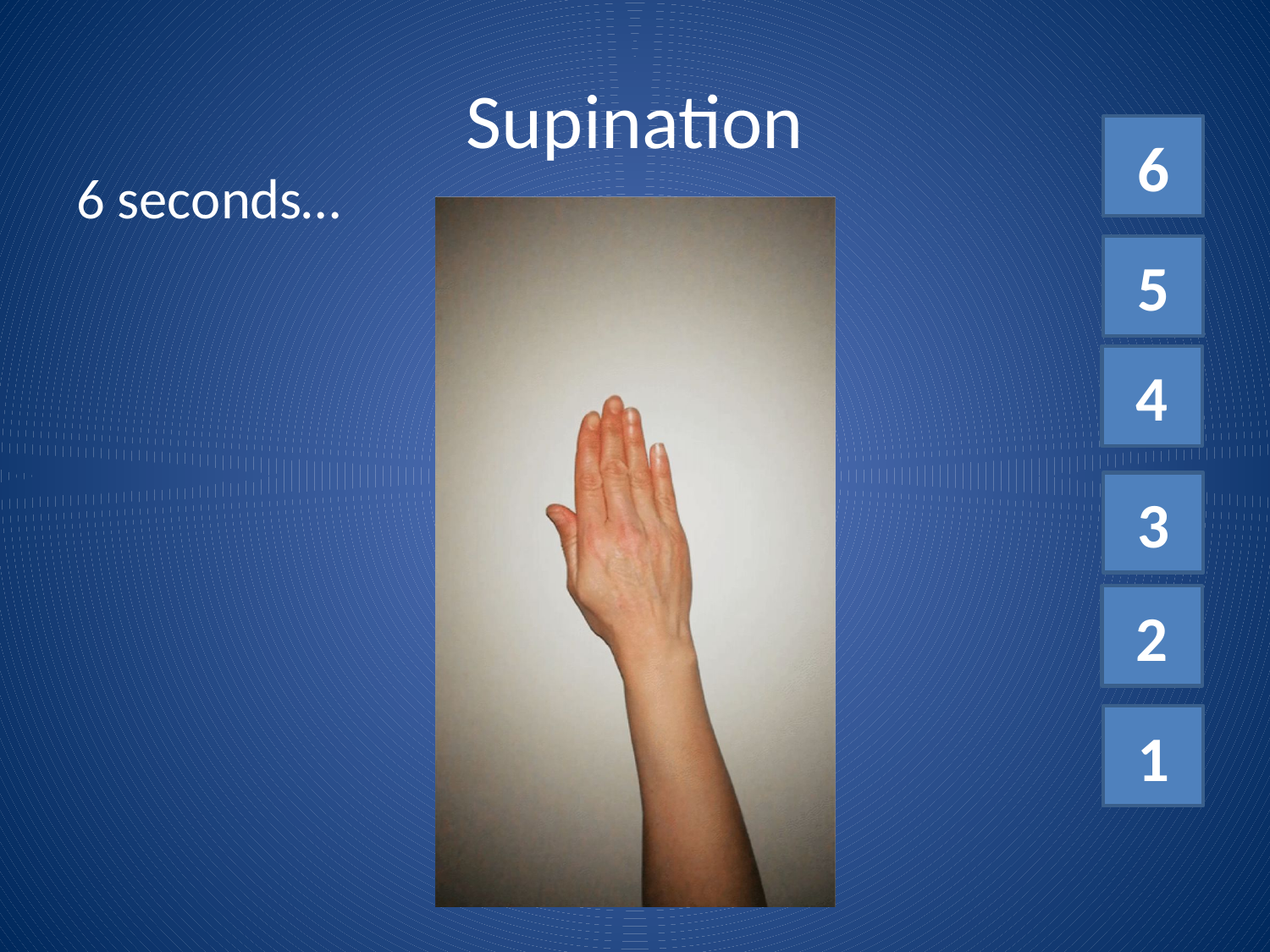

# Supination
6
6 seconds…
5
4
3
2
1

## Slide 66
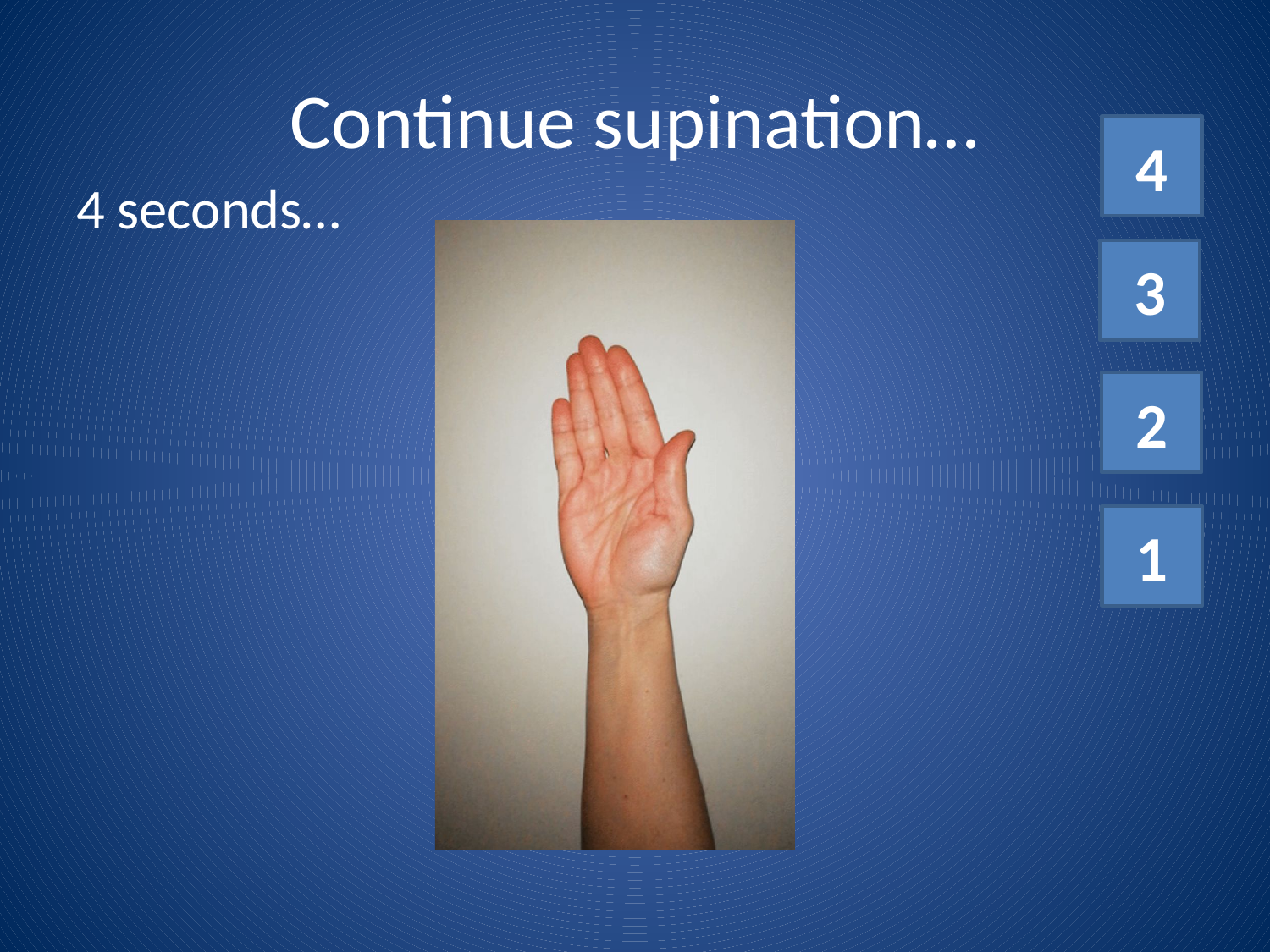

# Continue supination…
4
4 seconds…
3
2
1

## Slide 67
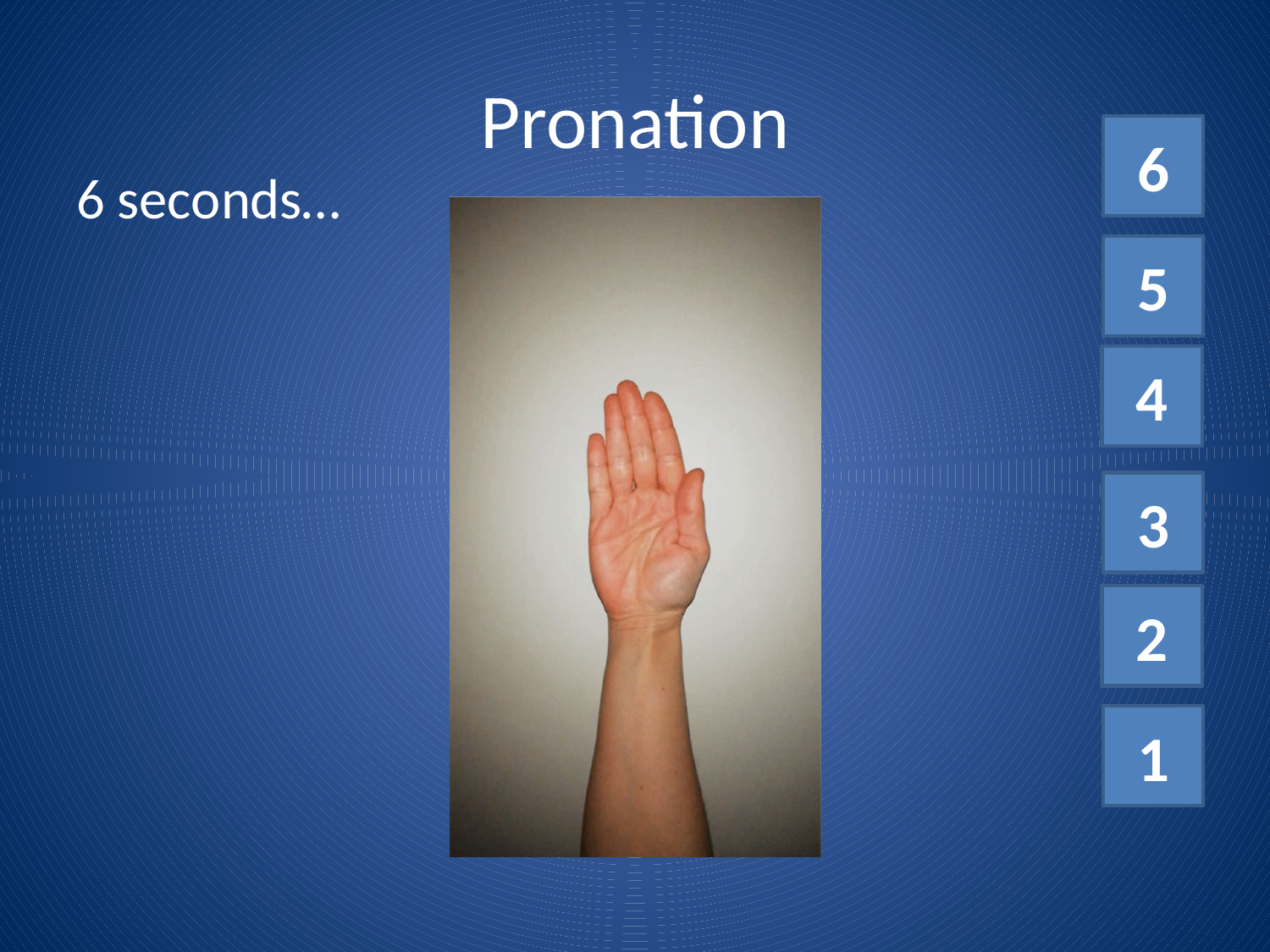

# Pronation
6
6 seconds…
5
4
3
2
1

## Slide 68
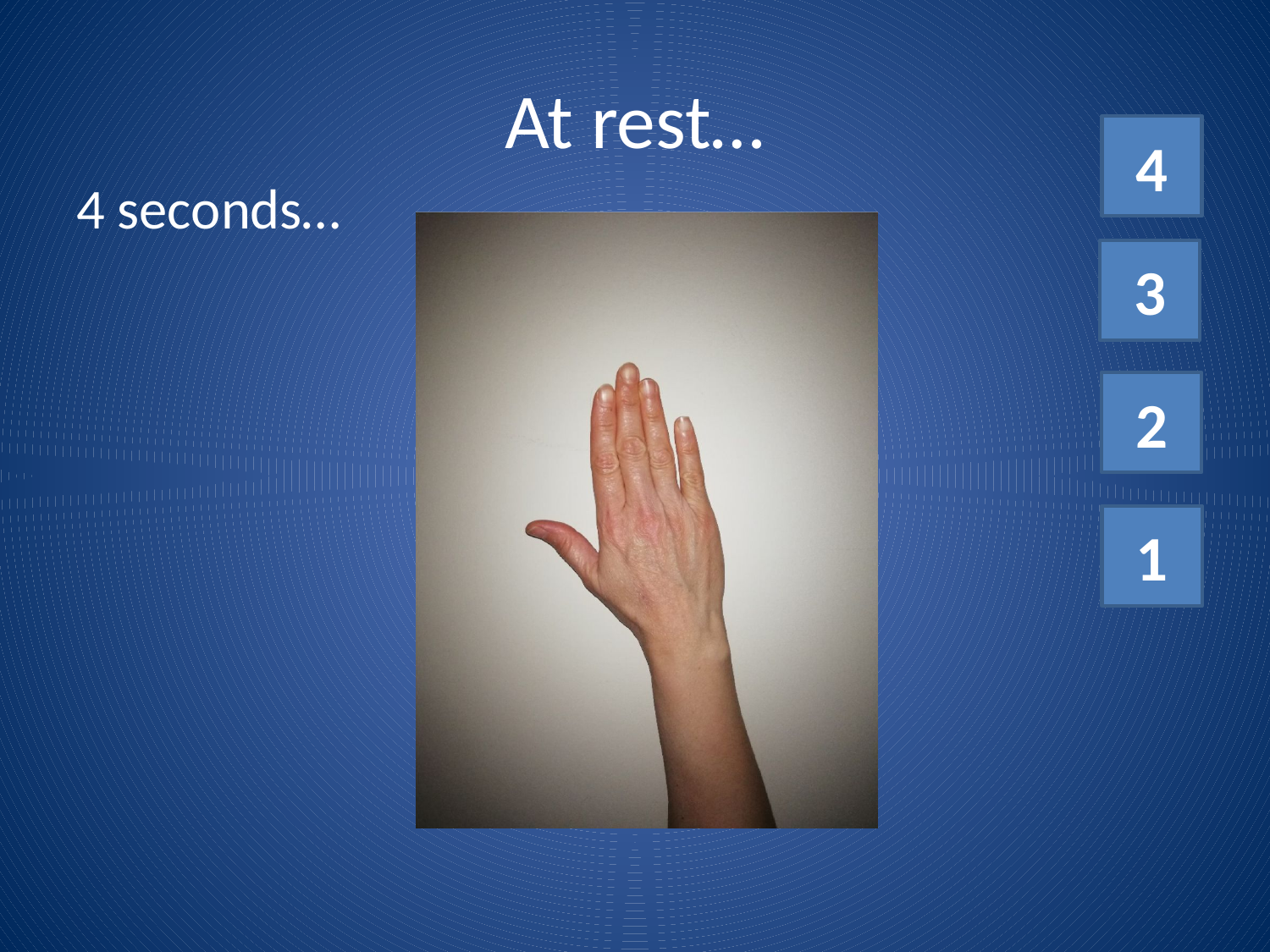

# At rest…
4
4 seconds…
3
2
1

## Slide 69
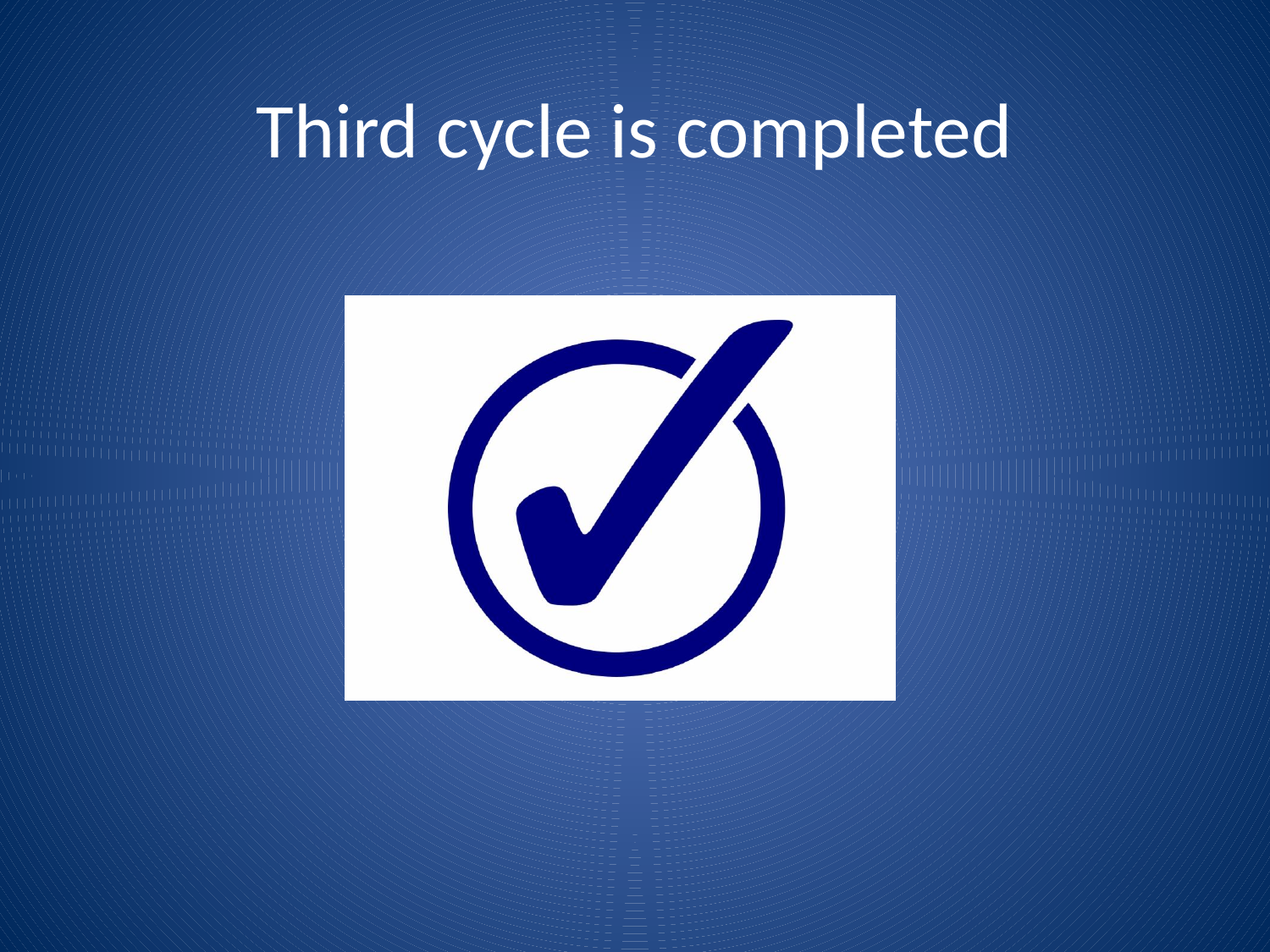

# Third cycle is completed

## Slide 70
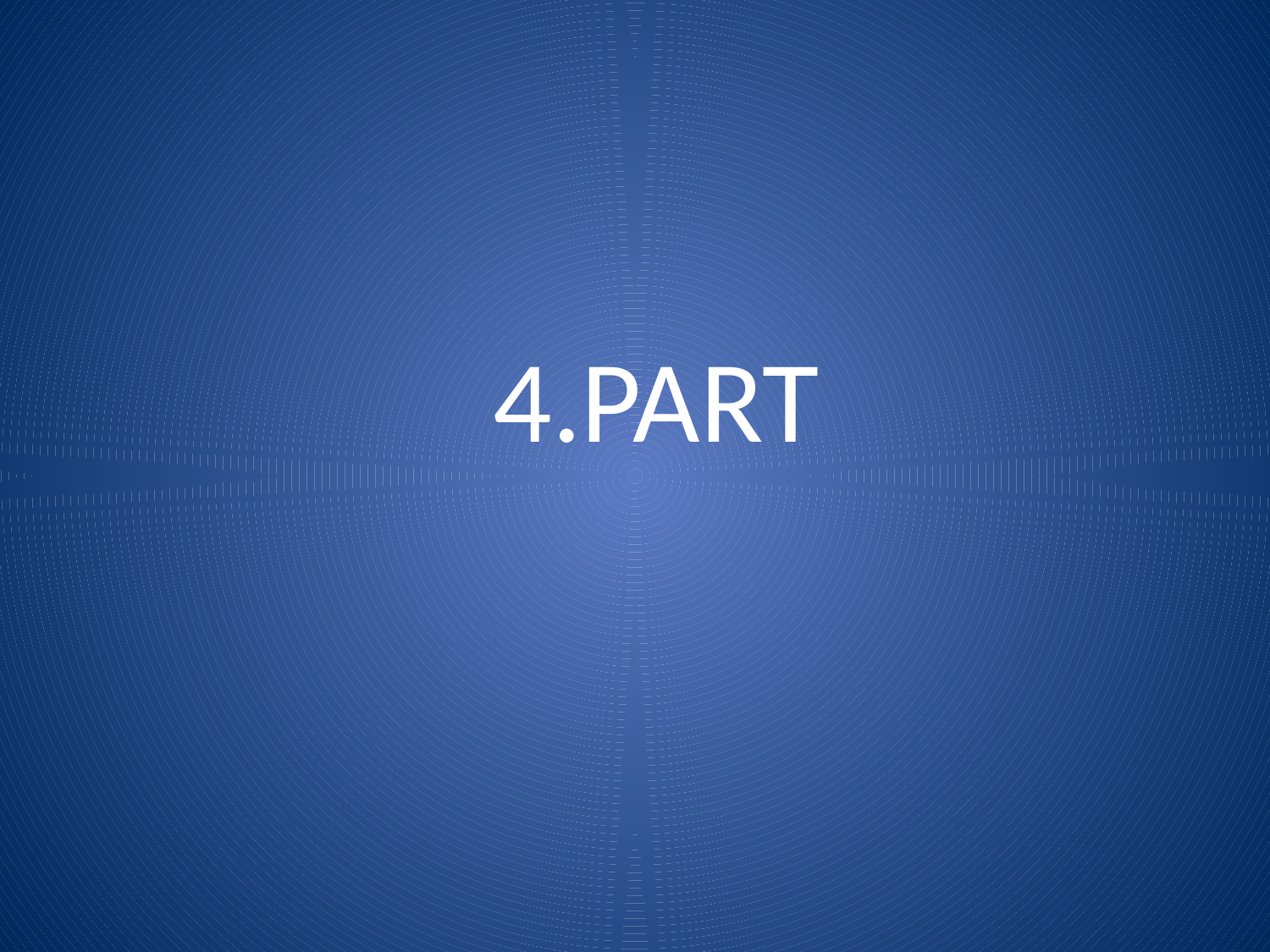

# 4.PART

## Slide 71
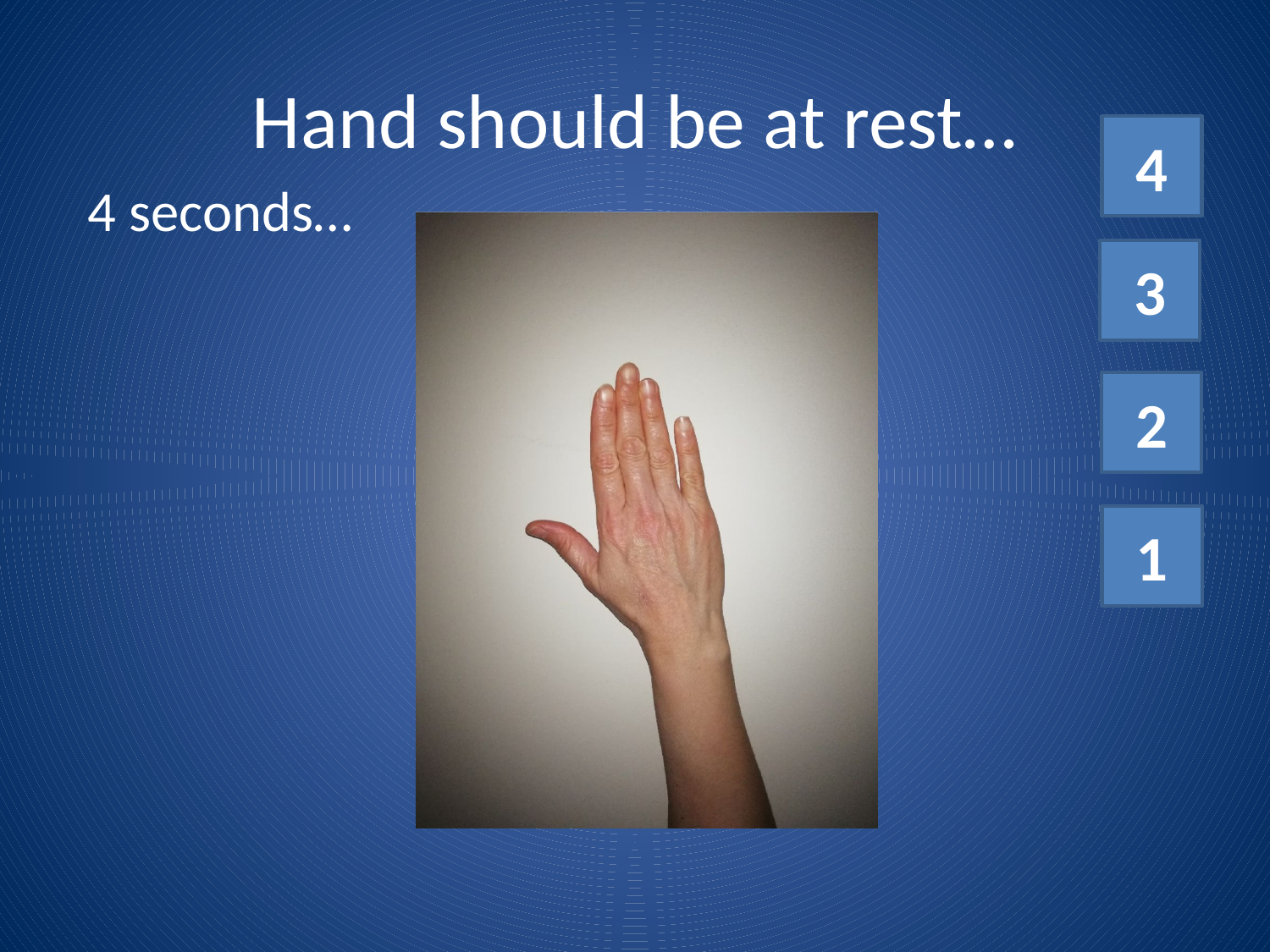

# Hand should be at rest…
4
4 seconds…
3
2
1

## Slide 72
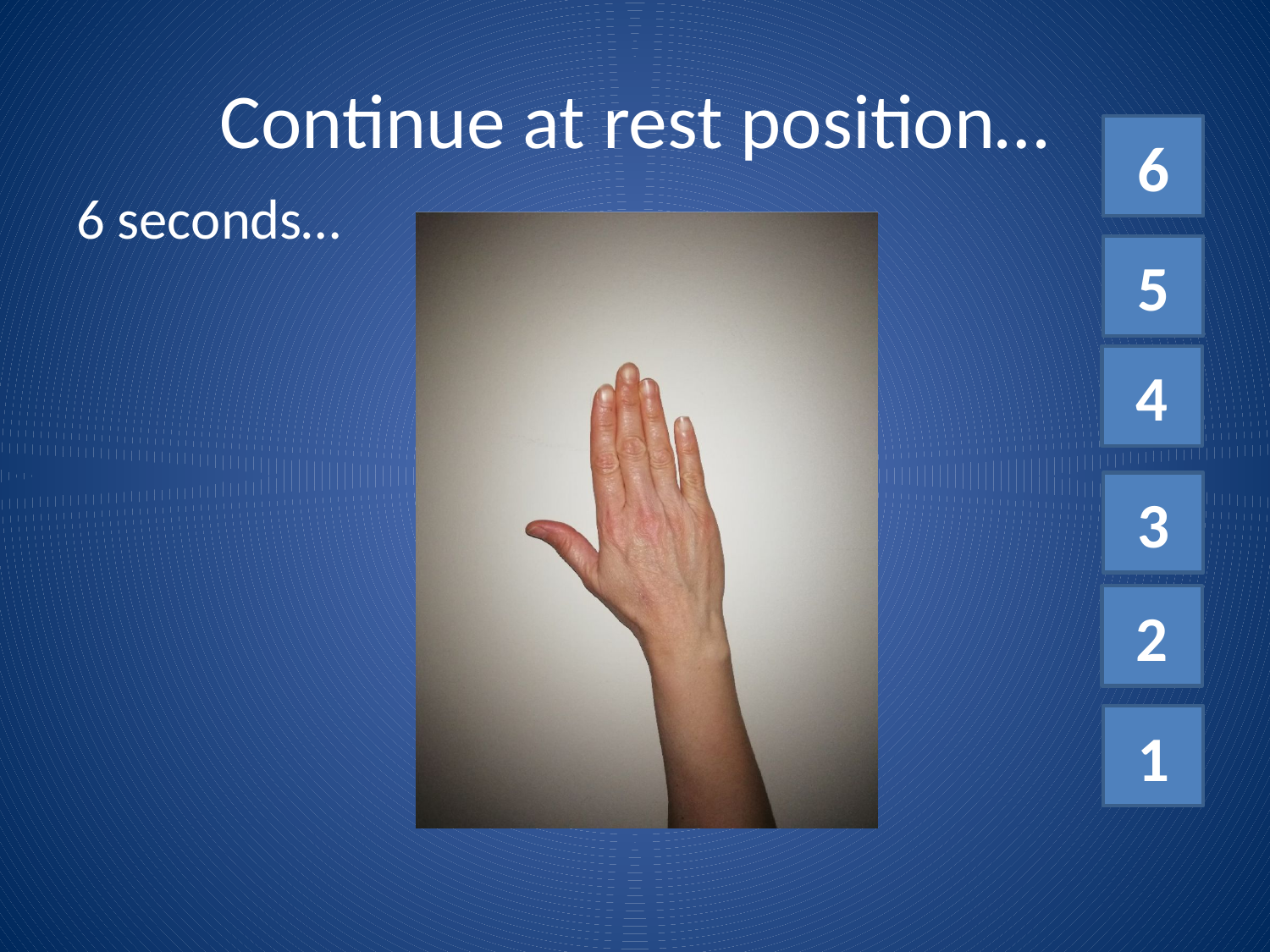

# Continue at rest position…
6
6 seconds…
5
4
3
2
1

## Slide 73
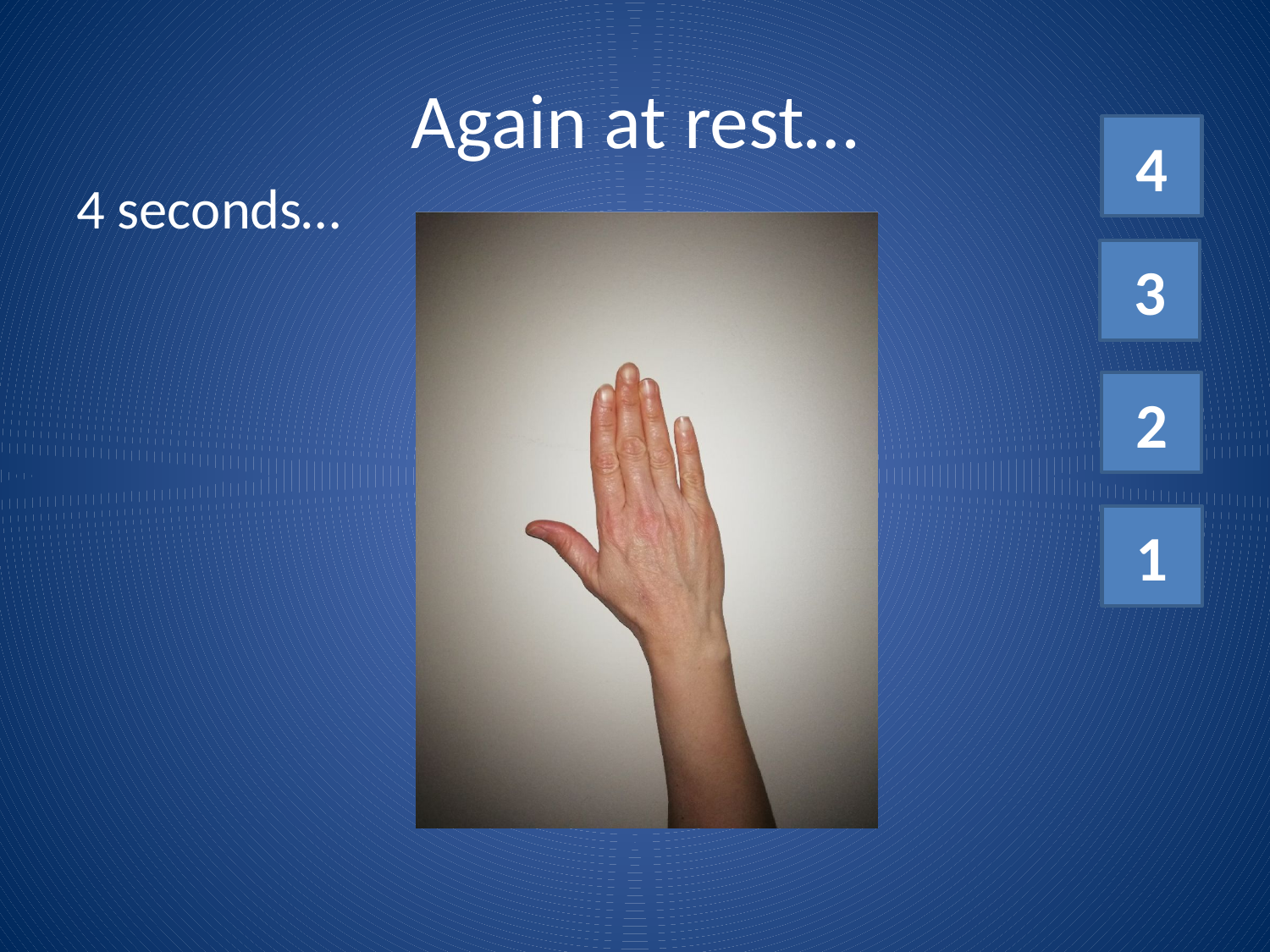

# Again at rest…
4
4 seconds…
3
2
1

## Slide 74
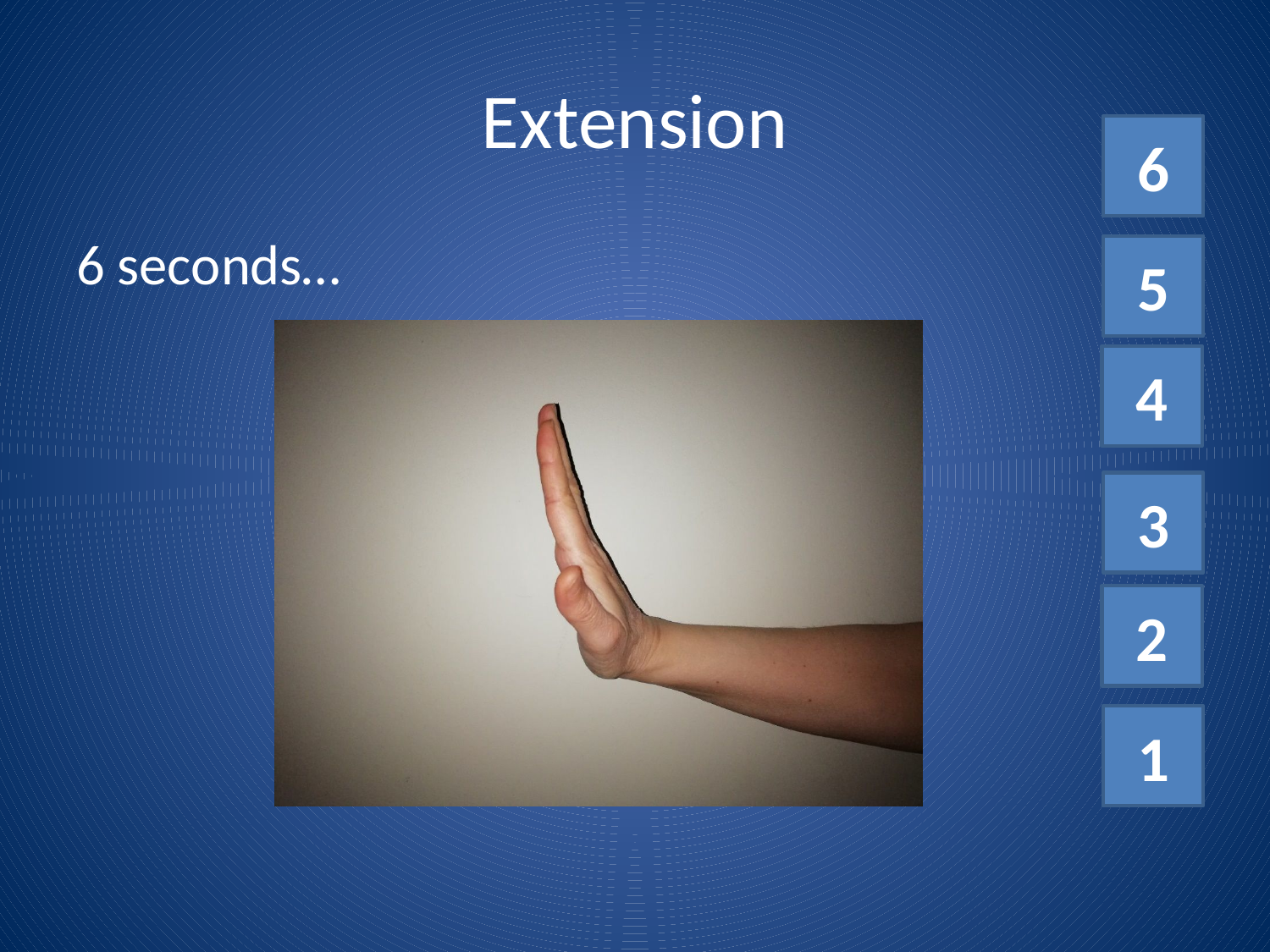

# Extension
6
6 seconds…
5
4
3
2
1

## Slide 75
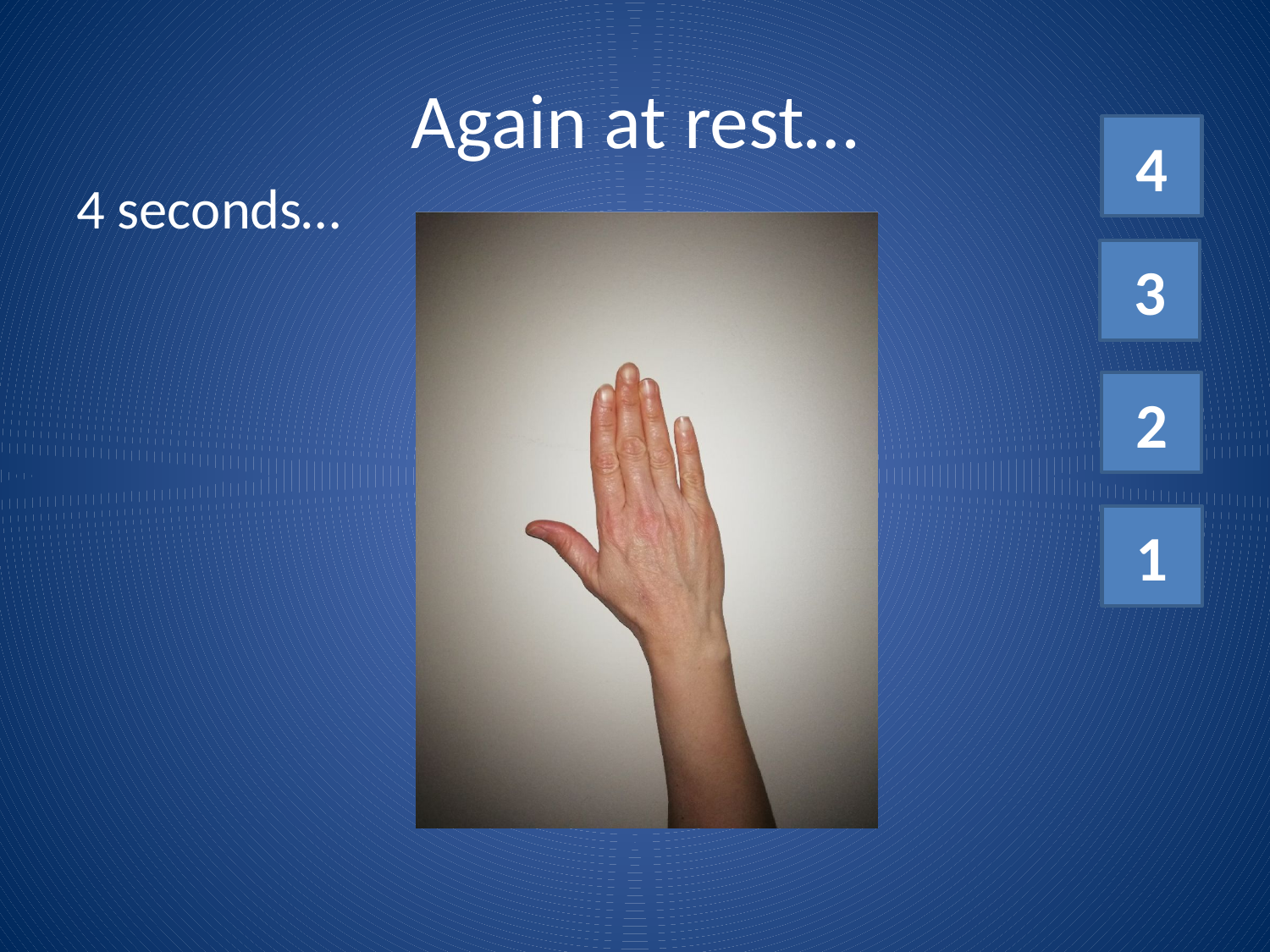

# Again at rest…
4
4 seconds…
3
2
1

## Slide 76
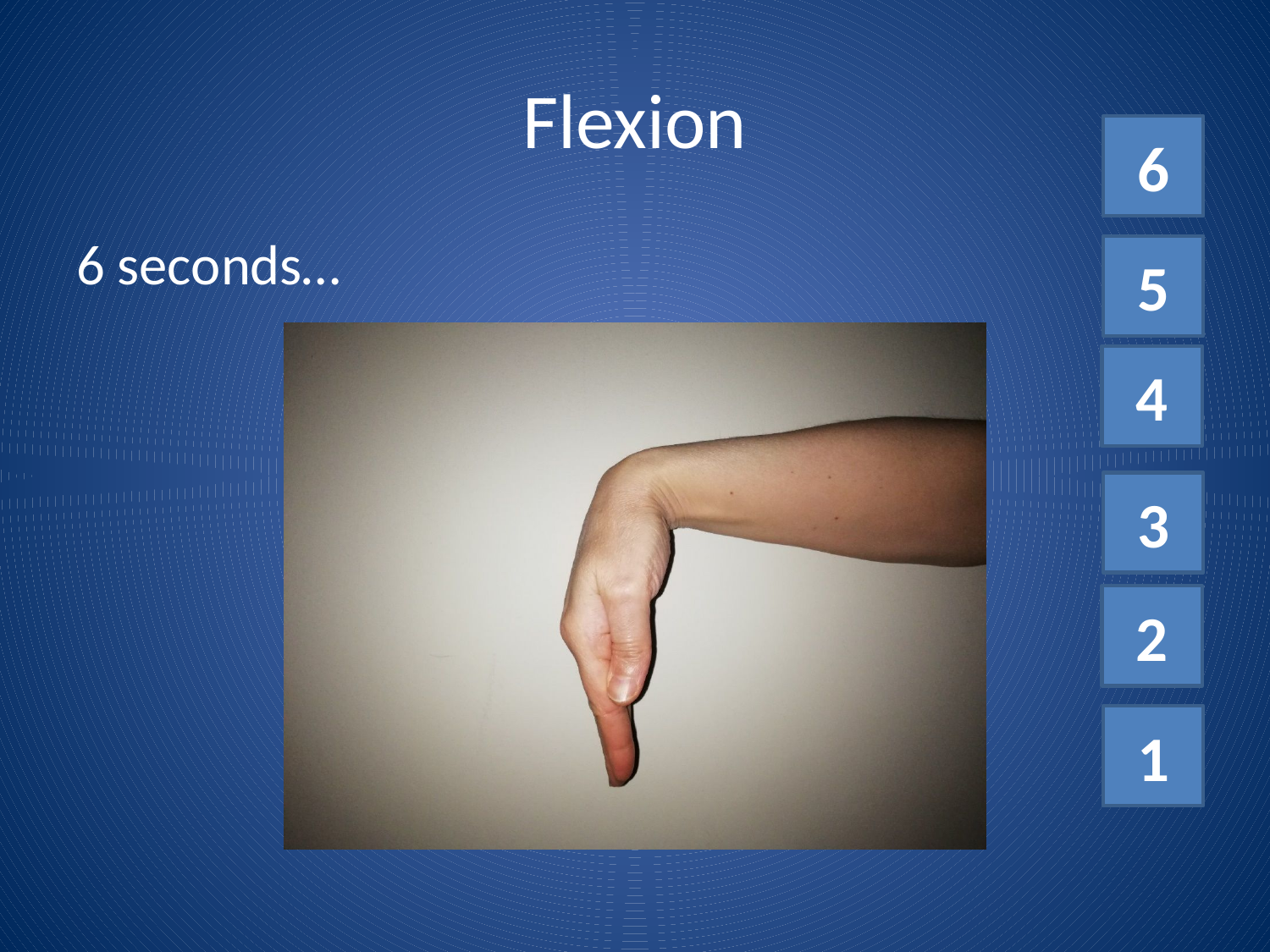

# Flexion
6
6 seconds…
5
4
3
2
1

## Slide 77
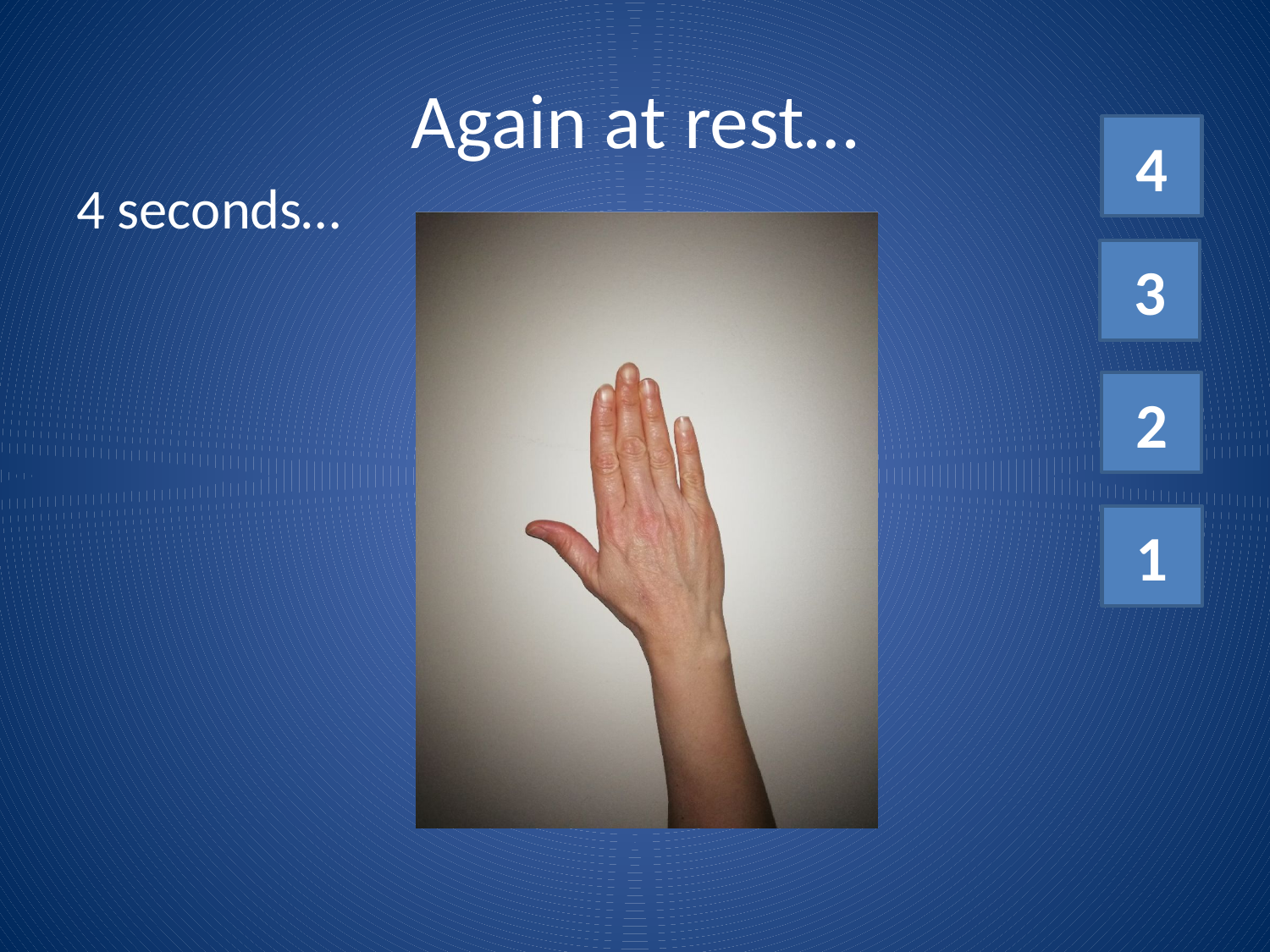

# Again at rest…
4
4 seconds…
3
2
1

## Slide 78
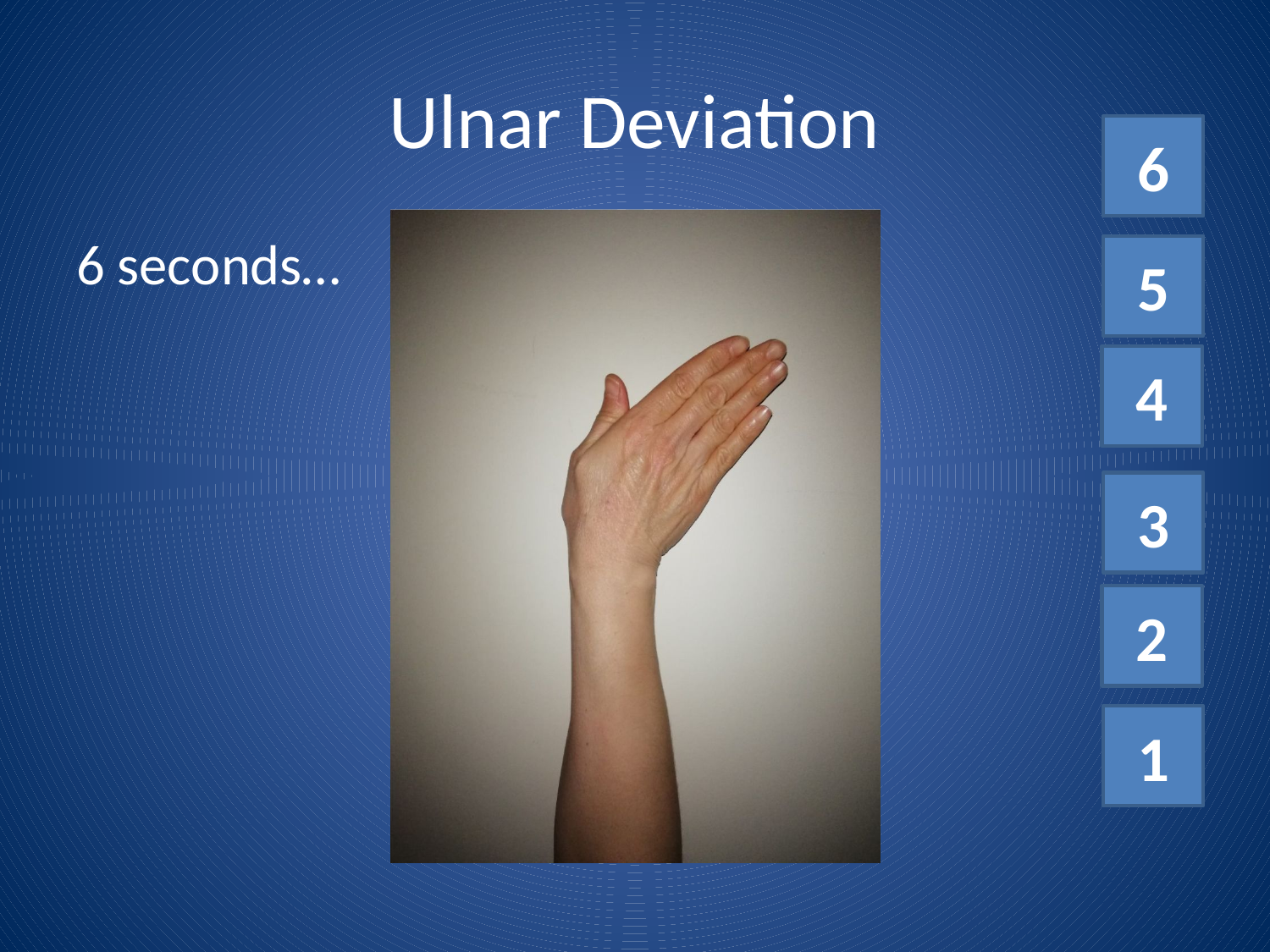

# Ulnar Deviation
6
6 seconds…
5
4
3
2
1

## Slide 79
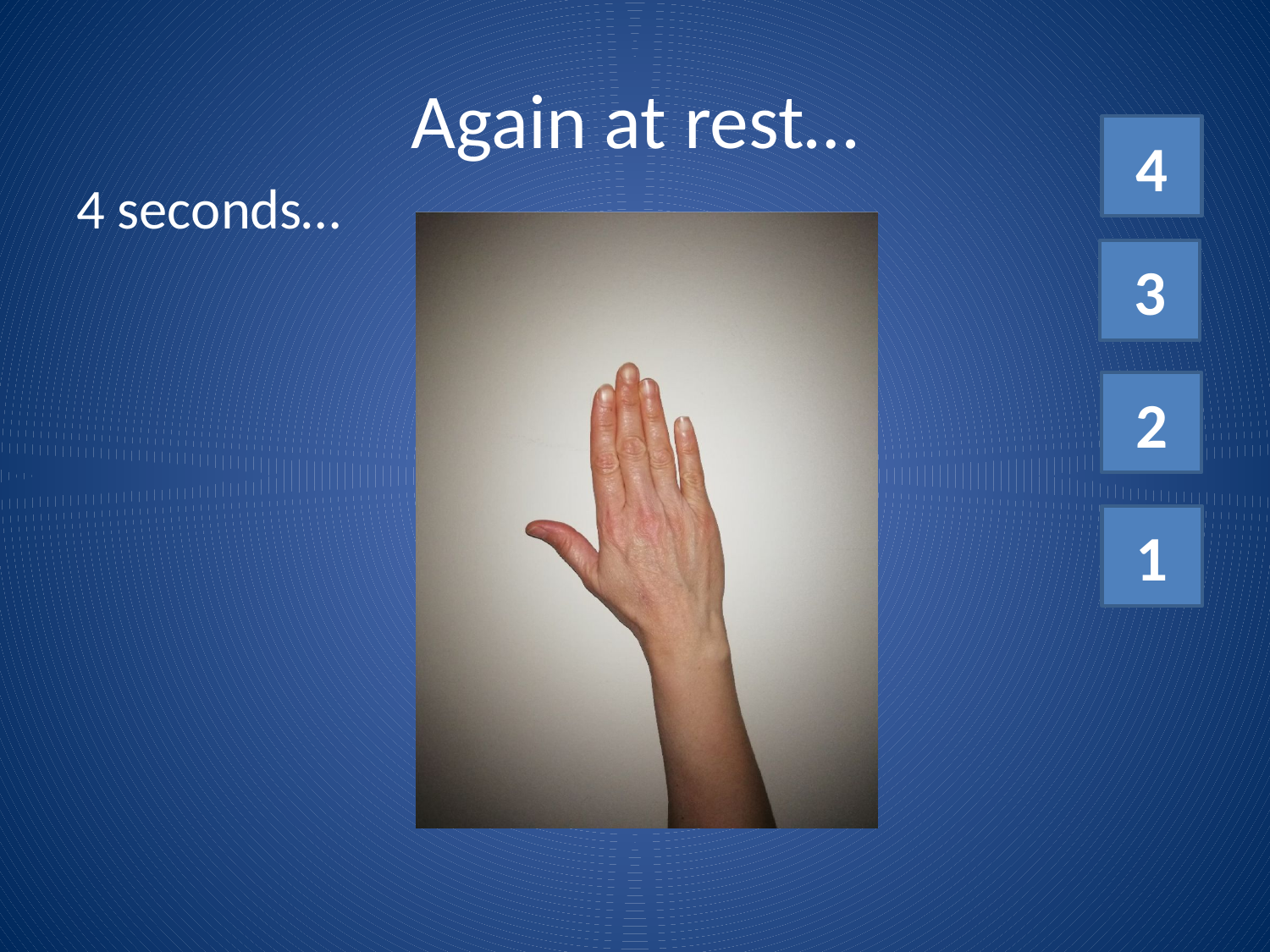

# Again at rest…
4
4 seconds…
3
2
1

## Slide 80
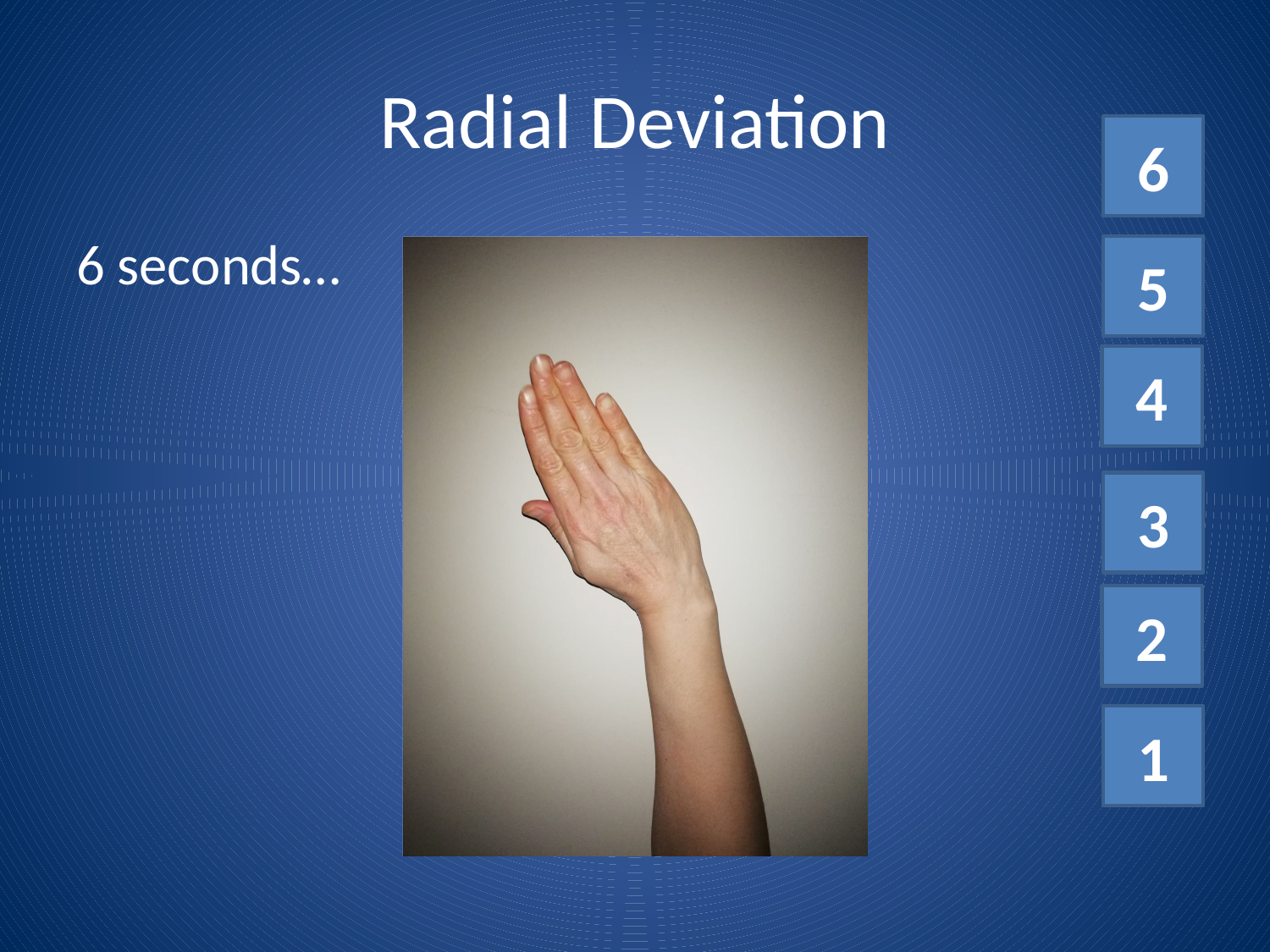

# Radial Deviation
6
6 seconds…
5
4
3
2
1

## Slide 81
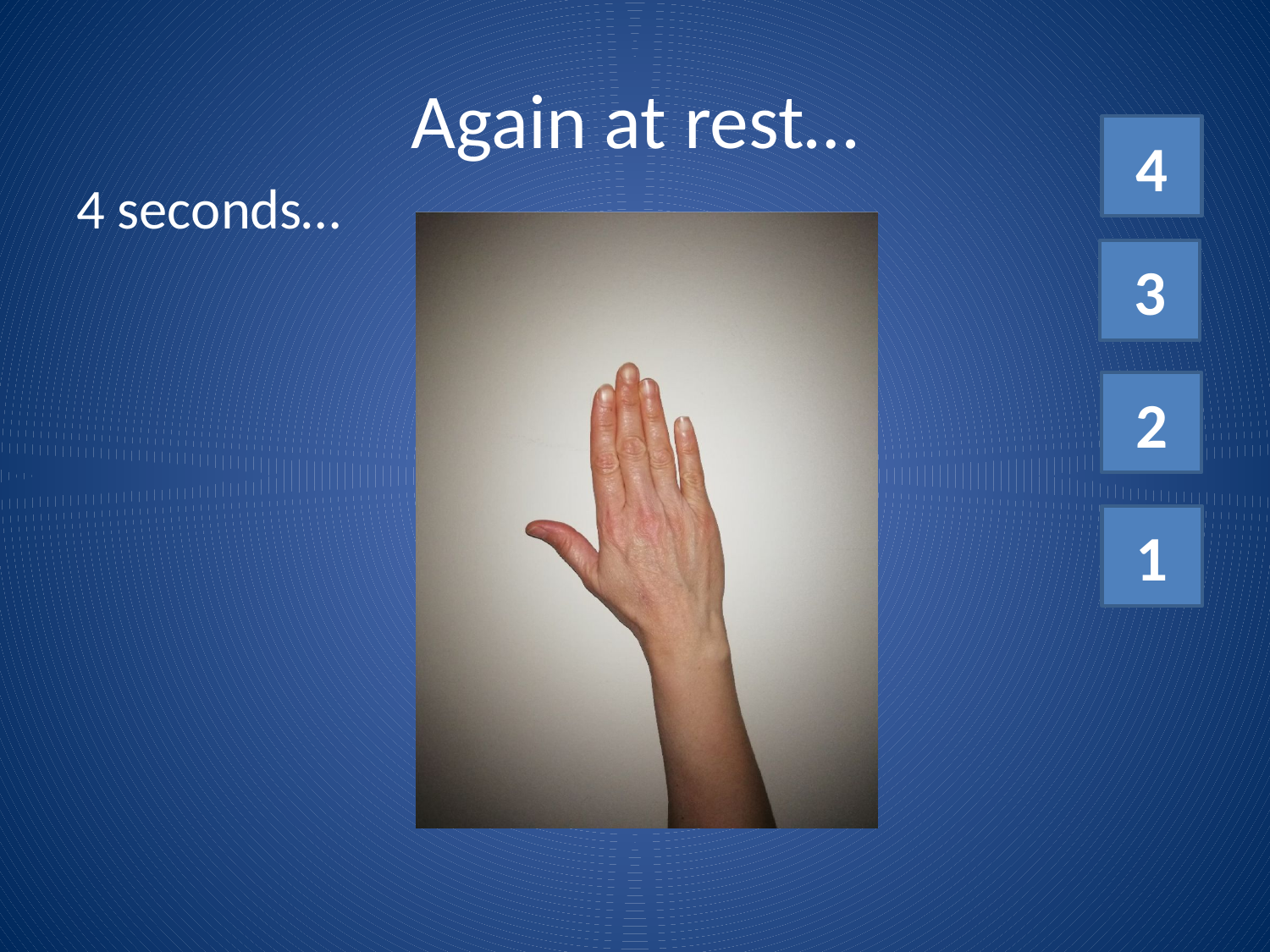

# Again at rest…
4
4 seconds…
3
2
1

## Slide 82
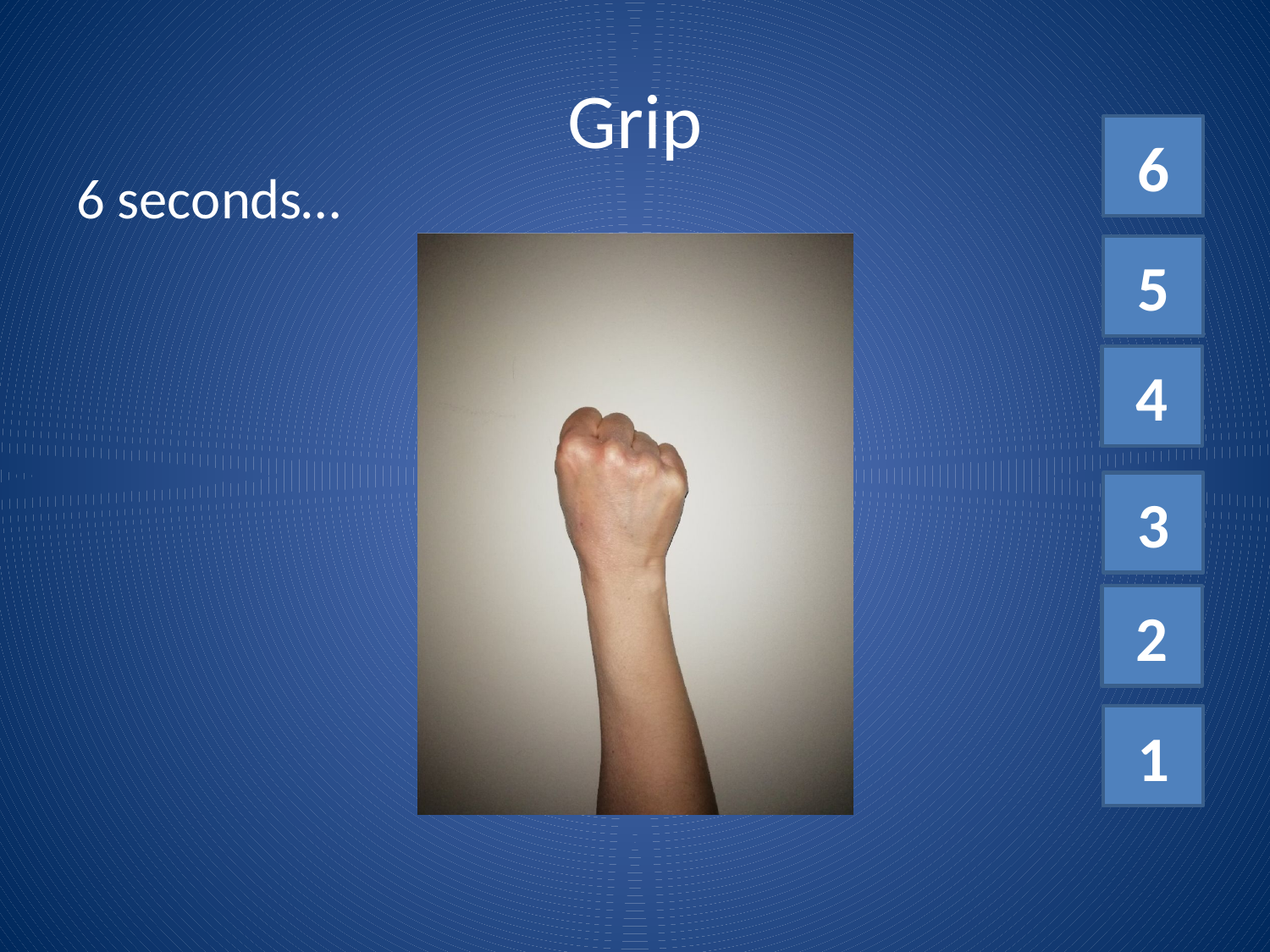

# Grip
6
6 seconds…
5
4
3
2
1

## Slide 83
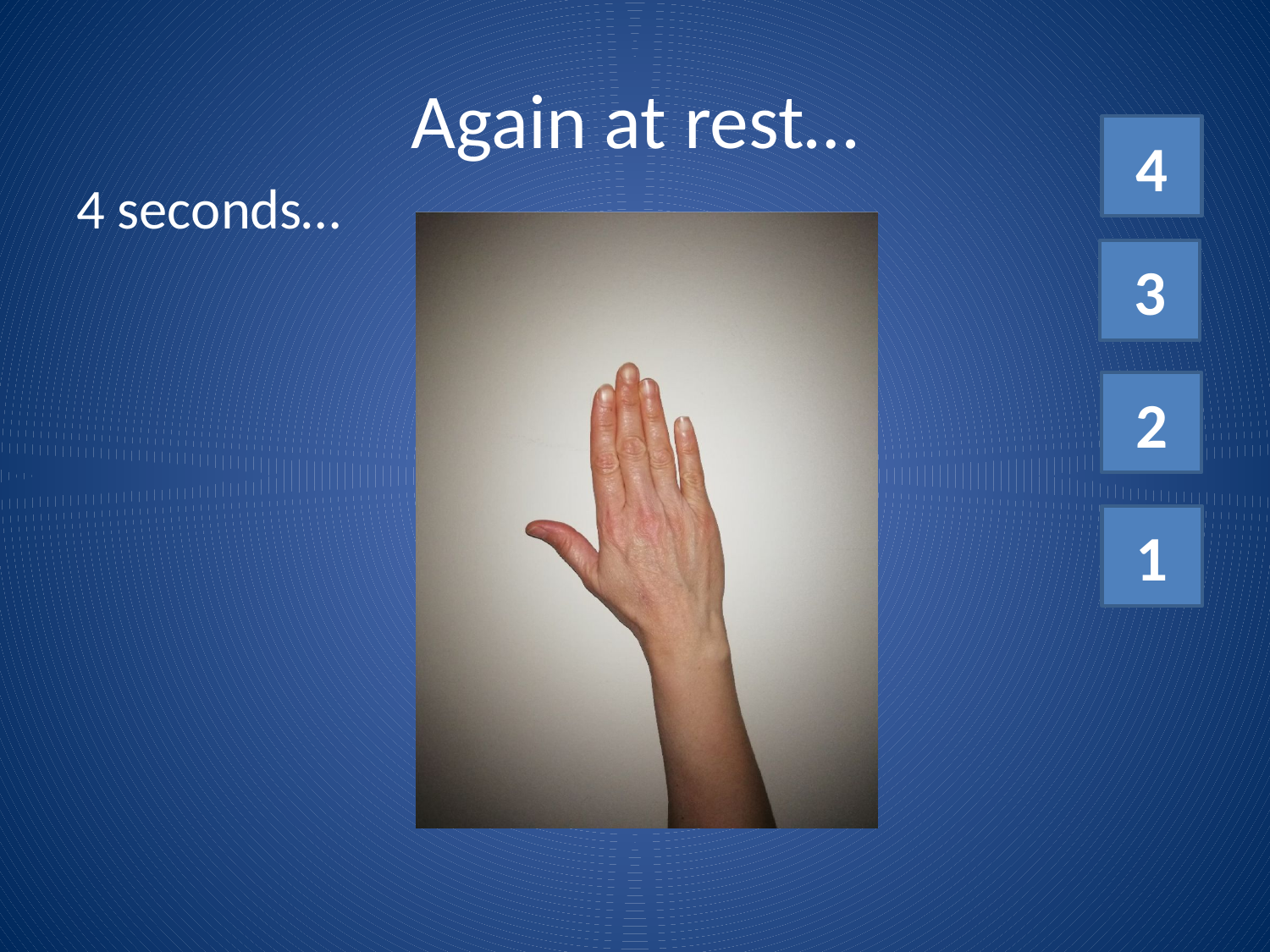

# Again at rest…
4
4 seconds…
3
2
1

## Slide 84
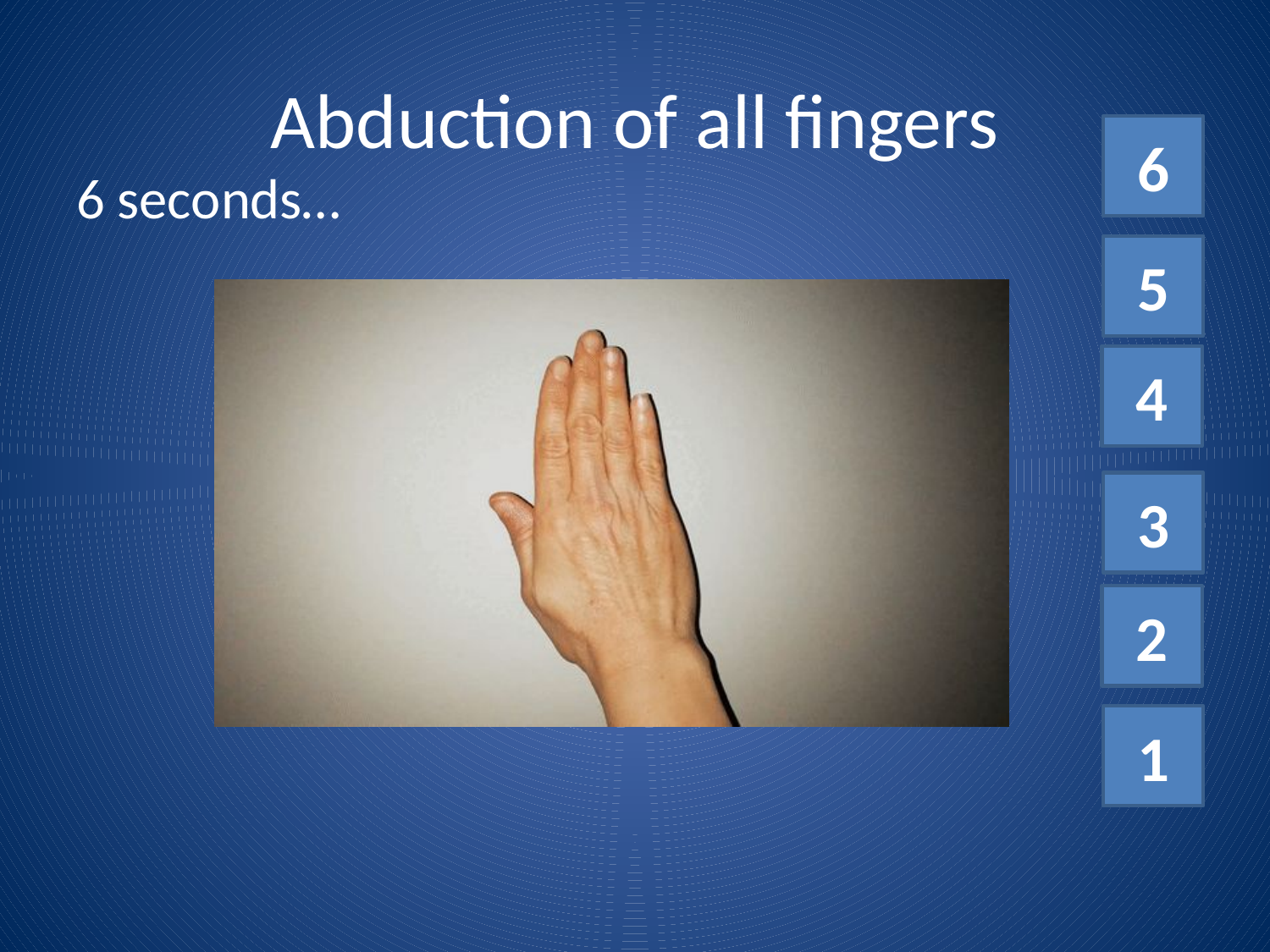

# Abduction of all fingers
6
6 seconds…
5
4
3
2
1

## Slide 85
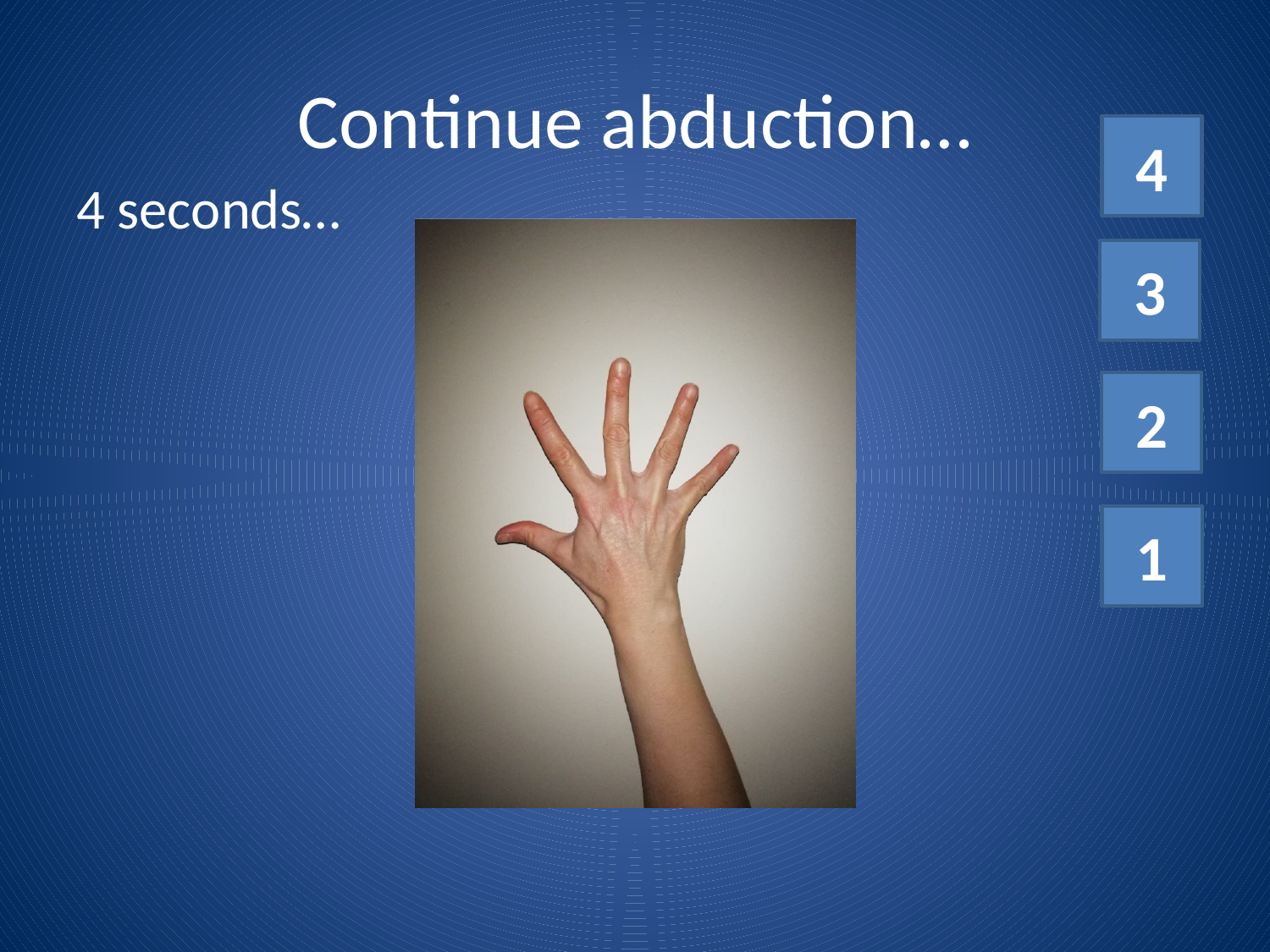

# Continue abduction…
4
4 seconds…
3
2
1

## Slide 86
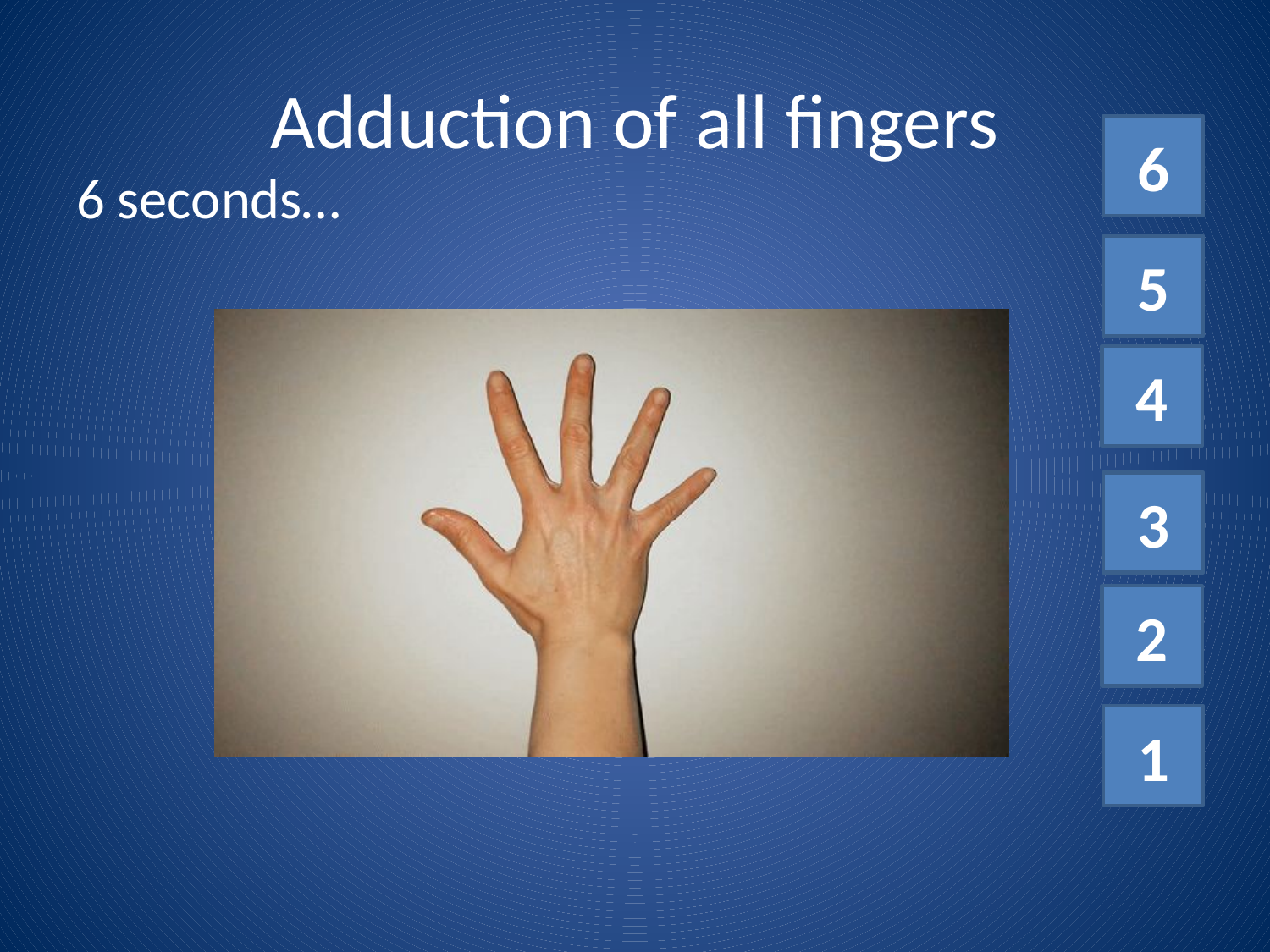

# Adduction of all fingers
6
6 seconds…
5
4
3
2
1

## Slide 87
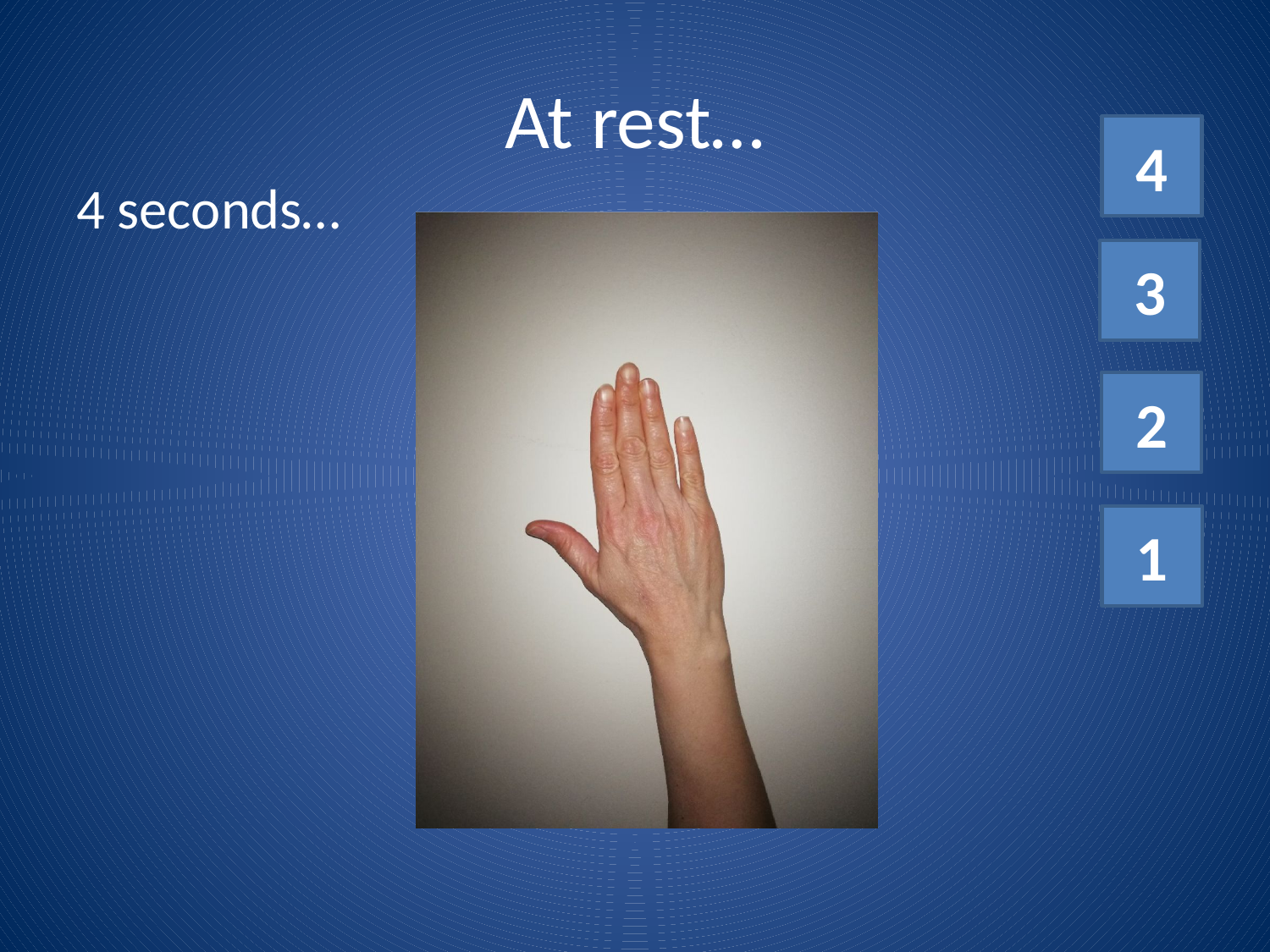

# At rest…
4
4 seconds…
3
2
1

## Slide 88
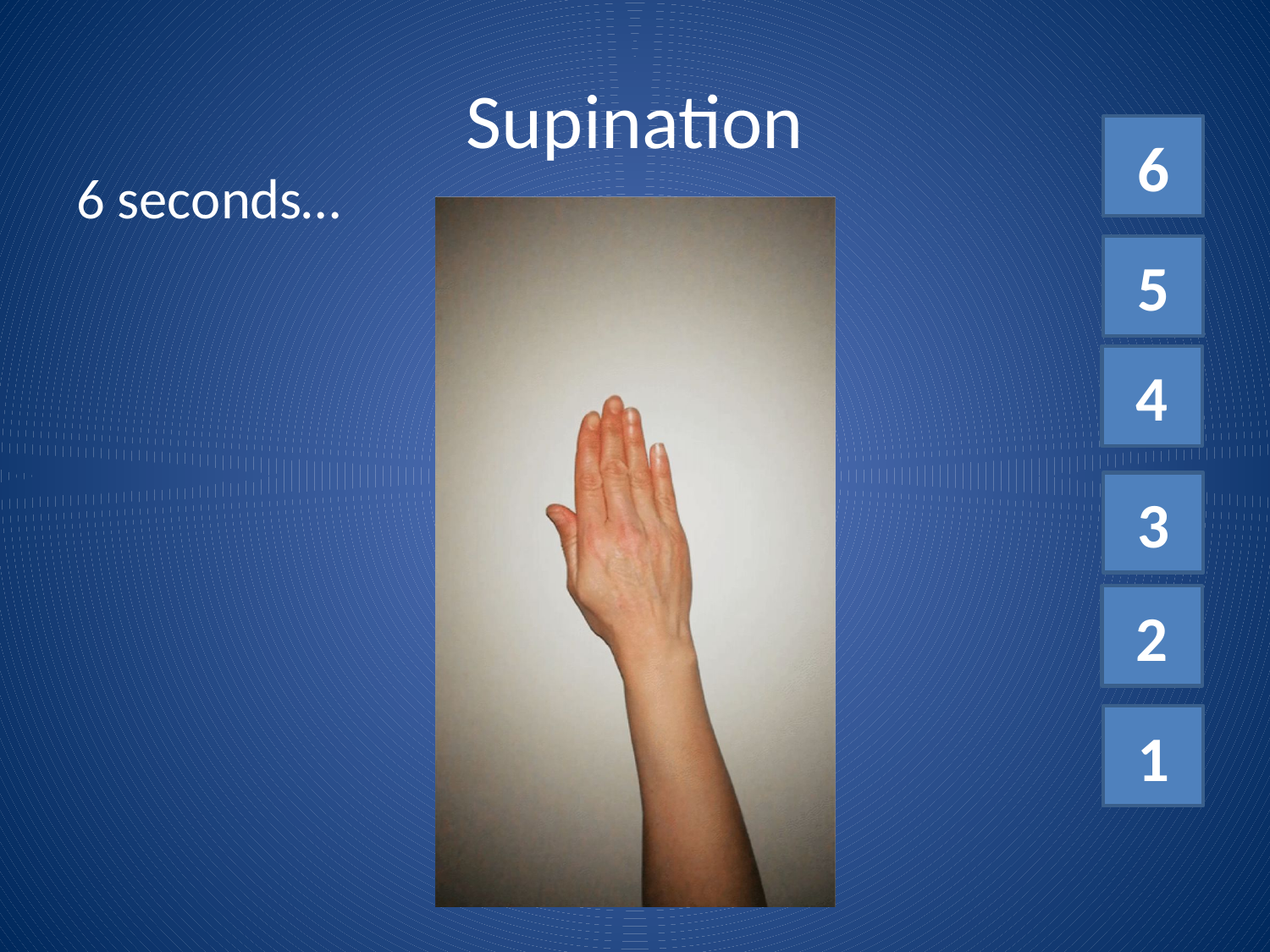

# Supination
6
6 seconds…
5
4
3
2
1

## Slide 89
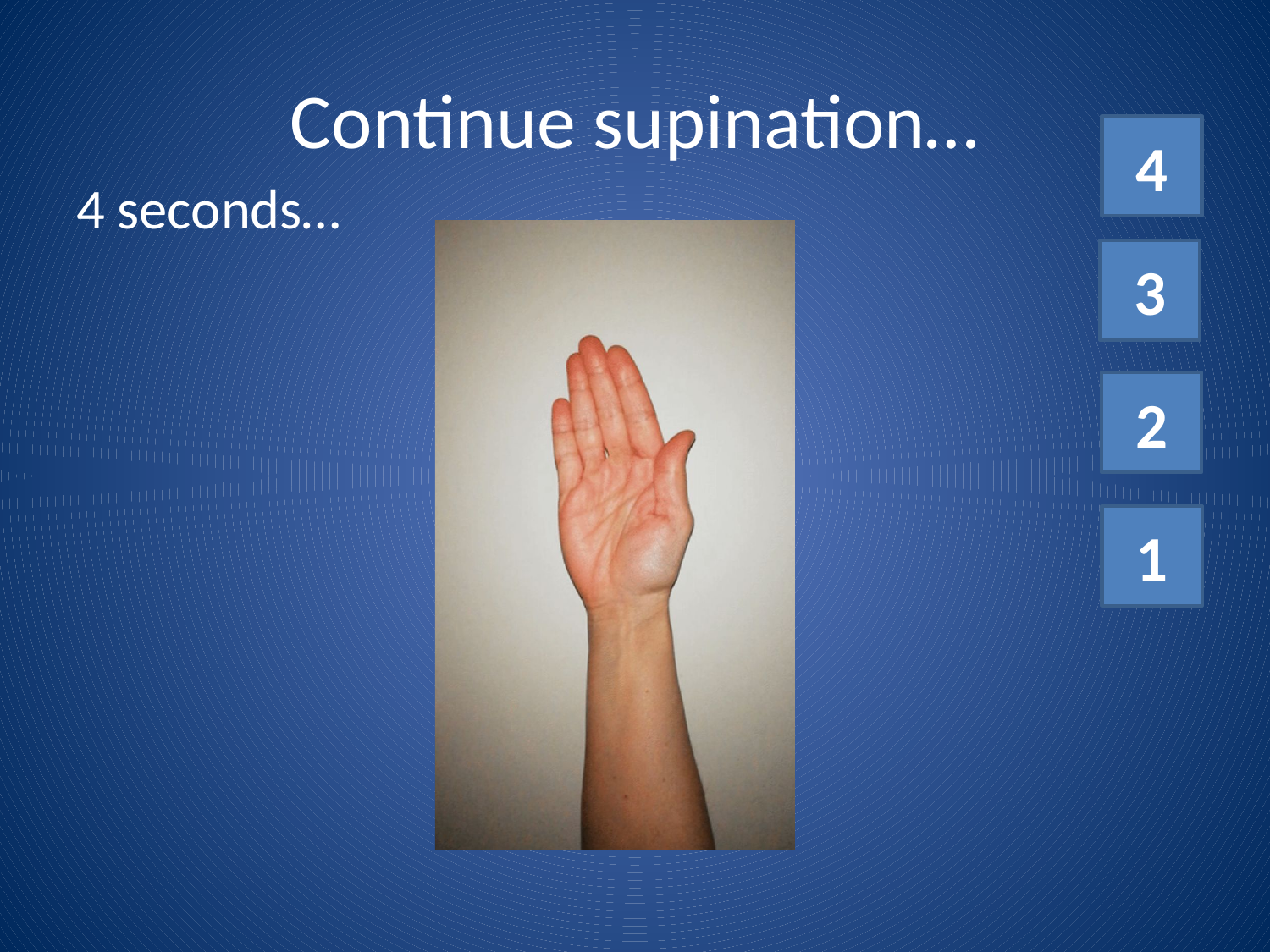

# Continue supination…
4
4 seconds…
3
2
1

## Slide 90
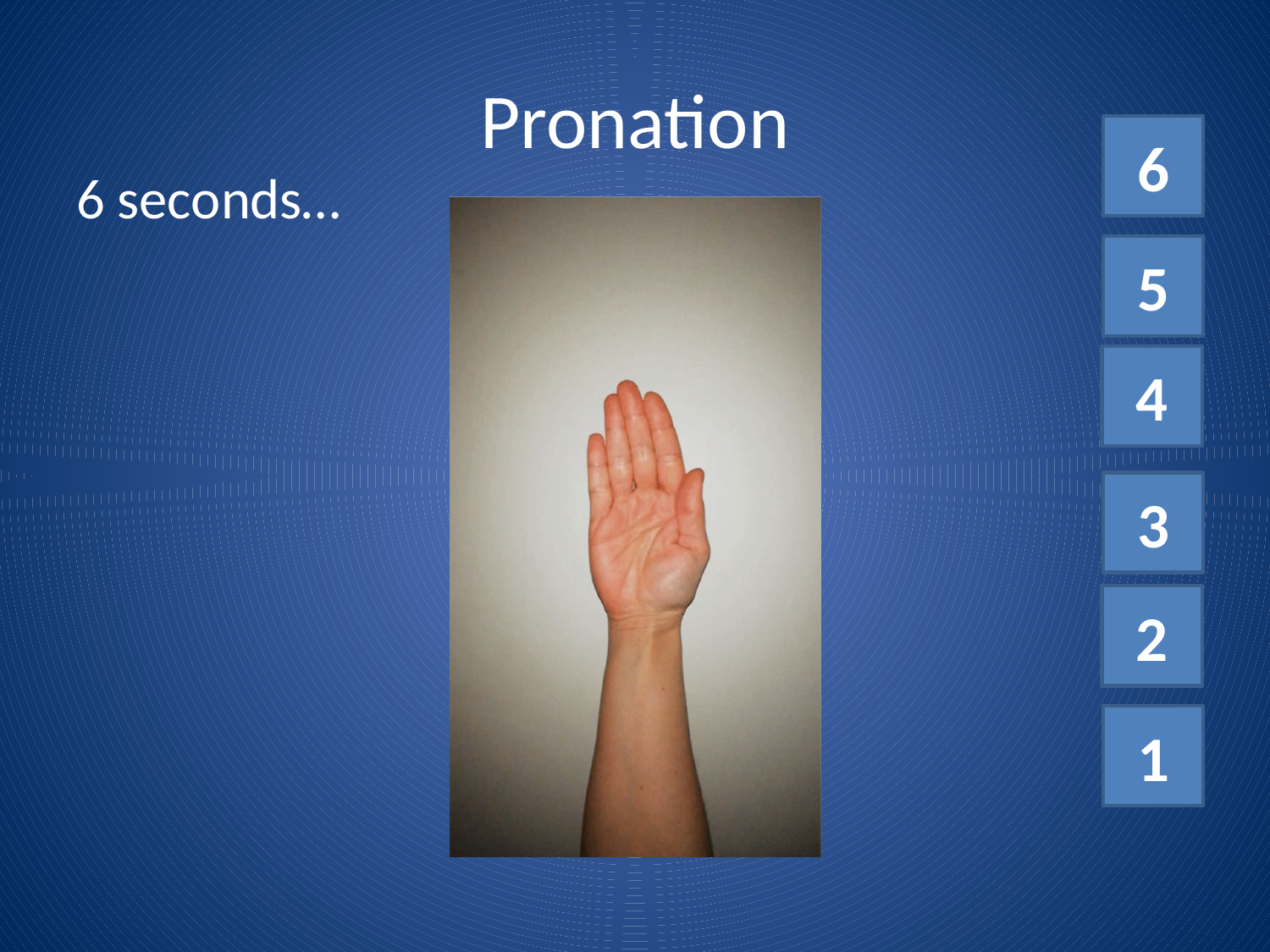

# Pronation
6
6 seconds…
5
4
3
2
1

## Slide 91
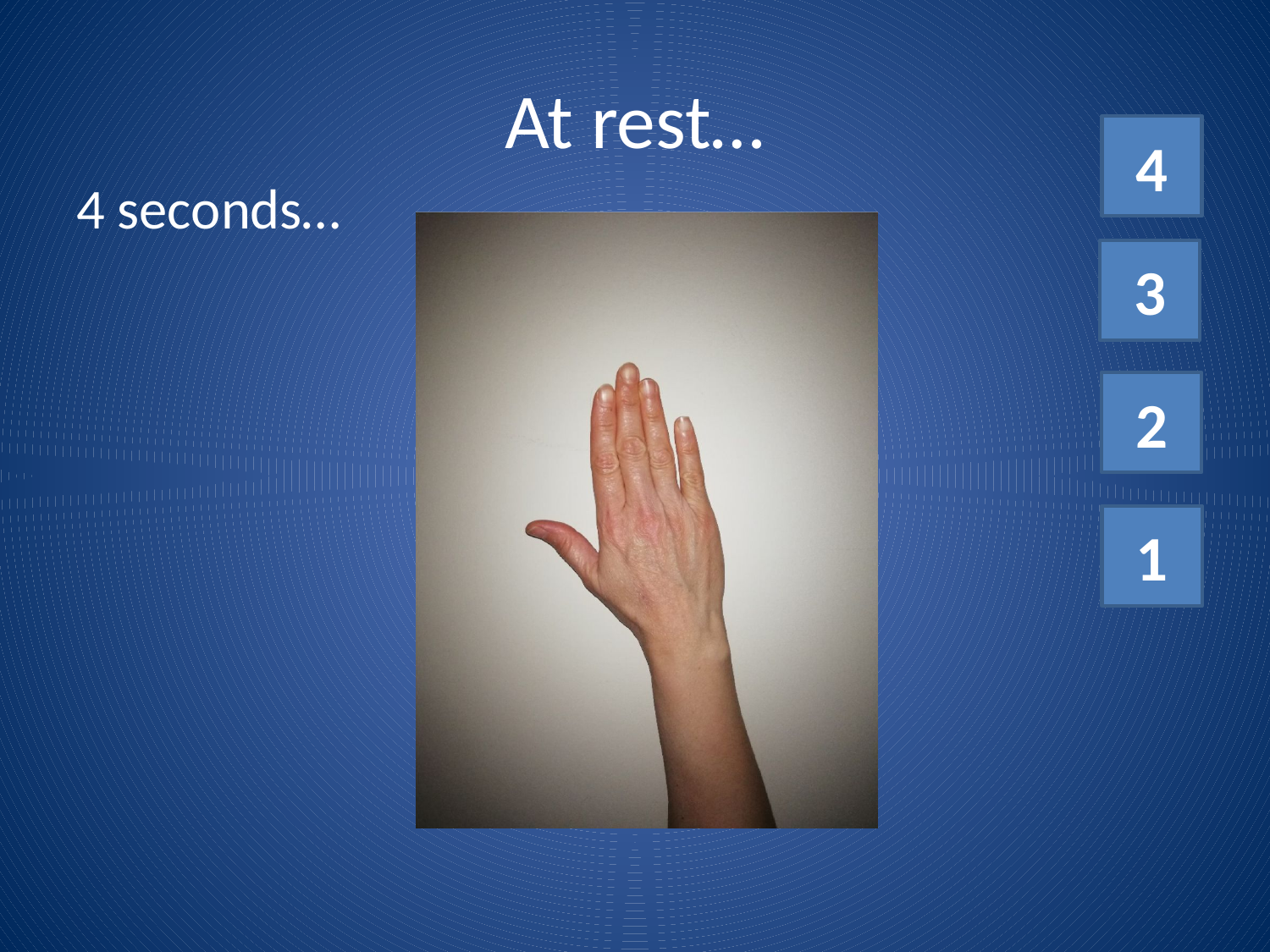

# At rest…
4
4 seconds…
3
2
1

## Slide 92
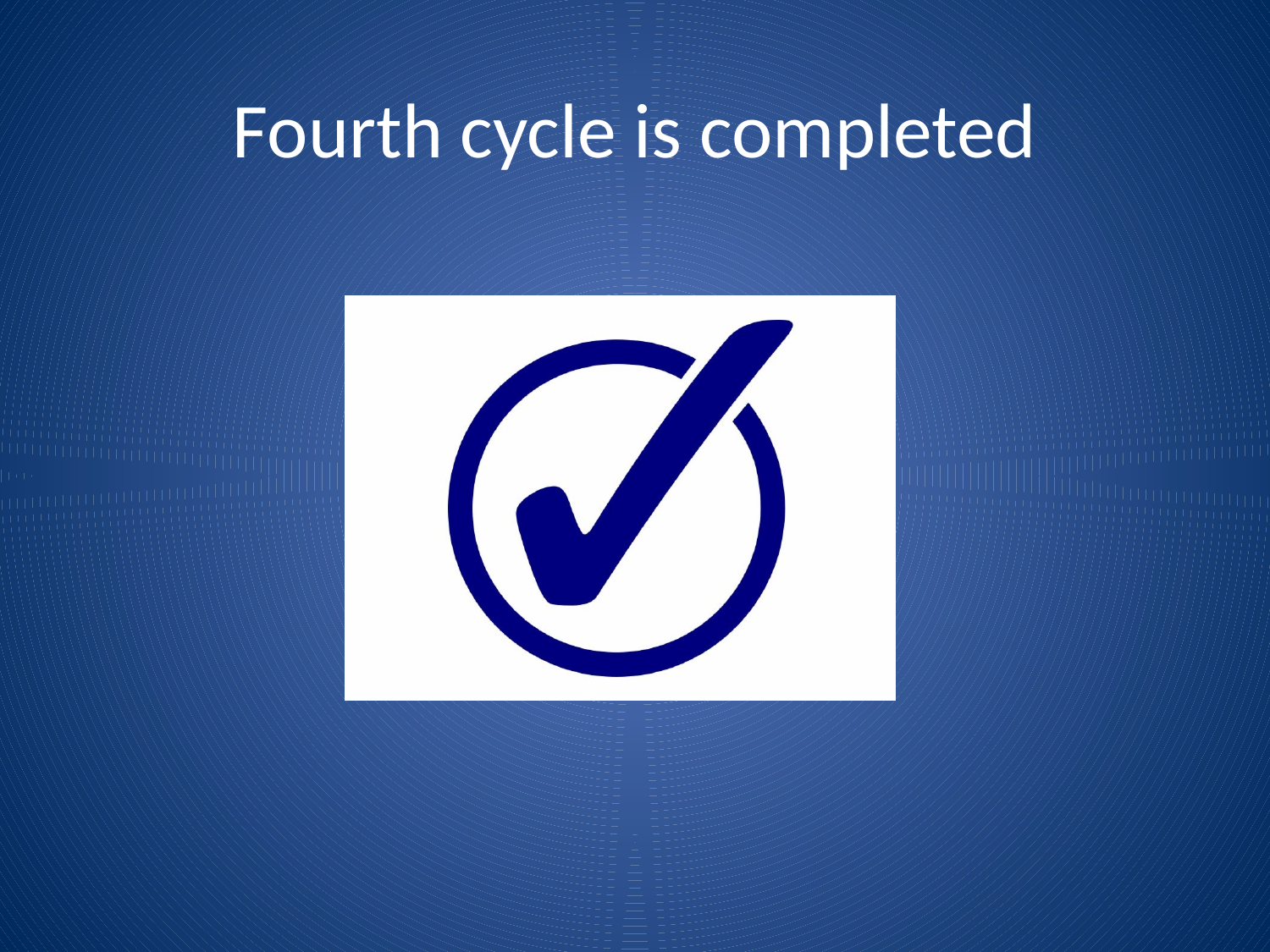

# Fourth cycle is completed

## Slide 93
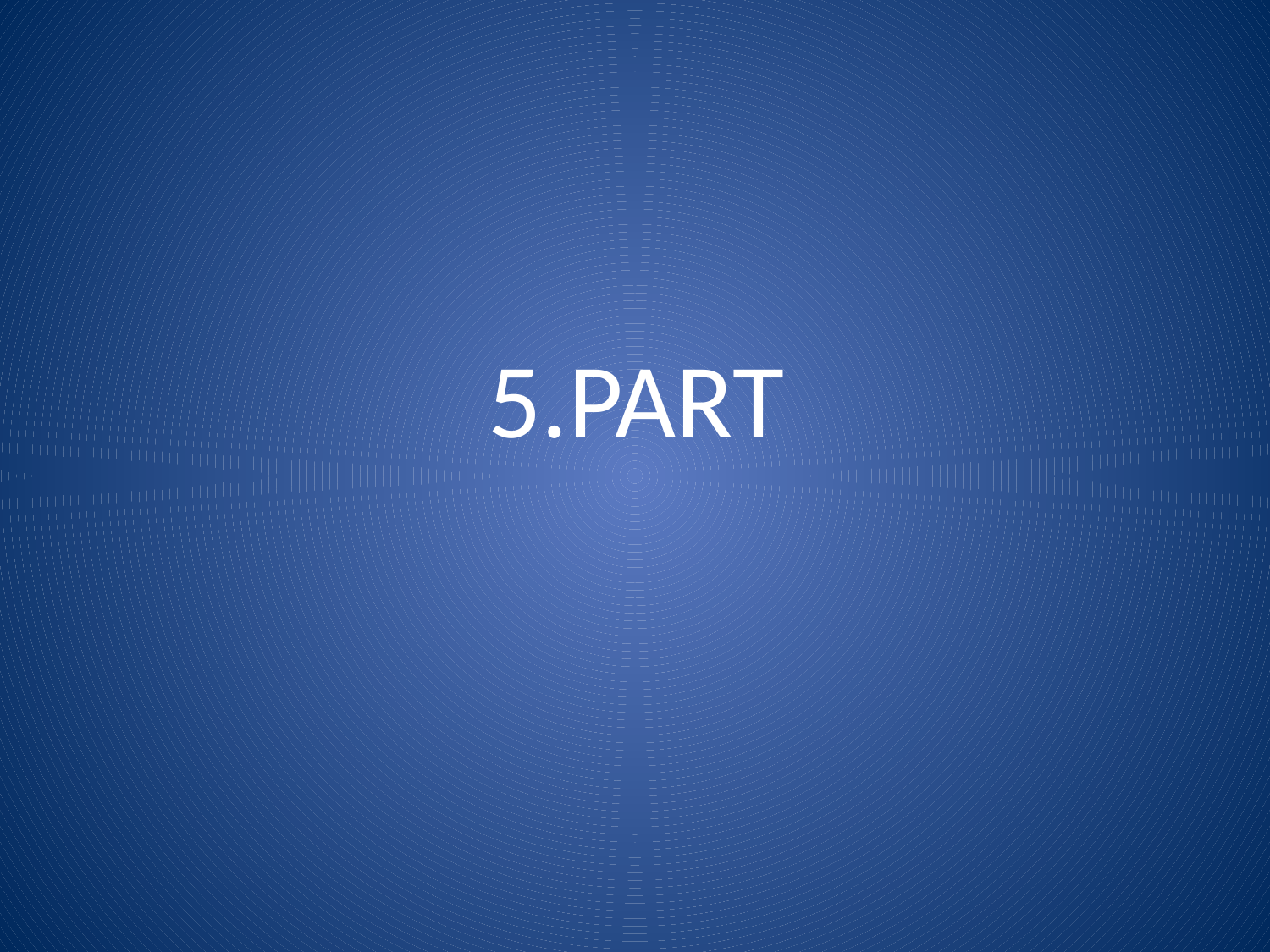

# 5.PART

## Slide 94
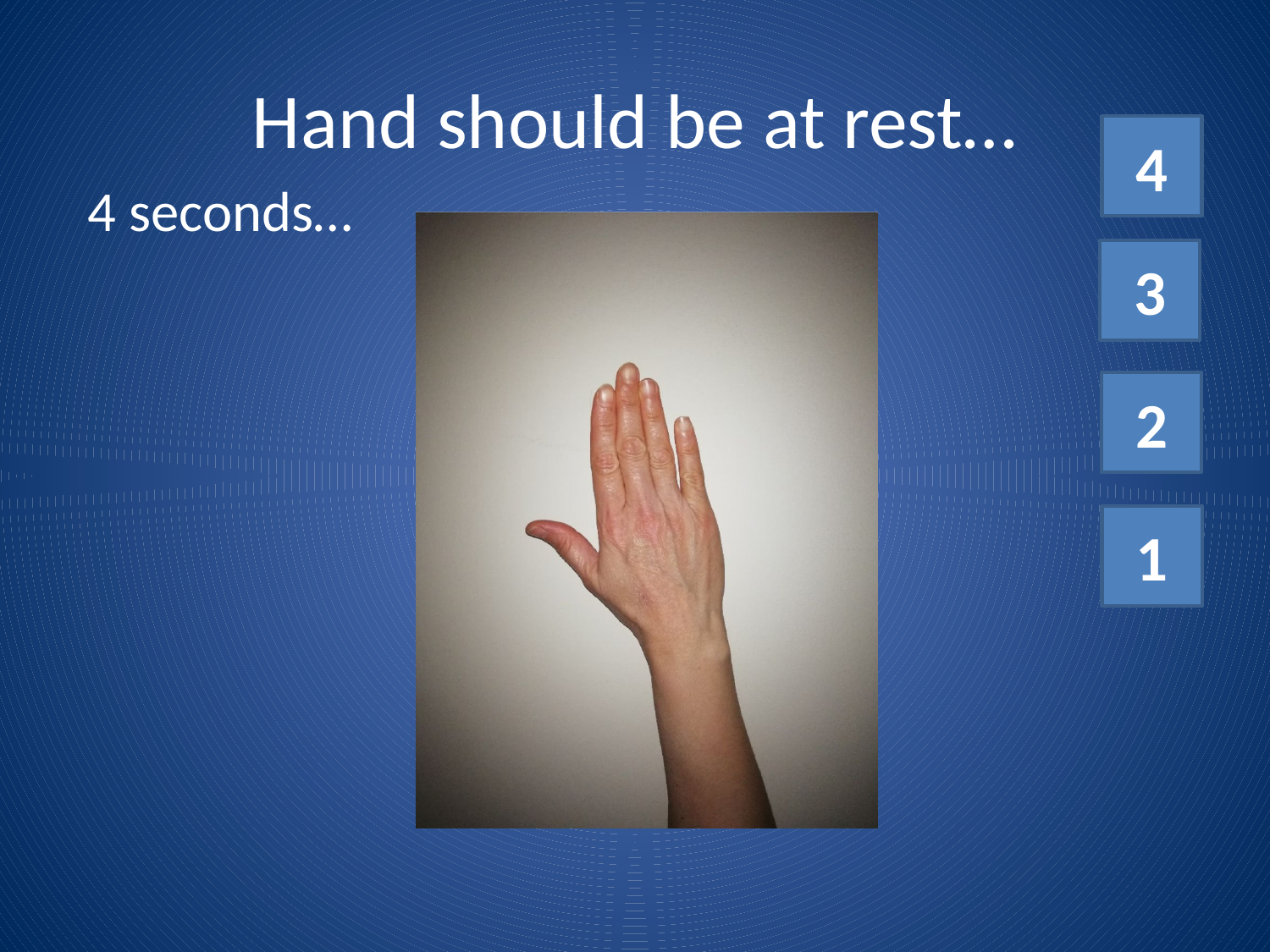

# Hand should be at rest…
4
4 seconds…
3
2
1

## Slide 95
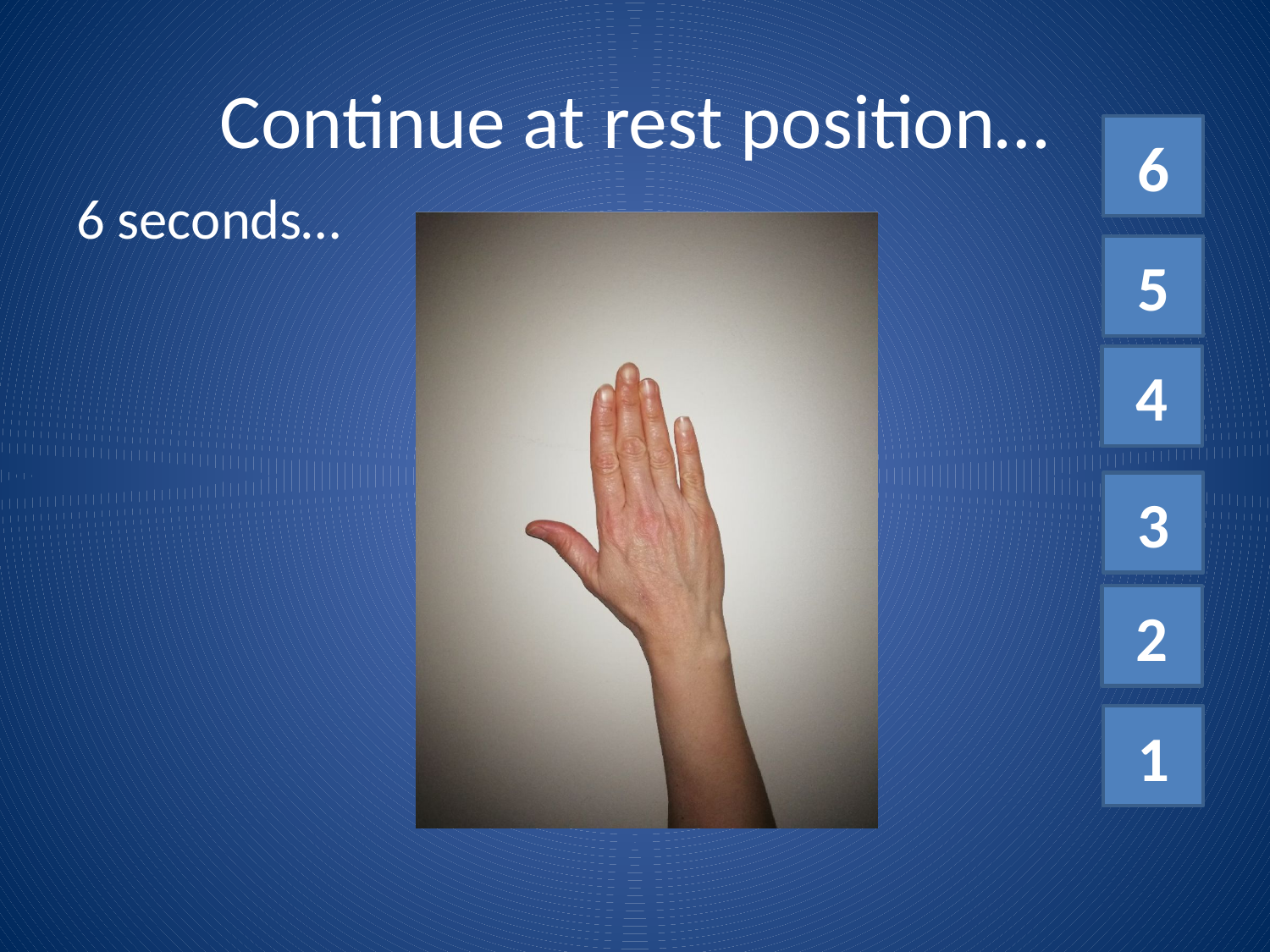

# Continue at rest position…
6
6 seconds…
5
4
3
2
1

## Slide 96
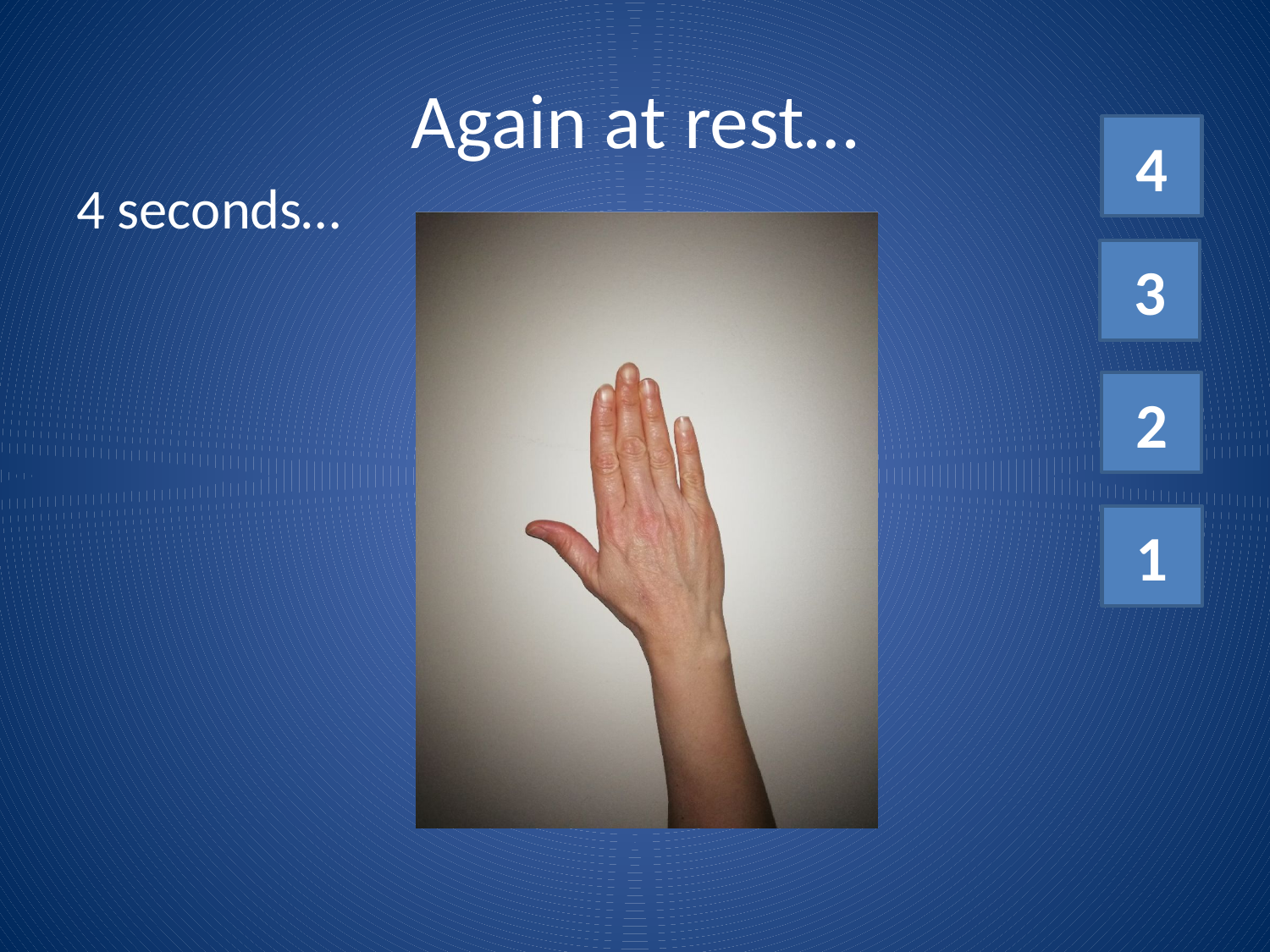

# Again at rest…
4
4 seconds…
3
2
1

## Slide 97
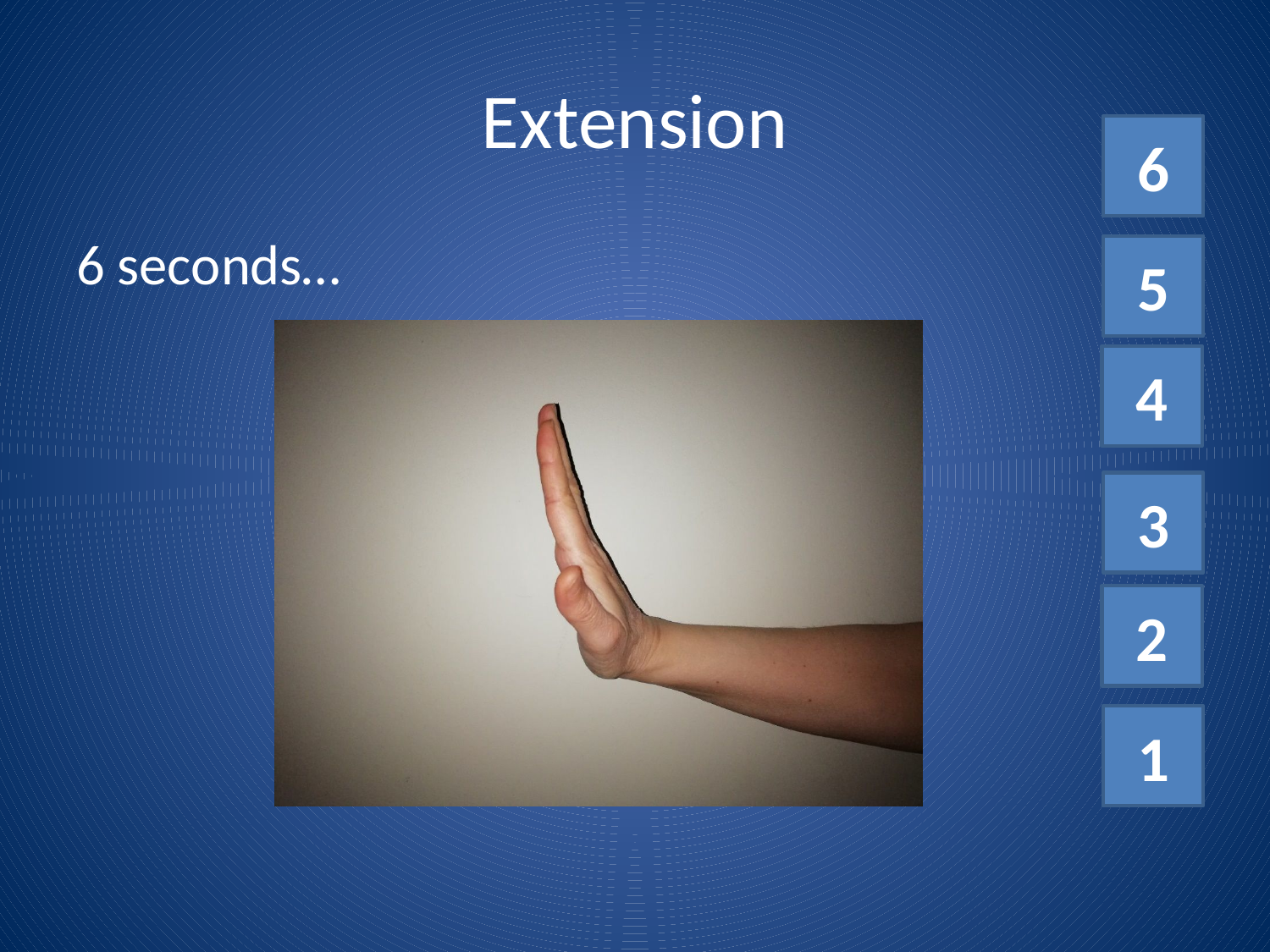

# Extension
6
6 seconds…
5
4
3
2
1

## Slide 98
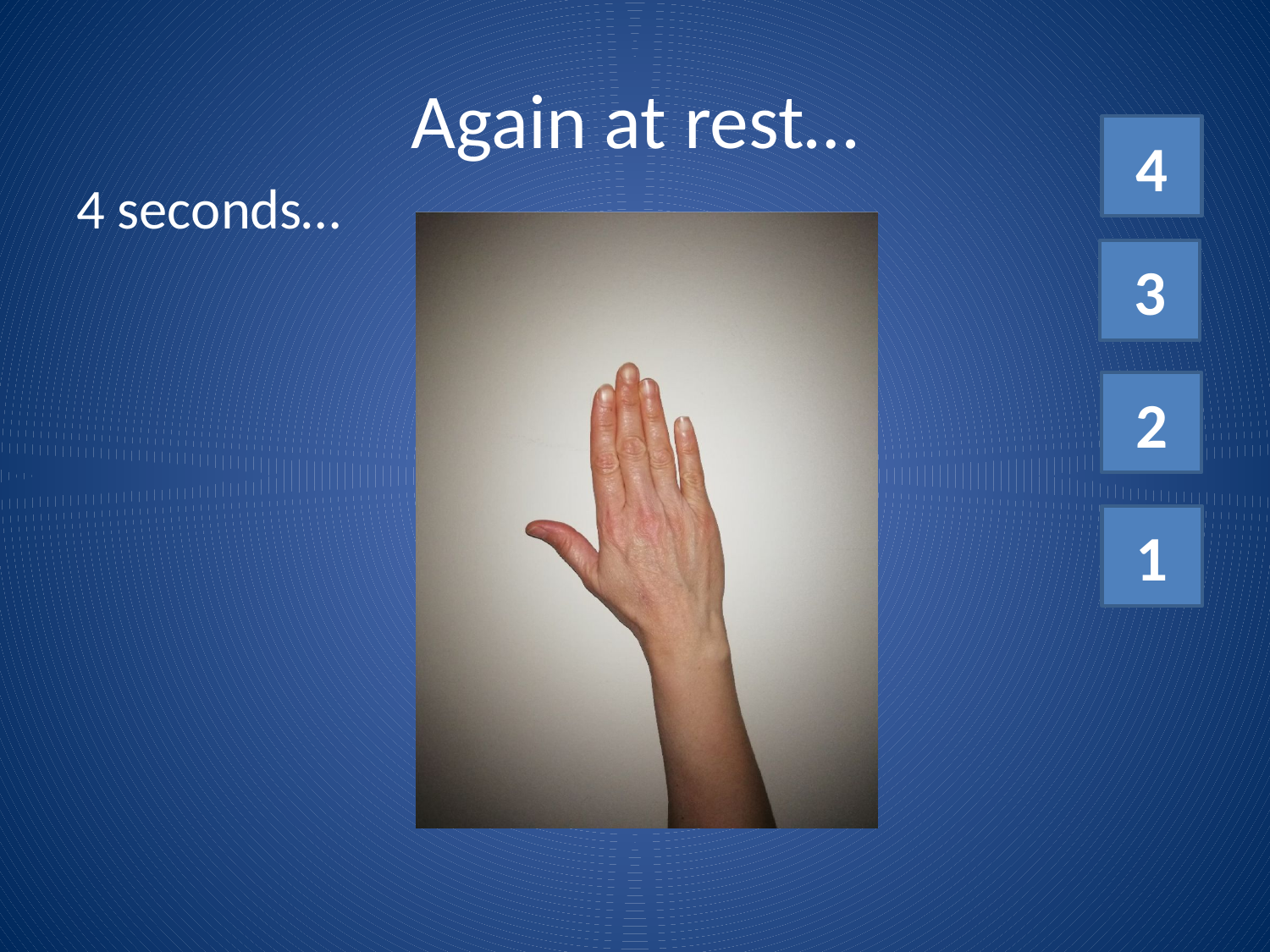

# Again at rest…
4
4 seconds…
3
2
1

## Slide 99
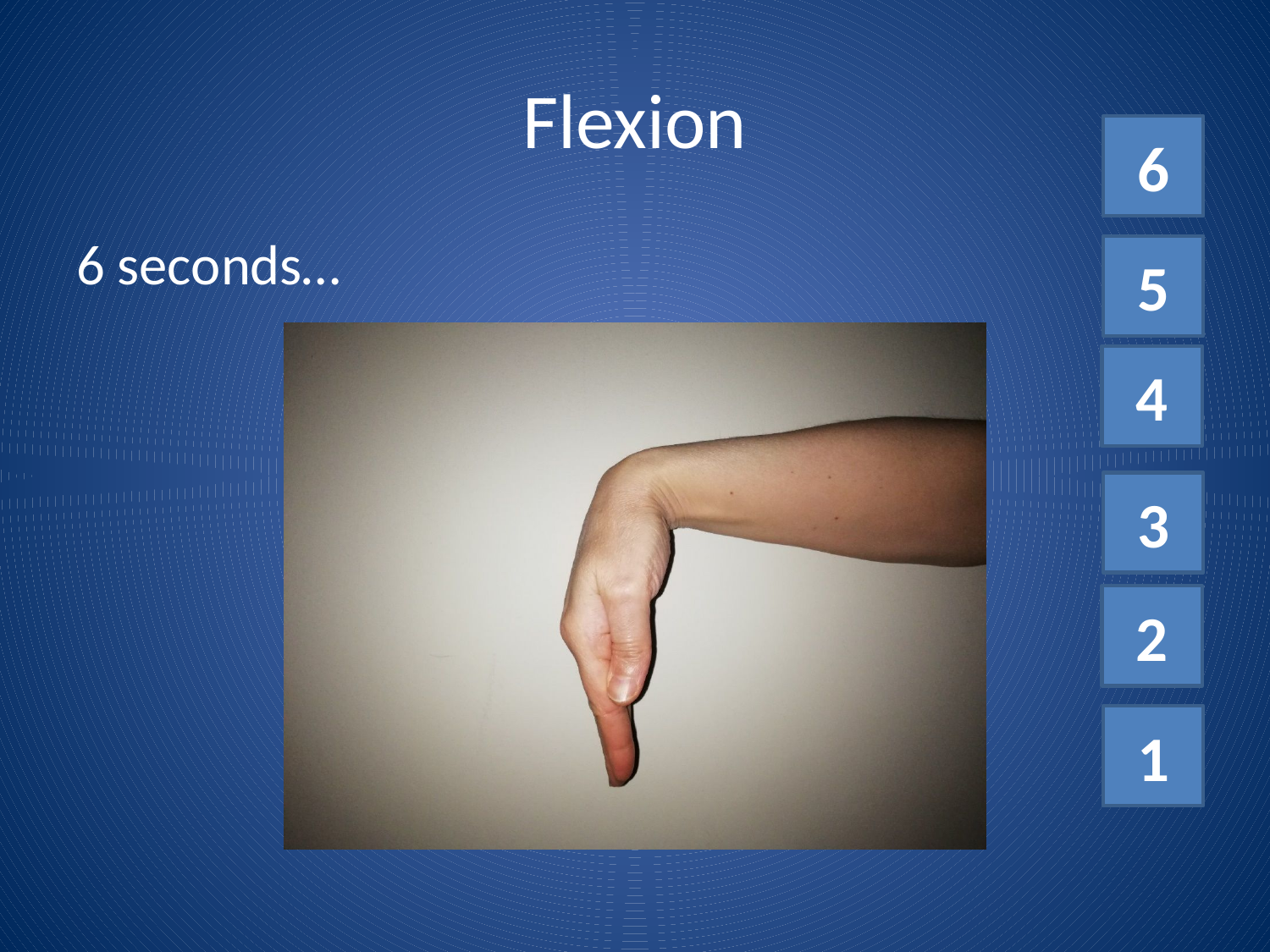

# Flexion
6
6 seconds…
5
4
3
2
1

## Slide 100
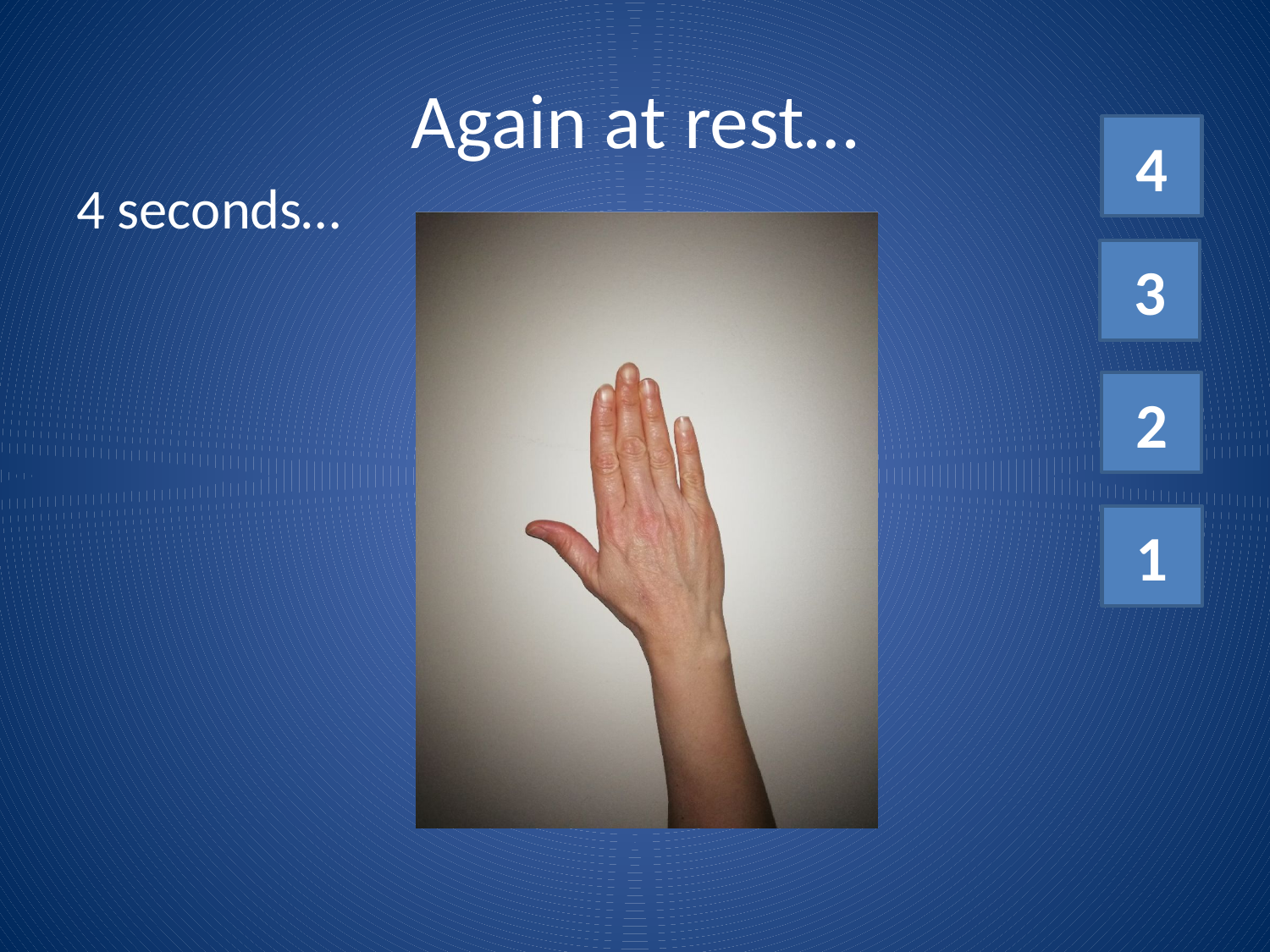

# Again at rest…
4
4 seconds…
3
2
1

## Slide 101
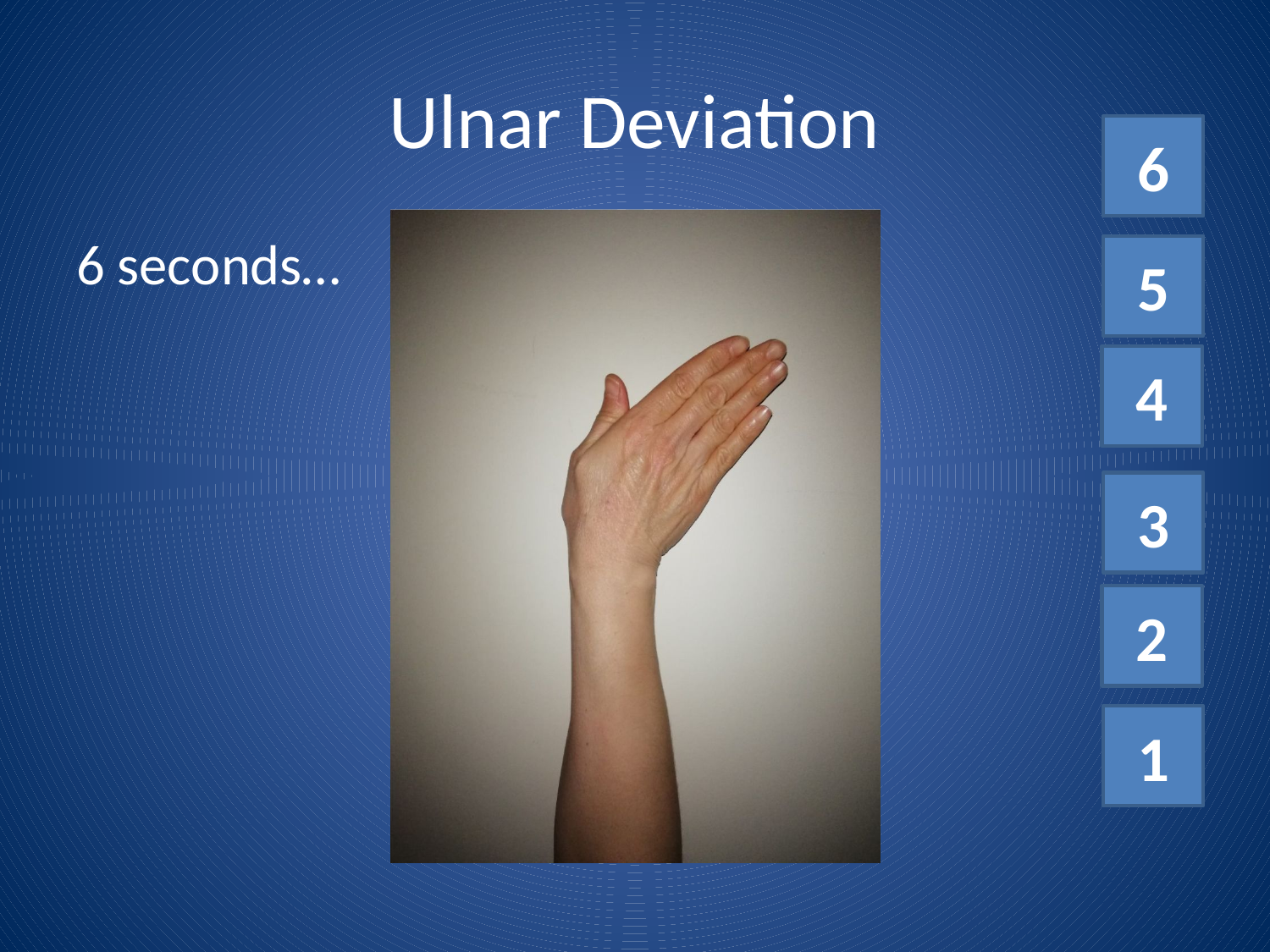

# Ulnar Deviation
6
6 seconds…
5
4
3
2
1

## Slide 102
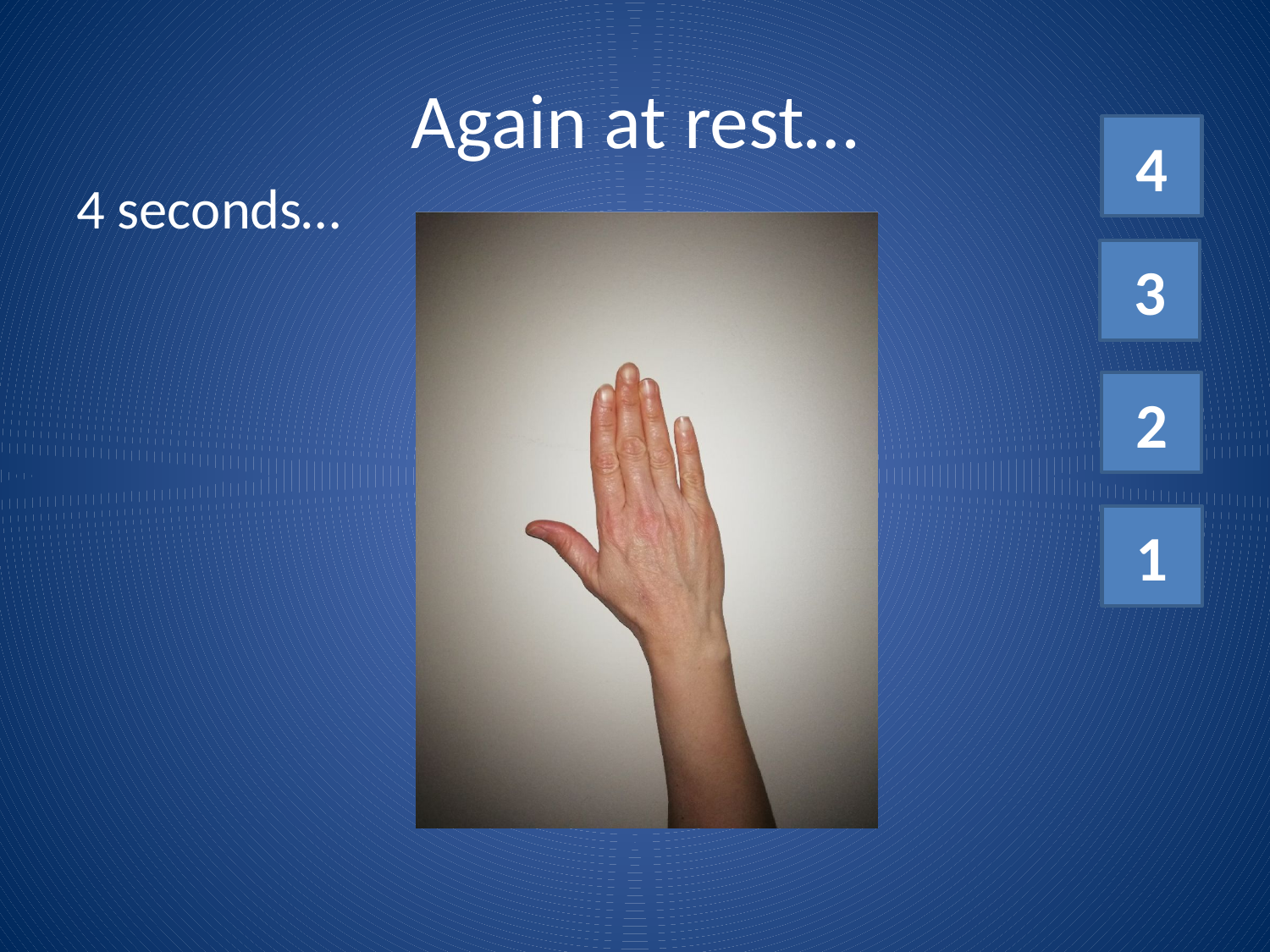

# Again at rest…
4
4 seconds…
3
2
1

## Slide 103
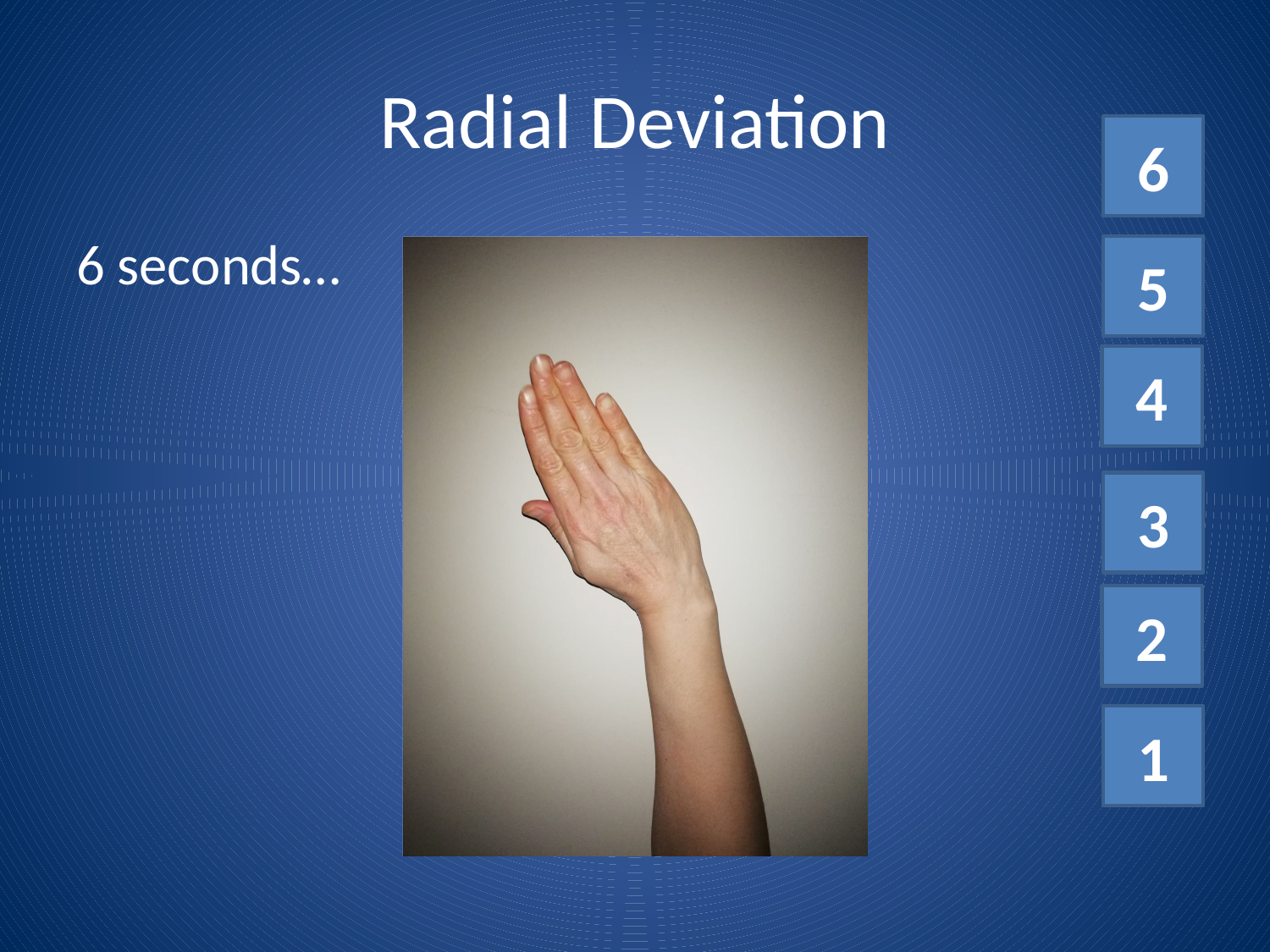

# Radial Deviation
6
6 seconds…
5
4
3
2
1

## Slide 104
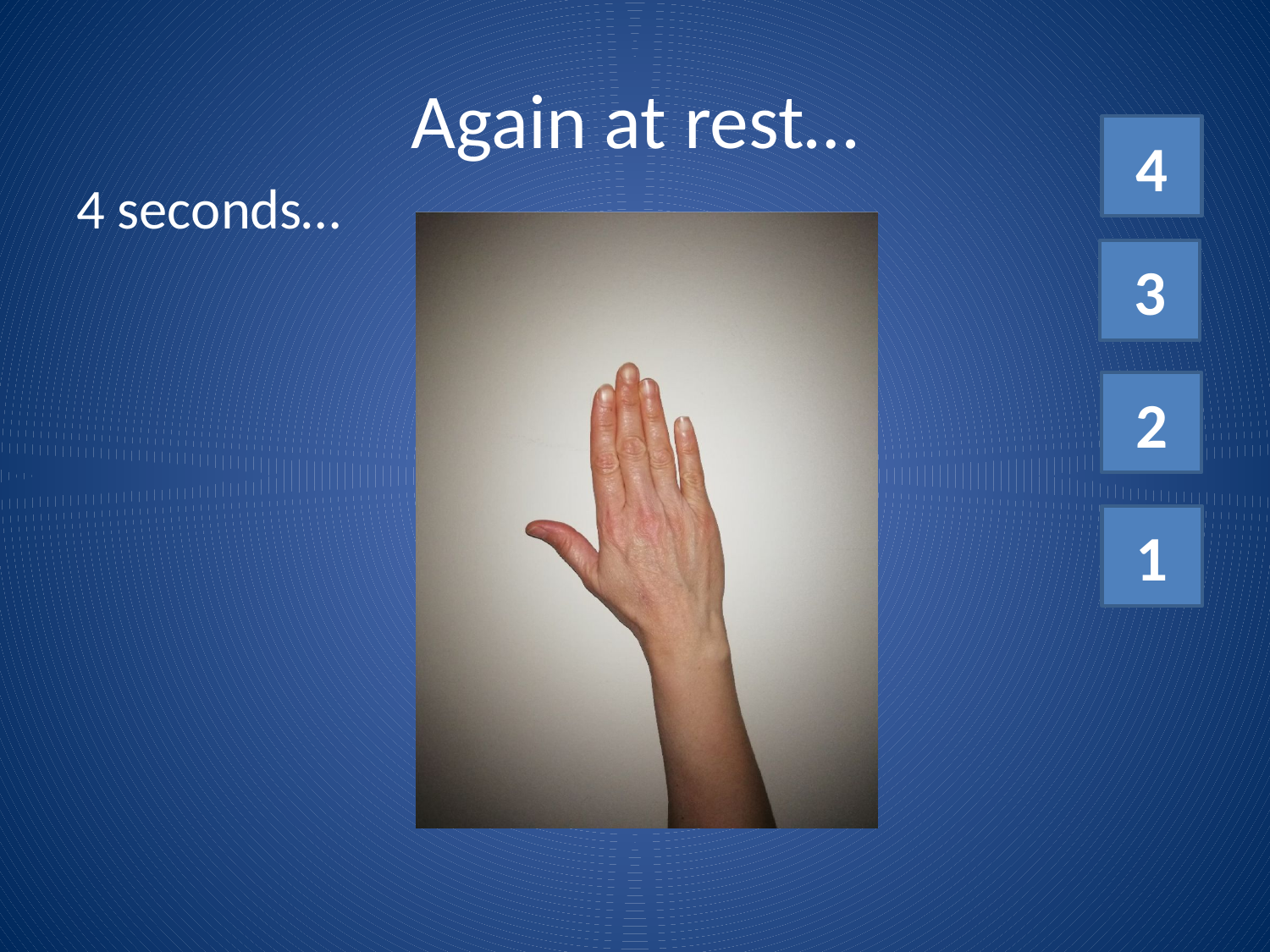

# Again at rest…
4
4 seconds…
3
2
1

## Slide 105
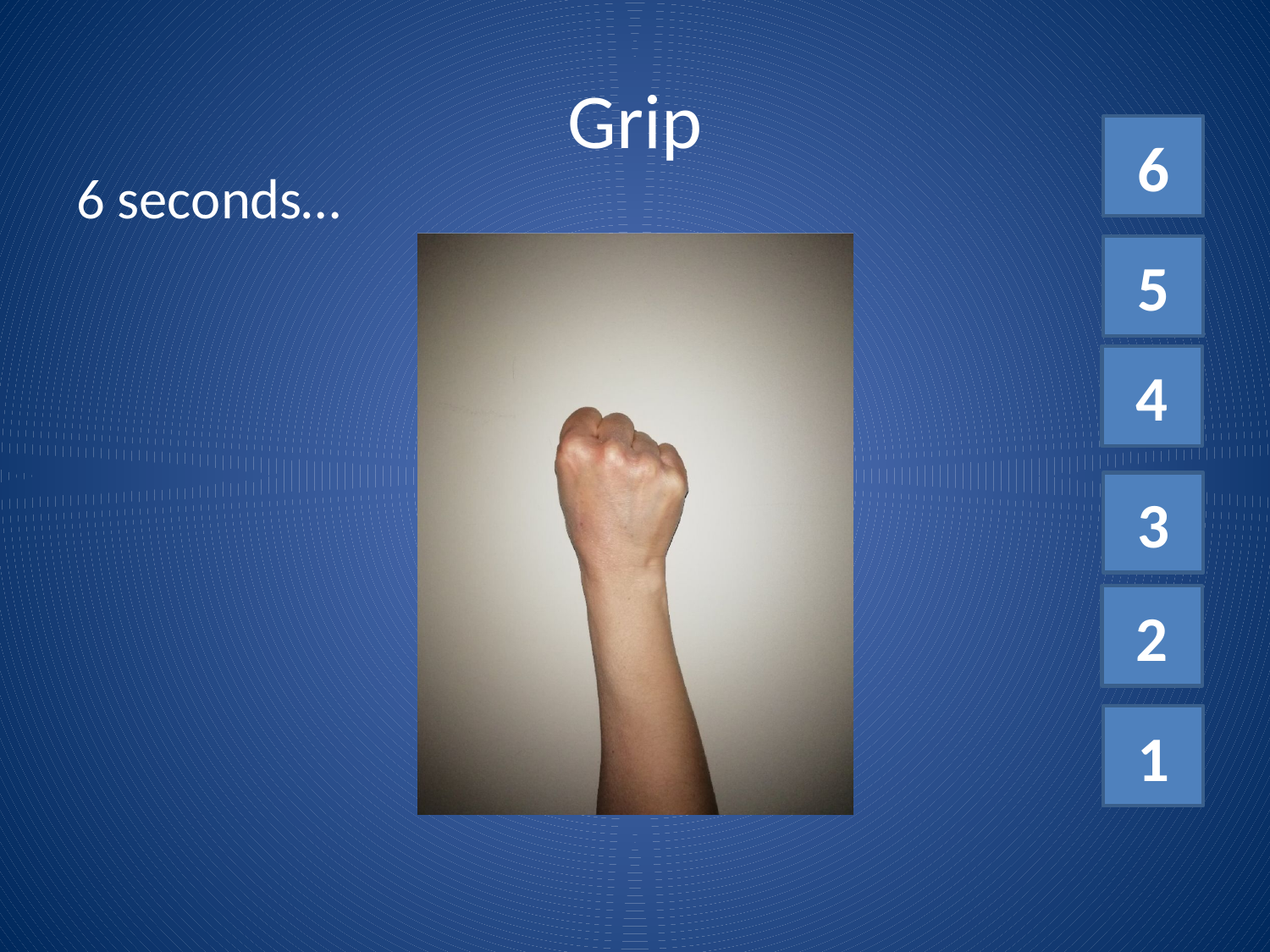

# Grip
6
6 seconds…
5
4
3
2
1

## Slide 106
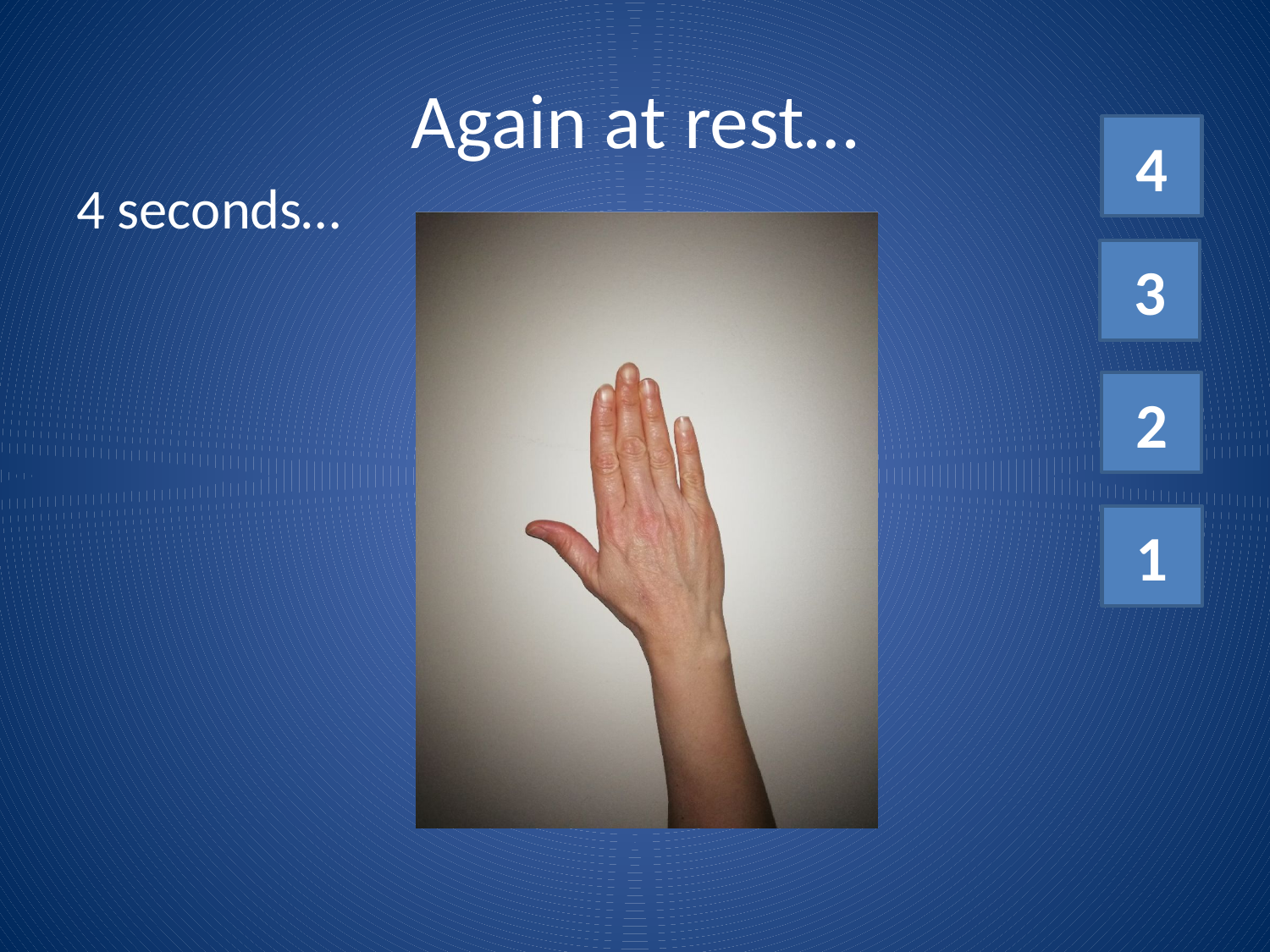

# Again at rest…
4
4 seconds…
3
2
1

## Slide 107
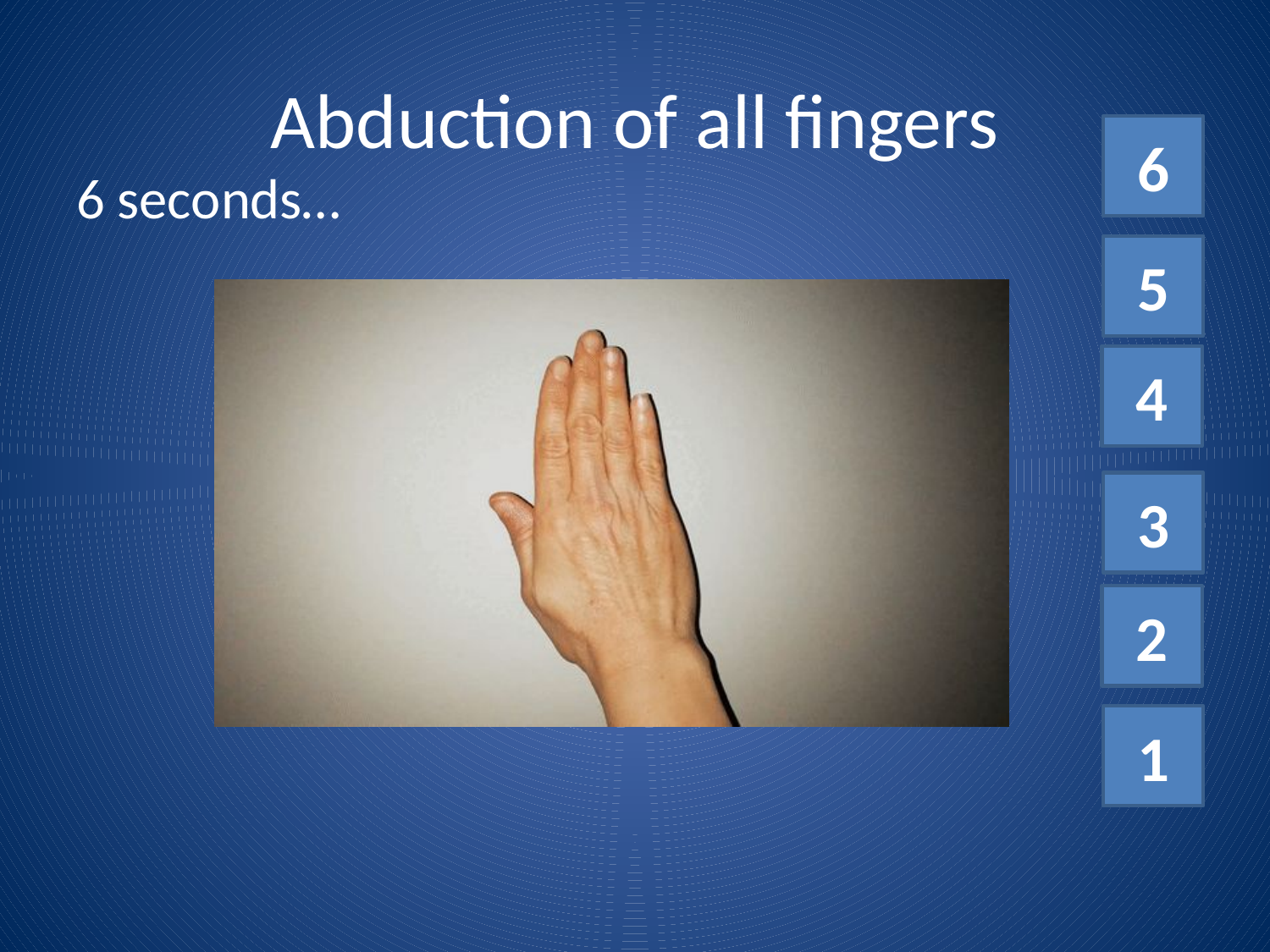

# Abduction of all fingers
6
6 seconds…
5
4
3
2
1

## Slide 108
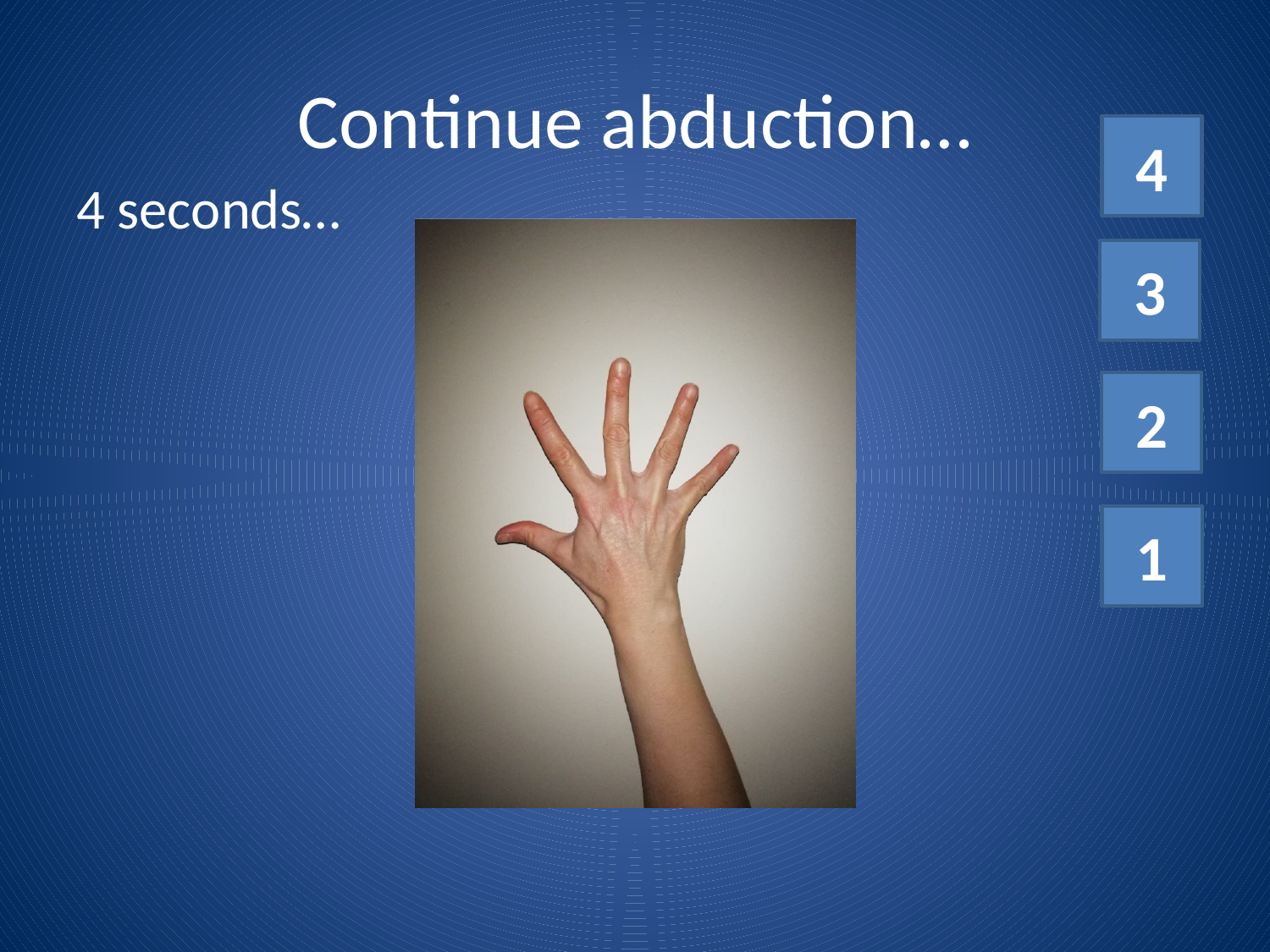

# Continue abduction…
4
4 seconds…
3
2
1

## Slide 109
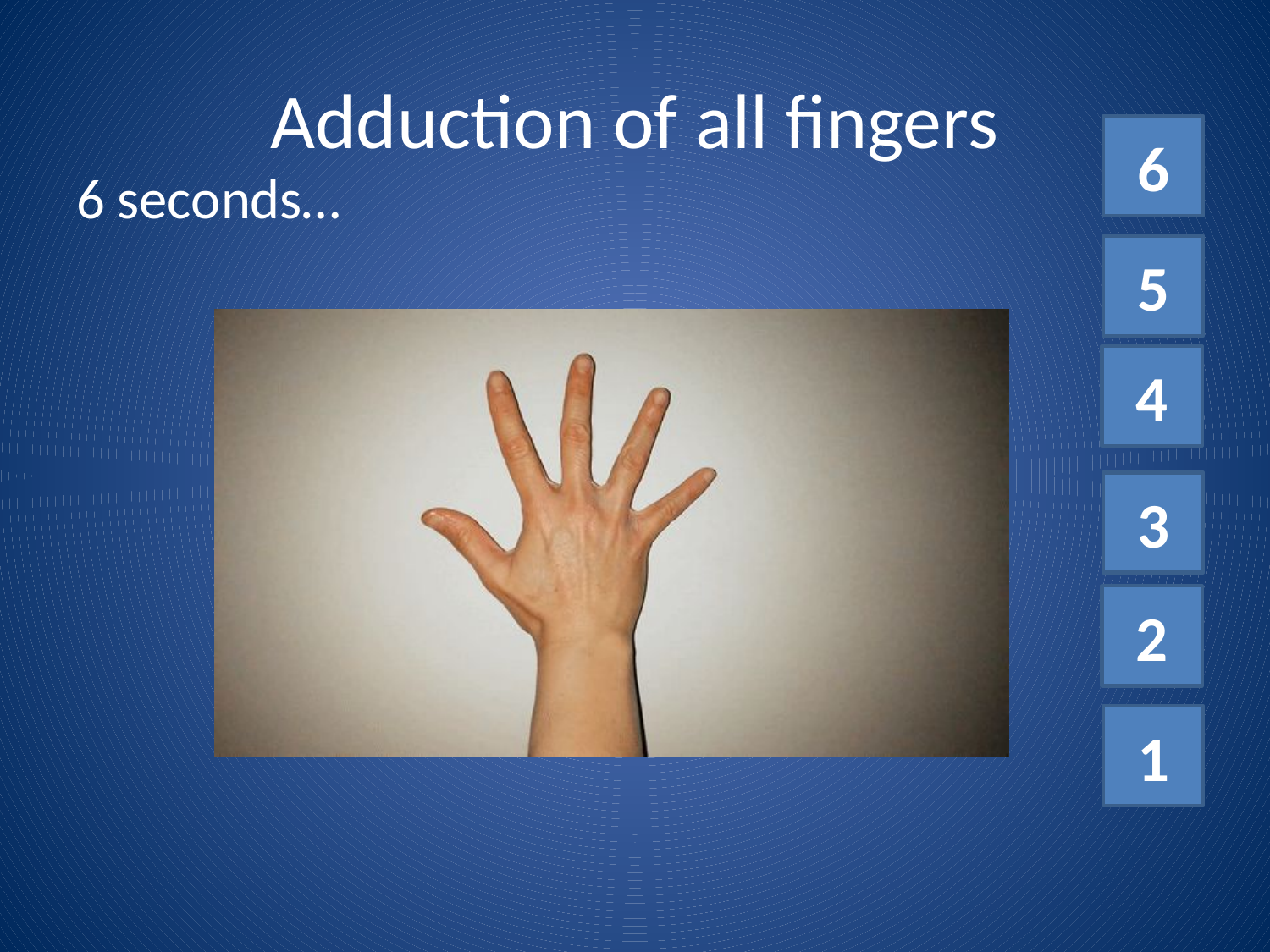

# Adduction of all fingers
6
6 seconds…
5
4
3
2
1

## Slide 110
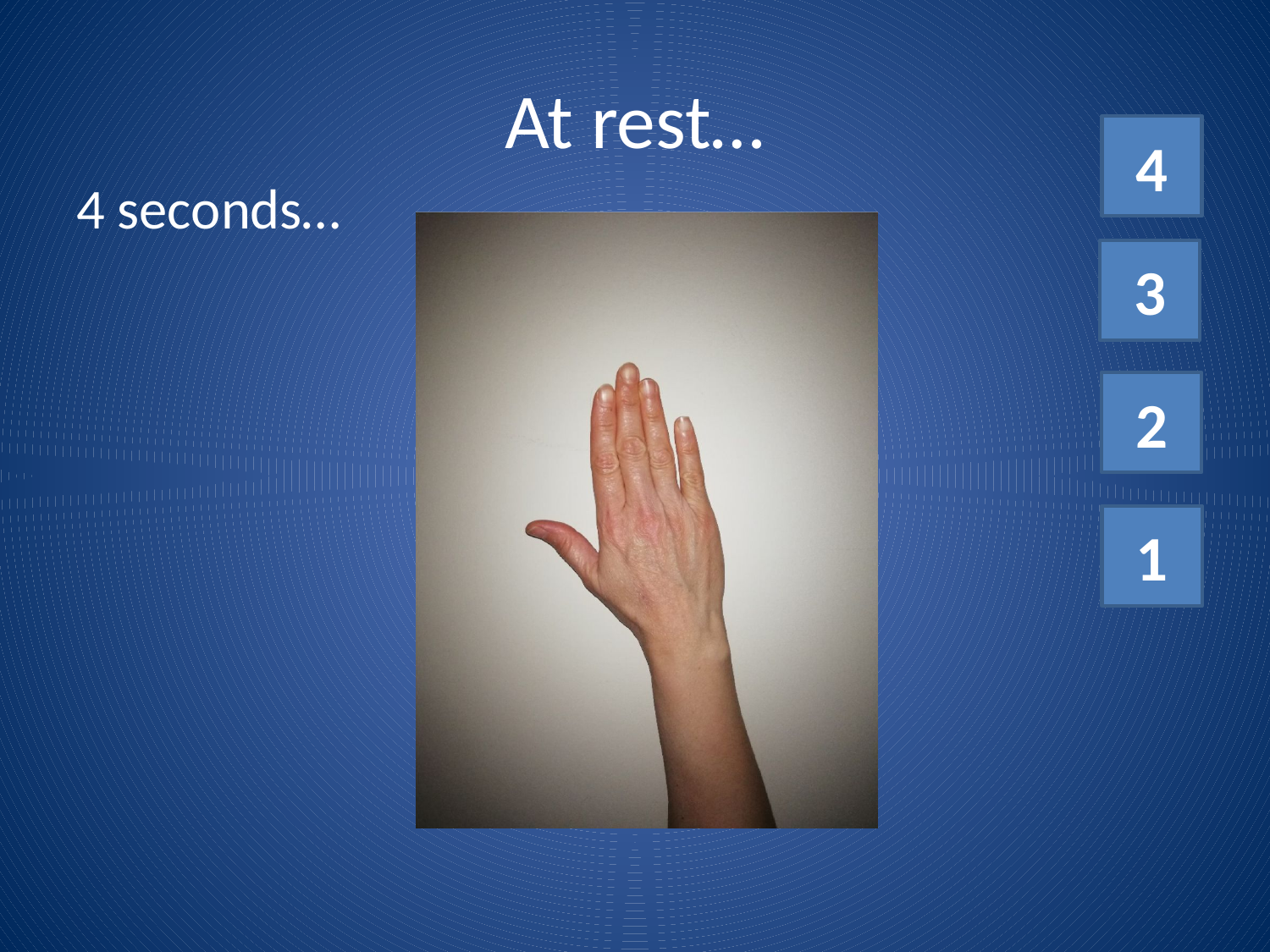

# At rest…
4
4 seconds…
3
2
1

## Slide 111
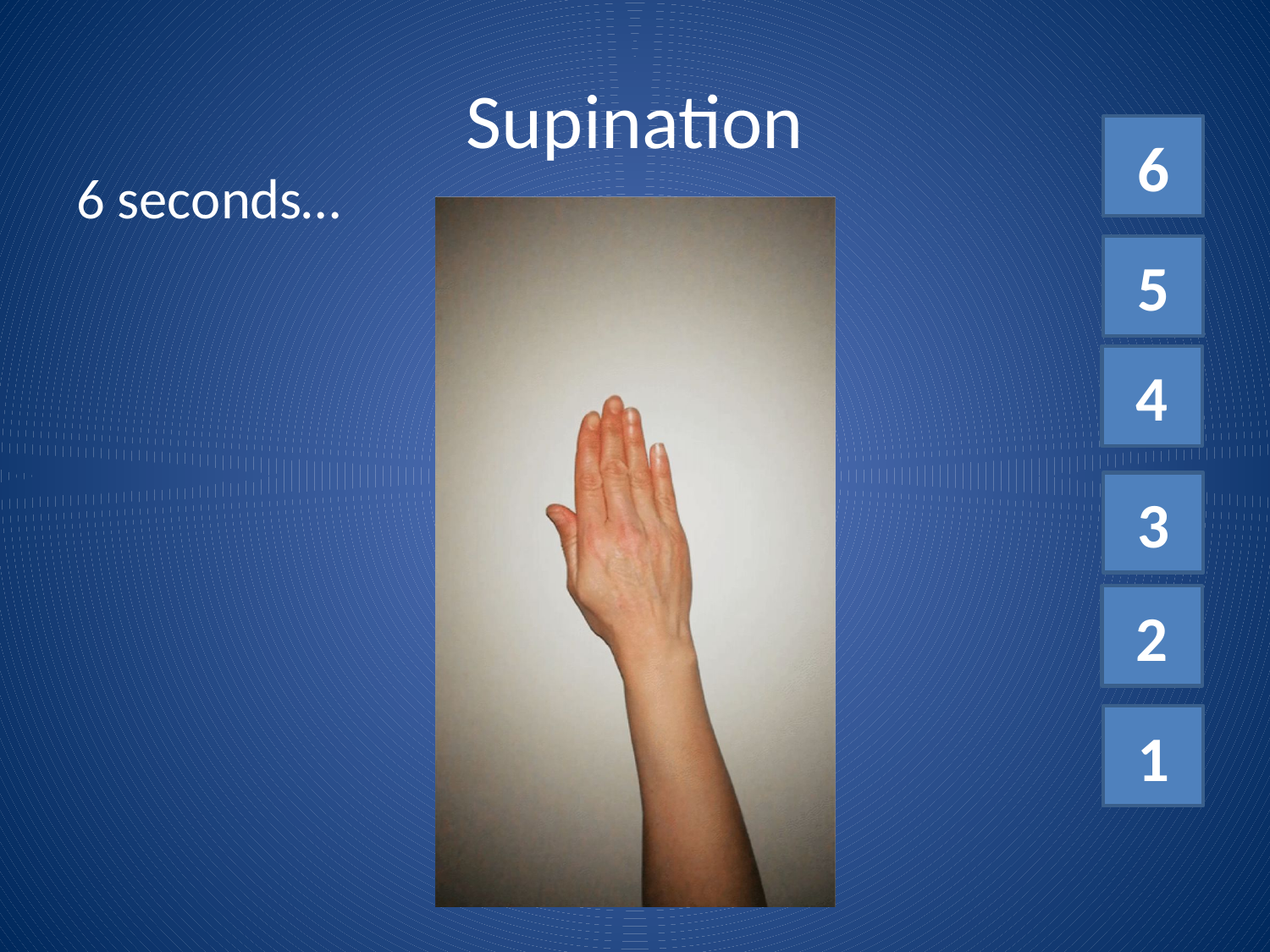

# Supination
6
6 seconds…
5
4
3
2
1

## Slide 112
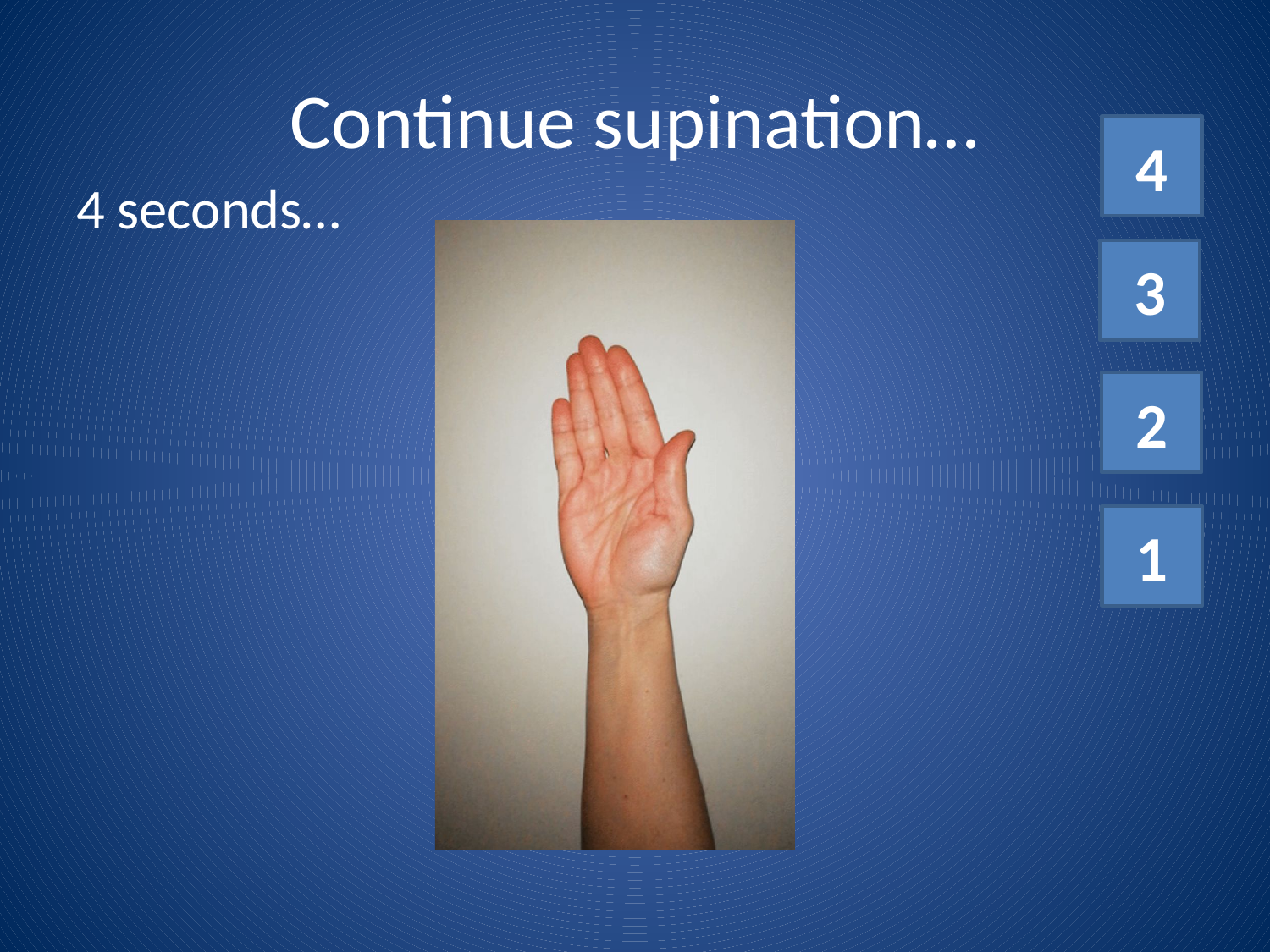

# Continue supination…
4
4 seconds…
3
2
1

## Slide 113
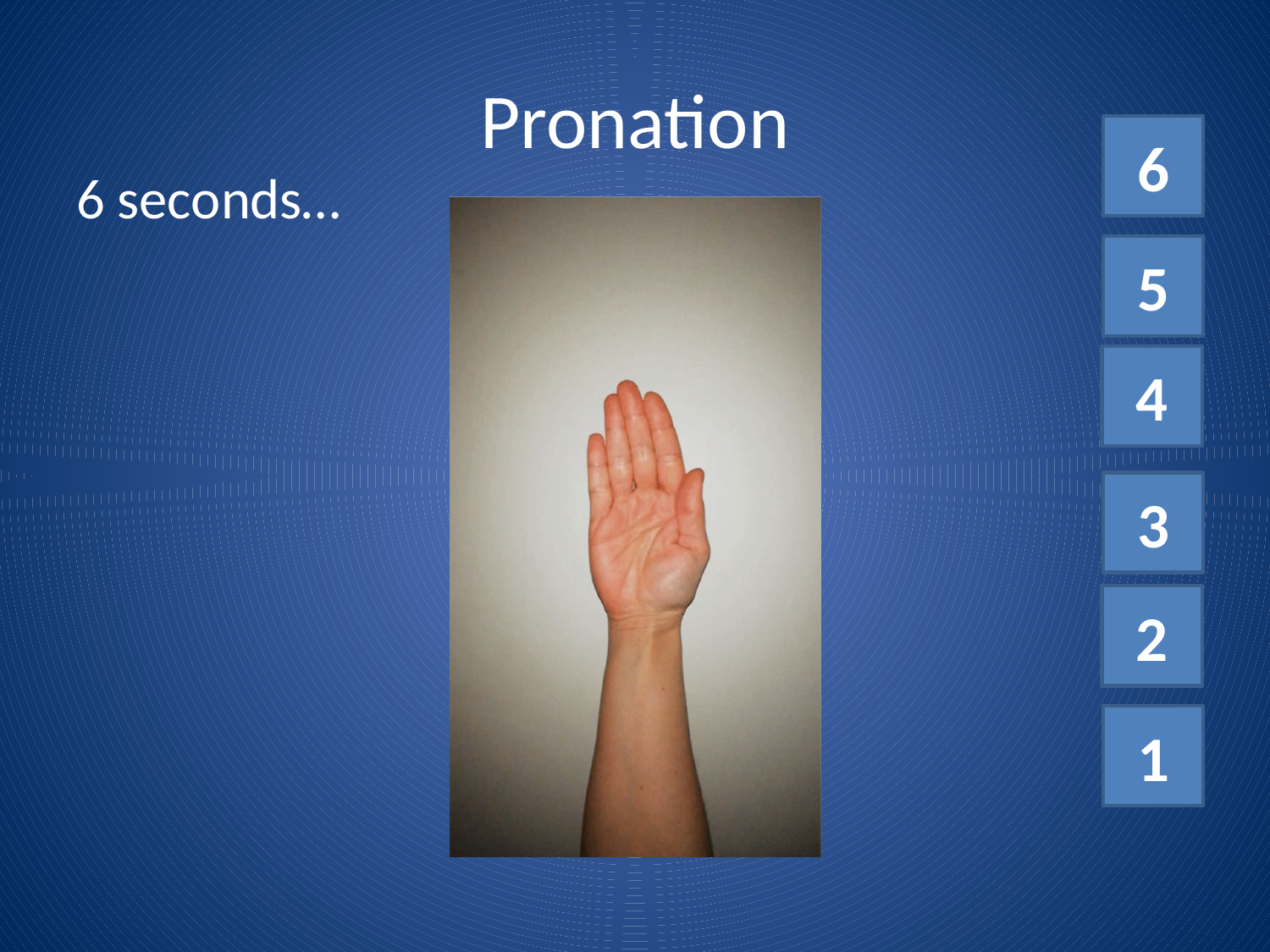

# Pronation
6
6 seconds…
5
4
3
2
1

## Slide 114
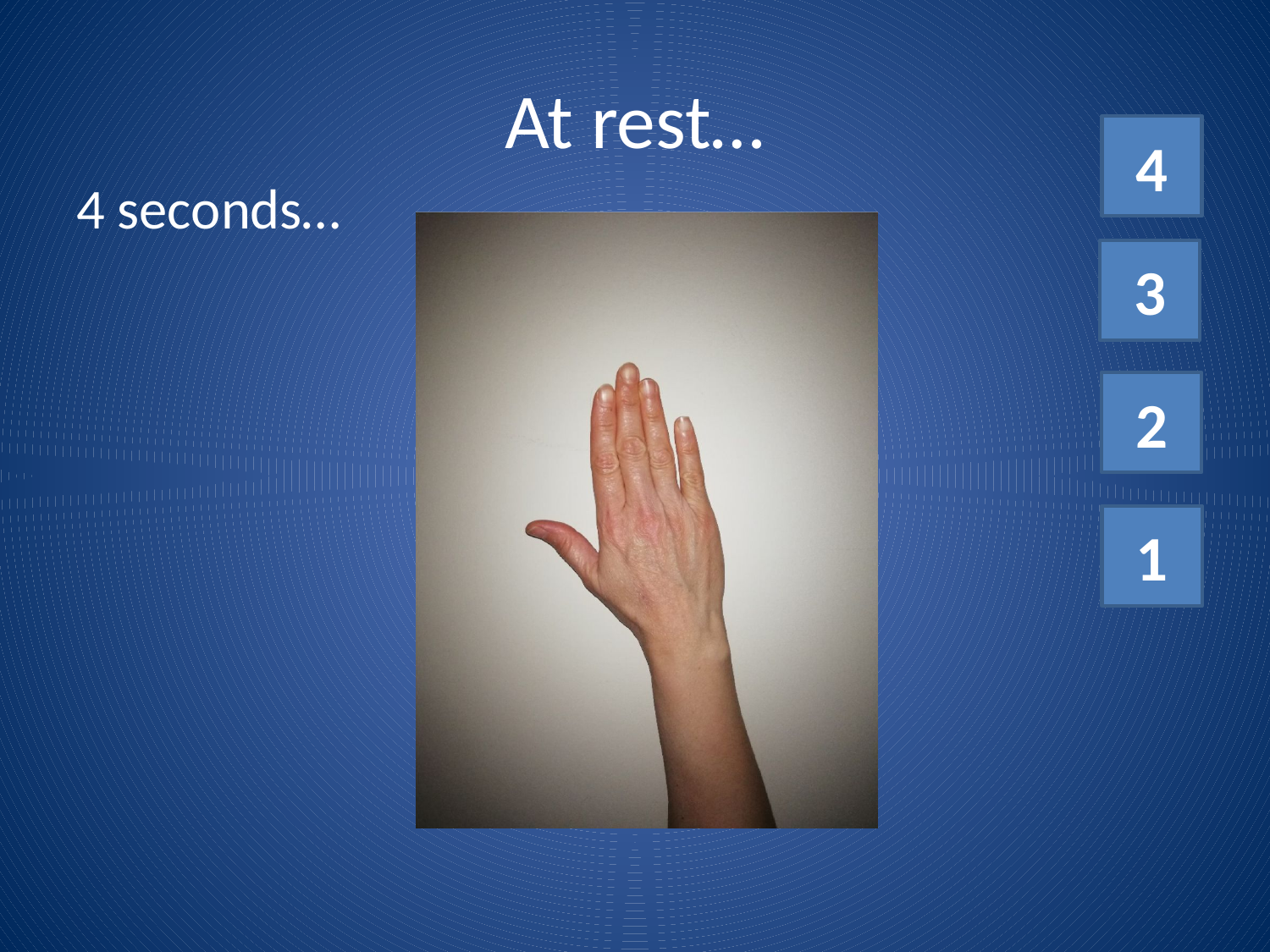

# At rest…
4
4 seconds…
3
2
1

## Slide 115
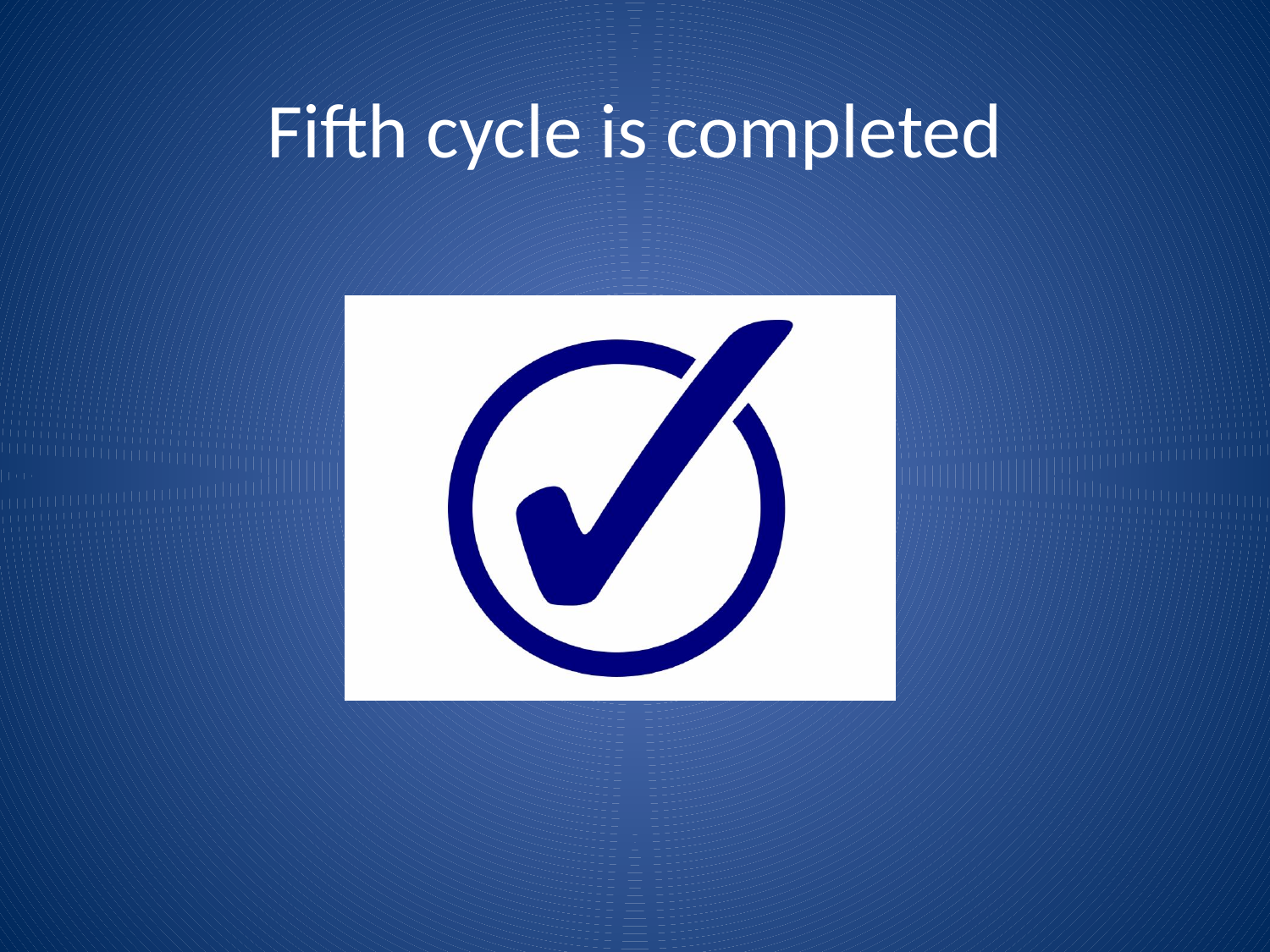

# Fifth cycle is completed

## Slide 116
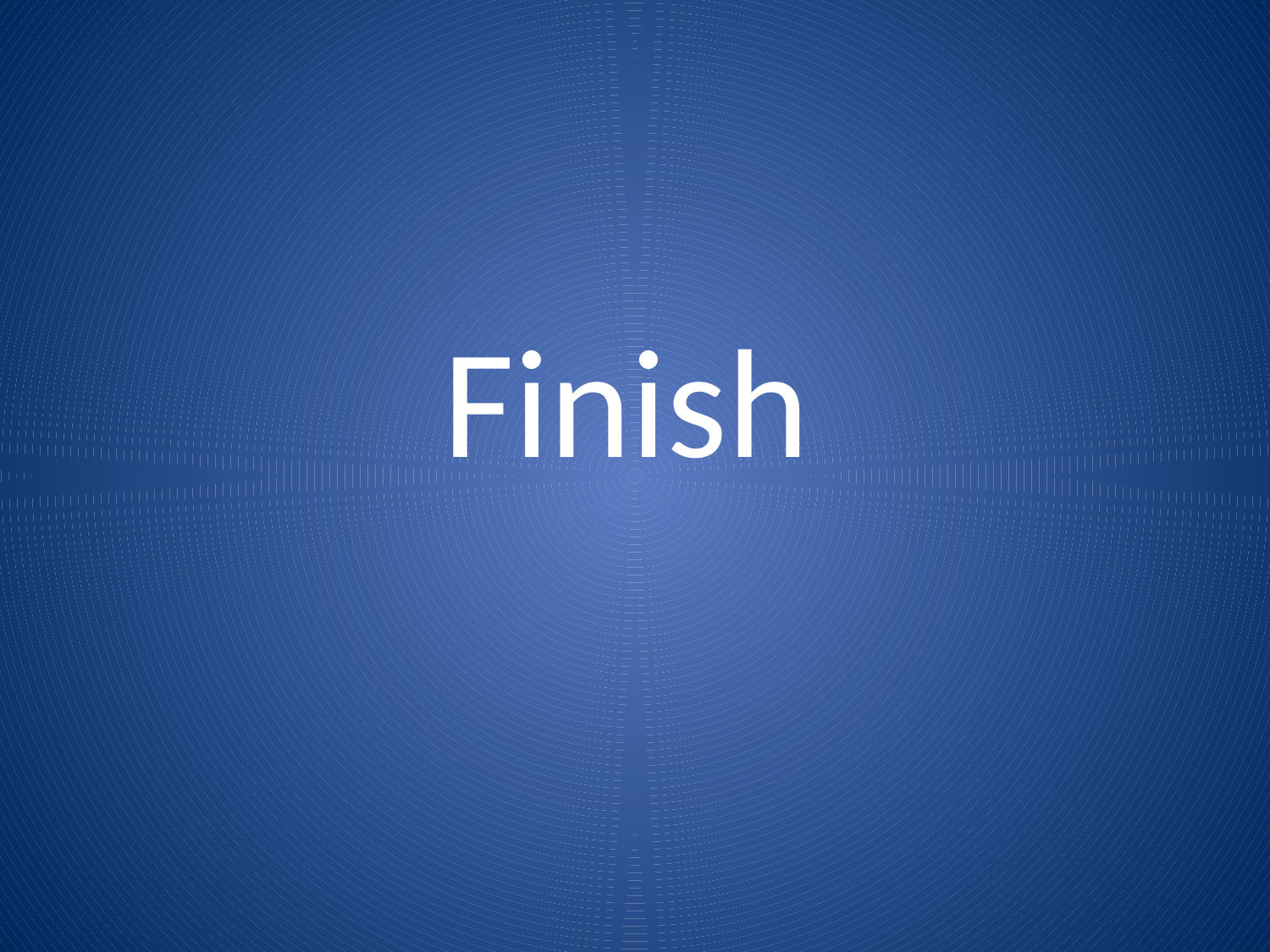

# Finish
