# Supplementary material for: Dataset for multi-channel surface electromyography (sEMG) signals of hand gestures
Source: Data Brief. 2022 Feb 4;41:107921. doi: 10.1016/j.dib.2022.107921 (PMC8844426; doi:10.1016/j.dib.2022.107921)
Supplement: Supplementary file 4 [file mmc4.docx]

| **Algorithm 1: sEMG Signal Segmentation** |
| --- |
| **Input:** 4-channel EMG data  **Output:** signal segments of the gesture moments  **Initialization:**  **set** *participant* count **to** 40  **set** *repetition* count **to** 5  **set** *gesture* count **to** 10  **set** *channel* count **to** 4  **set** *sample rate* **to** 2000 hertz  **set** *segment length*  **get** EMG data file locations from disk  **for** *all participant* **do**  **load** current participant EMG data from disk  **for** *all* *repetition* **do**  **calculate** current repetition coefficient  **for** *all* *gesture* **do**  **if** *multi-channel analysis is to be used* **then**  **calculate** *index* of current gesture  **compute** segmentation of *multi-channel EMG signal* of the current *gesture*  according to the specified *segment length*  **switch** *gesture*  **case** *rest* **then**  **compute** desired multivariate analysis for rest segments  **case** *extension* **then**  **compute** desired multivariate analysis for extension segments  **case** *flexion* **then**  **compute** desired multivariate analysis for flexion segments  **case** *ulnar deviation* **then**  **compute** desired multivariate analysis for ulnar deviation segments  **case** *radial deviation* **then**  **compute** desired multivariate analysis for radial deviation segments  **case** *grip* **then**  **compute** desired multivariate analysis for grip segments  **case** *abduction* **then**  **compute** desired multivariate analysis for abduction segments  **case** *adduction* **then**  **compute** desired multivariate analysis for adduction segments  **case** *supination* **then**  **compute** desired multivariate analysis for supination segments  **case** *pronation* **then**  **compute** desired multivariate analysis for pronation segments  **endswitch**  **elseif** *single-channel analysis is to be used* **then**  **for** *all channel* **do**  **calculate** *index* of current gesture  **compute** segmentation of *single-channel EMG signal* of the current *gesture*  according to the specified *segment length*  **switch** *gesture*  **case** *rest* **then**  **compute** desired single-channel analysis for rest segment  **case** *extension* **then**  **compute** desired single-channel analysis for extension segment  **case** *flexion* **then**  **compute** desired single-channel analysis for flexion segment  **case** *ulnar deviation* **then**  **compute** desired single-channel analysis for ulnar deviation segment  **case** *radial deviation* **then**  **compute** desired single-channel analysis for radial deviation segment  **case** *grip* **then**  **compute** desired single-channel analysis for grip segment  **case** *abduction* **then**  **compute** desired single-channel analysis for abduction segment  **case** *adduction* **then**  **compute** desired single-channel analysis for adduction segment  **case** *supination* **then**  **compute** desired single-channel analysis for supination segment  **case** *pronation* **then**  **compute** desired single-channel analysis for pronation segment  **endswitch**  **endfor**  **endif**  **endfor**  **endfor**  **endfor**  Remove all the related combinations of EMG data from memory |
